# Genetic variants for head size share genes and pathways with cancer

**DOI:** 10.1016/j.xcrm.2024.101529

**Published:** 2024-05-03

**Authors:** Maria J. Knol, Raymond A. Poot, Tavia E. Evans, Claudia L. Satizabal, Aniket Mishra, Muralidharan Sargurupremraj, Sandra van der Auwera, Marie-Gabrielle Duperron, Xueqiu Jian, Isabel C. Hostettler, Dianne H.K. van Dam-Nolen, Sander Lamballais, Mikolaj A. Pawlak, Cora E. Lewis, Amaia Carrion-Castillo, Theo G.M. van Erp, Céline S. Reinbold, Jean Shin, Markus Scholz, Asta K. Håberg, Anders Kämpe, Gloria H.Y. Li, Reut Avinun, Joshua R. Atkins, Fang-Chi Hsu, Alyssa R. Amod, Max Lam, Ami Tsuchida, Mariël W.A. Teunissen, Nil Aygün, Yash Patel, Dan Liang, Alexa S. Beiser, Frauke Beyer, Joshua C. Bis, Daniel Bos, R. Nick Bryan, Robin Bülow, Svenja Caspers, Gwenaëlle Catheline, Charlotte A.M. Cecil, Shareefa Dalvie, Jean-François Dartigues, Charles DeCarli, Maria Enlund-Cerullo, Judith M. Ford, Barbara Franke, Barry I. Freedman, Nele Friedrich, Melissa J. Green, Simon Haworth, Catherine Helmer, Per Hoffmann, Georg Homuth, M. Kamran Ikram, Clifford R. Jack, Neda Jahanshad, Christiane Jockwitz, Yoichiro Kamatani, Annchen R. Knodt, Shuo Li, Keane Lim, W.T. Longstreth, Fabio Macciardi, The Cohorts for Heart and Aging Research in Genomic Epidemiology (CHARGE) Consortium, Philippe Amouyel, Konstantinos Arfanakis, Benjamin S. Aribisala, Mark E. Bastin, Ganesh Chauhan, Christopher Chen, Ching-Yu Cheng, Philip L. de Jager, Ian J. Deary, Debra A. Fleischman, Rebecca F. Gottesman, Vilmundur Gudnason, Saima Hilal, Edith Hofer, Deborah Janowitz, J. Wouter Jukema, David C.M. Liewald, Lorna M. Lopez, Oscar Lopez, Michelle Luciano, Oliver Martinez, Wiro J. Niessen, Paul Nyquist, Jerome I. Rotter, Tatjana Rundek, Ralph L. Sacco, Helena Schmidt, Henning Tiemeier, Stella Trompet, Jeroen van der Grond, Henry Völzke, Joanna M. Wardlaw, Lisa Yanek, Jingyun Yang, The Enhancing NeuroImaging Genetics through Meta-Analysis (ENIGMA) Consortium, Ingrid Agartz, Saud Alhusaini, Laura Almasy, David Ames, Katrin Amunts, Ole A. Andreassen, Nicola Armstrong, Manon Bernard, John Blangero, Laura M.E. Blanken, Marco P. Boks, Dorret I. Boomsma, Adam M. Brickman, Henry Brodaty, Randy L. Buckner, Jan K. Buitelaar, Dara M. Cannon, Vaughan J. Carr, Stanley V. Catts, M. Mallar Chakravarty, Qiang Chen, Christopher R.K. Ching, Aiden Corvin, Benedicto Crespo-Facorro, Joanne E. Curran, Gareth E. Davies, Eco J.C. de Geus, Greig I. de Zubicaray, Anouk den Braber, Sylvane Desrivières, Allissa Dillman, Srdjan Djurovic, Wayne C. Drevets, Ravi Duggirala, Stefan Ehrlich, Susanne Erk, Thomas Espeseth, Iryna O. Fedko, Guillén Fernández, Simon E. Fisher, Tatiana M. Foroud, Tian Ge, Sudheer Giddaluru, David C. Glahn, Aaron L. Goldman, Robert C. Green, Corina U. Greven, Oliver Grimm, Narelle K. Hansell, Catharina A. Hartman, Ryota Hashimoto, Andreas Heinz, Frans Henskens, Derrek P. Hibar, Beng-Choon Ho, Pieter J. Hoekstra, Avram J. Holmes, Martine Hoogman, Jouke-Jan Hottenga, Hilleke E. Hulshoff Pol, Assen Jablensky, Mark Jenkinson, Tianye Jia, Karl-Heinz Jöckel, Erik G. Jönsson, Sungeun Kim, Marieke Klein, Peter Kochunov, John B. Kwok, Stephen M. Lawrie, Stephanie Le Hellard, Hervé Lemaître, Carmel Loughland, Andre F. Marquand, Nicholas G. Martin, Jean-Luc Martinot, Mar Matarin, Daniel H. Mathalon, Karen A. Mather, Venkata S. Mattay, Colm McDonald, Francis J. McMahon, Katie L. McMahon, Rebekah E, McWhirter, Patrizia Mecocci, Ingrid Melle, Andreas Meyer-Lindenberg, Patricia T. Michie, Yuri Milaneschi, Derek W. Morris, Bryan Mowry, Kwangsik Nho, Thomas E. Nichols, Markus N. Nöthen, Rene L. Olvera, Jaap Oosterlaan, Roel A. Ophoff, Massimo Pandolfo, Christos Pantelis, Irene Pappa, Brenda Penninx, G. Bruce Pike, Paul E. Rasser, Miguel E. Rentería, Simone Reppermund, Marcella Rietschel, Shannon L. Risacher, Nina Romanczuk-Seiferth, Emma Jane Rose, Perminder S. Sachdev, Philipp G. Sämann, Andrew J. Saykin, Ulrich Schall, Peter R. Schofield, Sara Schramm, Gunter Schumann, Rodney Scott, Li Shen, Sanjay M. Sisodiya, Hilkka Soininen, Emma Sprooten, Velandai Srikanth, Vidar M. Steen, Lachlan T. Strike, Anbupalam Thalamuthu, Arthur W. Toga, Paul Tooney, Diana Tordesillas-Gutiérrez, Jessica A. Turner, Maria del C. Valdés Hernández, Dennis van der Meer, Nic J.A. Van der Wee, Neeltje E.M. Van Haren, Dennis van 't Ent, Dick J. Veltman, Henrik Walter, Daniel R. Weinberger, Michael W. Weiner, Wei Wen, Lars T. Westlye, Eric Westman, Anderson M. Winkler, Girma Woldehawariat, Margaret J. Wright, Jingqin Wu, Outi Mäkitie, Bernard Mazoyer, Sarah E. Medland, Susumu Miyamoto, Susanne Moebus, Thomas H. Mosley, Ryan Muetzel, Thomas W. Mühleisen, Manabu Nagata, Soichiro Nakahara, Nicholette D. Palmer, Zdenka Pausova, Adrian Preda, Yann Quidé, William R. Reay, Gennady V. Roshchupkin, Reinhold Schmidt, Pamela J. Schreiner, Kazuya Setoh, Chin Yang Shapland, Stephen Sidney, Beate St Pourcain, Jason L. Stein, Yasuharu Tabara, Alexander Teumer, Anne Uhlmann, Aad van der Lugt, Meike W. Vernooij, David J. Werring, B. Gwen Windham, A. Veronica Witte, Katharina Wittfeld, Qiong Yang, Kazumichi Yoshida, Han G. Brunner, Quentin Le Grand, Kang Sim, Dan J. Stein, Donald W. Bowden, Murray J. Cairns, Ahmad R. Hariri, Ching-Lung Cheung, Sture Andersson, Arno Villringer, Tomas Paus, Sven Cichon, Vince D. Calhoun, Fabrice Crivello, Lenore J. Launer, Tonya White, Peter J. Koudstaal, Henry Houlden, Myriam Fornage, Fumihiko Matsuda, Hans J. Grabe, M. Arfan Ikram, Stéphanie Debette, Paul M. Thompson, Sudha Seshadri, Hieab H.H. Adams

**Affiliations:** 1 Department of Epidemiology, Erasmus MC University Medical Center, Rotterdam, the Netherlands; 2 Department of Cell Biology, Erasmus MC University Medical Center, Rotterdam, the Netherlands; 3 Department of Clinical Genetics, Erasmus MC University Medical Center, Rotterdam, the Netherlands; 4 Department of Radiology and Nuclear Medicine, Erasmus MC University Medical Center, Rotterdam, the Netherlands; 5 Glenn Biggs Institute for Alzheimer’s & Neurodegenerative Diseases, UT Health San Antonio, San Antonio, TX, USA; 6 The Framingham Heart Study, Framingham, MA, USA; 7 Department of Neurology, Boston University School of Medicine, Boston, MA, USA; 8 University of Bordeaux, Inserm, Bordeaux Population Health Research Center, team VINTAGE, UMR 1219, Bordeaux, France; 9 Department of Psychiatry and Psychotherapy, University Medicine Greifswald, Greifswald, Germany; 10 German Centre of Neurodegenerative Diseases (DZNE), Site Rostock/Greifswald, Greifswald, Germany; 11 Brown Foundation Institute of Molecular Medicine, McGovern Medical School, University of Texas Health Science Center at Houston, Houston, TX, USA; 12 Stroke Research Centre, University College London, Institute of Neurology, London, UK; 13 Department of Neurosurgery, Klinikum rechts der Isar, University of Munich, Munich, Germany; 14 Neurosurgical Department, Cantonal Hospital St. Gallen, St. Gallen, Switzerland; 15 Department of Neurology, Poznań University of Medical Sciences, Poznań, Poland; 16 Department of Human Genetics, Radboud University Medical Center, Nijmegen, the Netherlands; 17 Department of Epidemiology, School of Public Health, University of Alabama at Birmingham School of Medicine, Birmingham, AL, USA; 18 Language and Genetics Department, Max Planck Institute for Psycholinguistics, Nijmegen, the Netherlands; 19 Clinical Translational Neuroscience Laboratory, Department of Psychiatry and Human Behavior, University of California, Irvine, Irvine, CA, USA; 20 Center for the Neurobiology of Learning and Memory, University of California, Irvine, Irvine, CA, USA; 21 Department of Biomedicine, University of Basel, Basel, Switzerland; 22 Institute of Medical Genetics and Pathology, University Hospital Basel, Basel, Switzerland; 23 Institute of Computational Life Sciences, Zurich University of Applied Sciences, Wädenswil, Switzerland; 24 The Hospital for Sick Children, University of Toronto, Toronto, Canada; 25 Departments of Physiology and Nutritional Sciences, University of Toronto, Toronto, Canada; 26 Institute for Medical Informatics, Statistics and Epidemiology, University of Leipzig, Leipzig, Germany; 27 LIFE Research Center for Civilization Disease, Leipzig, Germany; 28 Department of Neuromedicine and Movement Science, Norwegian University of Science and Technology (NTNU), Trondheim, Norway; 29 Department of Radiology and Nuclear Medicine, St. Olavs University Hospital, Trondheim, Norway; 30 Department of Molecular Medicine and Surgery, Karolinska Institutet, Stockholm, Sweden; 31 Department of Clinical Genetics, Karolinska University Hospital, Stockholm, Sweden; 32 Department of Pharmacology and Pharmacy, Li Ka Shing Faculty of Medicine, The University of Hong Kong, Hong Kong, China; 33 Laboratory of NeuroGenetics, Department of Psychology & Neuroscience, Duke University, Durham, NC, USA; 34 School of Biomedical Sciences and Pharmacy, The University of Newcastle, Callaghan, NSW, Australia; 35 Centre for Brain and Mental Health Research, Hunter Medical Research Institute, Newcastle, NSW, Australia; 36 Department of Biostatistics and Data Science, Wake Forest University School of Medicine, Winston-Salem, NC, USA; 37 Department of Child and Adolescent Psychiatry, TU Dresden, Dresden, Germany; 38 North Region, Institute of Mental Health, Singapore, Singapore; 39 Population and Global Health, LKC Medicine, Nanyang Technological University, Singapore, Singapore; 40 Groupe d’imagerie neurofonctionnelle, Institut des Maladies Neurodégénératives, UMR 5293, CNRS, CEA, Université de Bordeaux, Bordeaux, France; 41 Department of Neurology, Maastricht University Medical Center+, Maastricht, the Netherlands; 42 Department of Genetics UNC Neuroscience Center, University of North Carolina at Chapel Hill, Chapel Hill, NC, USA; 43 Institute of Medical Sciences, University of Toronto, Toronto, ON, Canada; 44 Department of Biostatistics, Boston University School of Public Health, Boston, MA, USA; 45 Department of Neurology, Max Planck Institute for Cognitive and Brain Sciences, Leipzig, Germany; 46 Collaborative Research Center 1052 Obesity Mechanisms, Faculty of Medicine, University of Leipzig, Leipzig, Germany; 47 Day Clinic for Cognitive Neurology, University Hospital Leipzig, Leipzig, Germany; 48 Cardiovascular Health Research Unit, Department of Medicine, University of Washington, Seattle, WA, USA; 49 Department of Radiology, University of Pennsylvania, Philadelphia, PA, USA; 50 Institute of Diagnostic Radiology and Neuroradiology, University Medicine Greifswald, Greifswald, Germany; 51 Institute of Neuroscience and Medicine (INM-1), Research Centre Jülich, Jülich, Germany; 52 Institute for Anatomy I, Medical Faculty & University Hospital Düsseldorf, Heinrich Heine University Düsseldorf, Düsseldorf, Germany; 53 University of Bordeaux, CNRS, INCIA, UMR 5287, team NeuroImagerie et Cognition Humaine, Bordeaux, France; 54 EPHE-PSL University, Bordeaux, France; 55 Department of Child and Adolescent Psychiatry, Erasmus MC University Medical Center, Rotterdam, the Netherlands; 56 University of Bordeaux, Inserm, Bordeaux Population Health Research Center, team SEPIA, UMR 1219, Bordeaux, France; 57 Department of Neurology and Center for Neuroscience, University of California at Davis, Sacramento, CA, USA; 58 Children’s Hospital, University of Helsinki and Helsinki University Hospital, Helsinki, Finland; 59 Folkhälsan Research Center, Helsinki, Finland; 60 San Francisco Veterans Administration Medical Center, San Francisco, CA, USA; 61 University of California, San Francisco, San Francisco, CA, USA; 62 Department of Psychiatry, Radboud University Medical Center, Nijmegen, the Netherlands; 63 Donders Institute for Brain, Cognition, and Behaviour, Radboud University, Nijmegen, the Netherlands; 64 Department of Internal Medicine, Section on Nephrology, Wake Forest School of Medicine, Winston-Salem, NC, USA; 65 Institute of Clinical Chemistry and Laboratory Medicine, University Medicine Greifswald, Greifswald, Germany; 66 School of Clinical Medicine, University of New South Wales, Sydney, NSW, Australia; 67 Neuroscience Research Australia, Sydney, NSW, Australia; 68 Bristol Dental School, University of Bristol, Bristol, UK; 69 University of Bordeaux, Inserm, Bordeaux Population Health Research Center, team LEHA, UMR 1219, Bordeaux, France; 70 Institute of Human Genetics, University of Bonn Medical School, Bonn, Germany; 71 Interfaculty Institute for Genetics and Functional Genomics, University Medicine Greifswald, Greifswald, Germany; 72 Department of Neurology, Erasmus MC University Medical Center, Rotterdam, the Netherlands; 73 Department of Radiology, Mayo Clinic, Rochester, MN, USA; 74 Imaging Genetics Center, Mark & Mary Stevens Neuroimaging & Informatics Institute, Keck USC School of Medicine, Los Angeles, CA, USA; 75 Department of Psychiatry, Psychotherapy and Psychosomatics, RWTH Aachen University, Medical Faculty, Aachen, Germany; 76 Center for Genomic Medicine, Kyoto University Graduate School of Medicine, Kyoto, Japan; 77 Research Division, Institute of Mental Health, Singapore, Singapore; 78 Department of Neurology, University of Washington, Seattle, WA, USA; 79 Department of Epidemiology, University of Washington, Seattle, WA, USA; 80 Laboratory of Molecular Psychiatry, Department of Psychiatry and Human Behavior, School of Medicine, University of California, Irvine, Irvine, CA, USA; 81 Centre Hospitalo-Universitaire de Bordeaux, Bordeaux, France; 82 Psychiatric Genetics, QIMR Berghofer Medical Research Institute, Brisbane, QLD, Australia; 83 School of Psychology, University of Queensland, Brisbane, QLD, Australia; 84 Faculty of Medicine, University of Queensland, Brisbane, QLD, Australia; 85 Department of Neurosurgery, Kyoto University Graduate School of Medicine, Kyoto, Japan; 86 Institute for Urban Public Health, University of Duisburg-Essen, Essen, Germany; 87 Department of Medicine, Division of Geriatrics, University of Mississippi Medical Center, Jackson, MS, USA; 88 Memory Impairment and Neurodegenerative Dementia (MIND) Center, Jackson, MS, USA; 89 C. and O. Vogt Institute for Brain Research, Medical Faculty, Heinrich Heine University Düsseldorf, Düsseldorf, Germany; 90 Unit 2, Candidate Discovery Science Labs, Drug Discovery Research, Astellas Pharma Inc, 21 Miyukigaoka, Tsukuba, Ibaraki 305-8585, Japan; 91 Department of Biochemistry, Wake Forest School of Medicine, Winston-Salem, NC, USA; 92 Department of Psychiatry, University of California, Irvine, Irvine, CA, USA; 93 Clinical Division of Neurogeriatrics, Department of Neurology, Medical University of Graz, Graz, Austria; 94 University of Minnesota School of Public Health, Minneapolis, MN, USA; 95 MRC Integrative Epidemiology Unit, University of Bristol, Bristol, UK; 96 Population Health Sciences, University of Bristol, Bristol, UK; 97 Kaiser Permanente Division of Research, Oakland, CA, USA; 98 Institute for Community Medicine, University Medicine Greifswald, Greifswald, Germany; 99 Department of Human Genetics, Donders Institute for Brain, Cognition, and Behaviour, Radboud University Medical Center, Nijmegen, the Netherlands; 100 Department of Clinical Genetics MUMC+, GROW School of Oncology and Developmental Biology, and MHeNs School of Mental Health and Neuroscience, Maastricht University, Maastricht, the Netherlands; 101 Bordeaux Population Health, University of Bordeaux, INSERM U1219, Bordeaux, France; 102 West Region, Institute of Mental Health, Singapore, Singapore; 103 Yong Loo Lin School of Medicine, National University of Singapore, Singapore, Singapore; 104 Lee Kong Chian School of Medicine, Nanyang Technological University, Singapore, Singapore; 105 SAMRC Unit on Risk and Resilience, University of Cape Town, Cape Town, South Africa; 106 Centre for Genomic Sciences, Li Ka Shing Faculty of Medicine, The University of Hong Kong, Hong Kong, China; 107 Department of Medicine, Li Ka Shing Faculty of Medicine, The University of Hong Kong, Hong Kong, China; 108 Departments of Psychiatry and Neuroscience, Faculty of Medicine and Centre Hospitalier Universitaire Sainte-Justine, University of Montreal, Montreal, QC, Canada; 109 Department of Psychiatry, Faculty of Medicine, McGill University, Montreal, QC, Canada; 110 Tri-institutional Center for Translational Research in Neuroimaging and Data Science (TReNDS) {Georgia State, Georgia Tech, Emory}, Atlanta, GA, USA; 111 Laboratory of Epidemiology, Demography, and Biometry, Intramural Research Program, National Institute of Aging, The National Institutes of Health, Bethesda, MD, USA; 112 Human Genetics Center, School of Public Health, University of Texas Health Science Center at Houston, Houston, TX, USA; 113 Department of Neurology, Bordeaux University Hospital, Bordeaux, France; 114 Latin American Brain Health (BrainLat), Universidad Adolfo Ibáñez, Santiago, Chile

**Keywords:** genetics, genome-wide association study, head size, intracranial volume, head circumference, cancer, meta-analysis

## Abstract

The size of the human head is highly heritable, but genetic drivers of its variation within the general population remain unmapped. We perform a genome-wide association study on head size (*N* = 80,890) and identify 67 genetic loci, of which 50 are novel. Neuroimaging studies show that 17 variants affect specific brain areas, but most have widespread effects. Gene set enrichment is observed for various cancers and the p53, Wnt, and ErbB signaling pathways. Genes harboring lead variants are enriched for macrocephaly syndrome genes (37-fold) and high-fidelity cancer genes (9-fold), which is not seen for human height variants. Head size variants are also near genes preferentially expressed in intermediate progenitor cells, neural cells linked to evolutionary brain expansion. Our results indicate that genes regulating early brain and cranial growth incline to neoplasia later in life, irrespective of height. This warrants investigation of clinical implications of the link between head size and cancer.

## Highlights

• Knol, Poot, et al. identify 67 genetic loci associated with human head size
• Genes harboring or near head size genetic variants enrich for macrocephaly genes
• Head size genetic variants preferentially locate to cancer genes and pathways
• Further research is needed on the potential link between head size and cancer risk

## Graphical abstract

**Figure:**
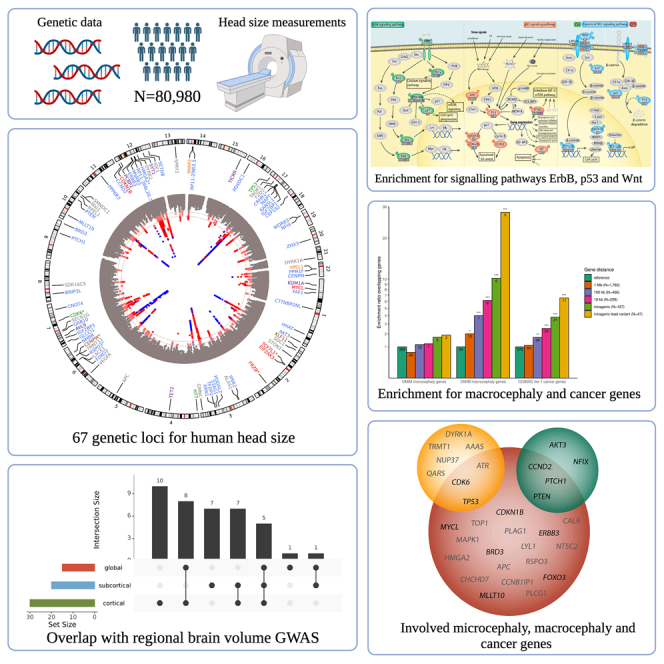

## Teaser

Knol, Poot, et al. identify 67 loci for human head size in a genome-wide association study. Genes harboring the lead variants enrich for cancer genes and pathways, which was not seen for height variants. These findings suggest a potential link between a larger head and a higher cancer risk.

## Introduction

The size of the human head, measured by head circumference or intracranial volume, correlates closely with brain size. Head size is determined by growth in the first years of life and is largely completed by 6 years of age, whereas the rest of the body typically grows until early adulthood.^1^ Head size is highly genetically determined, ranging from near 90% during childhood to 75% during adulthood.^2^ Rare genetic syndromes have revealed individual genes strongly affecting head size.^3^ Nevertheless, genetic determinants of its variation within the general population are still poorly characterized, with no coherent and well-supported picture of associated biological pathways.

A previous genome-wide association study (GWAS) on 47,000 individuals identified 18 genetic loci for intracranial volume,^4^ while another GWAS on head size in 46,000 children and adults identified 17 loci for head size including low-frequency variants in *TP53*.^5^ Here, we increased the sample size to a total GWAS discovery sample size of 80,890 individuals, and validated the results in an independent sample of 25,088 individuals. Our GWAS analyses show strong enrichment for genes and multiple pathways involved in cancer, macrocephaly genes, and show preferential expression of genes near variants in intermediate progenitor cells.

## Results

**Figure 1:**
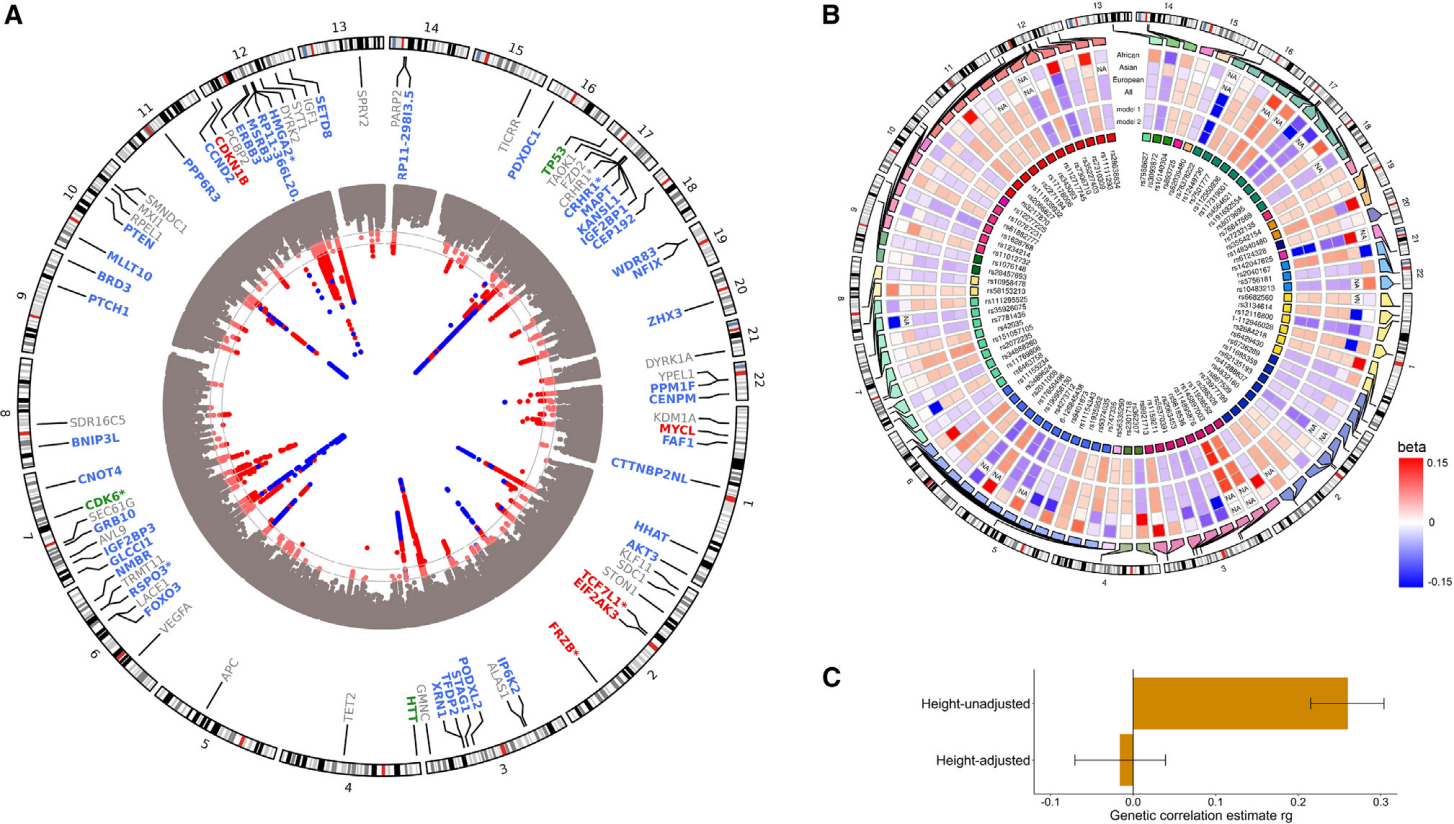
Genome-wide association studies on human head size (A) Circos Manhattan plot of the European ancestry head size GWAS, with gray lines corresponding to genome-wide significant (*p* < 5 × 10^−8^) or sub-significant (*p* < 1 × 10^−6^) *p* value thresholds. Known variants are in blue, novel ones in red. For each lead variant, the nearest gene is presented, with the color corresponding to its position to the lead variant: exonic (red), 3′-UTR (green), intronic (blue), intergenic including up- and downstream, exonic and intronic non-coding RNA (gray). Nearest genes for more than one locus are denoted with an asterisk (∗). (B) Circos heatmap showing the betas of lead variants in African, Asian, and European ancestry meta-analyses, as well as the transancestral meta-analysis. Differences between the height-unadjusted (model 1) and -adjusted (model 2) meta-analysis are also shown. (C) Bar plot of the genetic correlation coefficient (ρ_genetic_) of the height-unadjusted and -adjusted head size GWAS with the height GWAS, with their accompanying 95% confidence intervals.

We performed a meta-analysis of GWASs for head size, as proxied by intracranial volume from brain imaging, or head circumference (Tables S1–S3 and S4; STAR Methods). Compared with previous efforts,^5^^,^^6^ we nearly doubled the sample size (*N* = 80,890), in majority from European ancestry (*N* = 75,309). We identified 90 independent genetic variants in 67 loci associated with human head size in the European sample (Figure 1A; Table S6. Lead genetic variants and their effects on human head size in samples of different ethnicities, with and without adjustment for height. Related to Figure 1B, Table S7. Genome-wide significant genetic variants and variants in linkage disequilibrium (r2>0.6), including functional annotations. Related to Figure 1A and 1B, Table S8. Lead variants of the combined discovery and validation GWAS meta-analysis, related to Figure 1A and 1B; Data S1, S2, and S3), of which 50 loci were novel. Although the results showed some bias (linkage disequilibrium [LD] score regression intercept 1.056; Table S5), the identified variants remained genome-wide significant after correction for this amount of bias. Most variants (*N* = 48) showed consistent directions of association among the European, African (*N* = 1,356), and Asian (*N* = 4,225) ancestry samples (Figure 1B; Table S6), suggesting population-specific genetic effects on head size in these loci. Since we had limited non-European samples, we also tested the combined effect of the lead variants, which showed positive associations in African and East Asian ancestry samples (β_African_ = 0.34, confidence interval [CI] 0.08–0.60; β_East Asian_ = 0.40, CI 0.24–0.57). In the European validation sample (*N* = 25,088), 20 of the 89 lead variants were associated with head size at a Bonferroni significance level (*p* < 5.6 × 10^−4^) and 54 at a nominal significance level, while all lead variants showed the same direction of effect. In the UK Biobank validation sample (*N* = 23,046), the 89 available lead variants together explained 2.3% of the phenotypic head size variance. A meta-analysis combining the European discovery and validation sample (*N* = 101,241) identified 102 genomic loci with 126 lead variants (Table S8), of which 60 loci overlapped with the 67 genomic loci identified by the discovery meta-analysis.

### Head-specific growth vs. general growth

We investigated whether variants affecting head size are specific for growth of the human head or are driven, at least in part, by an effect on human body height. Accordingly, we performed a height-adjusted head size GWAS (*N* = 50,424). The genetic correlation between head size and height (ρ_genetic_ = 0.26, *p* = 2.1 × 10^−30^) disappeared in this model (ρ_genetic_ = −0.02, *p* = 0.58) (Figure 1C), confirming the removal of height-associated effects. Importantly, there was no significant reduction for any of the lead variants’ effect sizes with head size (Table S6). We further explored the effect of these variants on the size of other body parts using area measures obtained from bone density scans (*N* = 3,313). As expected, a polygenic score of the lead variants was associated with the skull area, even after adjusting for height (*p* = 2.1 × 10^−12^). One lead genetic variant (rs12277225) was significantly associated with the L1-L4 spine area (*p* = 1.3 × 10^−5^), but the other lead variants did not affect bone area measures of arm, leg, and spine (Table S9). Altogether, this indicates that the effect of the identified variants on head size is predominantly head-specific.

### Regional brain volumetric effects

**Figure 2:**
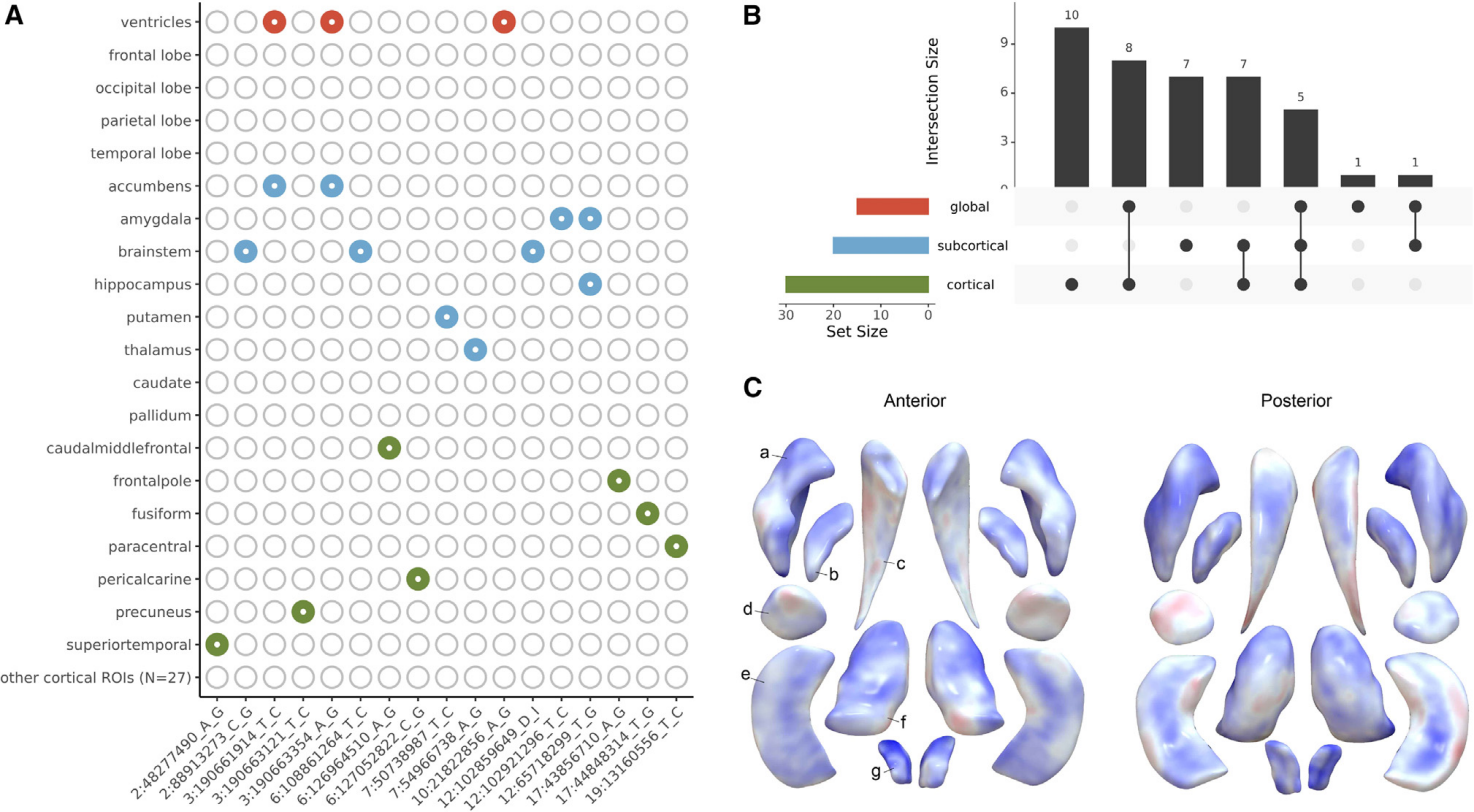
Genetic loci for head size and effects on regional brain volumes (A) Heatmap showing head size loci that overlap with previously identified loci for global brain volumes (red), subcortical volumes (blue), and cortical region of interest volumes (green). (B) UpSet plot of associations between head size lead variants and brain volumes. Intersection size corresponds to the frequency of the combination depicted below the bar. Set size corresponds to the frequency of associations with one of the brain volume categories (i.e., global, subcortical, or cortical). (C) Plot showing the subcortical shape analysis of rs111939932 using log Jacobian determinants. Colors correspond to t values, with positive associations depicted in blue, and negative ones in red. Letters point to different subcortical structures: a, putamen; b, pallidum; c, caudate; d, amygdala; e, hippocampus; f, thalamus; g, accumbens.

Head size may reflect growth of specific brain regions. Indeed, 15 lead genetic variants or variants in LD (r^2^ > 0.6) from 12 genetic loci were previously reported to affect volumes of subregions of the brain (Figure 2A; Table S10). We screened all loci previously associated with these regional brain volumes, and found 16 of those 132 loci significantly related with head size after multiple testing correction (Table S11). To determine if the current findings can be localized to specific brain regions, we investigated the 90 independent head size variants in relation to more fine-grained measures of brain morphometry—corrected for head size—in 22,145 individuals (Figure 2B; Table S12). Thirty-nine variants were associated with one or multiple cortical, subcortical, and global brain regions of which 17 variants were preferentially associated with one or two specific cortical or subcortical regions. For example, rs111939932, an intronic variant in *PCBP2*, is associated with nucleus accumbens volume and is an expression quantitative trait locus (eQTL) for several genes, including *ATP5G2* in the nucleus accumbens and basal ganglia. Further analysis revealed its localized effects on this structure’s shape (Figure 2C; Table S13). In the largest GWAS on nucleus accumbens volume,^7^ this variant was nominally significant (*p* = 0.02), showing the improved power of our current study to identify novel brain morphometry loci. For the other 51 variants there was no apparent association with particular brain regions. Overall, these results suggest that most head size variants affect generalized brain or cranial growth, while a minority influence regional brain growth.

### Genetic correlation with neuropsychiatric traits

Genetic correlation analyses with neuropsychiatric traits have been conducted previously.^5^^,^^6^ We replicated positive genetic correlations with cognitive functioning and Parkinson’s disease, also when only including new samples (Figure S1; Table S14). The replicated correlation with Parkinson’s disease provides independent evidence for the proposed brain overgrowth hypothesis in this disorder.^8^ Novel genetic correlations were found with multiple psychiatric traits; negative correlations with attention-deficit hyperactivity disorder (ρ_genetic_ = −0.18, *p* = 4.5 × 10^−7^), insomnia (ρ_genetic_ = −0.19, *p* = 1.8 × 10^−5^), major depressive disorder (ρ_genetic_ = −0.11, *p* = 2.6 × 10^−4^), and neuroticism (ρ_genetic_ = −0.11, *p* = 5.4 × 10^−4^) (Figure S1; Table S14). Since psychiatric disorders themselves are genetically correlated, incorporating head size and other brain anatomy traits could aid in disentangling underlying genetic factors.

### Pathway analysis

**Figure 3:**
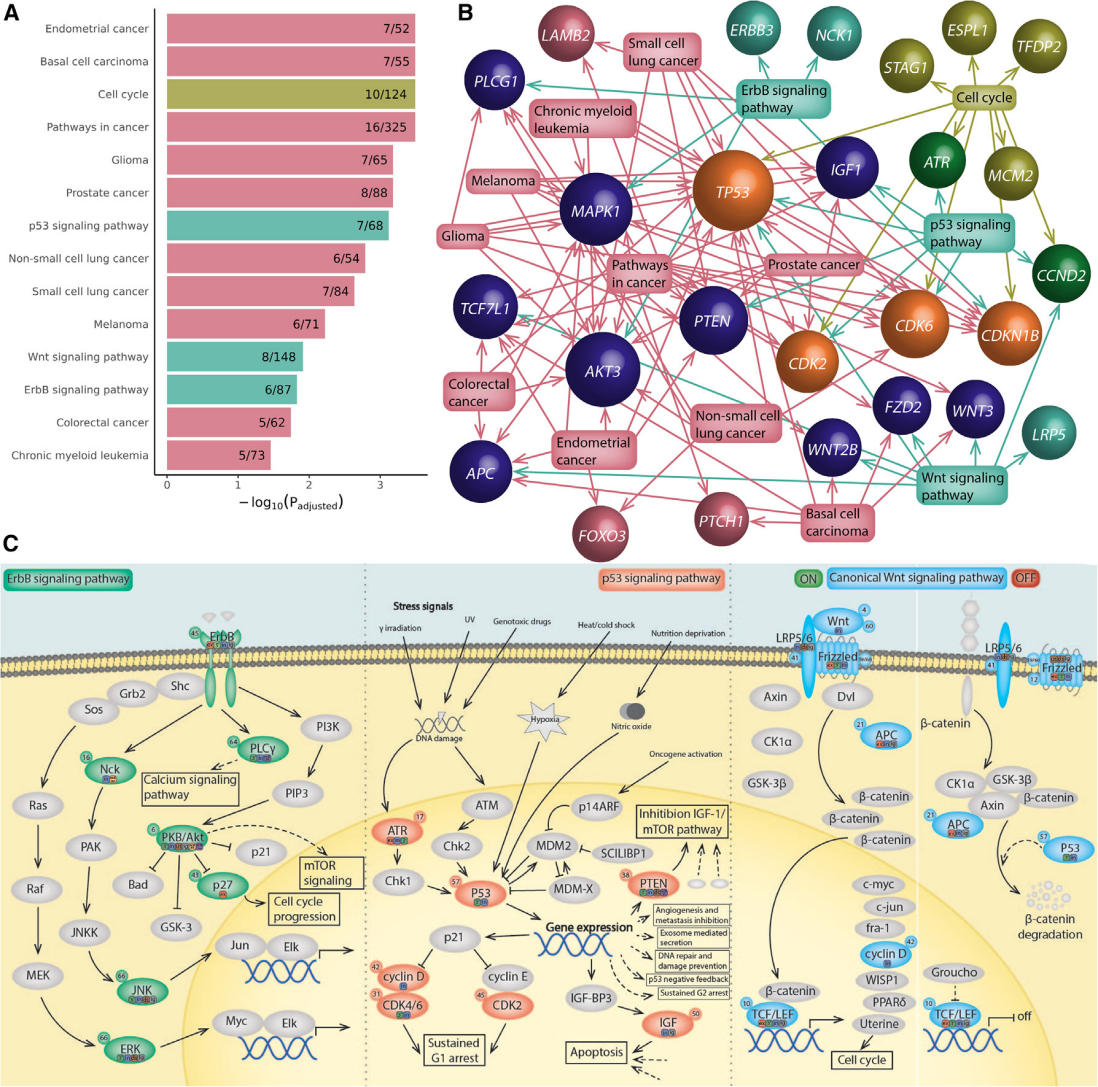
Gene sets enriched in human head size loci (A) Bar plots presenting enriched KEGG gene sets. –log_10_ of adjusted *p* value and proportion of nearby genes overlapping with the gene set are presented. Cancer gene sets are depicted in pink, cell growth and death gene sets in yellow-green, and signal transduction gene sets in turquoise. (B) Network graph showing enriched KEGG gene sets and their included genes near genetic lead variants. Gene sets are shown in squares with arrows to overlapping genes. Colors correspond to gene set categories: only cancer gene sets (pink), only cell growth and death gene sets (yellow-green), only signal transduction gene sets (turquoise), cancer gene sets and cell growth and death gene sets (dark blue), cell growth and death and signal transduction gene sets (green), or all three gene set categories (orange). Sphere size corresponds to the number of gene sets linked to that gene. (C) Schematic overview of enriched signaling pathways with proteins encoded by genes near (<10 kb) identified genetic loci. Proteins encoded by these genes are colored (green, ErbB pathway; red, p53 pathway; blue, Wnt pathway), other proteins are depicted in gray. Circles next to protein names provide the locus number of the encoding gene. Locations of lead variants and variants in LD (r^2^ > 0.6) are shown in squares next to the proteins: exonic (e; red), 3′-UTR (3′; green), 5′-UTR (5; light green), intronic (i; blue), intergenic including up- and downstream, exonic and intronic non-coding RNA (g; gray). For Frizzled, not only FZD2 but also FRZB is taken into consideration.

To obtain novel insights into the biological mechanisms underlying head size variation, we performed a gene set enrichment analysis of Kyoto Encyclopedia of Genes and Genomes (KEGG)^9^ gene sets and found 14 to be significantly enriched (Figure 3A; Table S15). Nine of those gene sets represent different cancer types that substantially overlap between each other and share underlying biological pathways (Figure 3B). The remaining gene sets represent the p53, Wnt, and ErbB signaling pathways, all involved in tumorigenesis including in the abovementioned cancer types.^10^ Remarkably, lead variants in our GWAS were predominantly intragenic for the seven genes in the p53 pathway, eight genes in the Wnt pathway, and six genes in the ErbB-EGFR pathway (Figure 3C), suggesting that modulation of these pathways plays an important role in head size variation.

The p53 signaling pathway showed the strongest enrichment (*p*_adjusted_ = 7.6 × 10^−4^) (Figure 3A; Table S15). Tumor suppressor protein p53, encoded by *TP53*, is activated by different stress signals to regulate the cell cycle and apoptosis. Our lead signal in this locus was *TP53* 3′-UTR variant rs78378222 with predicted deleterious effects (CADD = 15.93), which was identified previously.^5^ Three other genes in this pathway (*ATR*, *CDK6*, and *PTEN*) also contained 3′-UTR or exonic variants in LD (r^2^ > 0.6) with lead variants. Identified genes act in cell-cycle arrest and cellular senescence (*CDK6, CDK2*, and *CCND2*), apoptosis (*IGF1*), or inhibition of the insulin growth factor (IGF)-1/mammalian target of rapamycin (mTOR) pathway (*PTEN*), suggesting comprehensive involvement of the p53 signaling pathway in head growth. This finding is in line with evidence that p53 signaling regulates both normal and malignant neural stem cell populations.^11^^,^^12^^,^^13^

The Wnt signaling pathway has links to carcinogenesis and the developing and adult central nervous system,^14^^,^^15^ as well as to bone development including cranial growth.^16^ Of the eight overlapping genes, three contained exonic or 3′-UTR variants in LD (r^2^ > 0.6) with identified lead variants (*APC*, *TP53*, and *TCF7L1*). Wnt signaling pathway gene *FRZB*, not annotated in KEGG, also contained exonic and 3′-UTR variants. In total, 1,948 genetic variants in LD with the identified lead variants (r^2^ > 0.6), including 35 exonic variants, are eQTLs for *WNT3* in 27 different tissues including the cerebellar hemispheres. In addition, various exonic, 3′-UTR and 5′-UTR variants in LD with the lead variants are eQTLs for *TCF7L1* in brain tissues. These observations suggest that variants in this pathway affect brain and cranial growth in the human population.

The ErbB pathway (*p*_adjusted_ = 0.014, Figure 3A), also known as the EGFR signaling pathway, has six overlapping genes near head size variants, which are involved in calcium signaling (*PLCG1*), MAPK signaling (*NCK1* and *MAPK1*), and PI3K-AKT signaling (*ERBB3*, *AKT3*, and *CDKN1B*). In addition, five genetic variants are eQTLs for *EGFR* in the cerebellum. Interestingly, both *AKT3* and *CDKN1B* are linked to clinical head size syndromes and cancer risk^17^^,^^18^^,^^19^^,^^20^ and contain, respectively, 3′-UTR variants and an exonic variant that reach genome-wide significance. ErbB signaling is involved in neurodevelopment,^21^^,^^22^^,^^23^ making it a plausible pathway involved in head size variation.

Since the above signaling pathways also have universal roles in cell growth, we determined their enrichment in the height GWAS. We found that only the Wnt signaling pathway was significantly enriched in the height GWAS (*p*_adjusted_ = 0.038), suggesting that the p53 and ErbB signaling pathways are more specifically involved in head growth rather than generalized body growth.

### Functional prioritization using gene expression

Using a transcriptome-wide association study (TWAS), we identified 156 head size-associated variants functioning as eQTLs, regulating the expression of 112 genes (eGenes) in relevant tissue types (Table S16). Genomic overlap with additional gene-regulatory and epigenetic features provides evidence for 67 eQTLs regulating the expression of 58 eGenes (RegulomeDB probability score >0.5), including *AKT3* in brain tissue and *TCF7L1* in the cerebellum—part of the ErbB and Wnt pathway, respectively. In addition, 22 eGenes were suggested to be regulated by 22 splicing QTLs (sQTLs), including *AKT3*. The omnibus test revealed a shared effect for 80 eGenes across the tested gene expression panels (Table S17), including *WNT3*, *AKT3*, and *EGFR*.

### Enrichment of Mendelian head size genes and cancer genes

Target genes of GWAS variants are often close to the lead variant.^24^ Accordingly, we determined the enrichment of different categories of genes located nearby head size variants, stratified by their distance (Table S18).

**Figure 4:**
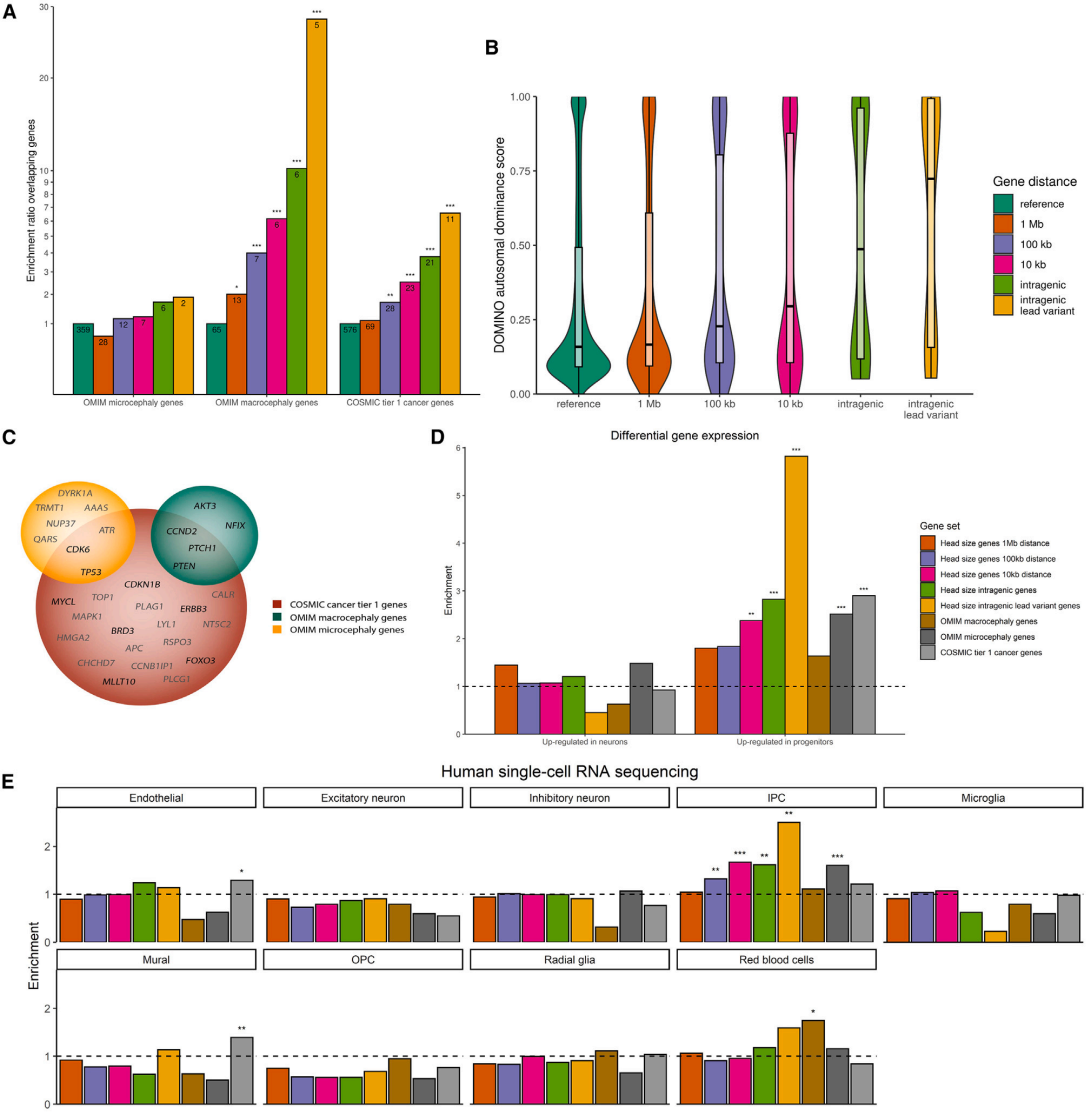
Gene enrichments stratified by distance from head size lead variants (A) Enrichment of OMIM macro- and microcephaly genes and COSMIC tier 1 genes near identified genetic loci. Depicted are enrichments of genes within 1 Mb (orange), 100 kb (purple), or 10 kb (pink) of identified genetic loci, genes with intragenic genetic variants (light green) and genes with intragenic genetic lead variants (yellow) in comparison with genes in the reference genome (dark green). ∗*p* < 0.05; ∗∗*p* < 0.0125 (0.05/4); ∗∗∗*p* < 0.0025 (0.05/4/5). (B) Violin plots showing DOMINO autosomal dominance scores of different gene sets. ∗*p* < 0.05; ∗∗*p* < 0.01; ∗∗∗*p* < 0.001. (C) Venn diagram showing genes within 10 kb of genetic loci that overlap with OMIM microcephaly genes (yellow) or macrocephaly genes (green) or COSMIC cancer tier 1 genes (red). Genes with intragenic lead variants are depicted in black, others in gray. (D) Bar plot showing enrichments of gene sets for genes differentially expressed in neurons and progenitors. ∗*p* < 0.05; ∗∗*p* < 0.025 (0.05/2); ∗∗∗*p* < 0.003 (0.05/2/8). (E) Bar plots showing enrichments of gene sets for the various cell types in the human cortical brain using single-cell RNA-sequencing data. ∗*p* < 0.05; ∗∗FDR < 0.05; ∗∗∗*p* < 0.0007 (0.05/9/8).

First, we investigated genes mutated in OMIM syndromes associated with abnormal head size, i.e., macrocephaly or microcephaly (Tables S19 and S20). We found increasing enrichment for macrocephaly genes with decreasing distance to the lead variants, culminating in a 37-fold enrichment of macrocephaly genes in genes containing an intragenic lead variant (Figure 4A). In contrast, microcephaly genes were not enriched with shorter distance from lead variants. The striking enrichment of macrocephaly genes did not change in the height-adjusted head size GWAS (Table S21). Furthermore, there was only a modest enrichment for macrocephaly genes in the height GWAS, even for the top 67 loci (i.e., the same number of loci as our GWAS; Table S21). Macrocephaly syndrome genes with intragenic lead variants include *AKT3*, *PTCH1*, *PTEN*, *CCND2*, and *NFIX* (Table S19). We conclude that common genetic variants near genes associated with macrocephaly syndromes, but not microcephaly syndromes, contribute to variation in head size in the general population. Our GWAS of head size may therefore identify novel macrocephaly genes. Accordingly, a patient with intellectual disability^25^ presented with macrocephaly and a mutation in *TICRR*, a gene for which a lead variant and variants in LD were eQTLs in 12 different tissues. *TICRR* acts in initiation of DNA replication and interacts with *CDK2*,^26^ a gene nearby another lead variant. *TICRR* is therefore an interesting candidate macrocephaly syndrome gene.

We determined whether cancer genes are enriched close to lead variants (Figure 4A). Indeed, there was a 9-fold enrichment for high-fidelity cancer genes (first-tier COSMIC^27^) among genes with an intragenic lead variant, which persisted after height adjustment (Table S21). There was only a modest enrichment of cancer genes close to height GWAS variants, providing additional evidence that cancer-related genes are specifically relevant for head size variation.

At a variant-level, no genetic correlation was found with GWAS meta-analyses of various cancer types^28^^,^^29^^,^^30^^,^^31^ (Table S22).

### Autosomal dominance score

We did not observe a significant enrichment for microcephaly genes (Figure 4A). This may be due to differences between the micro- and macrocephaly gene sets. Macrocephaly typically results from mutations with an autosomal dominant inheritance pattern (64.6%, Table S19), whereas microcephaly predominantly involves mutations with an autosomal recessive inheritance pattern (72.3%, Table S20). We observed a profound increase for genes with a predicted dominant inheritance pattern closer to our lead variants (Figure 4B). However, neither dominant nor recessive microcephaly genes were enriched (Table S21) and the predominant recessive inheritance patterns of microcephaly genes could not explain their lack of enrichment. An alternative explanation is that microcephaly syndromes are more clinically heterogeneous and the underlying mechanisms are less specific to brain and cranial growth.

### Gain of function and loss of function

The overlap among macrocephaly genes, microcephaly genes, and cancer genes is shown in Figure 4C. Macrocephaly-associated genes were more enriched for high-fidelity cancer genes than microcephaly-associated genes (enrichment ratio 12.9 vs. 3.2, Table S21). We therefore investigated whether the same mutation type, i.e., gain of function or loss of function, causes both macrocephaly syndromes as a germline mutation but also associate with cancer as somatic mutations. We found that this was the case for the vast majority of macrocephaly-associated genes with a defined role in cancer (37 of 41 genes, Table S19), i.e., the same type of mutation associated with both macrocephaly and cancer. Moreover, germline mutations in 14 of these 37 genes, including our GWAS genes *PTEN*, *PTCH1*, and *SUFU*, are associated with a syndrome or condition with a suggested cancer predisposition (Table S19). Our GWAS data and these observations may therefore suggest that subtle up-regulation of oncogenes and oncogenic pathways or down-regulation of tumor suppressor genes and pathways increases head size in the general population.

### Brain cell expression

As neural progenitors are the actively dividing cells in the developing brain, their expressed genes may explain the observed genetic variants for head size.^32^ Indeed, genes at or near the head size loci were enriched in differentially expressed neural progenitor cell genes (Figure 4D; Table S23). Subsequently, we looked at a single-cell RNA-sequencing (scRNA-seq) dataset from cell types in the human cortex.^33^^,^^34^ Intriguingly, we find that genes close to head size variants are strongly enriched for genes preferentially expressed in intermediate progenitor cells (IPCs) (Figure 4E; Tables S24 and S25; Figure S2). Increased proliferation of IPCs in a primate-specific area of the brain, the outer region subventricular zone, is believed to be responsible for the evolutionary expansion of the human brain.^35^^,^^36^ This suggests that genetic variation regulating the proliferation or neuronal differentiation of IPCs plays an important role in determining human head size. Indeed, Wnt pathway genes, p53 pathway genes, and *PTCH1*, *SUFU*, and *NFIX*, which we find near genetic variants determining head size, are examples of regulators of IPCs.^37^^,^^38^^,^^39^^,^^40^^,^^41^^,^^42^^,^^43^ To understand which type of variants influence head size, we performed a partitioned heritability analysis that classifies variants into categories based on functional elements. We found an enrichment for variants in the regulatory elements of both neural progenitors and their neuronal progenies (enrichment_progenitors_ = 12.7, *p* = 8.3 × 10^−4^; enrichment_neurons_ = 16.1, *p* = 3.7 × 10^−4^).

Finally, we assessed whether a similar pattern was seen for the Catalog of Somatic Mutations in Cancer (COSMIC) first-tier cancer genes. Indeed, our differential gene expression analysis dataset indeed showed an enrichment of cancer genes in the genes specific for neural progenitors (enrichment = 2.9, *p* = 1.7 × 10^−6^, Table S23). However, no significant enrichment was found for IPCs using the scRNA-seq data.

## Discussion

Here we performed the largest head size GWAS to date and found that associated genetic variants significantly locate to cancer genes and cancer-associated pathways. Genes near head size variants were enriched for high-fidelity cancer genes even after adjustment for height, suggesting a specific association of head growth with cancer, rather than general growth. Germline mutations in multiple macrocephaly syndrome genes are known to be an increased cancer risk, including *PTEN* (Cowden syndrome) and *PTCH1* (Gorlin syndrome) (Table S19). Our GWAS was performed in the general population, which prompts the question of whether the link between head size and cancer extends beyond rare genetic syndromes.

Previous meta-analyses of prospective observational studies found associations between adult height and increased risk for various forms of cancer.^44^ Similarly, head circumference at birth has previously been positively associated with brain cancer during childhood,^45^ and with different types of cancer later in life including stomach cancer and breast cancer,^46^ with stronger associations than for respectively birth weight or birth length. The correlation between head size at birth and breast cancer later in life was further supported by a pooled analysis of 32 studies,^47^ but not by another prospective cohort study.^48^ Our study provides further evidence for this link between head size and cancer.

The abovementioned observational studies together with our genetic results suggest that early growth rather than later adolescent growth may be associated with neoplasia, since cranial growth is completed around the sixth year of age, whereas height is primarily determined by peri-pubertal growth. Head size at birth and its growth during early infancy in relation to cancer risk therefore deserves further studies to identify potential underlying pathophysiological mechanisms and its potential clinical implications.^45^^,^^49^^,^^50^

### Limitations of the study

Although this study suggests an association between head growth and cancer, further studies are needed to investigate whether head size is causally related to cancer development. In our study, we were not able to account for environmental factors such as socio-economic status and diet, especially during childhood, which would be important to adjust for in future studies. In addition, the clinical implications of the findings of our study need to be investigated, for example if patients with clinical macrocephaly syndromes need to be screened for cancer more extensively.

## Consortia

The members of the Cohorts for Heart and Aging Research in Genomic Epidemiology (CHARGE) Consortium are Philippe Amouyel, Konstantinos Arfanakis, Benjamin S. Aribisala, Mark E. Bastin, Ganesh Chauhan, Christopher Chen, Ching-Yu Cheng, Philip L. de Jager, Ian J. Deary, Debra A. Fleischman, Rebecca F. Gottesman, Vilmundur Gudnason, Saima Hilal, Edith Hofer, Deborah Janowitz, J. Wouter Jukema, David C.M. Liewald, Lorna M. Lopez, Oscar Lopez, Michelle Luciano, Oliver Martinez, Wiro J. Niessen, Paul Nyquist, Jerome I. Rotter, Tatjana Rundek, Ralph L. Sacco, Helena Schmidt, Henning Tiemeier, Stella Trompet, Jeroen van der Grond, Henry Völzke, Joanna M. Wardlaw, Lisa Yanek, and Jingyun Yang.

The members of the Enhancing NeuroImaging Genetics through Meta-Analysis (ENIGMA) Consortium are Ingrid Agartz, Saud Alhusaini, Laura Almasy, David Ames, Katrin Amunts, Ole A. Andreassen, Nicola Armstrong, Manon Bernard, John Blangero, Laura M.E. Blanken, Marco P. Boks, Dorret I. Boomsma, Adam M. Brickman, Henry Brodaty, Randy L. Buckner, Jan K. Buitelaar, Dara M. Cannon, Vaughan J. Carr, Stanley V. Catts, M. Mallar Chakravarty, Qiang Chen, Christopher R.K. Ching, Aiden Corvin, Benedicto Crespo-Facorro, Joanne E. Curran, Gareth E. Davies, Eco J.C. de Geus, Greig I. de Zubicaray, Anouk den Braber, Sylvane Desrivières, Allissa Dillman, Srdjan Djurovic, Wayne C. Drevets, Ravi Duggirala, Stefan Ehrlich, Susanne Erk, Thomas Espeseth, Iryna O. Fedko, Guillén Fernández, Simon E. Fisher, Tatiana M. Foroud, Tian Ge, Sudheer Giddaluru, David C. Glahn, Aaron L. Goldman, Robert C. Green, Corina U. Greven, Oliver Grimm, Narelle K. Hansell, Catharina A. Hartman, Ryota Hashimoto, Andreas Heinz, Frans Henskens, Derrek P. Hibar, Beng-Choon Ho, Pieter J. Hoekstra, Avram J. Holmes, Martine Hoogman, Jouke-Jan Hottenga, Hilleke E. Hulshoff Pol, Assen Jablensky, Mark Jenkinson, Tianye Jia, Karl-Heinz Jöckel, Erik G. Jönsson, Sungeun Kim, Marieke Klein, Peter Kochunov, John B. Kwok, Stephen M. Lawrie, Stephanie Le Hellard, Hervé Lemaître, Carmel Loughland, Andre F. Marquand, Nicholas G. Martin, Jean-Luc Martinot, Mar Matarin, Daniel H. Mathalon, Karen A. Mather, Venkata S. Mattay, Colm McDonald, Francis J. McMahon, Katie L. McMahon, Rebekah E. McWhirter, Patrizia Mecocci, Ingrid Melle, Andreas Meyer-Lindenberg, Patricia T. Michie, Yuri Milaneschi, Derek W. Morris, Bryan Mowry, Kwangsik Nho, Thomas E. Nichols, Markus N. Nöthen, Rene L. Olvera, Jaap Oosterlaan, Roel A. Ophoff, Massimo Pandolfo, Christos Pantelis, Irene Pappa, Brenda Penninx, G. Bruce Pike, Paul E. Rasser, Miguel E. Rentería, Simone Reppermund, Marcella Rietschel, Shannon L. Risacher, Nina Romanczuk-Seiferth, Emma Jane Rose, Perminder S. Sachdev, Philipp G. Sämann, Andrew J. Saykin, Ulrich Schall, Peter R. Schofield, Sara Schramm, Gunter Schumann, Rodney Scott, Li Shen, Sanjay M. Sisodiya, Hilkka Soininen, Emma Sprooten, Velandai Srikanth, Vidar M. Steen, Lachlan T. Strike, Anbupalam Thalamuthu, Arthur W. Toga, Paul Tooney, Diana Tordesillas-Gutiérrez, Jessica A. Turner, Maria del C. Valdés Hernández, Dennis van der Meer, Nic J.A. Van der Wee, Neeltje E.M. Van Haren, Dennis van 't Ent, Dick J. Veltman, Henrik Walter, Daniel R. Weinberger, Michael W. Weiner, Wei Wen, Lars T. Westlye, Eric Westman, Anderson M. Winkler, Girma Woldehawariat, Margaret J. Wright, and Jingqin Wu.

## STAR★Methods

### Key resources table

**Table:** 

REAGENT or RESOURCE	SOURCE	IDENTIFIER
**Deposited data**		

Genome-wide association study summary statistics	CHARGE dbGaP and http://enigma.ini.usc.edu/research/download-enigma-gwas-results	phs000930 (dbGaP accession number)

**Software and algorithms**		

EasyQC	Winkler et al.^51^	Software - Universität Regensburg (uni-regensburg.de)
METAL	Willer et al.^52^	METAL Documentation - Genome Analysis Wiki (umich.edu)
LD score regression	Bulik-Sullivan et al.^53^	GitHub - bulik/ldsc: LD Score Regression (LDSC)
LocusZoom	Pruim et al.^54^	LocusZoom - Create Plots of Genetic Data
FUMA GWAS	Watanabe et al.^55^	Functional Mapping and Annotation of Genome-wide association studies (ctglab.nl)
TWAS-Fusion	Gusev et al.^56^	TWAS/FUSION (gusevlab.org)
DOMINO	Quinodoz et al.^57^	Domino (iob.ch)

**Other**		

OMIM database	Amberger et al.^58^	Home - OMIM
Cortical organoids’ scRNA-seq data	Bhaduri et al.^34^	https://organoidreportcard.cells.ucsc.edu
e/sQTLs, and allele-specific expression in cultured primary human neural progenitors and their sorted neuronal progeny	Aygün et al.^32^	https://bitbucket.org/steinlabunc/expression_splicing_qtls_public/src/master/

### Resource availability

#### Lead contact

Further information and requests for resources and reagents should be directed to and will be fulfilled by the lead contact, Hieab H.H. Adams (Hieab.Adams@radboudumc.nl).

#### Materials availability

This study did not generate new unique reagents.

#### Data and code availability

The genome-wide summary statistics that support the findings of this study will be made available through the CHARGE dbGaP (accession number phs000930) and ENIGMA (http://enigma.ini.usc.edu/research/download-enigma-gwas-results) websites.

No previously unreported custom computer code or mathematical algorithm was used to generate results central to the conclusions.

Any additional information required to re-analyse the data reported in this work paper is available from the lead contact upon request.

### Experimental model and subject details

#### Study population

Most studies participate in the Cohorts for Heart and Aging Research in Genomic Epidemiology (CHARGE)^59^ or the Enhancing NeuroImaging Genetics through Meta-Analysis (ENIGMA)^60^ consortium. We also included the results of the most recent head circumference GWAS.^5^ A complete overview of the included studies is shown in Table S1 and their population characteristics are presented in Table S2. Each contributing study was approved by their institutional review boards or local ethical committees. Written informed consent was obtained from all study participants.

#### Genotyping

Genotyping of individuals was performed on commercially available arrays, and imputed to 1000 Genomes (1KG) or Haplotype Reference Consortium (HRC) imputation panels (Table S3). Quality control was performed using the EasyQC software.^51^ In each study, genetic variants with an imputation quality r^2^ below 0.3 and a minor allele frequency (MAF) below 0.001 were excluded. Additionally, variants were filtered on study level requiring $\left(r^{2}\;x\;MAF\;x\;N\right)>5$.

#### Phenotyping

Different methods were used to measure human head size across studies. Briefly, either head circumference was measured, or intracranial volume was measured on computed tomography (CT) or magnetic resonance imaging (MRI) scans. In total, human head size was measured using intracranial volume measured on CT or MRI scans in respectively 1,283 and 84,171 individuals, and using head circumference in 20,524 individuals (Table S4). These measures have previously shown to be phenotypically and genetically correlated,.^5^^,^^6^^,^^61^ Genetic correlations between our MRI scans and head circumference measurements was 0.75. Together, this allowed us to perform a combined meta-analyse of different measures of head size.

### Method details

#### Genome-wide association studies

GWAS were performed for each study adjusted for age, age^2^ (if significant), gender, eigenstrat PC1-4 (if significant), study-specific adjustments and case-control status (if applicable). In a second model, additional adjustment for height were made. The METAL software^52^ was used to perform a sample size weighted *Z* score meta-analysis. After meta-analysis, genetic variants available in less than 5,000 individuals were excluded. Comparable betas were derived using the formula $Zscore\;x\;\sqrt{\frac{1}{N\;x\;2\;x\;MAF}}$ as was done previously.^62^ Genomic inflation and polygenic heterogeneity were assessed using the LD score regression software^53^ by comparing the genomic control inflation factor and the LD score regression intercept (Table S5).

GWAS meta-analyses were performed separately for African, Asian and European samples. We also performed a transancestral meta-analysis. Since the analyses in non-European samples were underpowered, we additionally used an inverse-variance weighted method to test the combined effects of the lead variants in the non-European samples. This analysis was performed using the gtx package as implemented in R.

#### Functional annotations

Regional association plots were made with the LocusZoom software.^54^ The Functional Mapping and Annotation of Genome-Wide Association Studies (FUMA GWAS) platform^55^ was used to derive the independent genomic loci and genetic lead variants, and to functionally annotate the identified genetic variants. Additionally, enrichment for KEGG^9^ biological pathways was assessed for genes located nearby the identified genetic loci using the default options in FUMA, using hypergeometric tests. Genotype-Tissue Expression (GTEx) v7 was used to identify expression quantitative trait loci (eQTL) for the lead genetic variants and variants in LD (r^2^ > 0.6).

We performed a transcriptome-wide association study (TWAS) using the association statistics from the European-only head size GWAS summary statistics and weights from 21 publicly available gene expression reference panels. We focused on the gene expression weights from blood (Young Finns Study, YFS), arterial (GTEx), brain (GTEx, CommonMind Consortium (CMC)) and peripheral nerve tissues (GTEx). Precomputed SNP-expression weights in the 1-Mb window were obtained for each gene in the reference panel, including the highly-tissue specific splicing QTL (sQTL) information on gene isoforms in the dorsolateral prefrontal cortex (DLPFC, CMC). Using the SNP-expression weights, SNP-trait effect estimates and the SNP correlation matrix, we used the TWAS-Fusion^56^ to estimate the association statistic between the predicted expression and head size (TWAS *Z* score). Transcriptome-wide significant genes (eGenes) and the corresponding QTLs (eQTLs) were determined using Bonferroni correction in each reference panel, based on the average number of features (4,320 genes) tested across all the reference panels.^56^ Finally, using a prior association probability of 1.1 × 10^−5^ and colocalization analysis (COLOC)^63^ for each locus we estimated the posterior probability of a shared causal variant (PP4>0.75) between the gene expression and trait association. eGene regions with eQTLs not reaching genome-wide significance in the head size GWAS were considered putatively novel TWAS signals. Furthermore, functional validation of the eGenes was performed by integrating eQTL with the functional genomics feature from the RegulomeDB.^64^ A RegulomeDB probability score greater than 0.5 and closer to 1 indicates the likelihood of the eQTL having a gene-regulatory role. Finally, accounting for pairwise correlation between the gene expression features we conducted the multiple degree of freedom omnibus analysis, to test for the shared effect of eGenes across the different gene reference panels. A significance threshold of *p* < 3.48 × 10^−6^ accounting for the number of genes (*N* = 14,385) tested was used to identify significant eGenes in the omnibus test.

#### Effects on anthropomorphic measures and regional brain volumes

The LD score regression software^53^^,^^65^ was used to assess genetic correlations with adult height,^66^ for both the height-unadjusted and height-adjusted model.

Dual-energy X-ray absorptiometry (DXA) measurements of the UK Biobank imaging subsample (*N* = 3,313) were used to examine the effect of the identified lead variants on anthropometric measures across the body, i.e., bone area of the arms, legs, pelvis, ribs, spine, trunk and vertebrae L1-L4. In these analyses values more than three standard deviations from the mean were considered outliers and removed from the analyses. We adjusted for age, age,^2^ gender and principal components (model 1), and additionally for height (model 2) to correct for an overall growth effect.

To investigate the effects of the identified variants for head size on growth in specific brain regions, we investigated the overlap between the identified loci for head size and previous genome-wide association studies (GWASs) on brain volumes.^7^^,^^67^^,^^68^^,^^69^^,^^70^ We also analyzed the associations between the identified lead genetic variants and global volumes (i.e., four brain lobes and lateral ventricle volumes), subcortical volumes (i.e., volumes of eight subcortical structures) and cortical volumes (i.e., volumes of 34 cortical regions of interest) in the UK Biobank (*N* = 22,145). Volumes were derived using the FreeSurfer 6.0 software. Values more than 3.5 standard deviations away from the mean were considered outliers and removed from the analysis. In the first model, we adjusted for age, age,^2^ gender and principal components, and in the second model additionally for intracranial volume.

Additionally, we took the lead variants specifically associated with one or two subcortical volumes, and investigated their effects on the shape of seven subcortical structures, i.e., amygdala, caudate nucleus, hippocampus, nucleus accumbens, pallidum, putamen and thalamus. The radial distances and log Jacobian determinants were derived using the ENIGMA-Shape package (http://enigma.usc.edu/ongoing/enigma-shape-analysis/). Volumetric outliers more than 3.5 standard deviations from the mean were removed from the analysis.

We performed 10,000 permutations to define the number of independent DXA, brain volumetric and subcortical shape outcomes. We used this number to define our multiple testing adjusted *p* value thresholds for significance, i.e., 0.05/(number of independent outcomes x number of lead genetic variants).

#### Genetic correlations

We investigated the genetic correlations with neuropsychiatric traits using the LD score regression software.^53^^,^^65^ Genetic correlation analyses were performed for educational attainment,^71^ general cognitive function,^72^ all stroke,^73^ Alzheimer’s disease,^74^ frontotemporal dementia,^75^ Parkinson’s disease,^76^ anorexia nervosa,^77^ attention-deficit hyperactivity disorder,^78^ autism spectrum disorder,^79^ bipolar disorder,^80^ extraversion,^81^ insomnia,^82^ major depressive disorder,^83^ neuroticism,^84^ obsessive compulsive disorder^85^ and schizophrenia.^80^ Analyses were performed in the entire GWAS dataset as well as in the GWAS set with newly included studies in comparison to the intracranial volume GWAS performed by Adams et al.^6^

We also performed genetic correlation analyses for publicly available cancer GWAS, namely for breast cancer,^28^ ovarian cancer^29^ and prostate cancer.^30^ To obtain information on more cancer types, we additionally included GWAS of cancer registries from the UK Biobank and Kaiser Permanente Genetic Epidemiology Research on Adult Health and Aging (GERA).^31^ Of those, we excluded cancer types with less than 1,000 cases, which left the following cancer types to be analyzed: bladder cancer (N_cases_ = 2,242), breast cancer (N_cases_ = 17,881), cervical cancer (N_cases_ = 6,563), colon cancer (N_cases_ = 3,793), endometrial cancer (N_cases_ = 2,037), esophageal/gastric cancer (N_cases_ = 1,091), kidney cancer (N_cases_ = 1,338), lung cancer (N_cases_ = 2,485), malignant melanoma (N_cases_ = 6,777), non-Hodgkin’s lymphoma (N_cases_ = 2,400), prostate cancer (N_cases_ = 10,792) and rectal cancer (N_cases_ = 2,091). Genetic correlations with oral cavity/pharyngeal cancer (N_cases_ = 1,223) and ovarian cancer (N_cases_ = 1,259) could not be calculated due to low heritability estimates.

#### Enrichment analyses

We performed enrichment analyses of different gene sets: genes within 1 Mb, 100 kb or 10 kb of the identified genetic loci, genes within 10 kb of the identified genetic loci with intragenic genetic variants, and genes within 10 kb of the identified genetic loci with intragenic genetic lead variants. As a reference, we used the rest of the protein-coding genome.

First, the Online Mendelian Inheritance in Man (OMIM) database^58^ was used to retrieve information on genes related to heritable phenotypes affecting head size (Tables S19 and S20). Second, the COSMIC database^27^ was used to extract Tier 1 cancer genes. Taking the rest of the genome as our reference gene set, we calculated the enrichment of these macrocephaly, microcephaly and cancer genes in the abovementioned gene sets.

Lastly, DOMINO,^57^ a previously developed machine learning tool, was used to assess if the genes in the different gene sets were more often predicted to harbor dominant changes in comparison with genes in the rest of the genome.

Mean autosomal dominance scores were compared with the reference genome using a Mann-Whitney test. Differences in the proportions for the OMIM macro- and microcephaly genes, intellectual disability genes and COSMIC genes were calculated using a Pearson’s χ^2^ test.

We performed these analyses for the head size height-unadjusted GWAS results, but also the GWAS in the subset of studies for which height was available, the height-adjusted GWAS and the height GWAS.^66^ For comparison, we also selected the top 67 loci for the height GWAS, so the results were not driven by a difference in the number of associated loci.

#### Experimental datasets of brain cells

To assess whether the identified genes in the current study are enriched for genes differentially expressed in human progenitors versus neurons, we utilized differential gene expression data of those cell lines, derived from a previously published sample population (N_donor_ = 85 in progenitors and N_donor_ = 74 in neurons).^32^ Using genes with at least 10 counts in more than 5% of the cell-type specific donors in either cell-type (resulting in 16,172 protein-coding genes out of 28,785 genes in total), we performed a paired differential gene expression analysis with design matrix: model.matrix(∼ CellType + as.factor(DonorID) + RIN, data) as described previously,^32^ using the limma R package.^86^ We detected 1,095/1,420 protein genes upregulated in progenitors/neurons, respectively, for abs(logFC) > 1.5 and adjusted *p* value < 0.05. Performing a hypergeometric test, we evaluated if multiple protein-coding gene sets: head size gene sets with different distances from the lead variants, OMIM macrocephaly and microcephaly genes, and COSMIC tier 1 cancer genes are enriched among the protein-coding genes upregulated in progenitors or neurons.

Using a different approach, scRNA-seq data were used to investigate whether our genes of interest were enriched for genes specific for certain cortical brain cell types. Specifically, scRNA-seq data from the developing human cortex (gestational week 6–22, more than 189,000 cells) were used to identify the top 10% of genes specific for a certain cell type.^34^ Using this data, we first performed LD score regression^53^ based enrichment analyses of the head size GWAS summary statistics, as previously described.^33^^,^^87^ Gene specificity was defined as the ratio of expression of a gene in a cell type by the total expression of that gene in all cell types. In parallel, we again tested the enrichment of various gene sets: head size gene sets with different distances from the lead variants, OMIM macrocephaly and microcephaly genes, and COSMIC tier 1 cancer genes, with the top 10% of cell specific genes for each cell type using hypergeometric tests. FDR correction was used to correct for the multiple gene sets tested for enrichment in each cell type.

To determine if regulatory elements of neural progenitors are enriched for the heritability of head size, we performed partitioned heritability analyses^53^^,^^88^ using chromatin accessibility profiles from a population of 76 primary human neural progenitor cells and 61 of their differentiated neuronal progenies, as was done previously.^89^

### Quantification and statistical analysis

Please see the statistical analyses and software in method details.

## Supplemental information

Document S1. Figures S1 and S2 and Tables S1–S3, S5, S10, S14, S15, S22, S24, and S25

Table S4. Phenotyping information, related to STAR Methods section "Phenotyping"

Table S6. Lead genetic variants and their effects on human head size in samples of different ethnicities, with and without adjustment for height. Related to Figure 1B

Table S7. Genome-wide significant genetic variants and variants in linkage disequilibrium (r2>0.6), including functional annotations. Related to Figure 1A and 1B

Table S8. Lead variants of the combined discovery and validation GWAS meta-analysis, related to Figure 1A and 1B

Table S9. Effects of lead genetic variants on bone size area measured using dual-energy X-ray absorptiometry (DXA), related to Figure 1C and the STAR Methods section "Effects on anthropomorphic measures and regional brain volumes"

Table S11. The effects of previously identified genetic variants for regional brain volumes in the current genome-wide association study, related to Figure 2A

Table S12. Association of identified lead genetic variants with regional brain volumes, related to Figure 2B

Table S13. Results of the subcortical shape analyses of seven lead genetic variants specifically associated with one or two subcortical structures, related to Figure 2C

Table S16. Conditional analysis with COLOC using significant TWAS associations. Related to STAR Methods section "Functional Annotations"

Table S17. TWAS omnibus test results, related to STAR Methods section "Functional Annotations"

Table S18. Genes in or nearby identified genetic loci (<1 Mb), related to Figure 1A, 3B and 3C, and to STAR Methods section "Enrichment analyses"

Table S19. List of genes linked to macrocephaly in the Online Mendelian Inheritance of Man (OMIM) database, related to STAR Methods section "Enrichment analyses" and Figure 4C

Table S20. List of genes linked to microcephaly in the Online Mendelian Inheritance of Man (OMIM) database, related to STAR Methods section "Enrichment analyses" and Figure 4C

Table S21. Enrichment of micro- and macrocephaly OMIM genes, COSMIC tier 1 cancer genes, intellectual disability trios and autosomal dominance DOMINO score, related to Figure 4A

Table S23. Differential gene expression analysis of neural progenitors versus neurons, related to Figure 4D

Data S1. Forest plots presenting the study-specific associations of the identified lead genetic variants with human head size, part 1, related to Figure 1B

Data S2. Forest plots presenting the study-specific associations of the identified lead genetic variants with human head size, part 2, related to Figure 1B

Data S3. Regional plots of the identified genetic loci for human head size (±100 kb), related to Figure 1A and 1B

Document S2. Article plus supplemental information
