# Supplementary material for: Genetic variants for head size share genes and pathways with cancer
Source: Cell Rep Med. 2024 May 3;5(5):101529. doi: 10.1016/j.xcrm.2024.101529 (PMC11148644; doi:10.1016/j.xcrm.2024.101529)
Supplement: Document S1. Figures S1 and S2 and Tables S1–S3, S5, S10, S14, S15, S22, S24, and S25 [file mmc1.pdf]

Cell Reports Medicine, Volume *5*

## Supplemental information

# Genetic variants for head size share genes and pathways with cancer

**Maria J. Knol, Raymond A. Poot, Tavia E. Evans, Claudia L. Satizabal, Aniket Mishra, Muralidharan Sargurupremraj, Sandra van der Auwera, Marie-Gabrielle Duperron, Xueqiu Jian, Isabel C. Hostettler, Dianne H.K. van Dam-Nolen, Sander Lamballais, Mikolaj A. Pawlak, Cora E. Lewis, Amaia Carrion-Castillo, Theo G.M. van Erp, Céline S. Reinbold, Jean Shin, Markus Scholz, Asta K. Håberg, Anders Kämpe, Gloria H.Y. Li, Reut Avinun, Joshua R. Atkins, Fang-Chi Hsu, Alyssa R. Amod, Max Lam, Ami Tsuchida, Mariël W.A. Teunissen, Nil Aygün, Yash Patel, Dan Liang, Alexa S. Beiser, Frauke Beyer, Joshua C. Bis, Daniel Bos, R. Nick Bryan, Robin Bülow, Svenja Caspers, Gwenaëlle Catheline, Charlotte A.M. Cecil, Shareefa Dalvie, Jean-François Dartigues, Charles DeCarli, Maria Enlund-Cerullo, Judith M. Ford, Barbara Franke, Barry I. Freedman, Nele Friedrich, Melissa J. Green, Simon Haworth, Catherine Helmer, Per Hoffmann, Georg Homuth, M. Kamran Ikram, Clifford R. JackJr., Neda Jahanshad, Christiane Jockwitz, Yoichiro Kamatani, Annchen R. Knodt, Shuo Li, Keane Lim, W.T. Longstreth, Fabio Macciardi, The Cohorts for Heart and Aging Research in Genomic Epidemiology (CHARGE) Consortium, The Enhancing NeuroImaging Genetics through Meta-Analysis (ENIGMA) Consortium, Outi Mäkitie, Bernard Mazoyer, Sarah E. Medland, Susumu Miyamoto, Susanne Moebus, Thomas H. Mosley, Ryan Muetzel, Thomas W. Mühleisen, Manabu Nagata, Soichiro Nakahara, Nicholette D. Palmer, Zdenka Pausova, Adrian Preda, Yann Quidé, William R. Reay, Gennady V. Roshchupkin, Reinhold Schmidt, Pamela J. Schreiner, Kazuya Setoh, Chin Yang Shapland, Stephen Sidney, Beate St Pourcain, Jason L. Stein, Yasuharu Tabara, Alexander Teumer, Anne Uhlmann, Aad van der Lugt, Meike W. Vernooij, David J. Werring, B. Gwen Windham, A. Veronica Witte, Katharina Wittfeld, Qiong Yang, Kazumichi Yoshida, Han G. Brunner, Quentin Le Grand, Kang Sim, Dan J. Stein, Donald W. Bowden, Murray J. Cairns, Ahmad R. Hariri, Ching-Lung Cheung, Sture Andersson, Arno Villringer, Tomas Paus, Sven Cichon, Vince D. Calhoun, Fabrice Crivello, Lenore J. Launer, Tonya White, Peter J. Koudstaal, Henry Houlden, Myriam Fornage, Fumihiko Matsuda, Hans J. Grabe, M. Arfan Ikram, Stéphanie Debette, Paul M. Thompson, Sudha Seshadri, and Hieab H.H. Adams**

# Table of Contents


**Supplementary Tables** .......... 2

**Table S1.** List of all contributing studies. *Related to STAR Methods' section "Study Population".* ... 2

**Table S2.** Population characteristics of new or updated contributing studies. *Related to STAR Methods' section "Study Population".* .......... 5

**Table S3.** Information on genotyping and quality control. *Related to STAR Methods' section "Genotyping".* .......... 7

**Table S5.** Lambda genomic control, LD score regression intercept and ratio, and SNP-based heritability for different models. *Related to STAR Methods' section "Genome-wide association studies".* .......... 10

**Table S10.** Overlap between identified loci and previously identified loci in genome-wide association studies of brain volumes. *Related to Figure 2A.* .......... 11

**Table S14.** Genetic correlation between the human head size and neuropsychiatric traits. *Related to Figure S3, Figure 1C and STAR Methods' section "Genetic correlations".* .......... 14

**Table S15.** Kyoto Encyclopaedia of Genes and Genomes (KEGG) pathway analysis. *Related to Figure 3A.* .......... 15

**Table S22.** Genetic correlation between the human head size and different cancer types. *Related to STAR Methods' section "Genetic correlations".* .......... 18

**Table S24.** Enrichment of head size GWAS for brain cell types based on human scRNA-seq data. *Related to Figure 4E.* .......... 19

**Table S25.** Enrichment of gene sets for brain cell types based on human scRNA-seq data. *Related to Figure 4E.* .......... 21

**Supplementary Figures** .......... 24

**Figure S1.** Genetic correlations with neuropsychiatric traits. *Related to Figure 1C.* .......... 24

**Figure S2.** Enrichment of expression of human head size GWAS genes in cell subtypes in the human cortical brain, derived from single-cell RNA sequencing data. *Related to Figure 4.* .......... 25

# Supplementary Tables

**Table S1.** List of all contributing studies. *Related to STAR Methods' section "Study Population".*

| **Study** |
|---|
| 1000Brains |
| 3C-Bordeaux |
| 3C-Dijon |
| AddNeuroMed |
| ADNI |
| ADNI2GO |
| AGES |
| ALSPAC |
| ARIC-Black |
| ARIC-White |
| ASPS |
| ASPSFam |
| ASRB |
| Betula |
| BFS |
| BIG |
| BIL&GIN |
| BrainSCALE |
| BRCDECC |
| CARDIA-Black |
| CARDIA-White |
| CHAP |
| CHAP-Black |
| CHS |
| COPSAC2000 |
| COPSAC2010 |
| CROATIA-KORCULA |
| CROMIS-2 ICH |
| DHS |
| DNS |
| EDIS-SCES |
| EDIS-SiMES |
| EPIGEN |
| EPOZ |
| ERF |
| ESS |
| FBIRN |
| FHS |
| GeneSTAR |

| |
|---|
| GenR |
| GIG |
| GSP |
| HKOS |
| HUBIN |
| HUNT |
| IMAGEN |
| IMH |
| INMA |
| i-Share |
| LBC1936 |
| LIFE-Adult |
| LLS |
| MCIC |
| Meth-CT |
| MooDS |
| MPIP |
| Nagahama |
| NCNG |
| NESDA |
| neuroIMAGE |
| NOMAS |
| NOMAS-Black |
| NOMAS-Hispanic |
| NTR - Adults |
| OATS |
| ORCADES |
| PAFIP |
| Poznan MS |
| PROSPER |
| QTIM |
| RAINE |
| ROSMAP1 |
| ROSMAP2 |
| RSI |
| RSII |
| RSIII |
| SHIP |
| SHIP-TREND |
| Sydney MAS |
| SYS adolescents |
| SYS adults |
| TOP |
| UK Biobank |
| UMCU |

| |
|---|
| VIDI |
| VIKING |
| WHICAP |
| WHICAP-Black |

**Table S2.** Population characteristics of new or updated contributing studies. *Related to STAR Methods' section "Study Population".*

| Cohort | Study Design | Ancestry | Total N | N Females | Mean Age (SD) | Age Range |
|---|---|---|---|---|---|---|
| **European samples** | | | | | | |
| **1000 Brains** | Population-based | European | 751 | 343 | 66.8 (6.8) | 53 - 85 |
| **3C-Bordeaux** | Population-based | European | 564 | 323 | 72.7 (4.0) | 65 - 82 |
| **ARIC whites** | Population-based | European | 1390 | 812 | 76.3 (5.3) | 65 - 90 |
| **ASRB** | Case-control (SCZ and healthy controls) | European | 233 | 95 | 38.5 (11.3) | 19 - 64 |
| **CARDIA whites** | Population-based | European | 349 | 185 | 51.0 (3.2 ) | 42 - 56 |
| **CROMIS-2 ICH** | Population-based | European | 642 | 270 | 75.7 (10.8) | 41-100 |
| **DHS** | Family-based study | European | 460 | 258 | 60.6 (9.0) | 33 - 82 |
| **DNS** | Population-based | European | 516 | 275 | 19.8 (1.24) | 18 - 22 |
| **EPOZ** | Population-based | European | 251 | 144 | 69.4 (6.0) | 60 - 86 |
| **ESS** | Case-only (TIA and stroke cases) | European | 641 | 293 | 62.6 (13.8) | 17 - 93 |
| **FHS (update - previous + additional samples)** | Population-based | European | 4710 | 2519 | 56.9 (13.0) | 26 - 97 |
| **GenR (additional samples)** | Population-based | European | 265 | 139 | 10.3 (0.7) | 9 - 12 |
| **HUNT** | Population-based | European | 903 | 478 | 58.9 (4.2) | 50 - 66 |
| **LIFE-Adult** | Population-based | European | 1825 | 890 | 63.2 (11.3) | 20 - 82 |
| **Poznan MS** | Case-only (Multiple sclerosis) | European | 204 | 142 | 34.0 (7.9) | 18 - 54 |
| **RSI (update - previous + additional samples)** | Population-based | European | 1103 | 635 | 79.1 (6.0) | 70 - 100 |
| **RSII (update - previous + additional samples)** | Population-based | European | 1159 | 603 | 69.6 (7.1) | 60 - 100 |
| **RSIII (update - previous + additional samples)** | Population-based | European | 2582 | 1427 | 57.3 (6.9) | 50 - 90 |
| **SHIP-TREND (additional samples)** | Population-based | European | 1084 | 530 | 51.7 (14.2) | 21-82 |

| | | | | | | |
|---|---|---|---|---|---|---|
| **SYS adolescents** | Family-based study | European | 980 | 506 | 15.0 (1.8) | 12 - 19 |
| **SYS adults** | Family-based study | European | 594 | 320 | 49.3 (5.0) | 36 - 63 |
| **UK Biobank** | Population-based | European | 14571 | 7592 | 55.1 (7.4) | 40 - 70 |
| **VIDI** | RCT/Population-based | European | 755 | 374 | 1.99 (0.03) | 1 - 2 |
| **Non-European/mixed ancestry samples** | | | | | | |
| **ARIC-Black** | Population-based | African-American | 675 | 434 | 74.3 (5.1) | 61 - 89 |
| **CARDIA-Black** | Population-based | African-American | 132 | 75 | 49.0 (3.6) | 43 - 56 |
| **FBIRN** | Case-control (SCZ and healthy controls) | Mixed | 275 | 65 | 38.9 (11.3) | 18 - 62 |
| **HKOS** | Population-based | Southern Chinese | 888 | 709 | 58.5 (11.5) | 32 - 96 |
| **IMH** | Case-control (SCZ and healthy controls) | Singaporean | 37 | 22 | 28.7 (6.5) | 19 - 45 |
| **Meth-CT** | Case-control (addiction, SIPD, healthy controls) | South African | 114 | 25 | 26.6 (6.4) | 18 - 53 |
| **Nagahama** | Population-based | Japanese | 2889 | 1861 | 68.3 (5.3) | 59 - 81 |
| **Validation samples** | | | | | | |
| **BIL&GIN** | Population-based | European | 265 | 136 | 25.5 (6.1) | 18 - 53 |
| **i-Share** | Population-based | European | 1777 | 1278 | 22.1 (2.3) | 18 - 35 |
| **UK Biobank (independent sample)** | Population-based | European | 23890 | 12600 | 65.1 (7.4) | 47 - 81 |

**Table S3.** Information on genotyping and quality control. *Related to STAR Methods' section "Genotyping".*

| Cohort | HWE | MAF | Call Rate | Association software | Imputation | Reference | Genotype Platform |
|---|---|---|---|---|---|---|---|
| **European samples** | | | | | | | |
| **1000 Brains** | 1x10-6 | 0.01 | 0.95 | plink 1.9 | minimac (release 2013-07-17) | EUR 1000 Genomes (phase 1 version 3; Nov 2010) | Illumina Human OmniExpress12, Human CoreExome12, HumanOmni1-Quad, Infinium OmniExpressExome-8 |
| **3C-Bordeaux** | 1x10-6 | 0.01 | 0.95 | plink 1.9 | minimac3 | Haplotype Reference Consortium 1.1 | Illumina Human 610-Quad BeadChip |
| **ARIC whites** | 1x10-6 | 0.01 | 0.95 | probABEL | minimac3 | Haplotype Reference Consortium 1.1 | Affymetrix 6.0 |
| **ASRB** | 1x10-6 | 0.01 | 0.95 | mach2qtl | minimac4 | EUR 1000 Genomes (phase 1 version 5; February 18 2015) | Illumina Human610-Quad BeadChip |
| **CARDIA whites** | 1x10-4 | 0.03 | 0.95 | probABEL | BEAGLE | ALL 1000 Genomes (phase 1 version 3; Nov 2010) | Affymetrix 6.0 |
| **CROMIS-2 ICH** | 1x10-8 | 0.01 | 0.95 | SNPTEST | Michigang Imputation Server | Haplotype Reference Consortium 1.1 | Illumina Infinium® GlobalScreeningArray-24v1.0 |
| **DHS** | 1x10-6 | 0.05 | 0.95 | GWAF | IMPUTE2 | ALL 1000 Genomes (phase 1 version 3; Nov 2010) | Affymetrix 5.0 |
| **DNS** | 1x10-6 | 0.01 | 0.9 | plink 1.9 | IMPUTE2 | full 1000 Genomes Project Phase 3 (May 2013, >70 million variants, release "v5a") | Illumina HumanOmniExpress/Illumina HumanOmniExpress-24 |
| **EPOZ** | 1x10-4 | - | 0.975 | rvtest | minimac3 | Haplotype Reference Consortium 1.1 | Infinium Global Screening Array |
| **ESS** | 1x10-7 | - | 0.975 | rvtest | minimac3 | Haplotype Reference Consortium 1.1 | Metabochip |

| | | | | | | | |
|---|---|---|---|---|---|---|---|
| **FHS** | 1x10-6 | 0.01 | 0.97 | Perl and R | MACH/minimac | EUR 1000 Genomes (phase 1 version 3; March 2012) | Affymetrix 500K (250K Nsp & 250K Sty), MIPS 50K |
| **GenR** | 1x10-6 | 0.01 | 0.98 | rvtest | minimac3 | Haplotype Reference Consortium 1.1 | Illumina 610 Quad array |
| **HUNT** | 1x10-6 | 0.01 | 0.95 | mach2qtl (1.1.2) | minimac (release 2013-07-17) | EUR 1000 Genomes (phase 1 version 3; Nov 2010) | Illumina Omni 2.5M BeadChip array |
| **LIFE-Adult** | 1x10-6 | - | 0.97 | plink 1.9 | IMPUTE v2.3.2 | 1000 Genomes (phase 1 version 3; March 2012) | Affymetrix Axiom-CEU |
| **Poznan MS** | NA | NA | NA | rvtest | minimac3 | Haplotype Reference Consortium 1.1 | Illumina HumanOmniExpress/Illumina HumanOmniExpress-24 |
| **RSI** | 1x10-6 | 0.01 | 0.98 | rvtest | minimac3 | Haplotype Reference Consortium 1.1 | Illumina Human 550 (+duo) and 610-Quad BeadChip |
| **RSII** | 1x10-6 | 0.01 | 0.98 | rvtest | minimac3 | Haplotype Reference Consortium 1.1 | Illumina Human 550 duo |
| **RSIII** | 1x10-6 | 0.01 | 0.98 | rvtest | minimac3 | Haplotype Reference Consortium 1.1 | Illumina Human610-Quad BeadChip |
| **SHIP-TREND** | 1x10-4 | - | 0.94 | EPACTS | minimac3 | Haplotype Reference Consortium 1.1 | Illumina GSA-24 |
| **SYS adolescents** | 1x10-6 | 0.01 | 0.95 | probABEL | IMPUTE v2.2.2 | EUR 1000 Genomes (phase 1 version 3; March 2012) | Illumina Human610-Quad BeadChip (610K SNPs) & Illumina HumanOmniExpress BeadChip |
| **SYS adults** | 1x10-6 | 0.01 | 0.95 | probABEL | IMPUTE v2.2.2 | EUR 1000 Genomes (phase 1 version 3; March 2012) | Illumina Human610-Quad BeadChip (610K SNPs) & Illumina HumanOmniExpress BeadChip |
| **UK Biobank** | 1x10-6 | 0.01 | 0.9 | HASE | IMPUTE4 | Haplotype Reference Consortium 1.1 | Applied Biosystems™ UK BiLEVE Axiom™ Array by Affymetrix and Applied Biosystems™ UK Biobank Axiom™ Array |
| **VIDI** | - | - | 0.95 | plink 1.9 | IMPUTE2 | 1000 Genomes (phase 3 version 5) | Illumina Infinium Global Screening Array |

| | | | | | | | |
|---|---|---|---|---|---|---|---|
| **Non-European/mixed ancestry samples** | | | | | | | |
| **ARIC-Black** | 1x10-6 | 0.01 | 0.95 | probABEL | IMPUTE2 2.2.2 | ALL 1000 Genomes (phase 1 version 3; Nov 2010) | Affymetrix 6.0 |
| **CARDIA-Black** | 1x10-4 | 0.03 | 0.95 | probABEL | BEAGLE | ALL 1000 Genomes (phase 1 version 3; Nov 2010) | Affymetrix 6.0 |
| **FBIRN** | 1x10-6 | 0.01 | 0.95 | raremetal/4.14.1 | Michigan Imputation | ALL 1000 Genomes (phase 1 version 3; Nov 2010) | Illumina MEGA+Psych chip |
| **HKOS** | 1x10-6 | 0.01 | 0.95 | GCTA MLMA | Michigan Imputation Server | Haplotype Reference Consortium 1.1 | Illumina Human 610-Quad BeadChip & Illumina Global Screening Array |
| **IMH** | 1x10-6 | 0.01 | 0.95 | rvtest | - | - | Illumina 1M duo3 array |
| **Meth-CT** | 1x10-6 | 0.01 | 0.95 | mach2qtl (1.1.2) | minimac (release 2013-07-17) | ALL 1000 Genomes (phase 1 version 3; Nov 2010) | Infinium PsychArray-24 v1.1 BeadChip |
| **Nagahama** | 1x10-7 | 0.01 | 0.99 | rvtest | Michigan Imputation Server | Japanese 3,000 reference panel (AGP) | Human 610K Quad, HumanOmni2.5-4, HumanOmni2.5-8, HumanOmni2.5s, HumanExome and Human core exome, 5.0M and ASA (Illumina, San Diego, CA, USA) |
| **Validation samples** | | | | | | | |
| **BIL&GIN** | 1x10-6 | 0.01 | 0.95 | plink 1.9 | minimac | Haplotype Reference Consortium 1.1 | Illumina InfiniumOmniExpressExome-8v1-4 |
| **i-Share** | 1x10-3 | 0.01 | 0.98 | GCTA MLMA | minimac4 | Haplotype Reference Consortium 1.1 | Affymetrix Precision Medicine Axiom Array |
| **UK Biobank (independent sample)** | 1x10-6 | 0.01 | 0.9 | BOLT-LMM | IMPUTE4 | Haplotype Reference Consortium version 1.1, UK10K and 1000Genomes | Applied Biosystems™ UK BiLEVE Axiom™ Array by Affymetrix and Applied Biosystems™ UK Biobank Axiom™ Array |

**Table S5.** Lambda genomic control, LD score regression intercept and ratio, and SNP-based heritability for different models. *Related to STAR Methods' section "Genome-wide association studies".*

| **Model** | **Lambda.GC** | **Intercept** | **SE intercept** | **Ratio** | **SE ratio** | **Heritability** | **SE heritability** |
|---|---|---|---|---|---|---|---|
| Model 1 | 1.2865 | 1.0562 | 0.0106 | 0.1315 | 0.0249 | 0.2487 | 0.0164 |
| Model 1 height subset | 1.2664 | 1.0488 | 0.0093 | 0.1320 | 0.0252 | 0.2935 | 0.0210 |
| Model 1 new cohorts | 1.1715 | 1.0247 | 0.0087 | 0.1031 | 0.0363 | 0.2448 | 0.0198 |
| Model 2 | 1.2498 | 1.0461 | 0.0086 | 0.1351 | 0.0252 | 0.2970 | 0.0205 |

**Table S10.** Overlap between identified loci and previously identified loci in genome-wide association studies of brain volumes.
*Related to Figure 2A.*
*Highest linkage disequilibrium (r2) value with a genome-wide significant genetic variant is presented in brackets.*
*Abbreviations: HC - head circumference, ICV - intracranial volume.*

| Genomic Locus | rsID | trait1 | trait2 | trait3 | trait4 |
|---|---|---|---|---|---|
| 6 | rs6429430 | HC + ICV, Haworth et al. 2019 (r2=1) | | | |
| 7 | rs6736289 | HC + ICV, Haworth et al. 2019 (r2=1) | | | |
| 9 | rs62135193 | Superiortemporal, Grasby et al. 2020 (r2=1) | | | |
| 10 | rs41288837 | HC + ICV, Haworth et al. 2019 (r2=1) | | | |
| 11 | rs867529 | Brainstem, Satizabal et al. 2019 (r2=1) | | | |
| 12 | rs288326 | HC + ICV, Haworth et al. 2019 (r2=1) | HC, Haworth et al. 2019 (r2=1) | | |
| 17 | rs2063453 | HC + ICV, Haworth et al. 2019 (r2=1) | | | |
| 18 | rs1159211 | Accumbens, Satizabal et al. 2019 (r2=1) | Precuneus, Grasby et al. 2020 (r2=1) | Lateral ventricles, Vojinovic et al. 2018 (r2=1) | HC + ICV, Haworth et al. 2019 (r2=0.798) |
| 18 | rs9821713 | HC + ICV, Haworth et al. 2019 (r2=1) | Accumbens, Satizabal et al. 2019 (r2=1) | Lateral ventricles, Vojinovic et al. 2018 (r2=1) | |
| 23 | rs1935952 | HC + ICV, Haworth et al. 2019 (r2=1) | Brainstem, Satizabal et al. 2019 (r2=1) | | |
| 24 | 6-126845438 | HC + ICV, Haworth et al. 2019 (r2=1) | ICV, Adams et al. 2016 (r2=1) | | |

| | | | | | |
|---|---|---|---|---|---|
| 24 | rs11154343 | HC + ICV, Haworth et al. 2019 (r2=1) | | | |
| 24 | rs17650496 | HC + ICV, Haworth et al. 2019 (r2=1) | ICV, Adams et al. 2016 (r2=1) | | |
| 24 | rs190958130 | Pericalcarine, Grasby et al. 2020 (r2=1) | | | |
| 24 | rs2011008 | HC + ICV, Haworth et al. 2019 (r2=1) | ICV, Adams et al. 2016 (r2=1) | | |
| 24 | rs4273712 | HC + ICV, Haworth et al. 2019 (r2=1) | ICV, Adams et al. 2016 (r2=1) | Caudalmiddlefrontal, Grasby et al. 2020 (r2=1) | |
| 24 | rs9401873 | ICV, Adams et al. 2016 (r2=1) | HC + ICV, Haworth et al. 2019 (r2=1) | | |
| 28 | rs34888260 | HC + ICV, Haworth et al. 2019 (r2=0.992) | | | |
| 29 | rs2072235 | Putamen, Satizabal et al. 2019 (r2=0.654) | | | |
| 30 | rs151057105 | Thalamus, Satizabal et al. 2019 (r2=0.991) | | | |
| 37 | rs11012732 | Lateral ventricles, Vojinovic et al. 2018 (r2=0.897) | | | |
| 39 | rs1628768 | HC + ICV, Haworth et al. 2019 (r2=1) | ICV, Adams et al. 2016 (r2=1) | | |
| 42 | rs3217870 | HC + ICV, Haworth et al. 2019 (r2=1) | | | |
| 43 | rs2066827 | HC + ICV, Haworth et al. 2019 (r2=1) | | | |
| 46 | rs17178006 | Hippocampus, Hibar et al. 2017 (r2=1) | Amygdala, Satizabal et al. 2019 (r2=1) | | |
| 47 | rs7306710 | HC + ICV, Haworth et al. 2019 (r2=1) | | | |
| 50 | rs11111293 | Amygdala, Satizabal et al. 2019 (r2=1) | Brainstem, Satizabal et al. 2019 (r2=0.753) | | |

| | | | | | |
|---|---|---|---|---|---|
| 51 | rs28636834 | HC + ICV, Haworth et al. 2019 (r2=1) | HC, Haworth et al. 2019 (r2=1) | | |
| 57 | rs78378222 | HC + ICV, Haworth et al. 2019 (r2=1) | HC, Haworth et al. 2019 (r2=1) | | |
| 60 | rs4564621 | ICV, Adams et al. 2016 (r2=1) | HC + ICV, Haworth et al. 2019 (r2=1) | Fusiform, Grasby et al. 2020 (r2=1) | Frontalpole, Grasby et al. 2020 (r2=1) |
| 60 | rs8079695 | ICV, Adams et al. 2016 (r2=1) | Fusiform, Grasby et al. 2020 (r2=1) | | |
| 63 | rs148340480 | Paracentral, Grasby et al. 2020 (r2=0.805) | | | |
| 67 | rs10483213 | HC + ICV, Haworth et al. 2019 (r2=1) | | | |

**Table S14.** Genetic correlation between the human head size and neuropsychiatric traits. *Related to Figure S3, Figure 1C and STAR Methods' section "Genetic correlations".*
*Abbreviations: P - P-value; Rg - genetic correlation estimate; SE - standard error.*
*Bold p-values represent Bonferroni significant correlations (P < (0.05/16))*

| | | **Model 1 - full set** | | | **Model 1 - new set** | | |
|---|---|---|---|---|---|---|---|
| **Trait** | **Category** | **Rg** | **SE** | **P** | **Rg** | **SE** | **P** |
| Educational attainment | Cognitive functioning | 0.2599 | 0.0210 | **3.98E-35** | 0.2356 | 0.0248 | **1.90E-21** |
| General cognitive function | Cognitive functioning | 0.2340 | 0.0240 | **2.06E-22** | 0.2251 | 0.0272 | **1.14E-16** |
| All stroke | Neurological disorders | -0.0295 | 0.0438 | 5.00E-01 | 0.0003 | 0.0517 | 9.95E-01 |
| Alzheimer's disease | Neurological disorders | -0.0950 | 0.0743 | 2.01E-01 | -0.1506 | 0.0875 | 8.54E-02 |
| Frontotemporal dementia | Neurological disorders | 0.1335 | 0.1569 | 3.95E-01 | 0.2343 | 0.1886 | 2.14E-01 |
| Parkinson's disease | Neurological disorders | 0.2035 | 0.0446 | **5.07E-06** | 0.2049 | 0.0487 | **2.54E-05** |
| Anorexia nervosa | Psychiatric traits | 0.0864 | 0.0515 | 9.35E-02 | 0.0187 | 0.0656 | 7.76E-01 |
| Attention-deficit hyperactivity disorder | Psychiatric traits | -0.1804 | 0.0357 | **4.46E-07** | -0.1421 | 0.0410 | **5.37E-04** |
| Autism spectrum disorder | Psychiatric traits | 0.0746 | 0.0505 | 1.40E-01 | 0.1006 | 0.0671 | 1.34E-01 |
| Bipolar disorder | Psychiatric traits | 0.0464 | 0.0293 | 1.14E-01 | 0.0521 | 0.0368 | 1.57E-01 |
| Extraversion | Psychiatric traits | -0.0557 | 0.0621 | 3.70E-01 | -0.0664 | 0.0722 | 3.58E-01 |
| Insomnia | Psychiatric traits | -0.1899 | 0.0443 | **1.78E-05** | -0.1759 | 0.0509 | **5.47E-04** |
| Major depressive disorder | Psychiatric traits | -0.1098 | 0.0300 | **2.57E-04** | -0.0983 | 0.0358 | 5.98E-03 |
| Neuroticism | Psychiatric traits | -0.1101 | 0.0319 | **5.43E-04** | -0.0887 | 0.0355 | 1.23E-02 |
| Obsessive compulsive disorder | Psychiatric traits | 0.1298 | 0.0526 | 1.35E-02 | 0.1733 | 0.0634 | 6.26E-03 |
| Schizophrenia | Psychiatric traits | -0.0171 | 0.0287 | 5.51E-01 | -0.0363 | 0.0336 | 2.80E-01 |

**Table S15.** Kyoto Encyclopaedia of Genes and Genomes (KEGG) pathway analysis. *Related to Figure 3A.*

| GeneSet | N_genes | N_overlap | p | adjP | genes | link |
|---|---|---|---|---|---|---|
| KEGG ENDOMETRIAL CANCER | 52 | 7 | 4.71E-06 | 0.000321 | AKT3, PTEN, TP53, TCF7L1, MAPK1, APC, FOXO3 | http://www.broadinstitute.org/gsea/msigdb/cards/KEGG_ENDOMETRIAL_CANCER |
| KEGG CELL CYCLE | 124 | 10 | 5.16E-06 | 0.000321 | CCND2, CDKN1B, ESPL1, CDK2, TP53, MCM2, STAG1, TFDP2, ATR, CDK6 | http://www.broadinstitute.org/gsea/msigdb/cards/KEGG_CELL_CYCLE |
| KEGG PATHWAYS IN CANCER | 325 | 16 | 5.95E-06 | 0.000321 | WNT2B, AKT3, PTEN, CDKN1B, CDK2, IGF1, TP53, FZD2, WNT3, TCF7L1, PLCG1, MAPK1, LAMB2, APC, CDK6, PTCH1 | http://www.broadinstitute.org/gsea/msigdb/cards/KEGG_PATHWAYS_IN_CANCER |
| KEGG BASAL CELL CARCINOMA | 55 | 7 | 6.90E-06 | 0.000321 | WNT2B, TP53, FZD2, WNT3, TCF7L1, APC, PTCH1 | http://www.broadinstitute.org/gsea/msigdb/cards/KEGG_BASAL_CELL_CARCINOMA |
| KEGG PROSTATE CANCER | 88 | 8 | 1.92E-05 | 0.000658 | AKT3, PTEN, CDKN1B, CDK2, IGF1, | http://www.broadinstitute.org/gsea/msigdb/cards/KEGG_PROSTATE_CANCER |

| | | | | | | |
|---|---|---|---|---|---|---|
| | | | | | TP53, TCF7L1, MAPK1 | |
| KEGG GLIOMA | 65 | 7 | 2.12E-05 | 0.000658 | AKT3, PTEN, IGF1, TP53, PLCG1, MAPK1, CDK6 | http://www.broadinstitute.org/gsea/msigdb/cards/KEGG_GLIOMA |
| KEGG P53 SIGNALING PATHWAY | 68 | 7 | 2.86E-05 | 0.000759 | PTEN, CCND2, CDK2, IGF1, TP53, ATR, CDK6 | http://www.broadinstitute.org/gsea/msigdb/cards/KEGG_P53_SIGNALING_PATHWAY |
| KEGG NON SMALL CELL LUNG CANCER | 54 | 6 | 6.99E-05 | 0.001624 | AKT3, TP53, PLCG1, MAPK1, FOXO3, CDK6 | http://www.broadinstitute.org/gsea/msigdb/cards/KEGG_NON_SMALL_CELL_LUNG_CANCER |
| KEGG SMALL CELL LUNG CANCER | 84 | 7 | 0.000112 | 0.002313 | AKT3, PTEN, CDKN1B, CDK2, TP53, LAMB2, CDK6 | http://www.broadinstitute.org/gsea/msigdb/cards/KEGG_SMALL_CELL_LUNG_CANCER |
| KEGG MELANOMA | 71 | 6 | 0.000322 | 0.005995 | AKT3, PTEN, IGF1, TP53, MAPK1, CDK6 | http://www.broadinstitute.org/gsea/msigdb/cards/KEGG_MELANOMA |
| KEGG WNT SIGNALING PATHWAY | 148 | 8 | 0.000722 | 0.012211 | WNT2B, LRP5, CCND2, TP53, FZD2, WNT3, TCF7L1, APC | http://www.broadinstitute.org/gsea/msigdb/cards/KEGG_WNT_SIGNALING_PATHWAY |
| KEGG ERBB SIGNALING PATHWAY | 87 | 6 | 0.000956 | 0.014823 | AKT3, CDKN1B, ERBB3, PLCG1, MAPK1, NCK1 | http://www.broadinstitute.org/gsea/msigdb/cards/KEGG_ERBB_SIGNALING_PATHWAY |

| | | | | | | |
|---|---|---|---|---|---|---|
| KEGG COLORECTAL CANCER | 62 | 5 | 0.001266 | 0.018109 | AKT3, TP53, TCF7L1, MAPK1, APC | http://www.broadinstitute.org/gsea/msigdb/cards/KEGG_COLORECTAL_CANCER |
| KEGG CHRONIC MYELOID LEUKEMIA | 73 | 5 | 0.002618 | 0.034778 | AKT3, CDKN1B, TP53, MAPK1, CDK6 | http://www.broadinstitute.org/gsea/msigdb/cards/KEGG_CHRONIC_MYELOID_LEUKEMIA |

**Table S22.** Genetic correlation between the human head size and different cancer types. *Related to STAR Methods' section "Genetic correlations".*

*Abbreviations: P - P-value; Rg - genetic correlation estimate; SE - standard error.*

*Bold p-values represent nominally significant correlations (P < 0.05). No correlations survived Bonferroni multiple testing correction (P < (0.05/15)).*

| | | **Model 1 - full set** | | | **Model 1 - new set** | | |
|---|---|---|---|---|---|---|---|
| **Trait** | **Category** | **Rg** | **SE** | **P** | **Rg** | **SE** | **P** |
| Breast cancer Zhang et al. | Cancer | -0.0117 | 0.0293 | 6.89E-01 | -0.0173 | 0.0309 | 5.77E-01 |
| Ovarian cancer Phelan et al. | Cancer | -0.1831 | 0.0646 | **4.62E-03** | -0.1637 | 0.0682 | **1.63E-02** |
| Prostate cancer Schumacher et al. | Cancer | -0.0033 | 0.0379 | 9.30E-01 | -0.0520 | 0.0405 | 1.99E-01 |
| Bladder cancer Rashkin et al. | Cancer | -0.0699 | 0.1101 | 5.26E-01 | -0.1205 | 0.1258 | 3.38E-01 |
| Breast cancer Rashkin et al. | Cancer | 0.0199 | 0.0433 | 6.45E-01 | 0.0430 | 0.0451 | 3.41E-01 |
| Cervical cancer Rashkin et al. | Cancer | -0.0962 | 0.0747 | 1.97E-01 | -0.0929 | 0.0830 | 2.63E-01 |
| Colon cancer Rashkin et al. | Cancer | -0.0023 | 0.0770 | 9.76E-01 | 0.0449 | 0.0789 | 5.69E-01 |
| Endometrial cancer Rashkin et al. | Cancer | -0.0297 | 0.0795 | 7.09E-01 | 0.0518 | 0.0877 | 5.55E-01 |
| Oesphageal and gastric cancer Rashkin et al. | Cancer | 0.0800 | 0.1399 | 5.68E-01 | -0.0038 | 0.1548 | 9.81E-01 |
| Kidney cancer Rashkin et al. | Cancer | -0.0525 | 0.0850 | 5.37E-01 | -0.1515 | 0.0907 | 9.51E-02 |
| Lung cancer Rashkin et al. | Cancer | 0.0952 | 0.0590 | 1.07E-01 | 0.0813 | 0.0720 | 2.59E-01 |
| Malignant melanoma Rashkin et al. | Cancer | 0.0519 | 0.0905 | 5.66E-01 | 0.0013 | 0.1044 | 9.90E-01 |
| Non-Hodgkin lymphoma Rashkin et al. | Cancer | 0.0774 | 0.2177 | 7.22E-01 | 0.1016 | 0.2201 | 6.45E-01 |
| Prostate cancer Rahskin et al. | Cancer | 0.0699 | 0.0417 | 9.40E-02 | 0.0564 | 0.0477 | 2.37E-01 |
| Rectal cancer Rashkin et al. | Cancer | -0.0445 | 0.0859 | 6.04E-01 | -0.0072 | 0.0973 | 9.41E-01 |

**Table S24.** Enrichment of head size GWAS for brain cell types based on human scRNA-seq data. *Related to Figure S2.*

| **Cell type or subtype** | **(Sub)type** | **Enrichment** | **Enrichment SE** | **Enrichment P** | **P** | **FDR** |
|---|---|---|---|---|---|---|
| Cell type | IPC | 2.023 | 0.1437 | 3.14E-11 | 0.0005 | 0.0050 |
| Cell type | Endothelial | 1.742 | 0.1226 | 8.30E-09 | 0.7043 | 0.7825 |
| Cell type | Red blood cells | 1.672 | 0.1191 | 6.85E-08 | 0.0226 | 0.1131 |
| Cell type | Microglia | 1.693 | 0.1359 | 9.06E-07 | 0.3655 | 0.6092 |
| Cell type | Mural | 1.611 | 0.1229 | 1.21E-06 | 0.9050 | 0.9050 |
| Cell type | Radial glia | 1.600 | 0.1216 | 2.51E-06 | 0.1545 | 0.3863 |
| Cell type | Inhibitory neuron | 1.406 | 0.1018 | 0.000112 | 0.2911 | 0.5822 |
| Cell type | Excitatory neuron | 1.291 | 0.0980 | 0.003729 | 0.1111 | 0.3703 |
| Cell type | OPC | 1.240 | 0.0934 | 0.010566 | 0.5065 | 0.6968 |
| Cell subtype | vRG | 2.149 | 0.1543 | 5.61E-12 | 0.0009 | 0.0090 |
| Cell subtype | IPC.div1 | 2.243 | 0.1608 | 1.05E-11 | 0.0002 | 0.0064 |
| Cell subtype | tRG | 1.904 | 0.1338 | 3.51E-11 | 0.0423 | 0.1588 |
| Cell subtype | IPC.new | 2.053 | 0.1491 | 1.05E-10 | 0.0042 | 0.0313 |
| Cell subtype | Newborn_Neuron | 1.844 | 0.1236 | 2.26E-10 | 0.0006 | 0.0090 |
| Cell subtype | Endothelial | 1.807 | 0.1260 | 1.48E-09 | 0.4992 | 0.6511 |
| Cell subtype | Layer_VI_Occipital | 1.811 | 0.1338 | 1.99E-09 | 0.0869 | 0.2606 |
| Cell subtype | early_RG | 1.920 | 0.1490 | 3.11E-09 | 0.0831 | 0.2606 |
| Cell subtype | IPC.div2 | 1.968 | 0.1533 | 3.46E-09 | 0.0271 | 0.1356 |
| Cell subtype | PFC | 1.779 | 0.1330 | 1.11E-08 | 0.1159 | 0.3074 |
| Cell subtype | IPCnewborn | 2.000 | 0.1638 | 1.12E-08 | 0.0103 | 0.0620 |
| Cell subtype | oRG | 1.711 | 0.1273 | 3.53E-08 | 0.1873 | 0.4015 |
| Cell subtype | SST.MGE1 | 1.610 | 0.1131 | 2.26E-07 | 0.2219 | 0.4160 |
| Cell subtype | oRGAstrocyte | 1.641 | 0.1269 | 5.81E-07 | 0.2835 | 0.4476 |
| Cell subtype | Microglia | 1.738 | 0.1434 | 6.50E-07 | 0.8210 | 0.8493 |

| | | | | | | |
|---|---|---|---|---|---|---|
| Cell subtype | Mural | 1.638 | 0.1285 | 1.60E-06 | 0.8765 | 0.8765 |
| Cell subtype | late_RG | 1.495 | 0.1042 | 4.33E-06 | 0.7382 | 0.7909 |
| Cell subtype | Upper_Layer_Occipital | 1.529 | 0.1152 | 2.02E-05 | 0.1383 | 0.3192 |
| Cell subtype | Cajal_Retzius | 1.347 | 0.1047 | 0.000944 | 0.4594 | 0.6265 |
| Cell subtype | Parietal_and_Temporal | 1.314 | 0.0957 | 0.001451 | 0.1229 | 0.3074 |
| Cell subtype | Upper_Layer_PFC | 1.356 | 0.1170 | 0.002683 | 0.6781 | 0.7557 |
| Cell subtype | OPC | 1.293 | 0.1004 | 0.003865 | 0.6801 | 0.7557 |
| Cell subtype | Upper_Layer | 1.305 | 0.1052 | 0.005159 | 0.2009 | 0.4019 |
| Cell subtype | Deep_Layer | 1.270 | 0.1072 | 0.012312 | 0.6409 | 0.7557 |
| Cell subtype | MGE2 | 1.209 | 0.0848 | 0.016121 | 0.2697 | 0.4476 |
| Cell subtype | Layer_IV | 1.225 | 0.0951 | 0.020491 | 0.4418 | 0.6265 |
| Cell subtype | Layer_VI_Pan.area | 1.177 | 0.0884 | 0.045774 | 0.3694 | 0.5541 |

**Table S25.** Enrichment of gene sets for brain cell types based on human scRNA-seq data. *Related to Figure 4E.*

| Gene list | Cell type | Fold change | P | FDR |
|---|---|---|---|---|
| Head size intragenic lead variant genes | Endothelial | 1.1362 | 0.4530 | 0.8965 |
| Head size intragenic lead variant genes | Excitatory neuron | 0.9083 | 0.6545 | 0.9999 |
| Head size intragenic lead variant genes | Inhibitory neuron | 0.9090 | 0.6539 | 0.9896 |
| Head size intragenic lead variant genes | IPC | 2.4997 | 3.31E-03 | 0.0088 |
| Head size intragenic lead variant genes | Microglia | 0.2271 | 0.9904 | 0.9973 |
| Head size intragenic lead variant genes | Mural | 1.1354 | 0.4536 | 0.9997 |
| Head size intragenic lead variant genes | OPC | 0.6808 | 0.8308 | 0.9996 |
| Head size intragenic lead variant genes | Radial glia | 0.9090 | 0.6539 | 0.9908 |
| Head size intragenic lead variant genes | Red blood cells | 1.5907 | 0.1454 | 0.4105 |
| Head size intragenic genes | Endothelial | 1.2421 | 0.1827 | 0.7306 |
| Head size intragenic genes | Excitatory neuron | 0.8688 | 0.7491 | 0.9999 |
| Head size intragenic genes | Inhibitory neuron | 0.9937 | 0.5497 | 0.9896 |
| Head size intragenic genes | IPC | 1.6147 | 9.58E-03 | 0.0192 |
| Head size intragenic genes | Microglia | 0.6206 | 0.9669 | 0.9973 |
| Head size intragenic genes | Mural | 0.6206 | 0.9669 | 0.9997 |
| Head size intragenic genes | OPC | 0.5582 | 0.9843 | 0.9996 |
| Head size intragenic genes | Radial glia | 0.8694 | 0.7482 | 0.9908 |
| Head size intragenic genes | Red blood cells | 1.1800 | 0.2565 | 0.4105 |
| Head size genes 10kb distance | Endothelial | 0.9919 | 0.5486 | 0.8965 |
| Head size genes 10kb distance | Excitatory neuron | 0.7930 | 0.8901 | 0.9999 |
| Head size genes 10kb distance | Inhibitory neuron | 0.9919 | 0.5486 | 0.9896 |
| Head size genes 10kb distance | IPC | 1.6664 | 6.43E-04 | 0.0026 |
| Head size genes 10kb distance | Microglia | 1.0705 | 0.3836 | 0.9973 |
| Head size genes 10kb distance | Mural | 0.7930 | 0.8901 | 0.9997 |
| Head size genes 10kb distance | OPC | 0.5547 | 0.9962 | 0.9996 |
| Head size genes 10kb distance | Radial glia | 0.9919 | 0.5486 | 0.9908 |

| | | | | |
|---|---|---|---|---|
| Head size genes 10kb distance | Red blood cells | 0.9522 | 0.6318 | 0.8424 |
| Head size genes 100kb distance | Endothelial | 0.9869 | 0.5603 | 0.8965 |
| Head size genes 100kb distance | Excitatory neuron | 0.7267 | 0.9753 | 0.9999 |
| Head size genes 100kb distance | Inhibitory neuron | 1.0128 | 0.4917 | 0.9896 |
| Head size genes 100kb distance | IPC | 1.3245 | 0.0226 | 0.0361 |
| Head size genes 100kb distance | Microglia | 1.0381 | 0.4255 | 0.9973 |
| Head size genes 100kb distance | Mural | 0.7786 | 0.9441 | 0.9997 |
| Head size genes 100kb distance | OPC | 0.5706 | 0.9992 | 0.9996 |
| Head size genes 100kb distance | Radial glia | 0.8311 | 0.8883 | 0.9908 |
| Head size genes 100kb distance | Red blood cells | 0.9090 | 0.7511 | 0.8584 |
| Head size genes 1Mb distance | Endothelial | 0.8962 | 0.9124 | 0.9949 |
| Head size genes 1Mb distance | Excitatory neuron | 0.9034 | 0.8969 | 0.9999 |
| Head size genes 1Mb distance | Inhibitory neuron | 0.9430 | 0.7756 | 0.9896 |
| Head size genes 1Mb distance | IPC | 1.0443 | 0.3040 | 0.3474 |
| Head size genes 1Mb distance | Microglia | 0.9112 | 0.8778 | 0.9973 |
| Head size genes 1Mb distance | Mural | 0.9190 | 0.8564 | 0.9997 |
| Head size genes 1Mb distance | OPC | 0.7471 | 0.9996 | 0.9996 |
| Head size genes 1Mb distance | Radial glia | 0.8417 | 0.9805 | 0.9908 |
| Head size genes 1Mb distance | Red blood cells | 1.0599 | 0.2403 | 0.4105 |
| OMIM macrocephaly genes | Endothelial | 0.4761 | 0.9583 | 0.9949 |
| OMIM macrocephaly genes | Excitatory neuron | 0.7930 | 0.7689 | 0.9999 |
| OMIM macrocephaly genes | Inhibitory neuron | 0.3174 | 0.9896 | 0.9896 |
| OMIM macrocephaly genes | IPC | 1.1110 | 0.4444 | 0.4444 |
| OMIM macrocephaly genes | Microglia | 0.7930 | 0.7689 | 0.9973 |
| OMIM macrocephaly genes | Mural | 0.6344 | 0.8875 | 0.9997 |
| OMIM macrocephaly genes | OPC | 0.9509 | 0.6136 | 0.9996 |
| OMIM macrocephaly genes | Radial glia | 1.1110 | 0.4444 | 0.9908 |
| OMIM macrocephaly genes | Red blood cells | 1.7458 | 0.0464 | 0.3715 |
| OMIM microcephaly genes | Endothelial | 0.6231 | 0.9949 | 0.9949 |

| | | | | |
|---|---|---|---|---|
| OMIM microcephaly genes | Excitatory neuron | 0.5930 | 0.9973 | 0.9999 |
| OMIM microcephaly genes | Inhibitory neuron | 1.0681 | 0.3630 | 0.9896 |
| OMIM microcephaly genes | IPC | 1.6022 | 3.26E-04 | 0.0026 |
| OMIM microcephaly genes | Microglia | 0.5930 | 0.9973 | 0.9973 |
| OMIM microcephaly genes | Mural | 0.5040 | 0.9997 | 0.9997 |
| OMIM microcephaly genes | OPC | 0.5333 | 0.9994 | 0.9996 |
| OMIM microcephaly genes | Radial glia | 0.6527 | 0.9908 | 0.9908 |
| OMIM microcephaly genes | Red blood cells | 1.1571 | 0.1876 | 0.4105 |
| COSMIC tier 1 cancer genes | Endothelial | 1.2939 | 0.0172 | 0.1373 |
| COSMIC tier 1 cancer genes | Excitatory neuron | 0.5486 | 0.9999 | 0.9999 |
| COSMIC tier 1 cancer genes | Inhibitory neuron | 0.7646 | 0.9734 | 0.9896 |
| COSMIC tier 1 cancer genes | IPC | 1.2155 | 0.0603 | 0.0805 |
| COSMIC tier 1 cancer genes | Microglia | 0.9796 | 0.5847 | 0.9973 |
| COSMIC tier 1 cancer genes | Mural | 1.3910 | 0.0025 | 0.0203 |
| COSMIC tier 1 cancer genes | OPC | 0.7636 | 0.9740 | 0.9996 |
| COSMIC tier 1 cancer genes | Radial glia | 1.0391 | 0.4044 | 0.9908 |
| COSMIC tier 1 cancer genes | Red blood cells | 0.8430 | 0.9018 | 0.9018 |

## Supplementary Figures

**Figure S1.** Genetic correlations with neuropsychiatric traits. *Related to Figure 1C.*

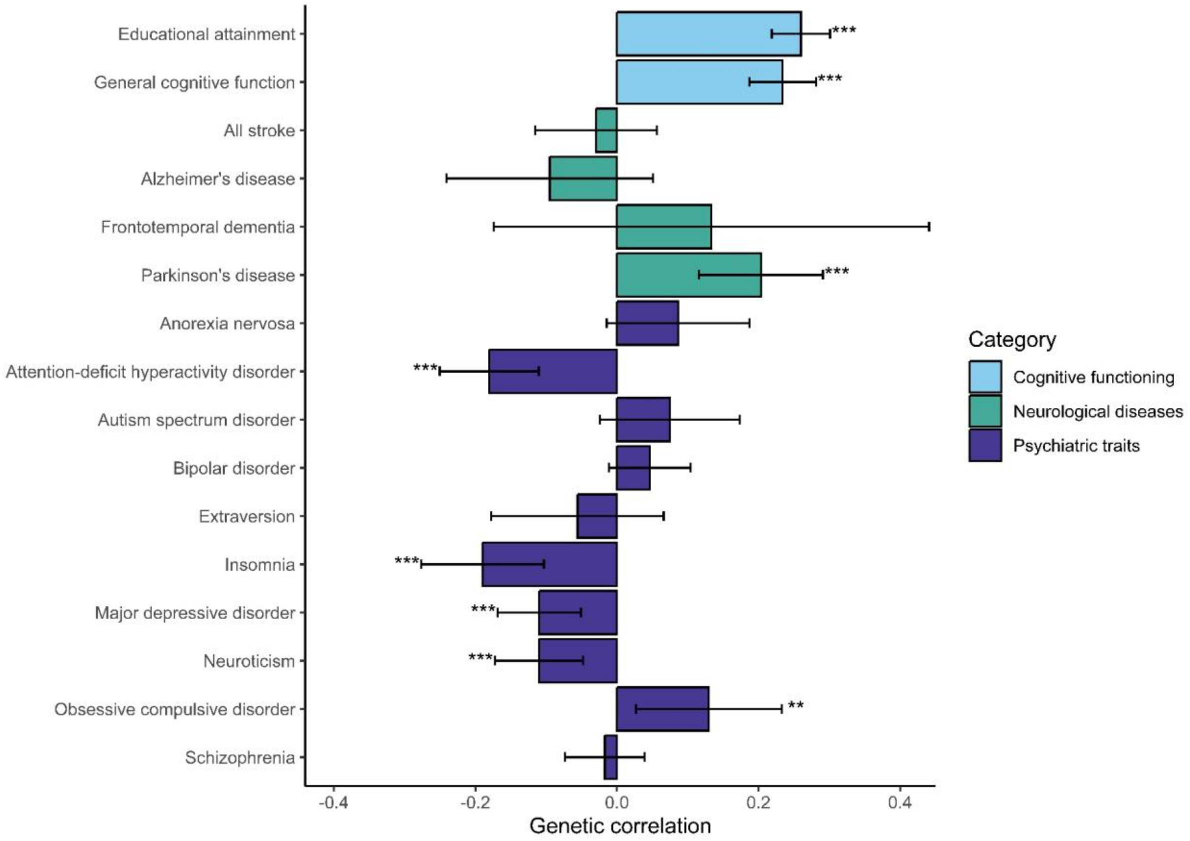


Genetic correlations of the human head size with cognitive functioning, neurological diseases and psychiatric traits. Analyses were performed in both the complete GWAS set, and in the GWAS set with only newly contributing studies. Significant results are denoted by asterisks: *$P$<0.05; **$P$<0.017 (0.05/3); ***$P$<0.003 (0.05/16).

**Figure S2.** Enrichment of expression of human head size GWAS genes in cell subtypes in the human cortical brain, derived from single-cell RNA sequencing data. *Related to Figure 4.*

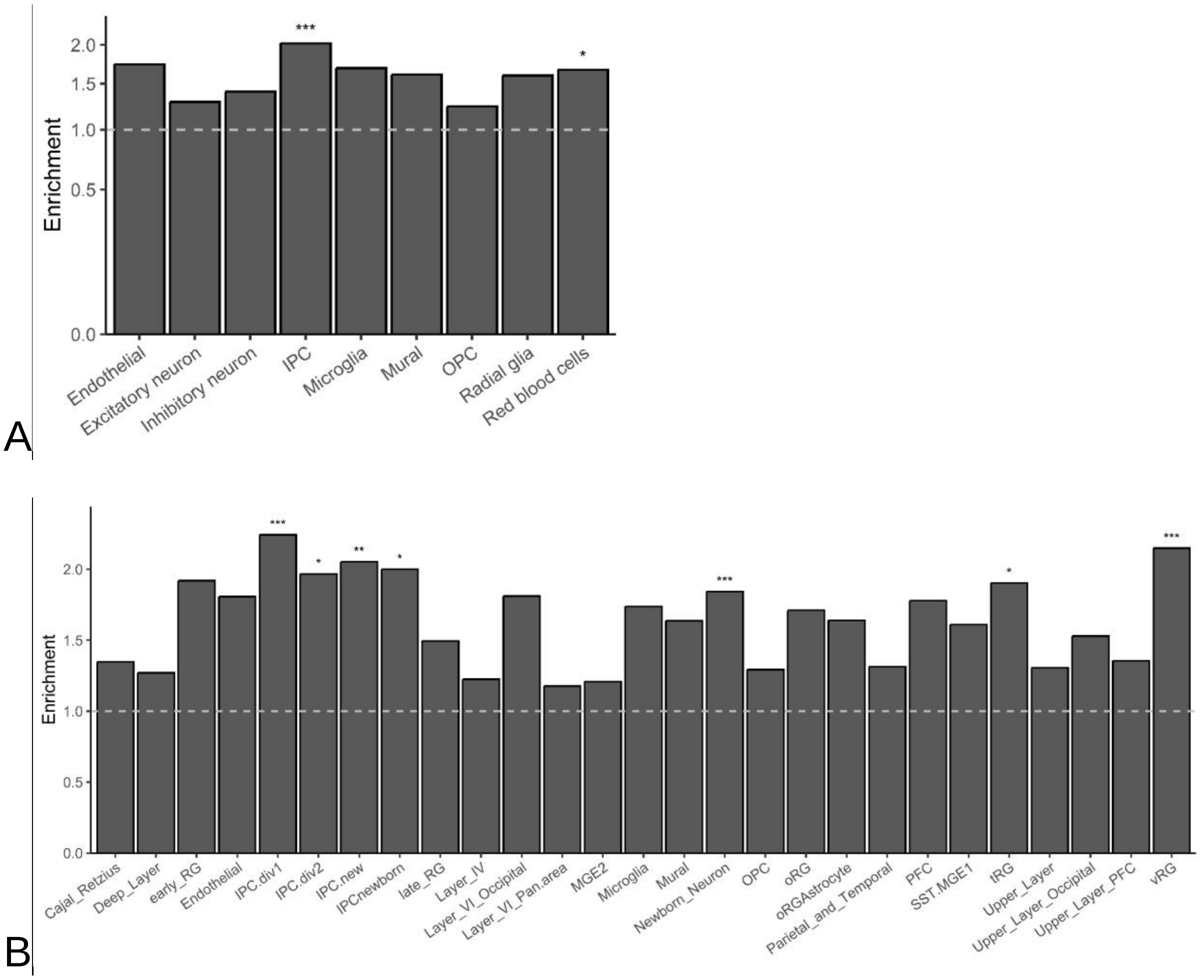


**(A)** Enrichment of expression of human head size GWAS genes in cell types of the human cortical brain, derived from single-cell RNA sequencing data. Significant results are denoted by asterisks: *$P$<0.05; **$FDR$<0.05; ***$P$<0.006 (0.05/9).

**(B)** Enrichment of expression of human head size GWAS genes in cell subtypes in the human cortical brain, derived from single-cell RNA sequencing data. Significant results are denoted by asterisks: *$P$<0.05; **$FDR$<0.05; ***$P$<0.002 (0.05/27).
