# Supplementary figures and images for: Genetic variants for head size share genes and pathways with cancer
Source: Cell Rep Med. 2024 May 3;5(5):101529. doi: 10.1016/j.xcrm.2024.101529 (PMC11148644; doi:10.1016/j.xcrm.2024.101529)

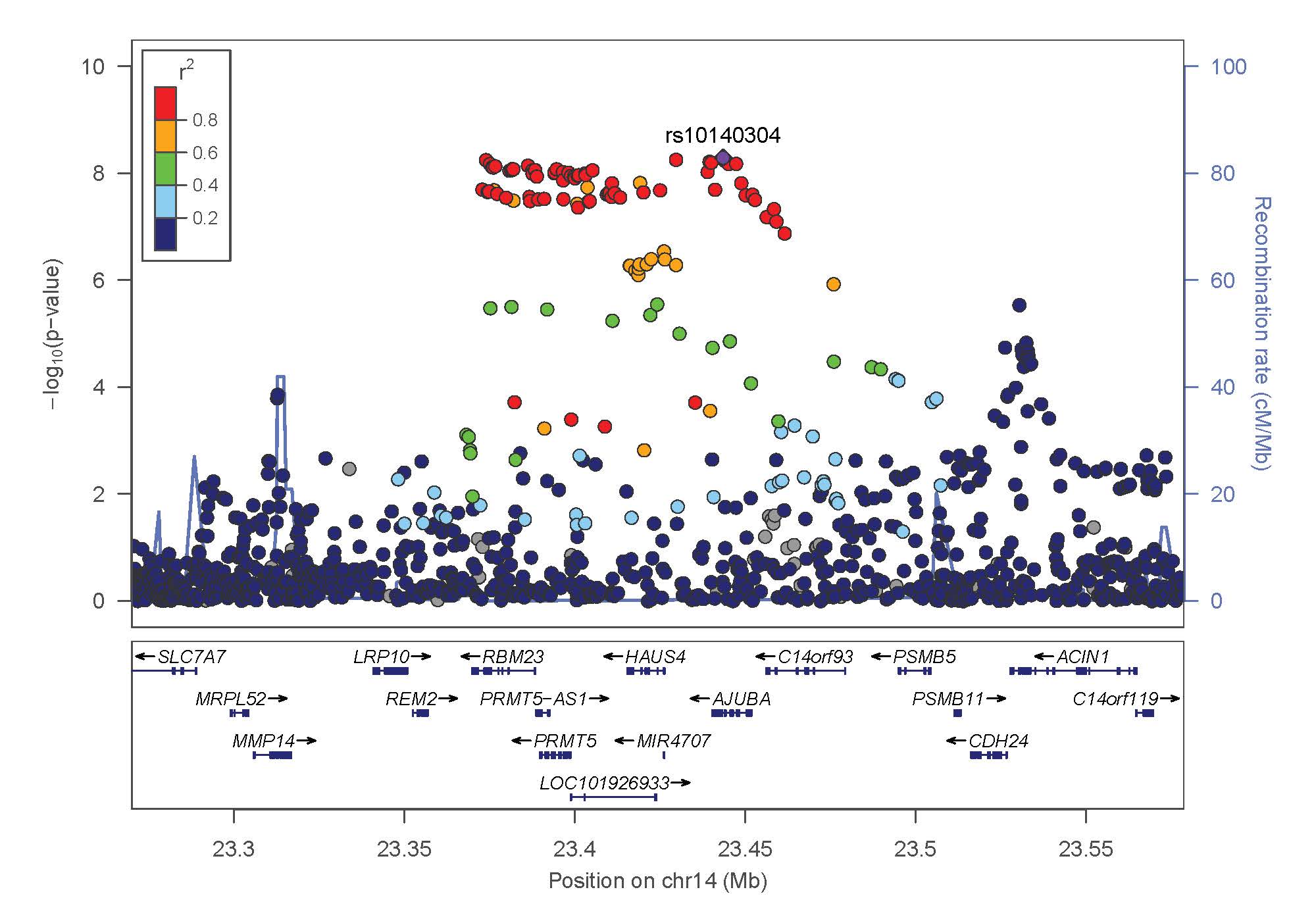

Supplement: Data S3. Regional plots of the identified genetic loci for human head size (±100 kb), related to Figure 1A and 1B [file mmc19.zip › Data S2/rs10140304.jpg]

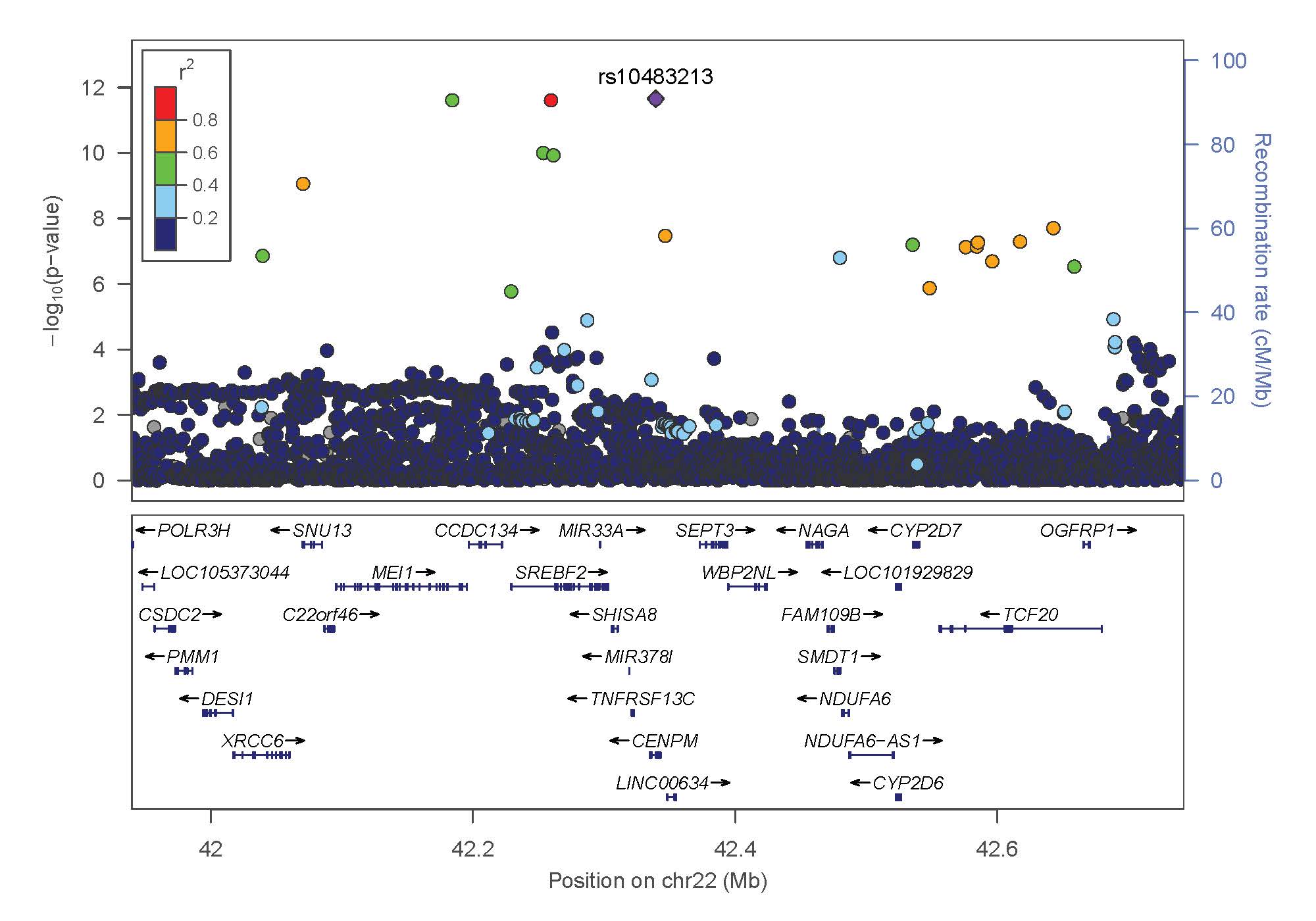

Supplement: Data S3. Regional plots of the identified genetic loci for human head size (±100 kb), related to Figure 1A and 1B [file mmc19.zip › Data S2/rs10483213.jpg]

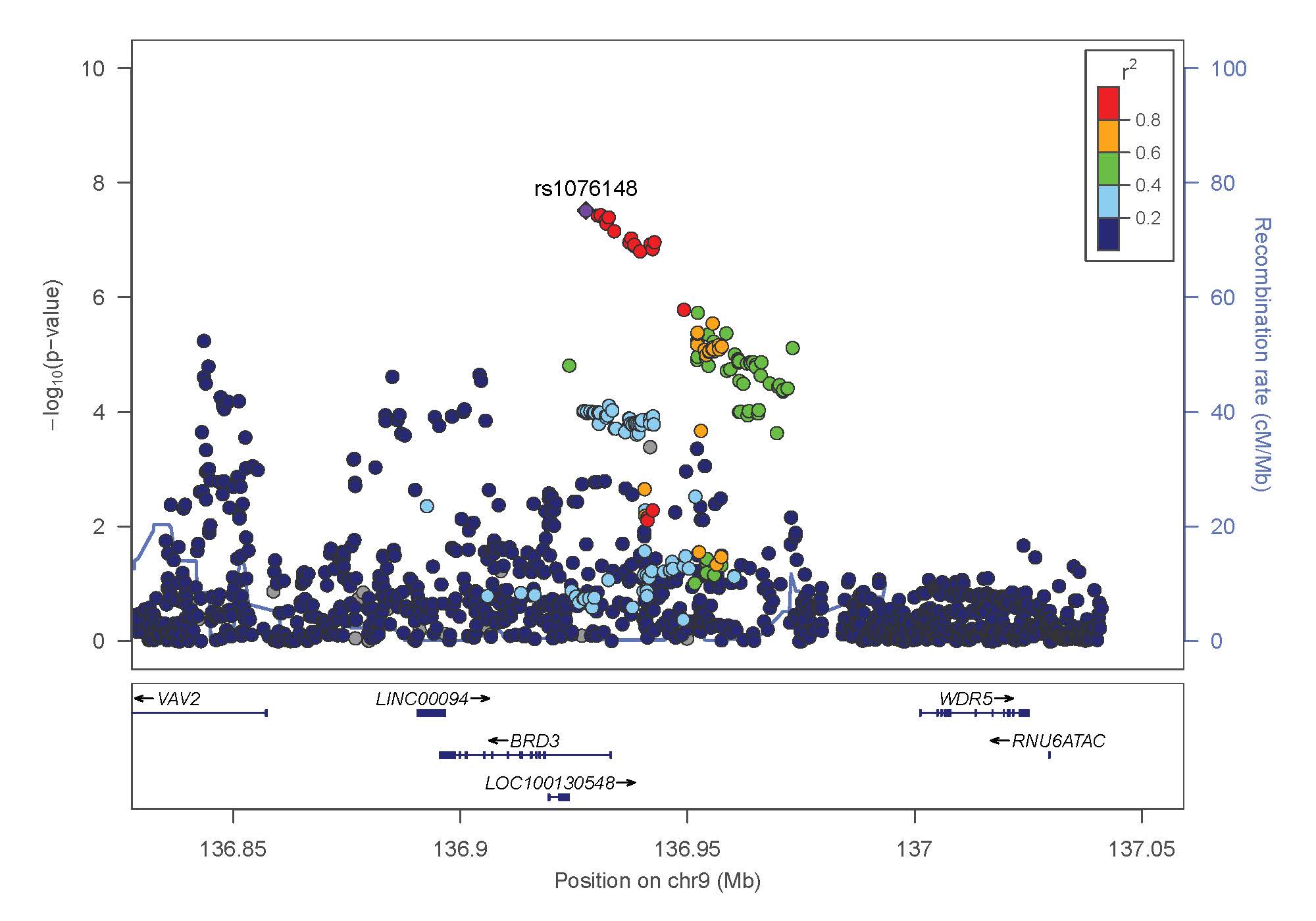

Supplement: Data S3. Regional plots of the identified genetic loci for human head size (±100 kb), related to Figure 1A and 1B [file mmc19.zip › Data S2/rs1076148.jpg]

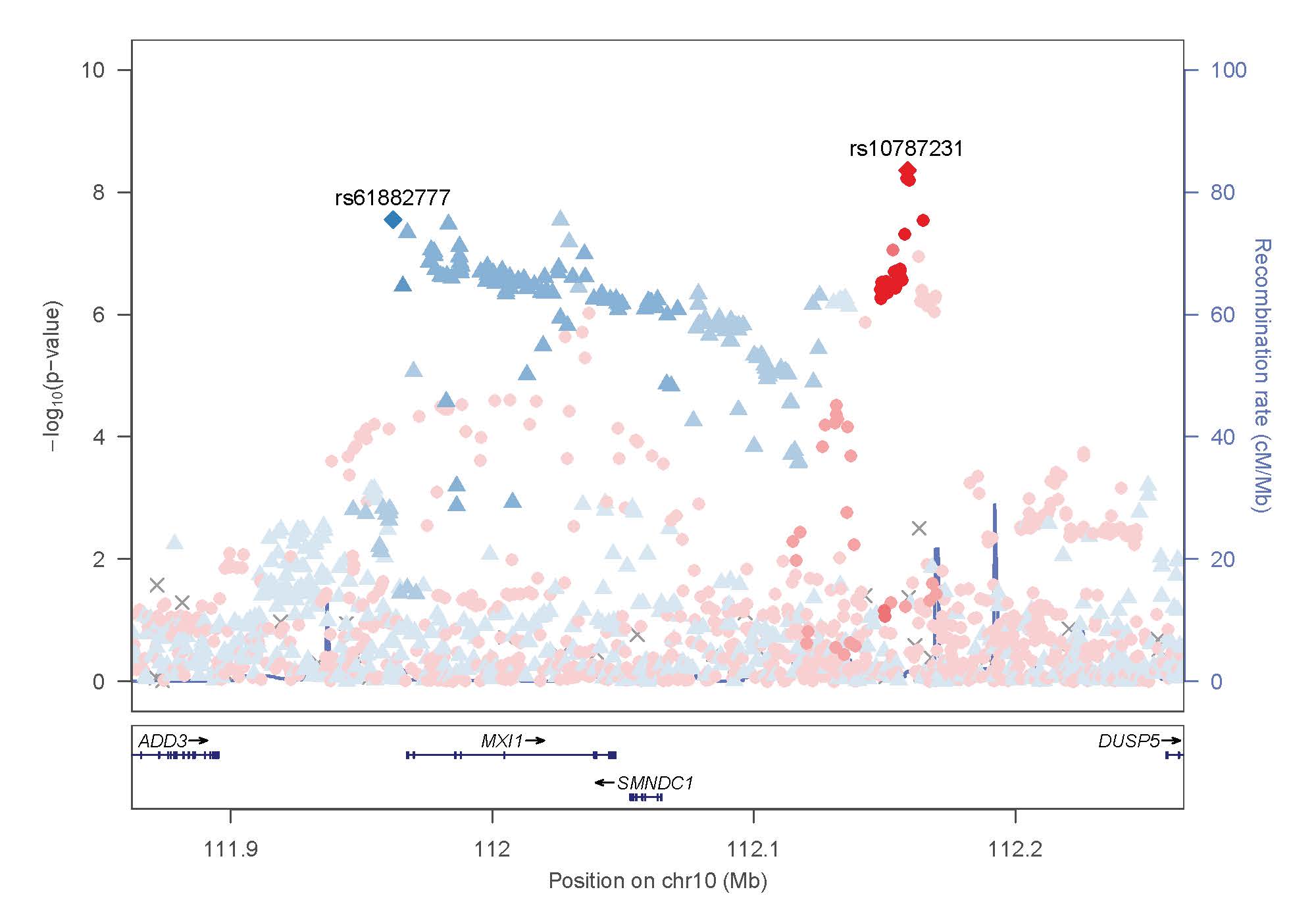

Supplement: Data S3. Regional plots of the identified genetic loci for human head size (±100 kb), related to Figure 1A and 1B [file mmc19.zip › Data S2/rs10787231.jpg]

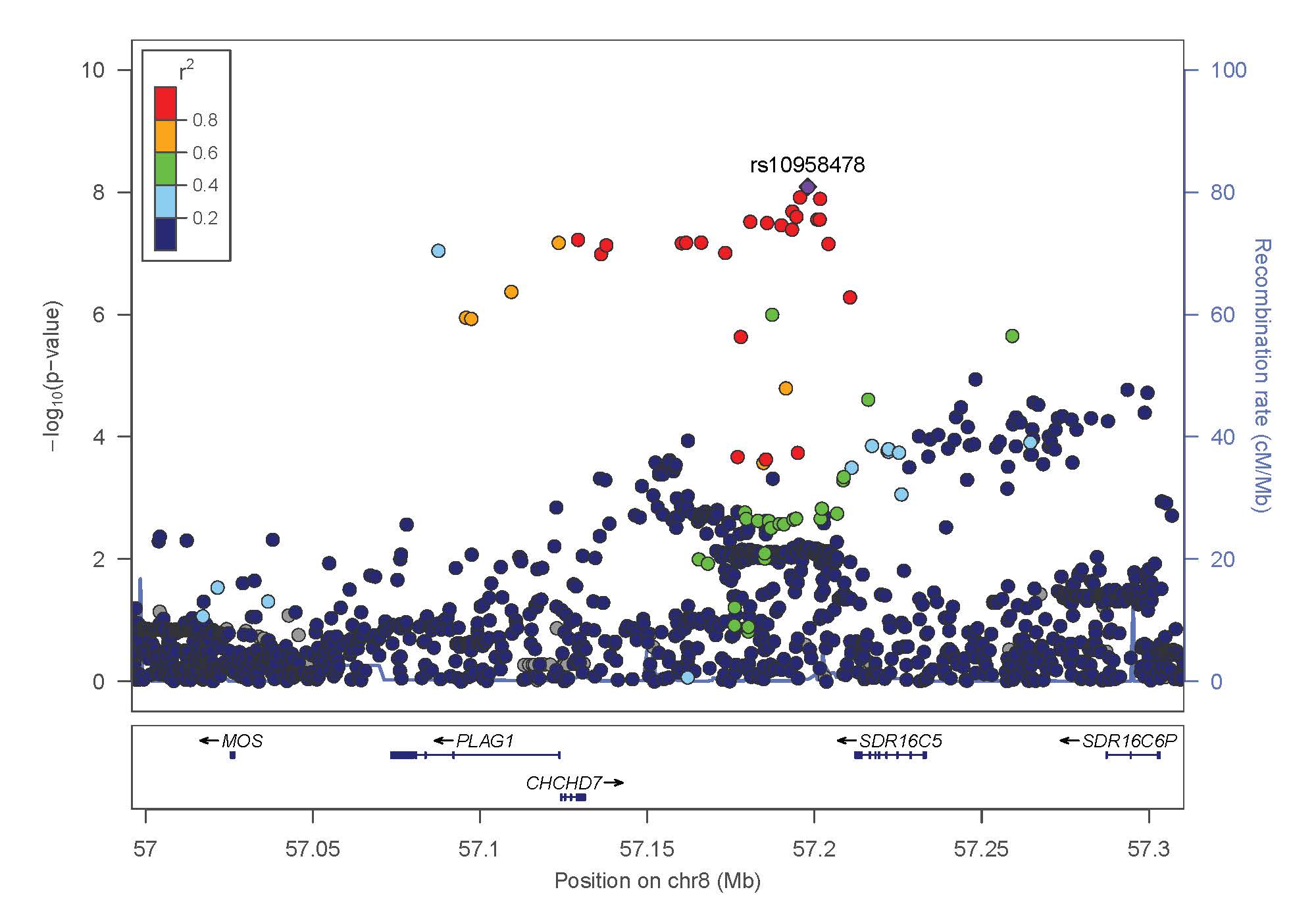

Supplement: Data S3. Regional plots of the identified genetic loci for human head size (±100 kb), related to Figure 1A and 1B [file mmc19.zip › Data S2/rs10958478.jpg]

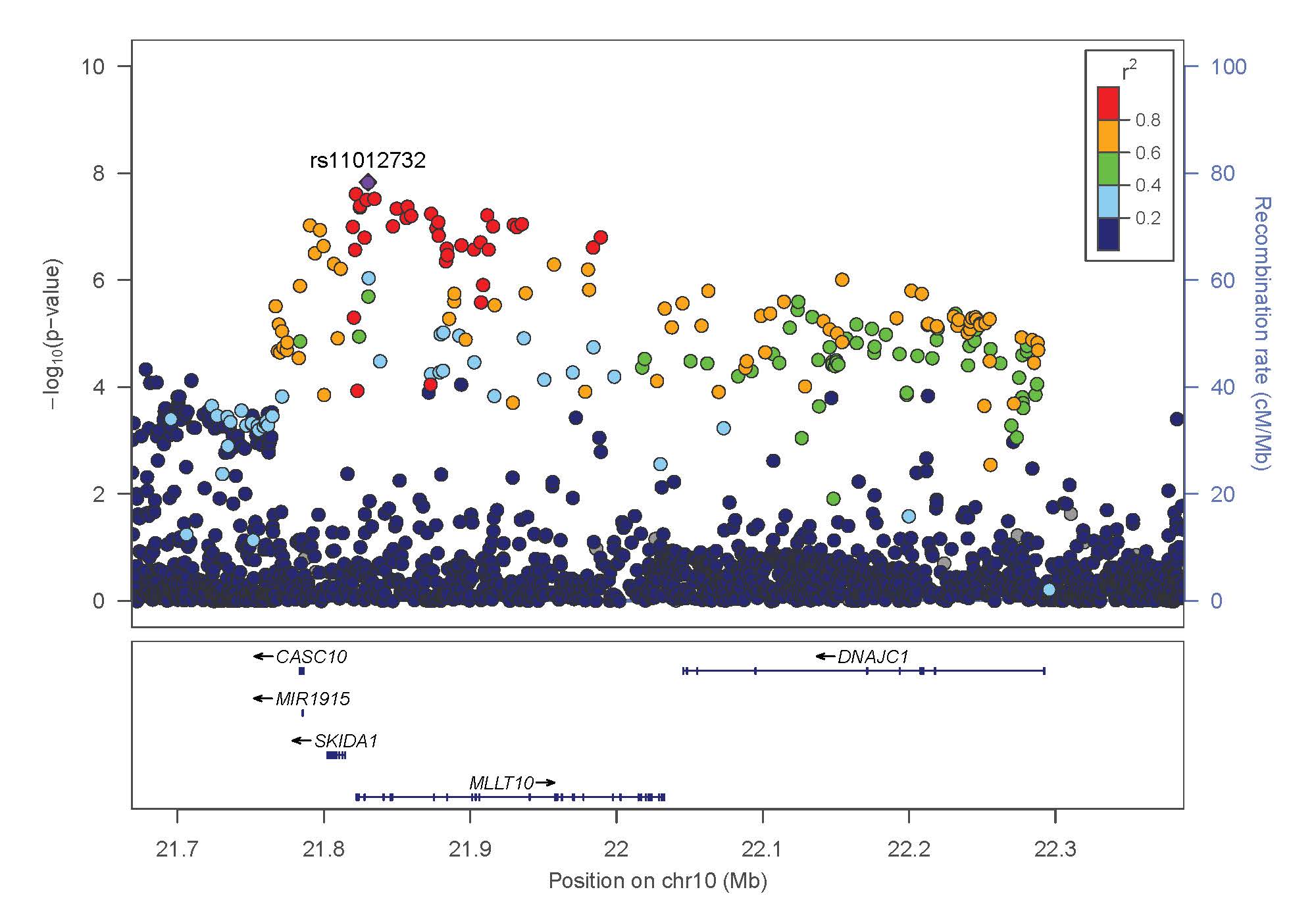

Supplement: Data S3. Regional plots of the identified genetic loci for human head size (±100 kb), related to Figure 1A and 1B [file mmc19.zip › Data S2/rs11012732.jpg]

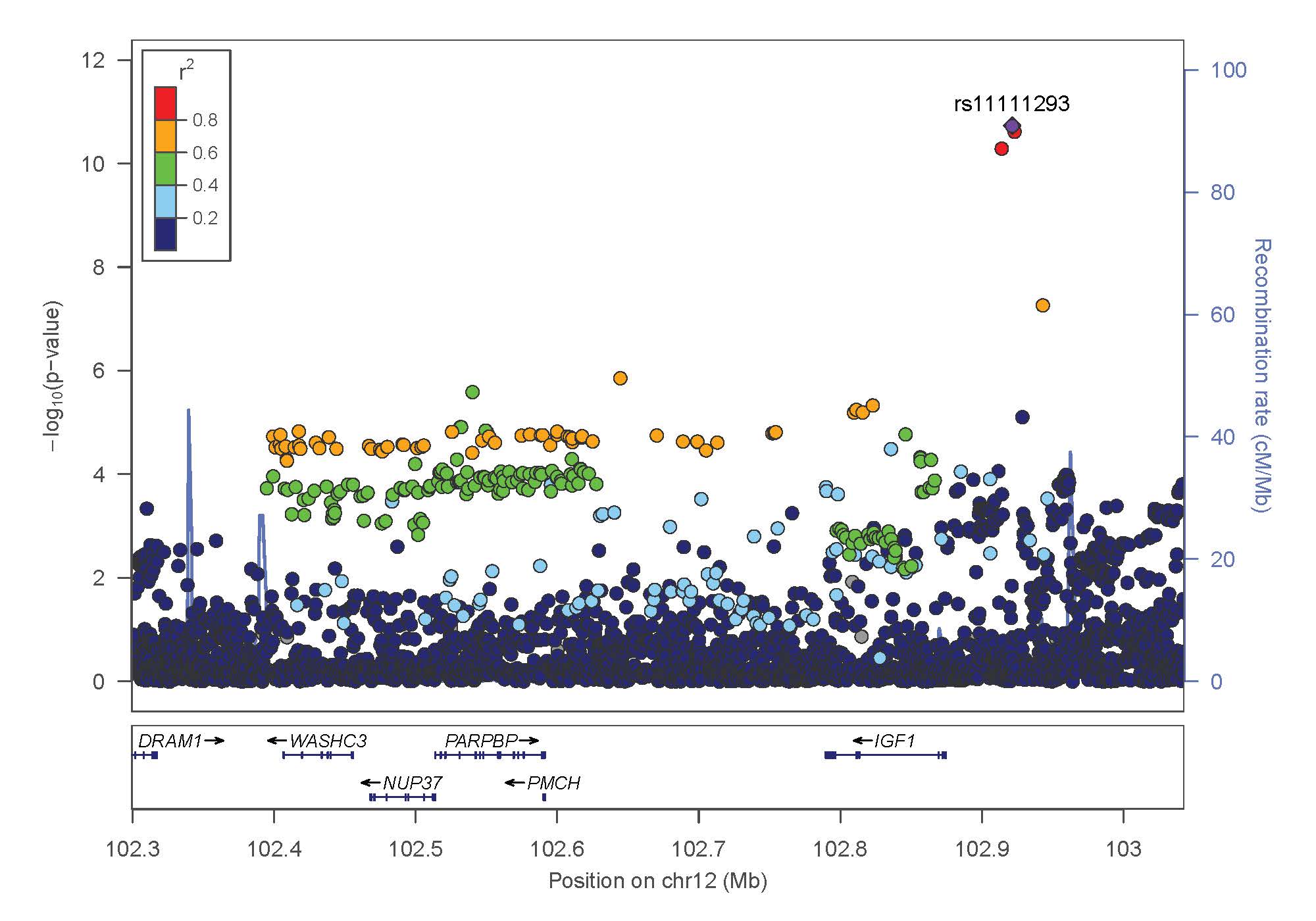

Supplement: Data S3. Regional plots of the identified genetic loci for human head size (±100 kb), related to Figure 1A and 1B [file mmc19.zip › Data S2/rs11111293.jpg]

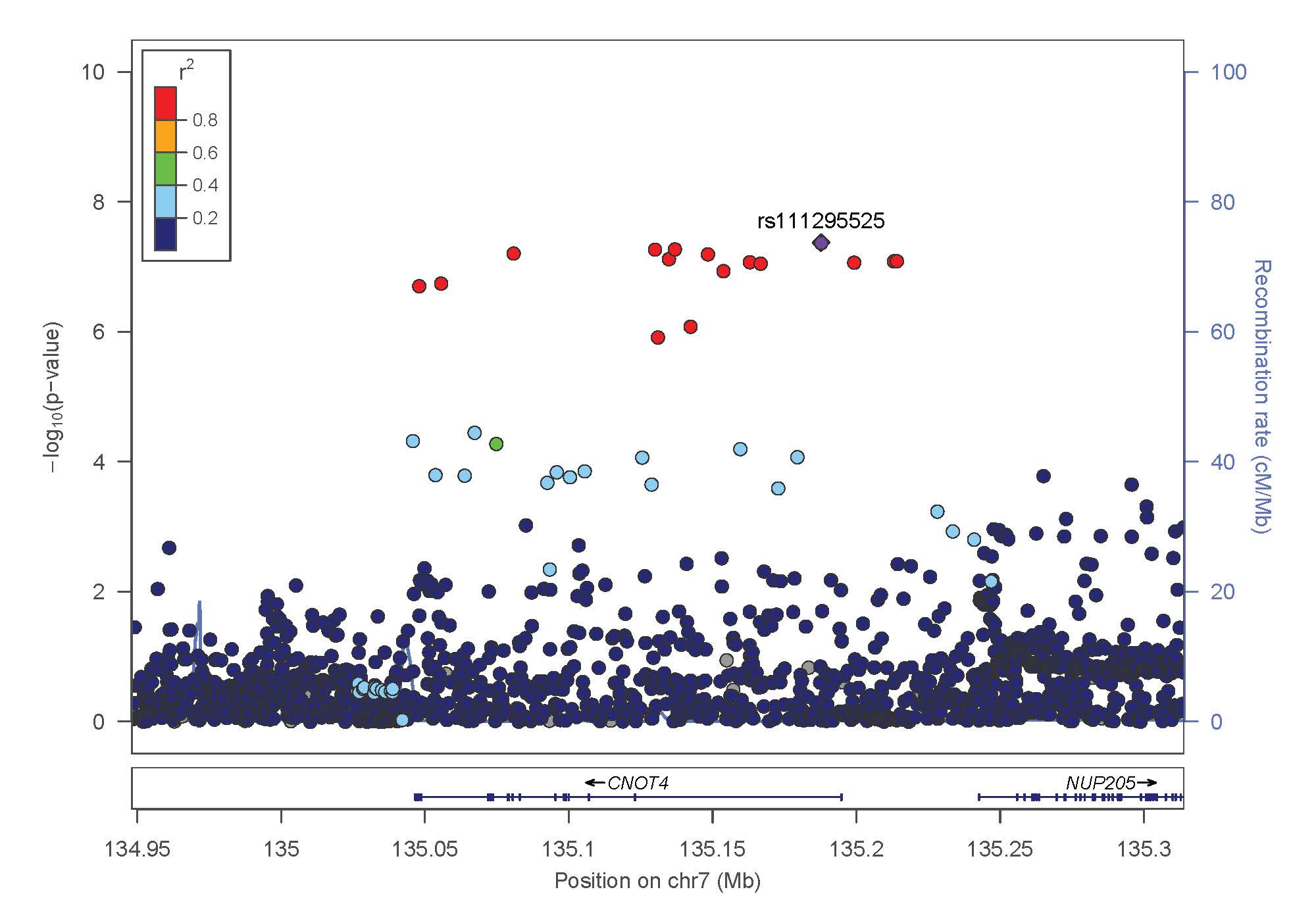

Supplement: Data S3. Regional plots of the identified genetic loci for human head size (±100 kb), related to Figure 1A and 1B [file mmc19.zip › Data S2/rs111295525.jpg]

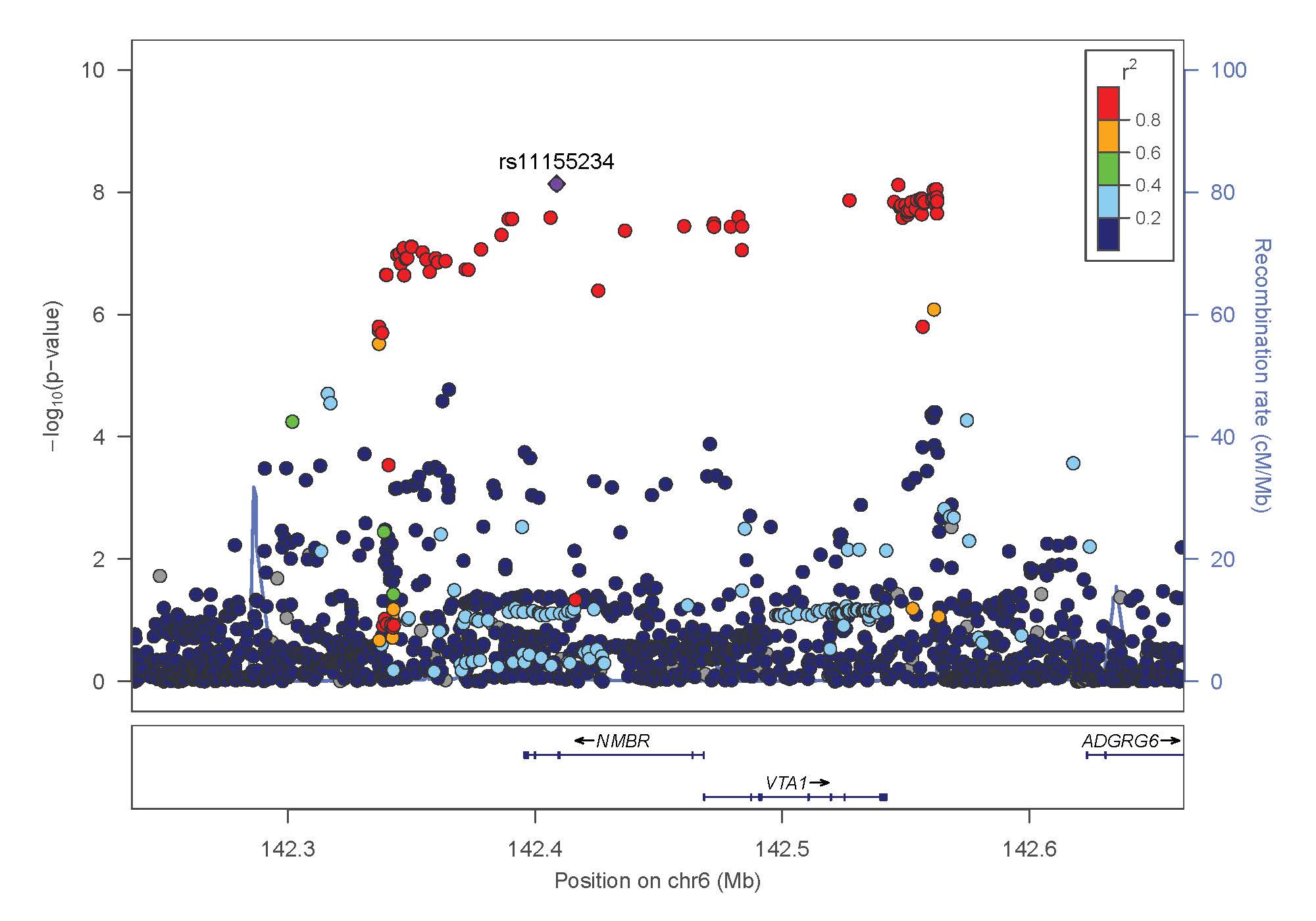

Supplement: Data S3. Regional plots of the identified genetic loci for human head size (±100 kb), related to Figure 1A and 1B [file mmc19.zip › Data S2/rs11155234.jpg]

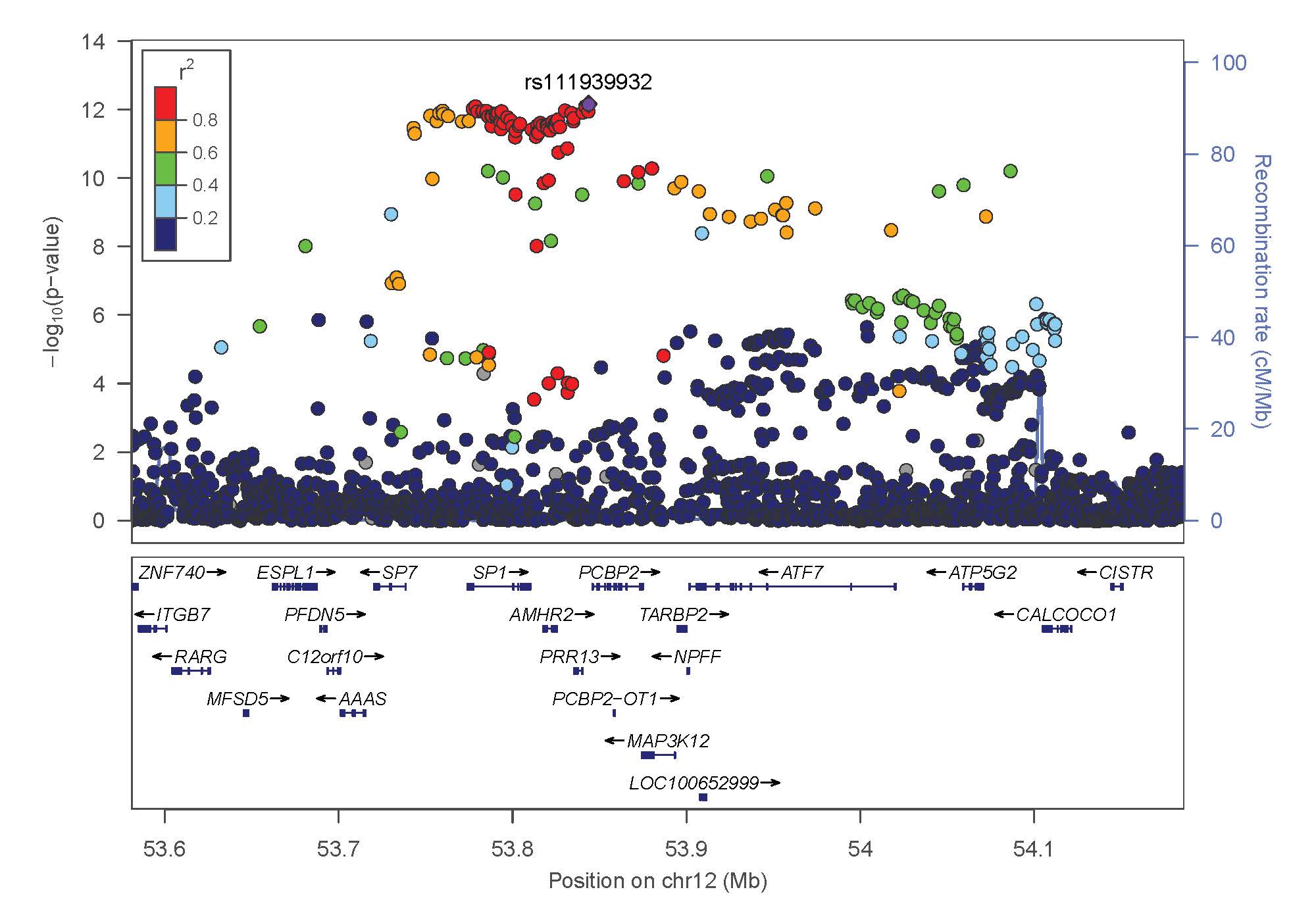

Supplement: Data S3. Regional plots of the identified genetic loci for human head size (±100 kb), related to Figure 1A and 1B [file mmc19.zip › Data S2/rs111939932.jpg]

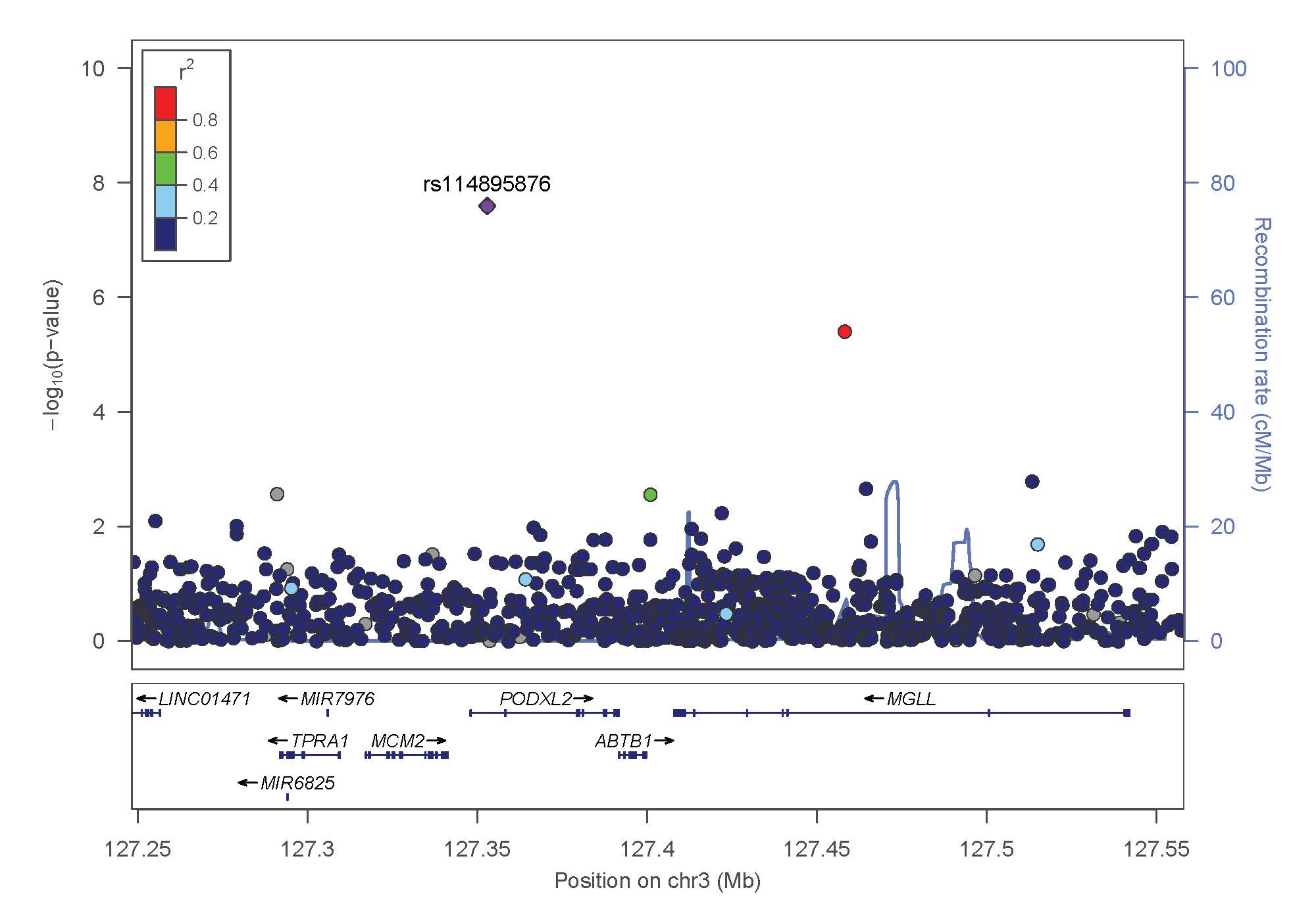

Supplement: Data S3. Regional plots of the identified genetic loci for human head size (±100 kb), related to Figure 1A and 1B [file mmc19.zip › Data S2/rs114895876.jpg]

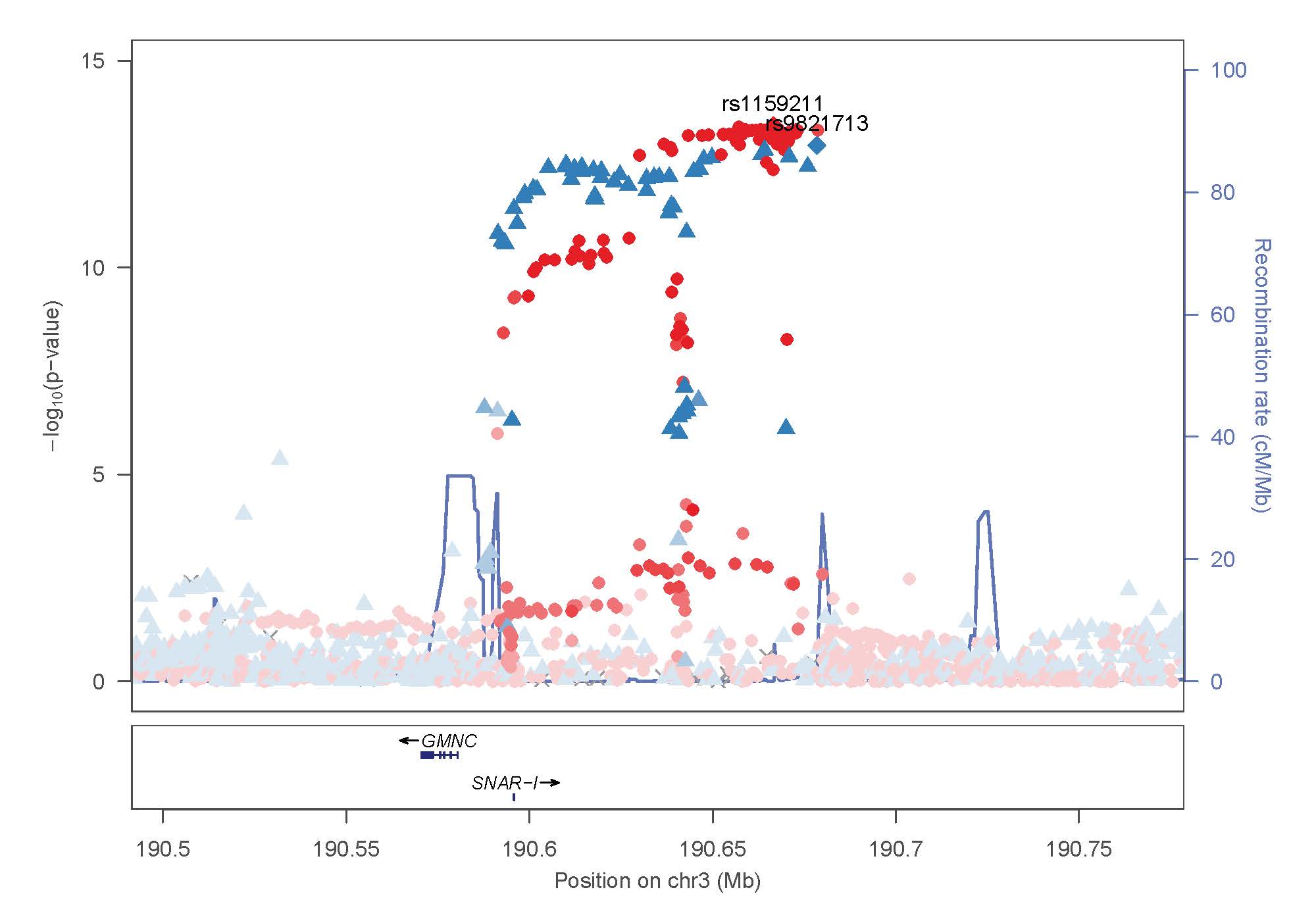

Supplement: Data S3. Regional plots of the identified genetic loci for human head size (±100 kb), related to Figure 1A and 1B [file mmc19.zip › Data S2/rs1159211.jpg]

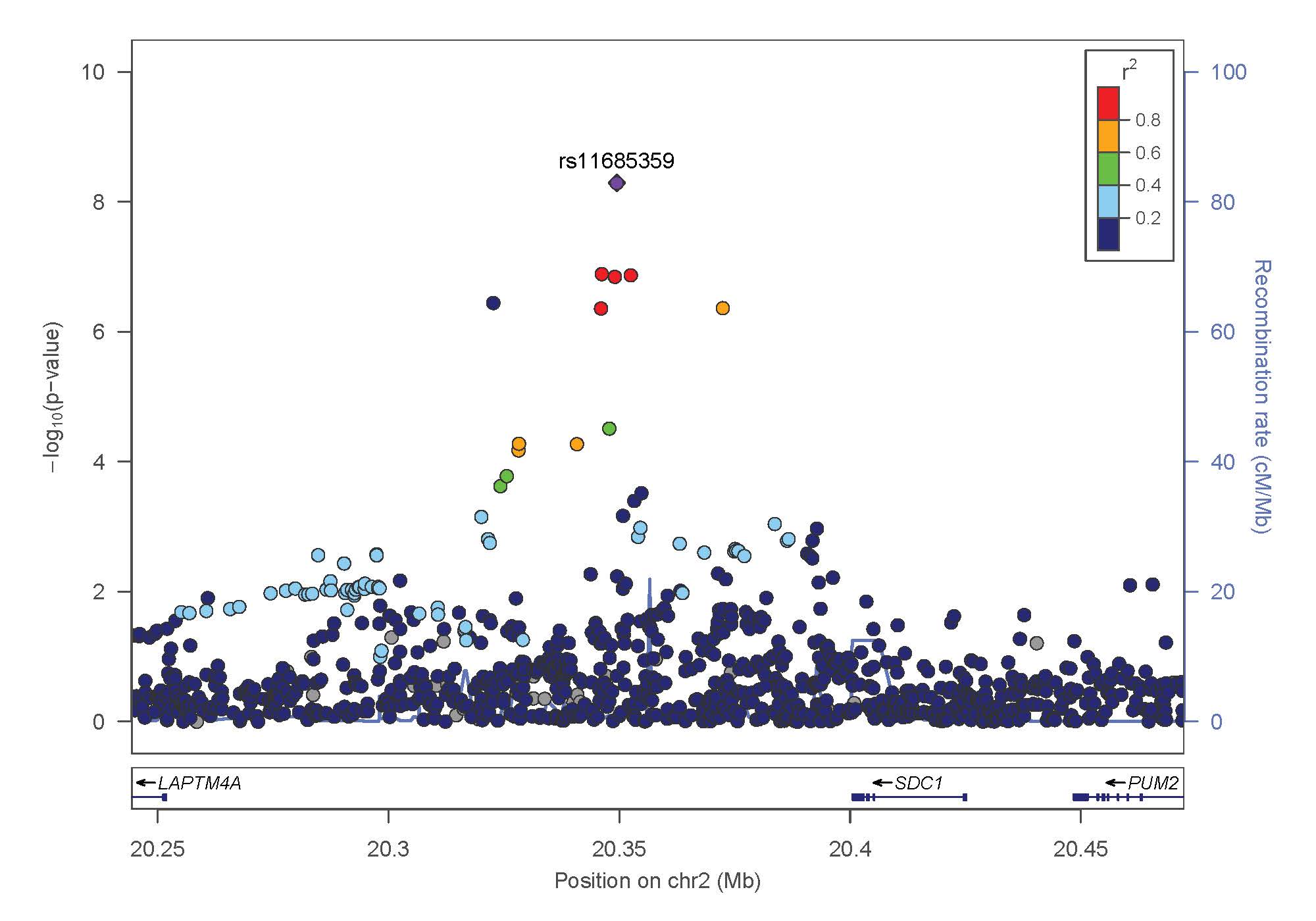

Supplement: Data S3. Regional plots of the identified genetic loci for human head size (±100 kb), related to Figure 1A and 1B [file mmc19.zip › Data S2/rs11685359.jpg]

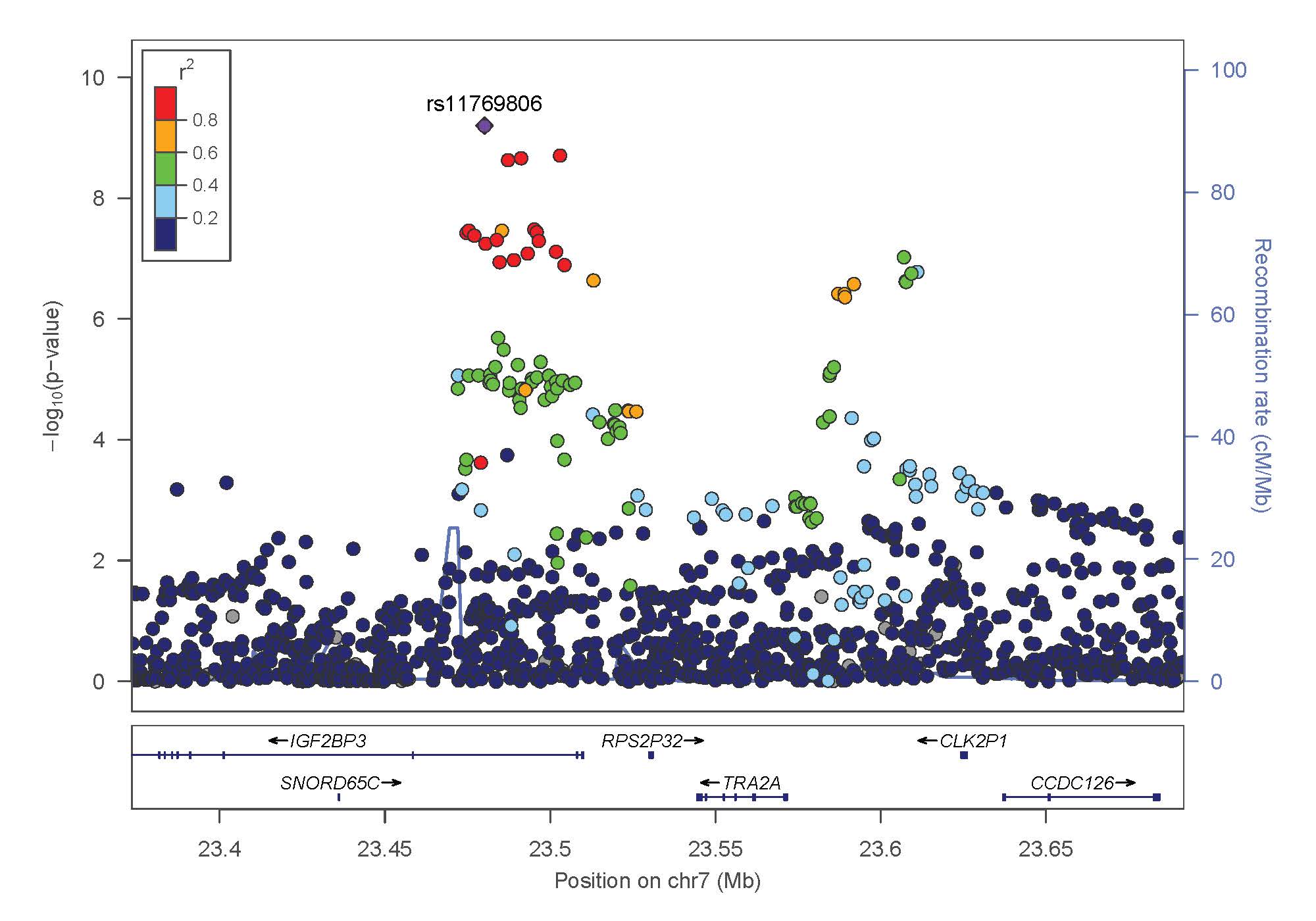

Supplement: Data S3. Regional plots of the identified genetic loci for human head size (±100 kb), related to Figure 1A and 1B [file mmc19.zip › Data S2/rs11769806.jpg]

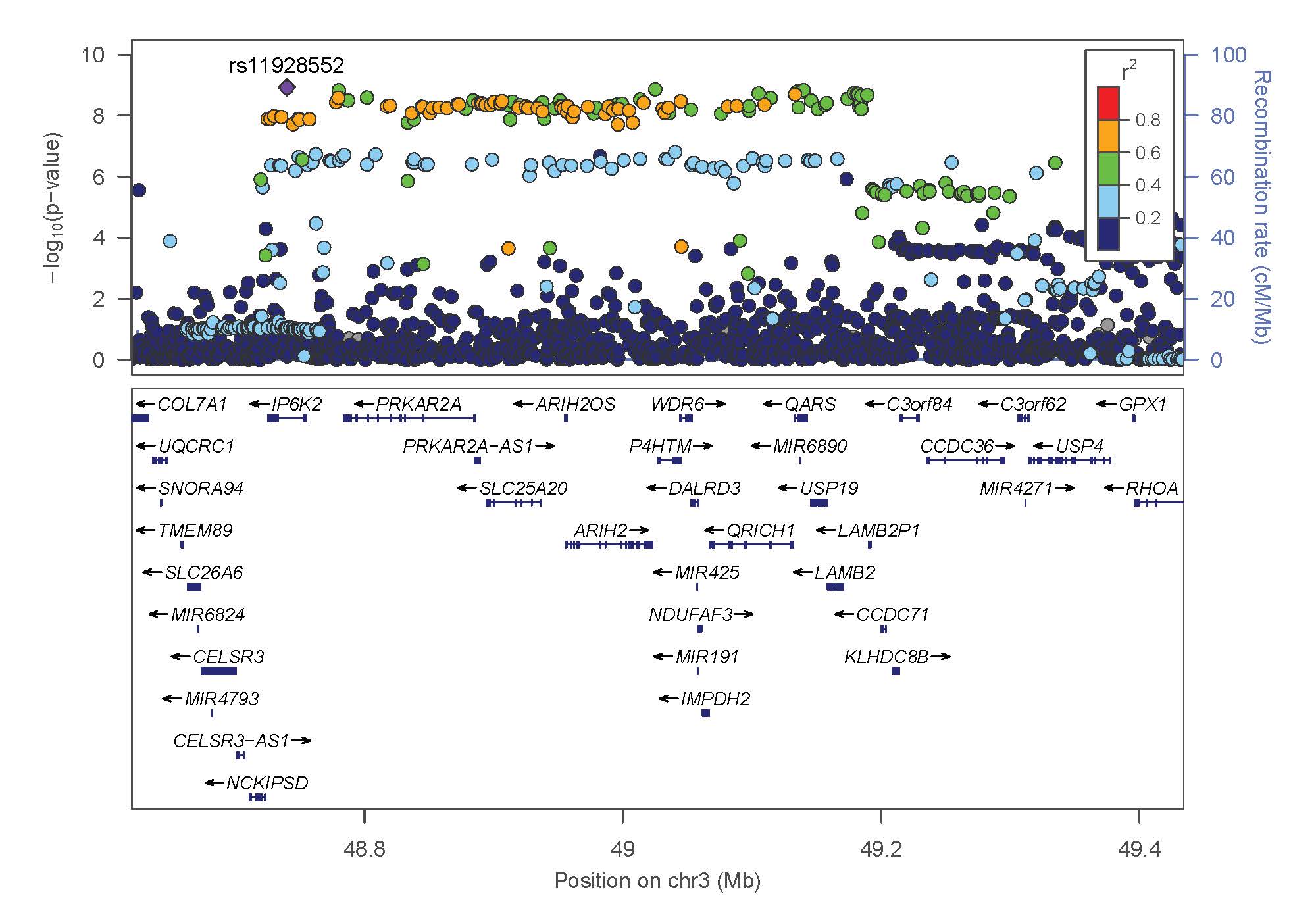

Supplement: Data S3. Regional plots of the identified genetic loci for human head size (±100 kb), related to Figure 1A and 1B [file mmc19.zip › Data S2/rs11928552.jpg]

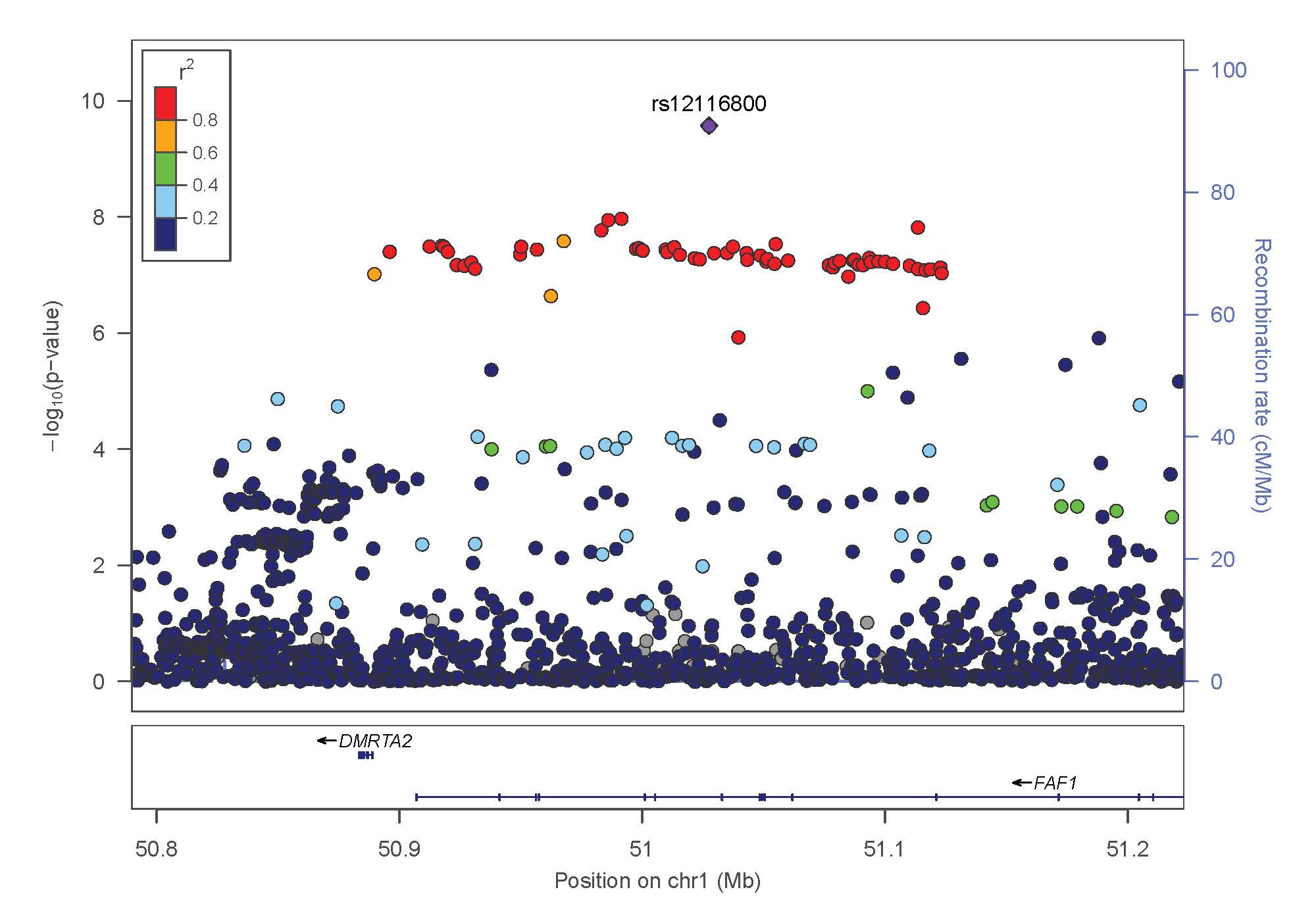

Supplement: Data S3. Regional plots of the identified genetic loci for human head size (±100 kb), related to Figure 1A and 1B [file mmc19.zip › Data S2/rs12116800.jpg]

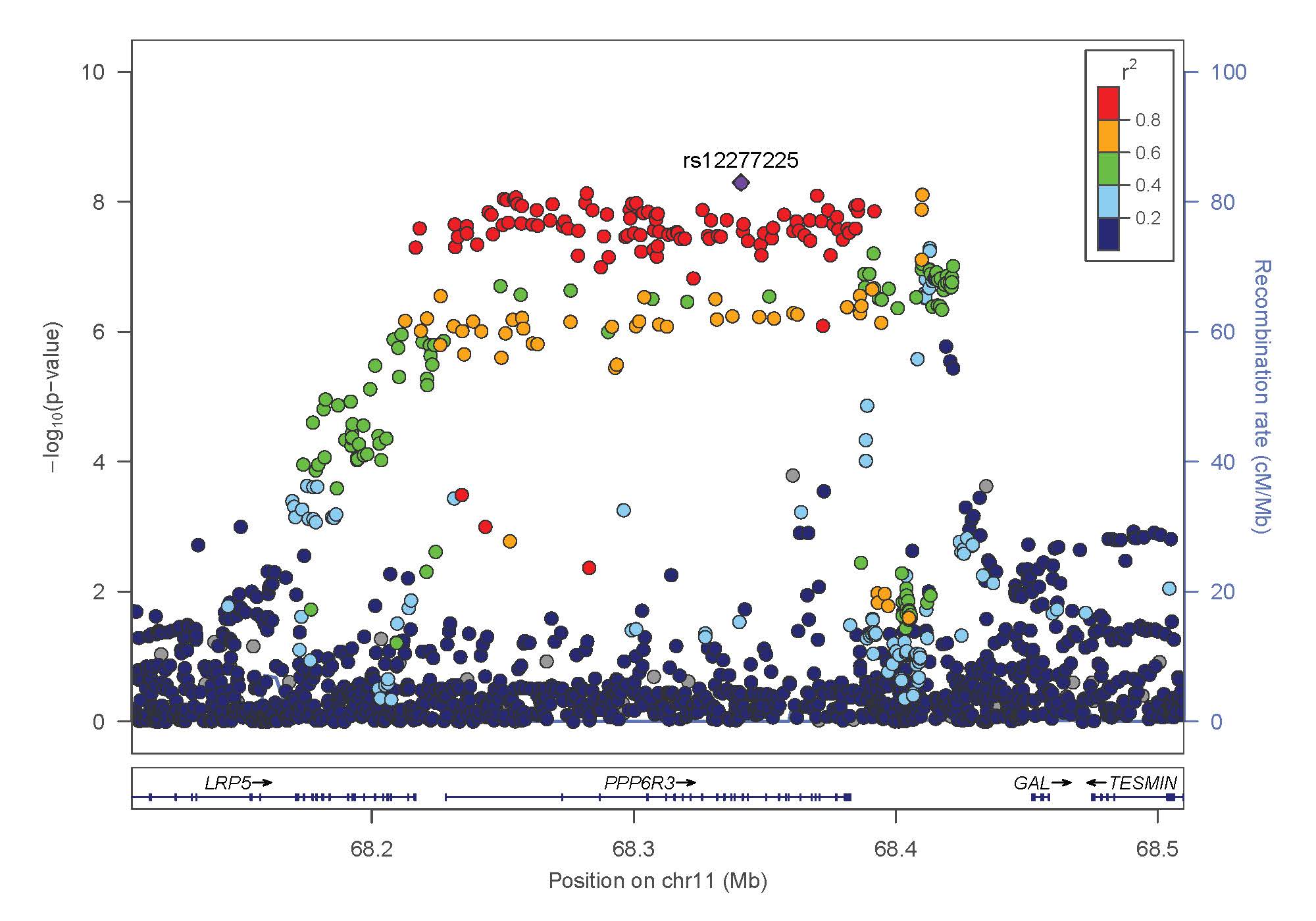

Supplement: Data S3. Regional plots of the identified genetic loci for human head size (±100 kb), related to Figure 1A and 1B [file mmc19.zip › Data S2/rs12277225.jpg]

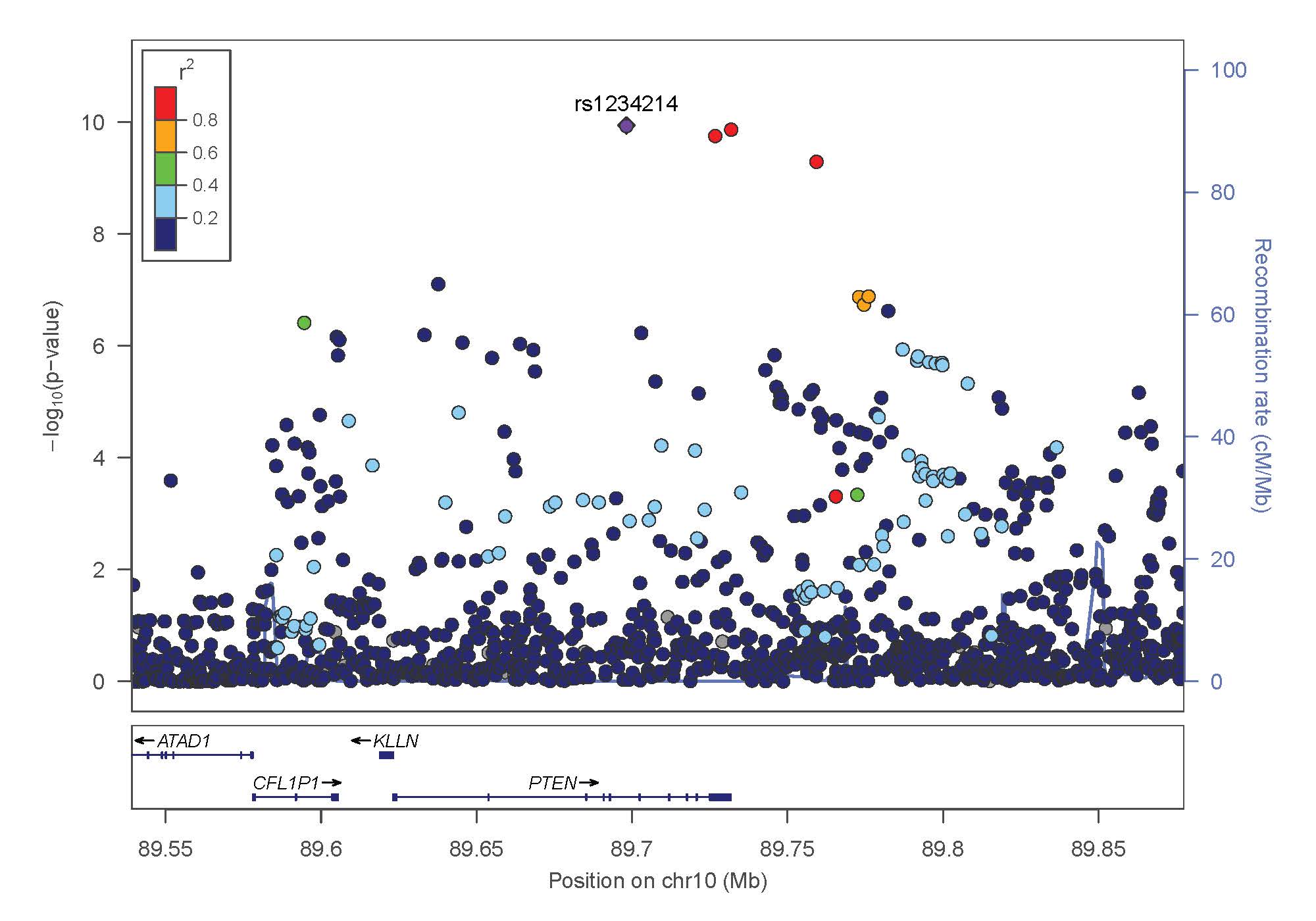

Supplement: Data S3. Regional plots of the identified genetic loci for human head size (±100 kb), related to Figure 1A and 1B [file mmc19.zip › Data S2/rs1234214.jpg]

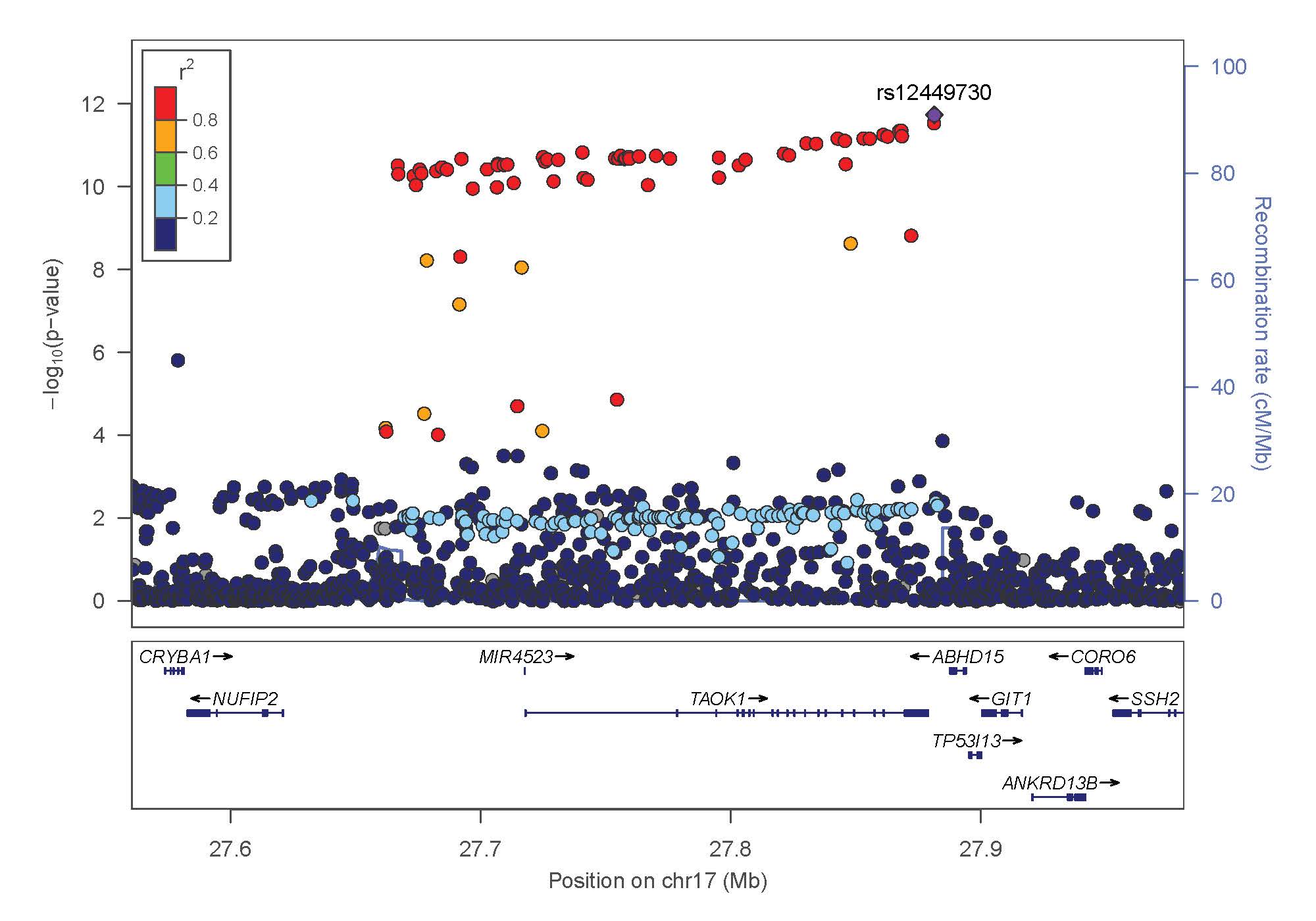

Supplement: Data S3. Regional plots of the identified genetic loci for human head size (±100 kb), related to Figure 1A and 1B [file mmc19.zip › Data S2/rs12449730.jpg]

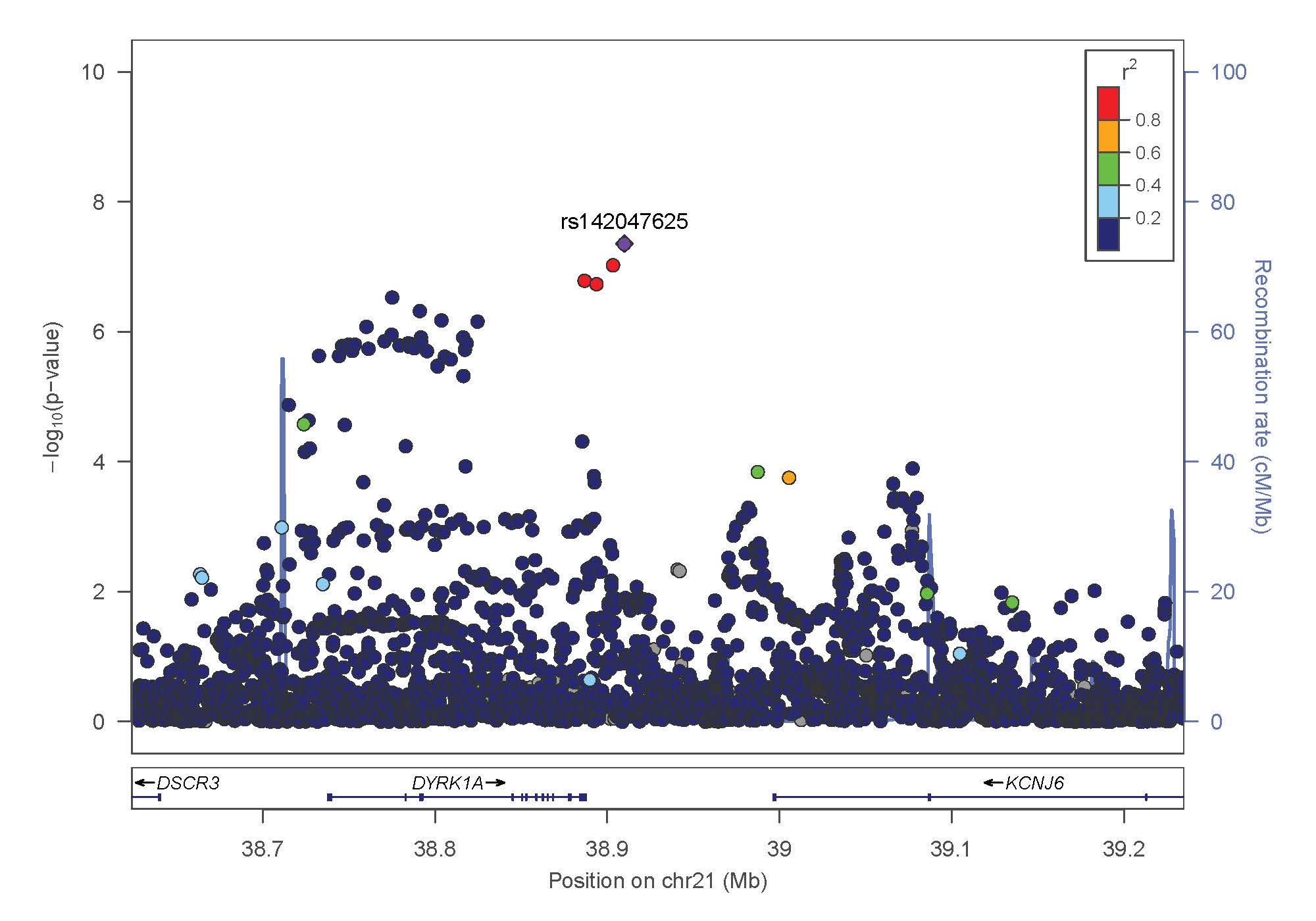

Supplement: Data S3. Regional plots of the identified genetic loci for human head size (±100 kb), related to Figure 1A and 1B [file mmc19.zip › Data S2/rs142047625.jpg]

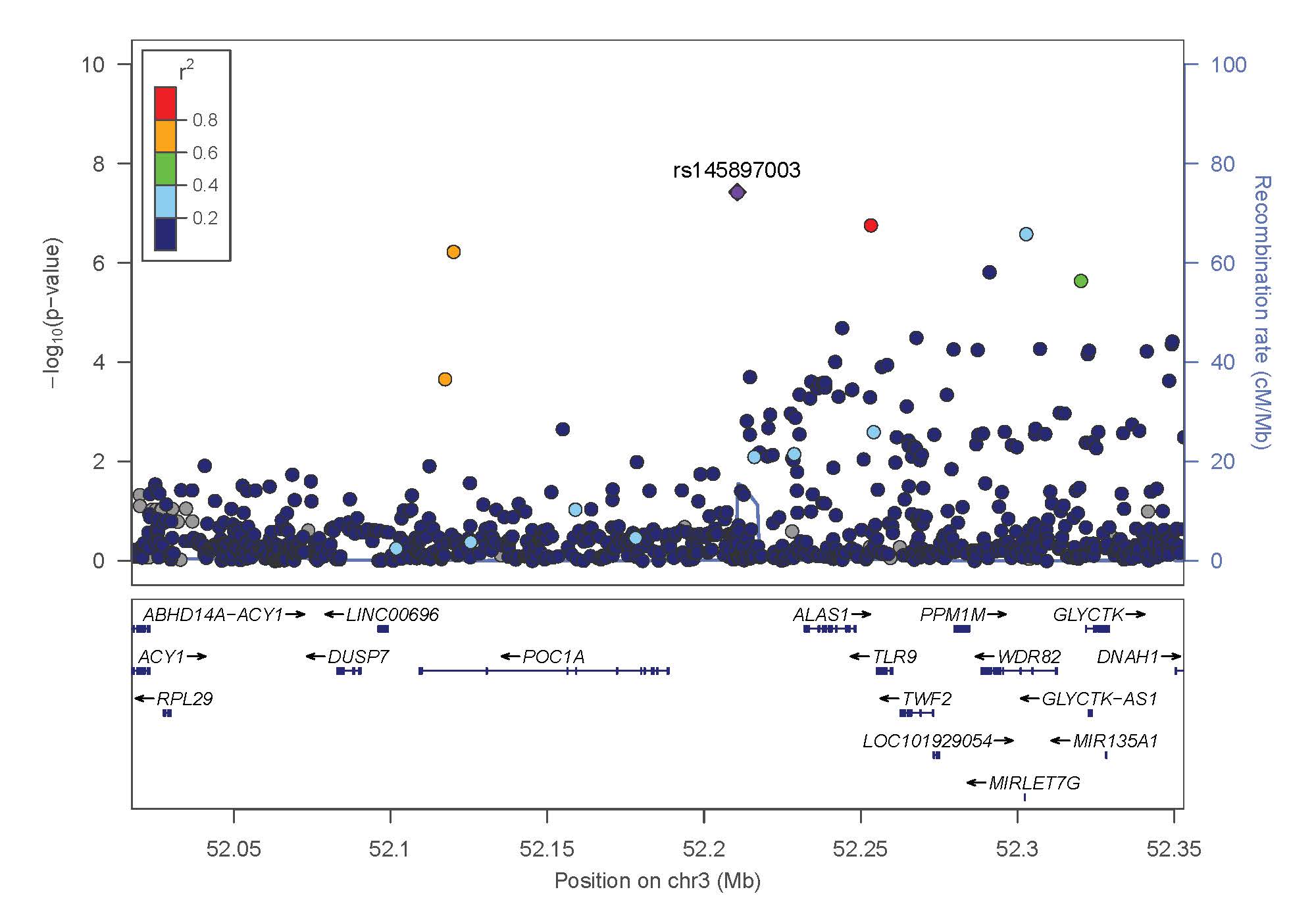

Supplement: Data S3. Regional plots of the identified genetic loci for human head size (±100 kb), related to Figure 1A and 1B [file mmc19.zip › Data S2/rs145897003.jpg]

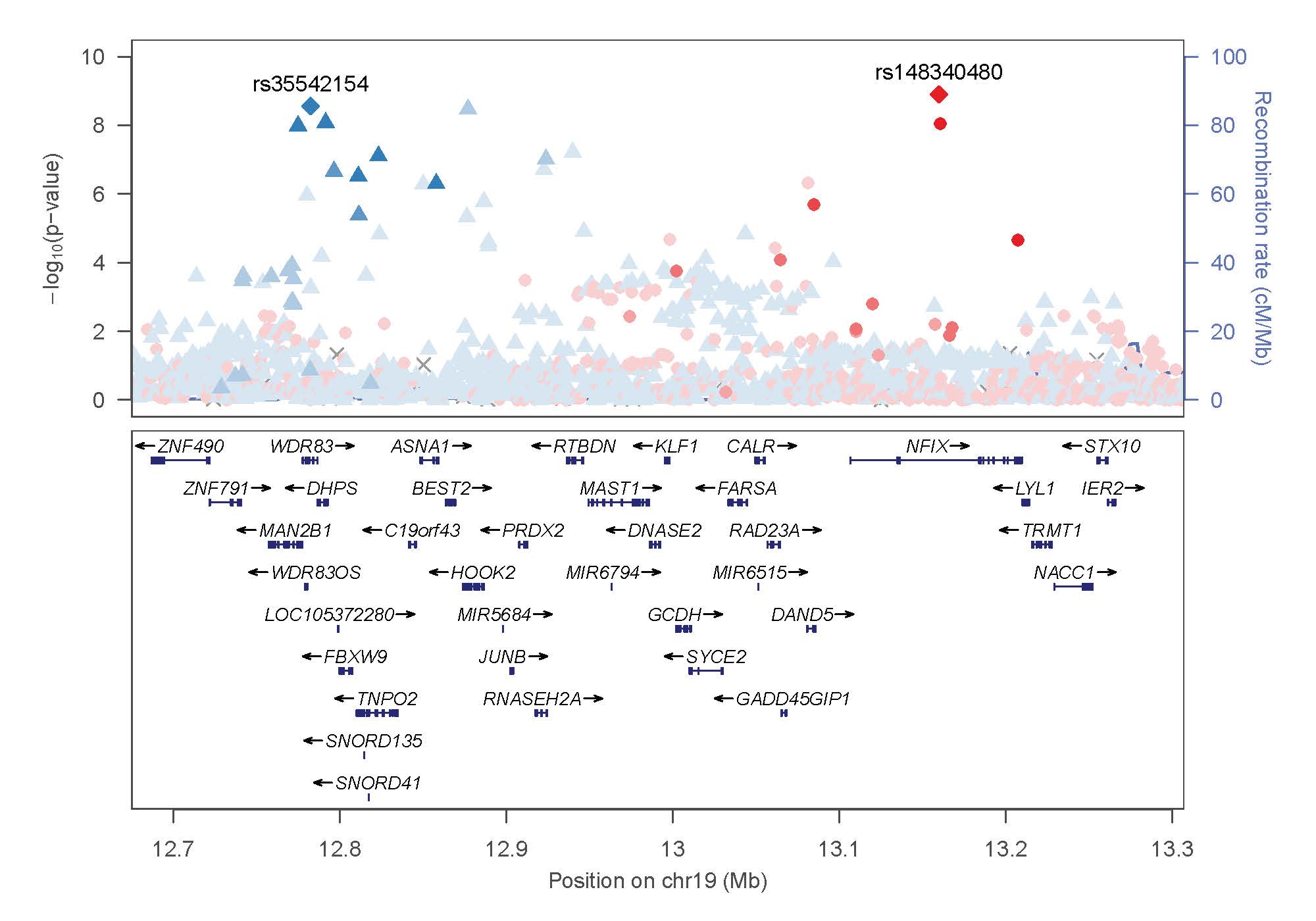

Supplement: Data S3. Regional plots of the identified genetic loci for human head size (±100 kb), related to Figure 1A and 1B [file mmc19.zip › Data S2/rs148340480.jpg]

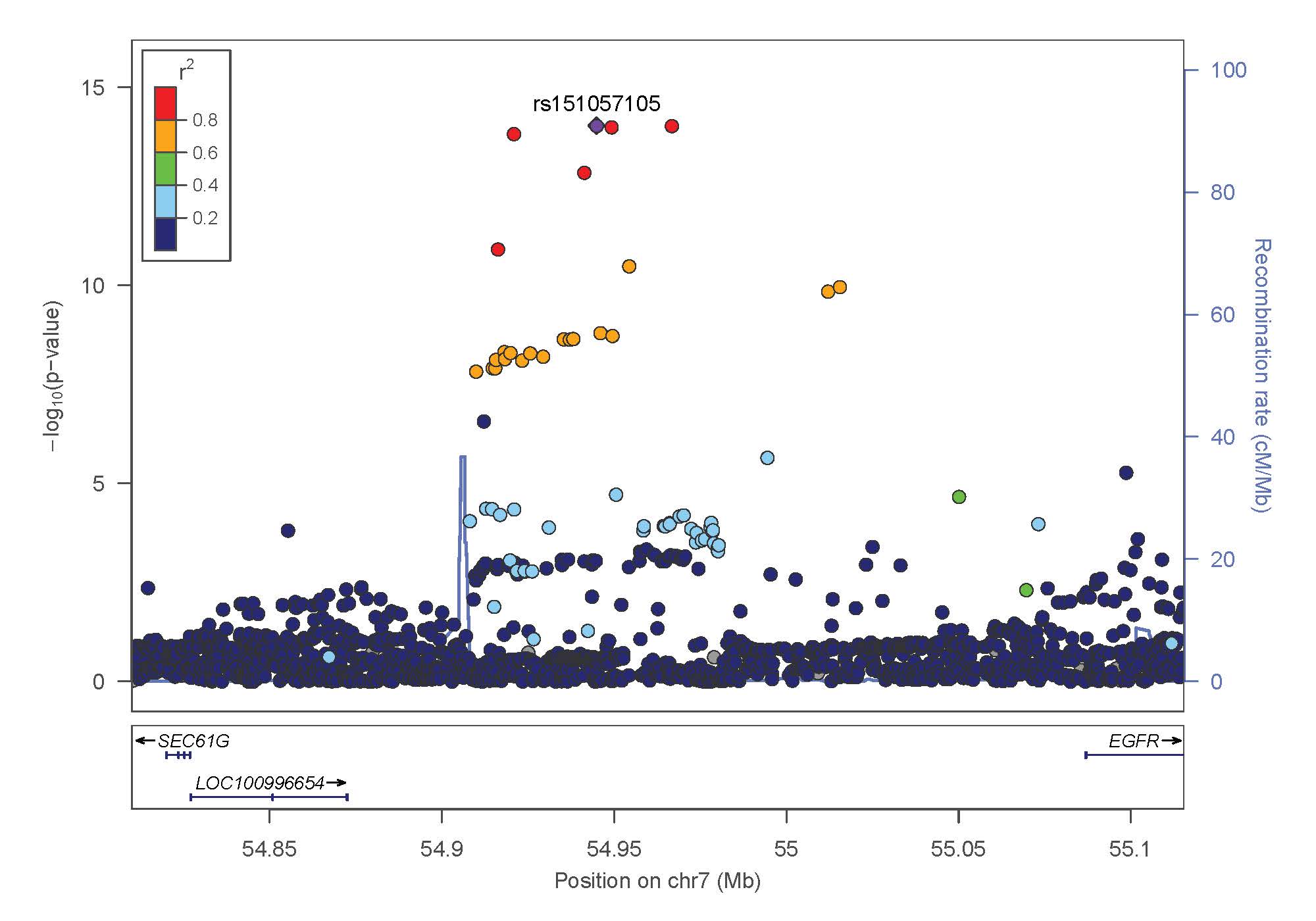

Supplement: Data S3. Regional plots of the identified genetic loci for human head size (±100 kb), related to Figure 1A and 1B [file mmc19.zip › Data S2/rs151057105.jpg]

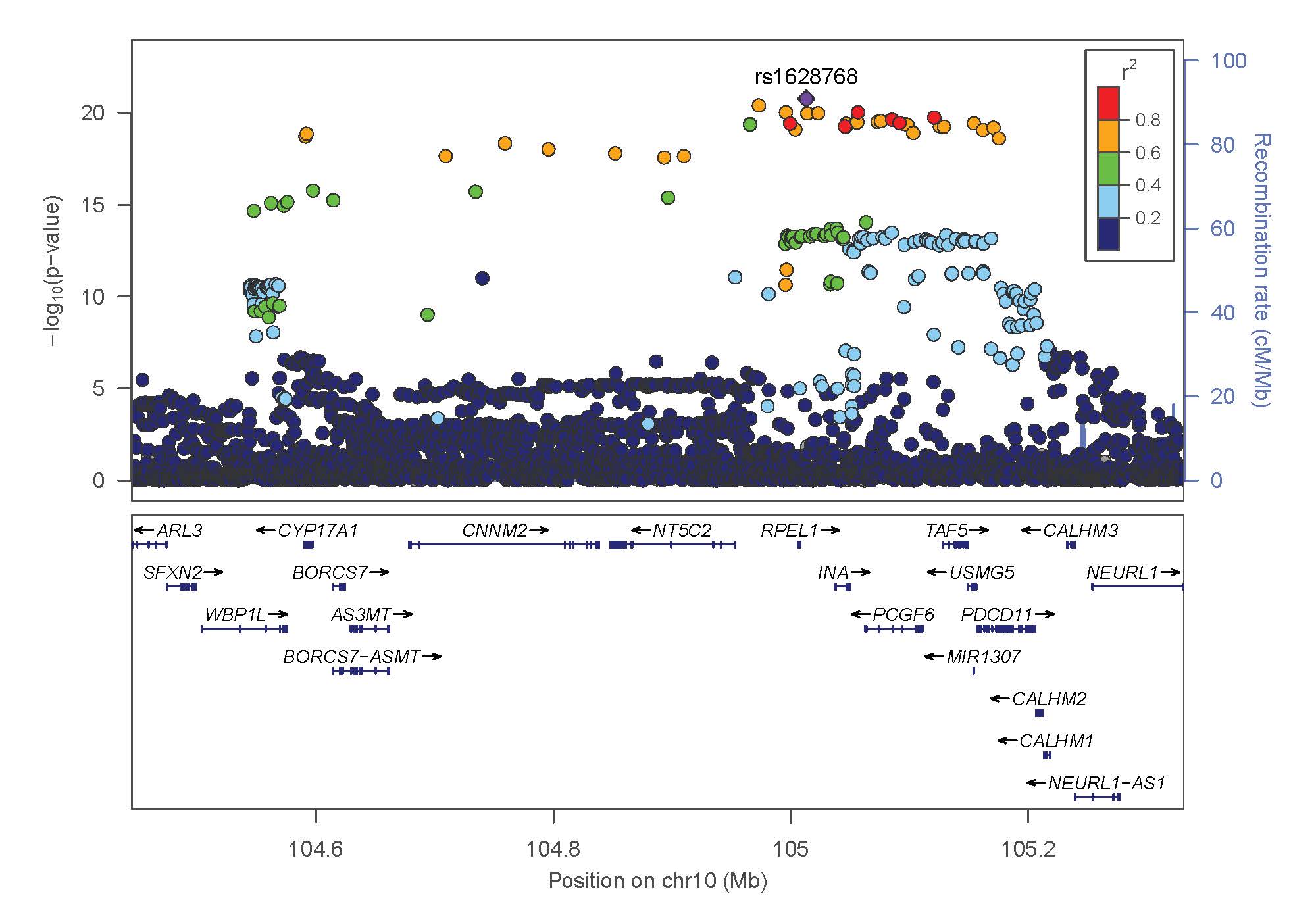

Supplement: Data S3. Regional plots of the identified genetic loci for human head size (±100 kb), related to Figure 1A and 1B [file mmc19.zip › Data S2/rs1628768.jpg]

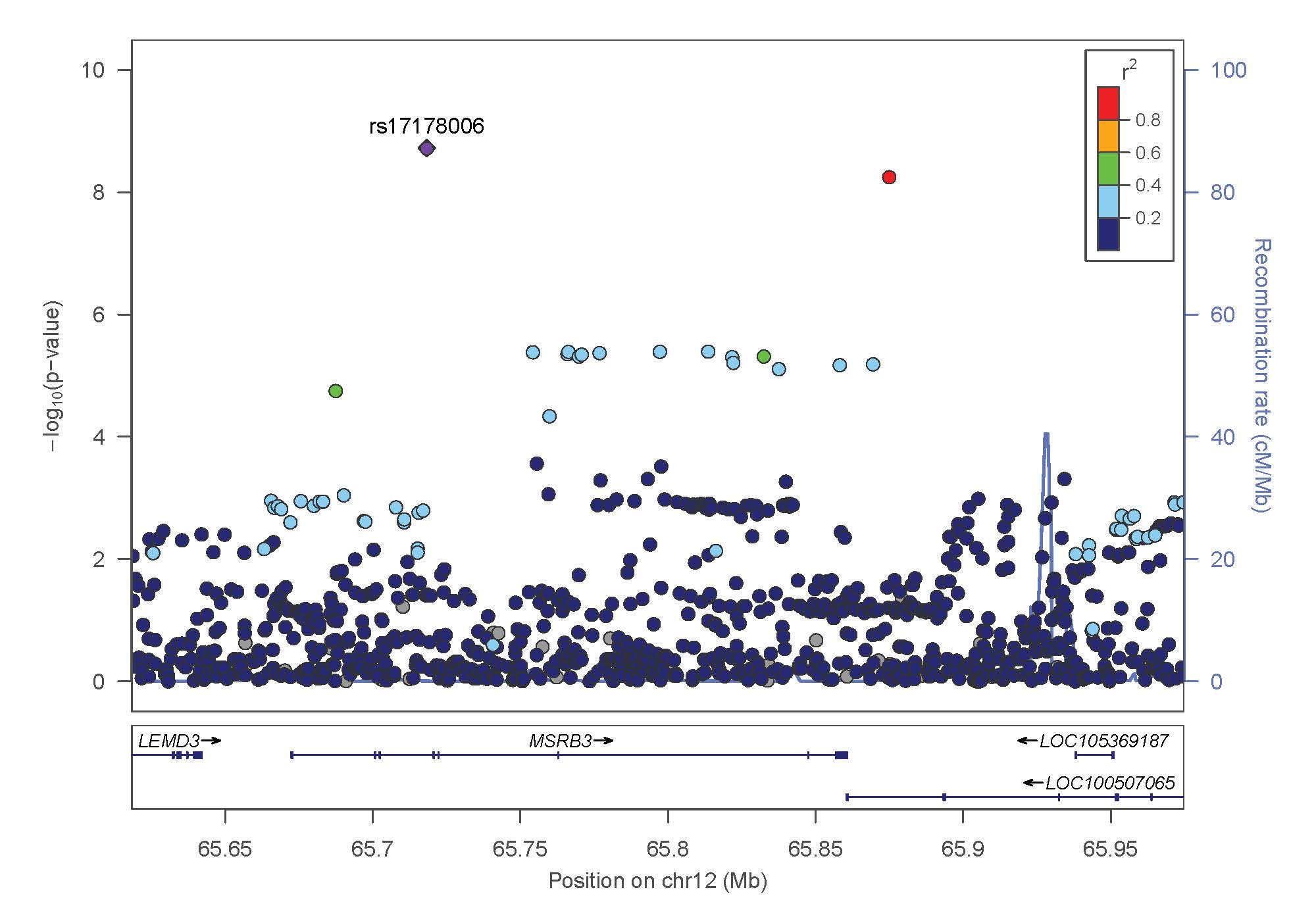

Supplement: Data S3. Regional plots of the identified genetic loci for human head size (±100 kb), related to Figure 1A and 1B [file mmc19.zip › Data S2/rs17178006.jpg]

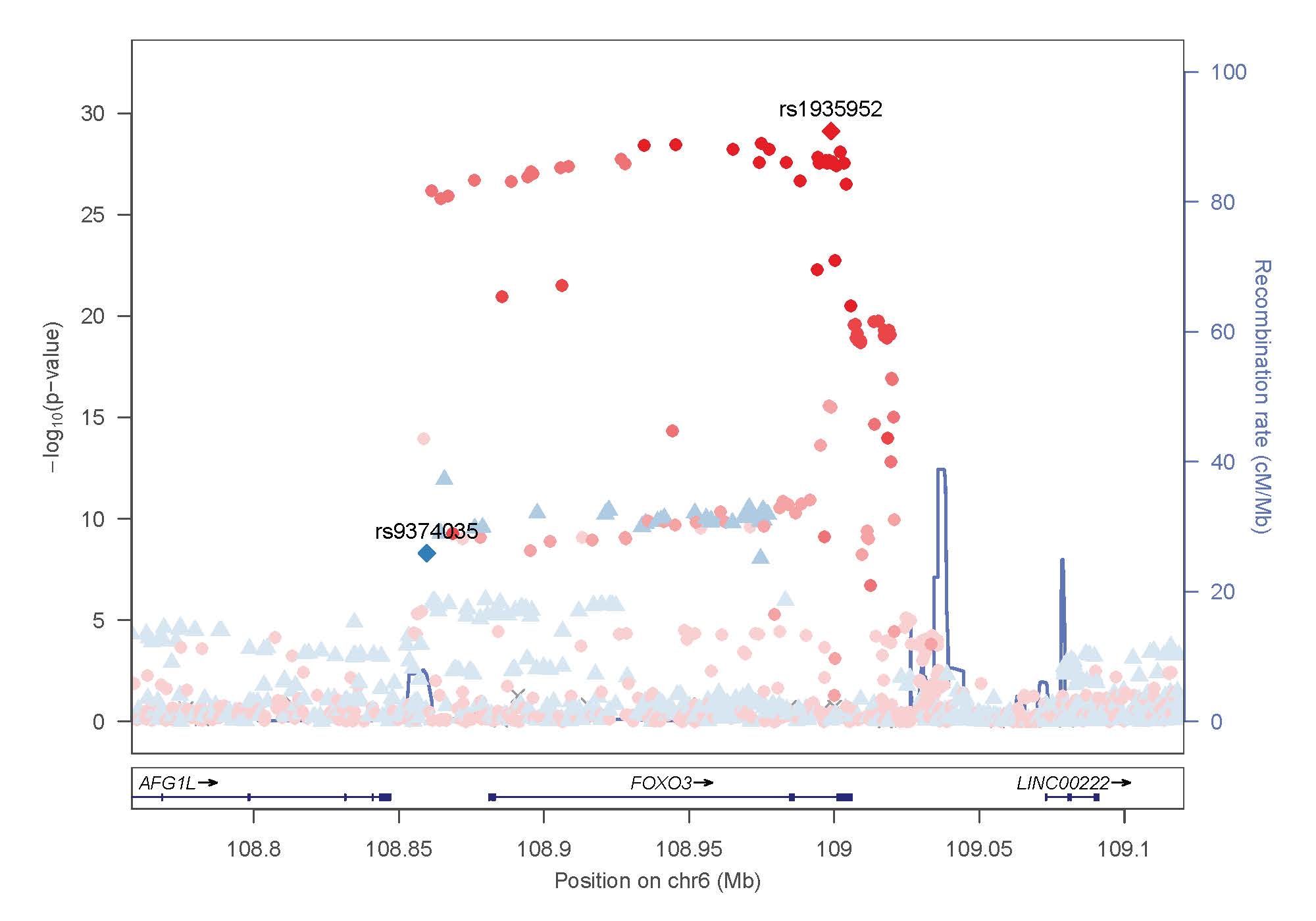

Supplement: Data S3. Regional plots of the identified genetic loci for human head size (±100 kb), related to Figure 1A and 1B [file mmc19.zip › Data S2/rs1935952.jpg]

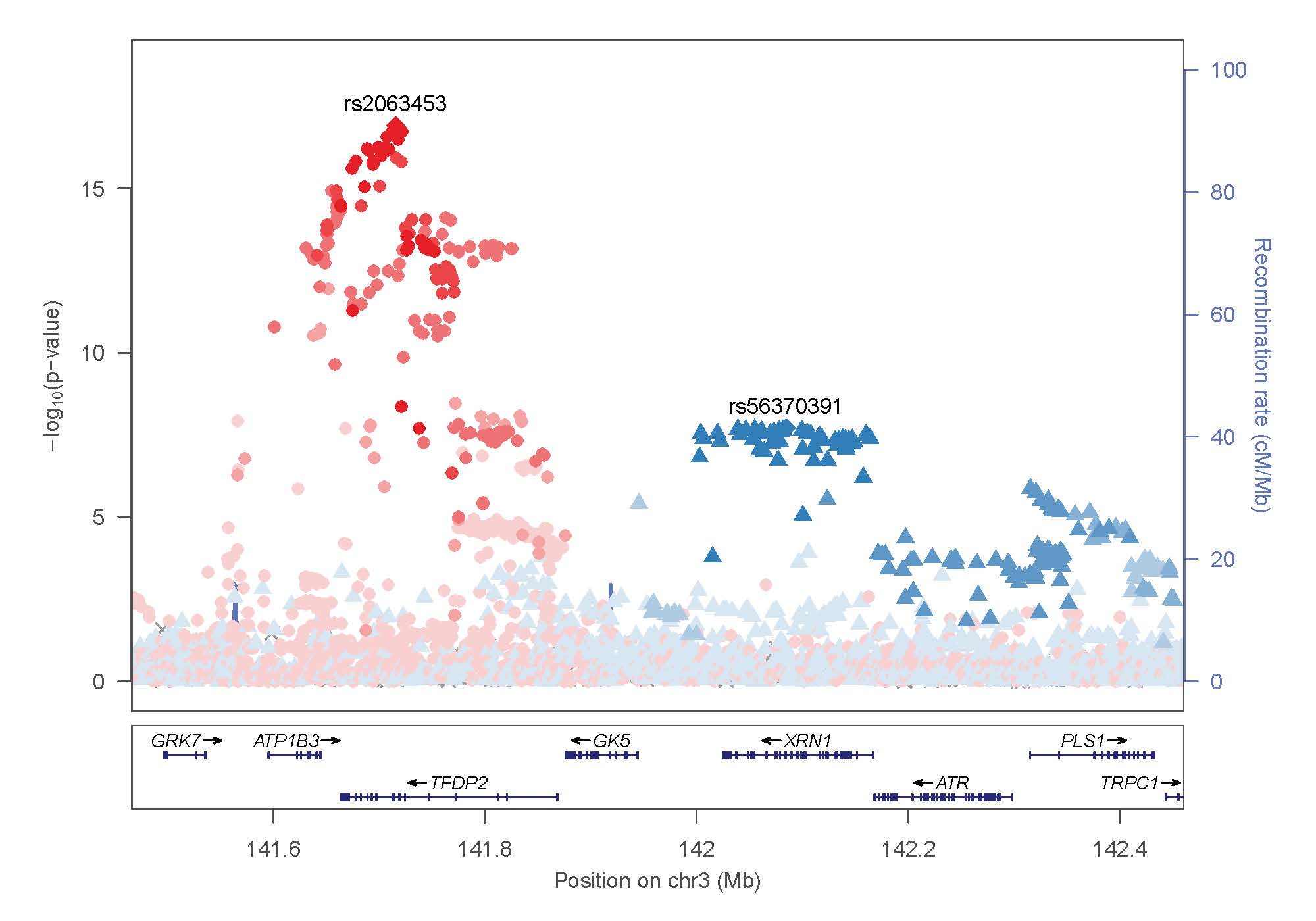

Supplement: Data S3. Regional plots of the identified genetic loci for human head size (±100 kb), related to Figure 1A and 1B [file mmc19.zip › Data S2/rs2063453.jpg]

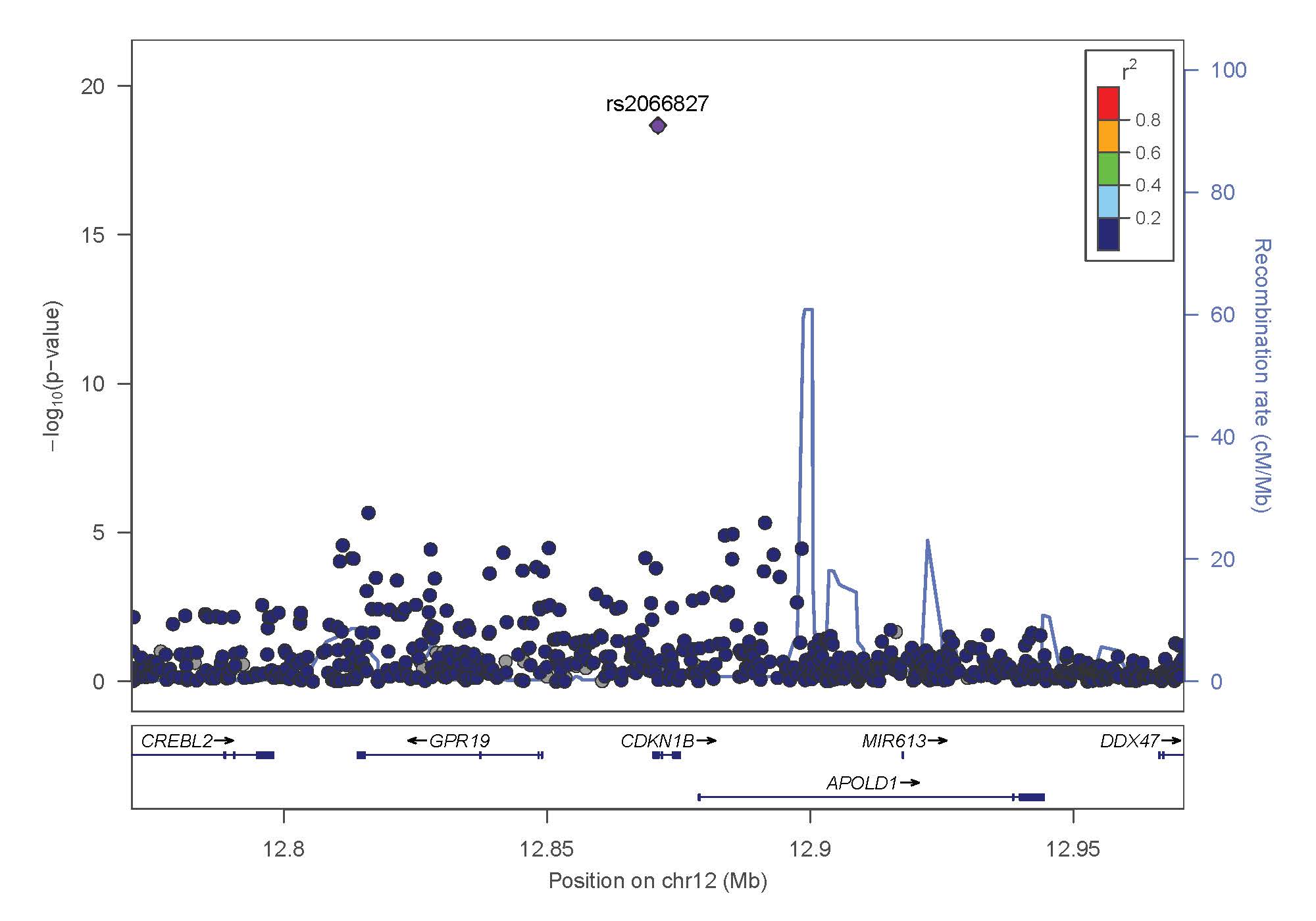

Supplement: Data S3. Regional plots of the identified genetic loci for human head size (±100 kb), related to Figure 1A and 1B [file mmc19.zip › Data S2/rs2066827.jpg]

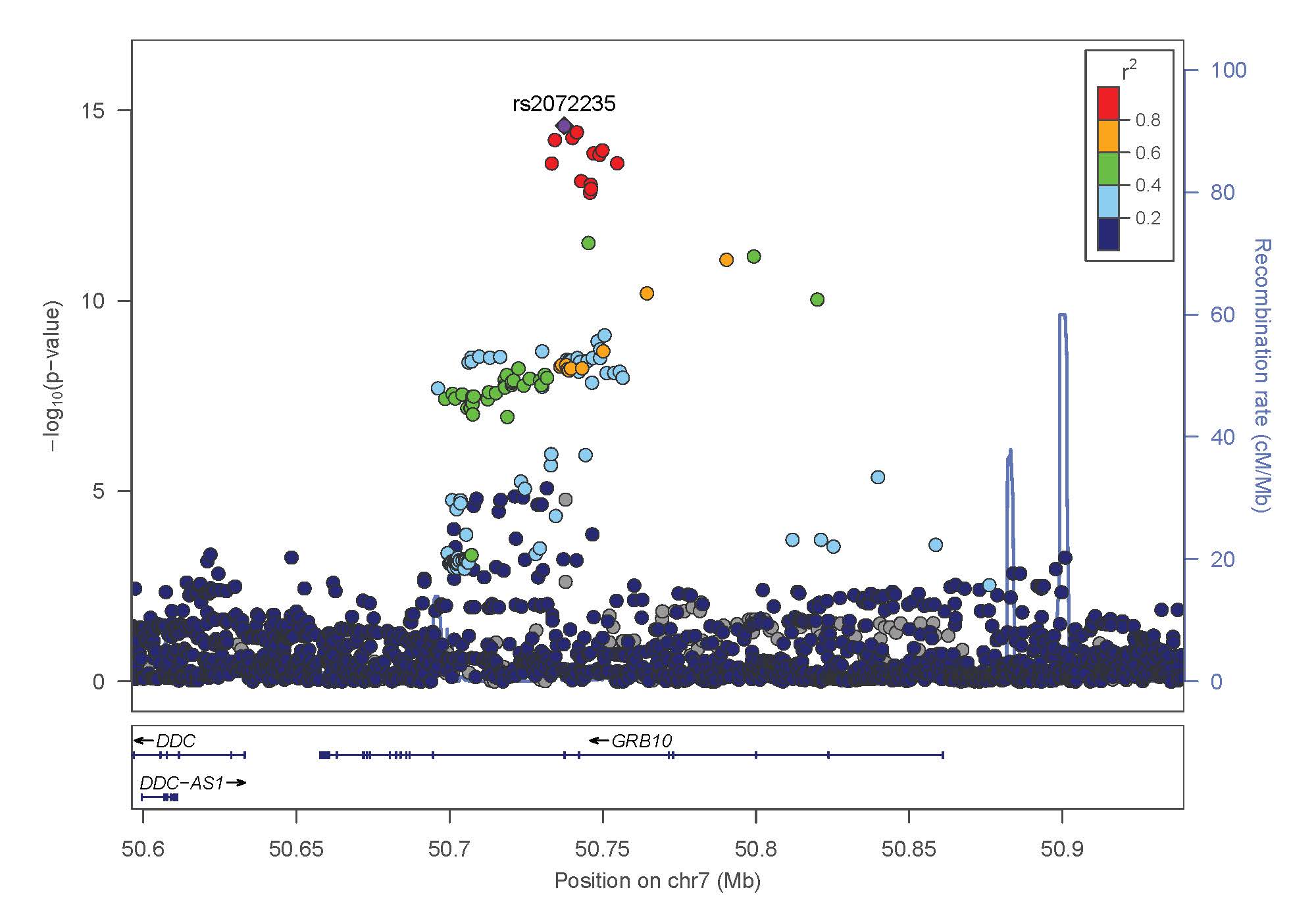

Supplement: Data S3. Regional plots of the identified genetic loci for human head size (±100 kb), related to Figure 1A and 1B [file mmc19.zip › Data S2/rs2072235.jpg]

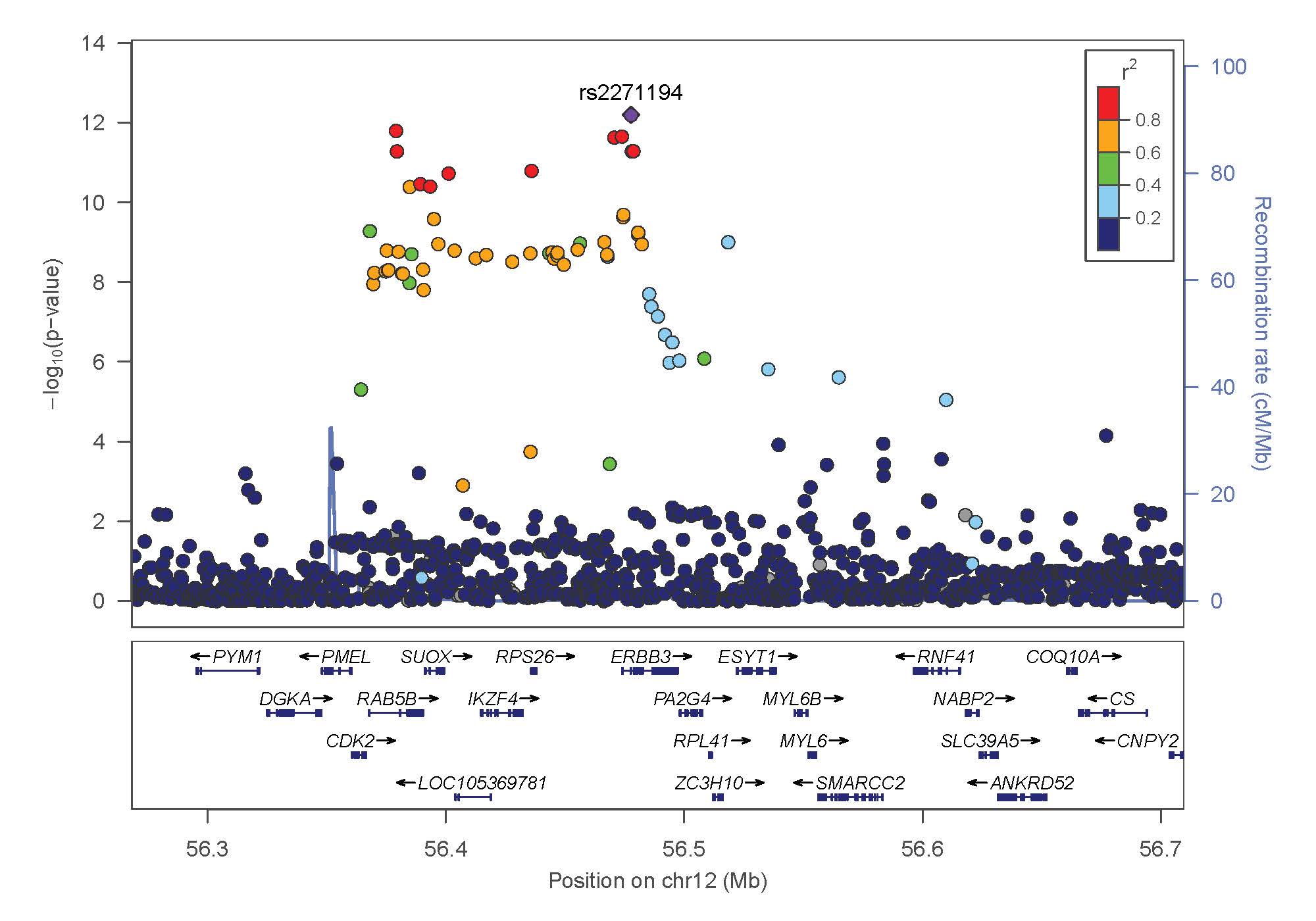

Supplement: Data S3. Regional plots of the identified genetic loci for human head size (±100 kb), related to Figure 1A and 1B [file mmc19.zip › Data S2/rs2271194.jpg]

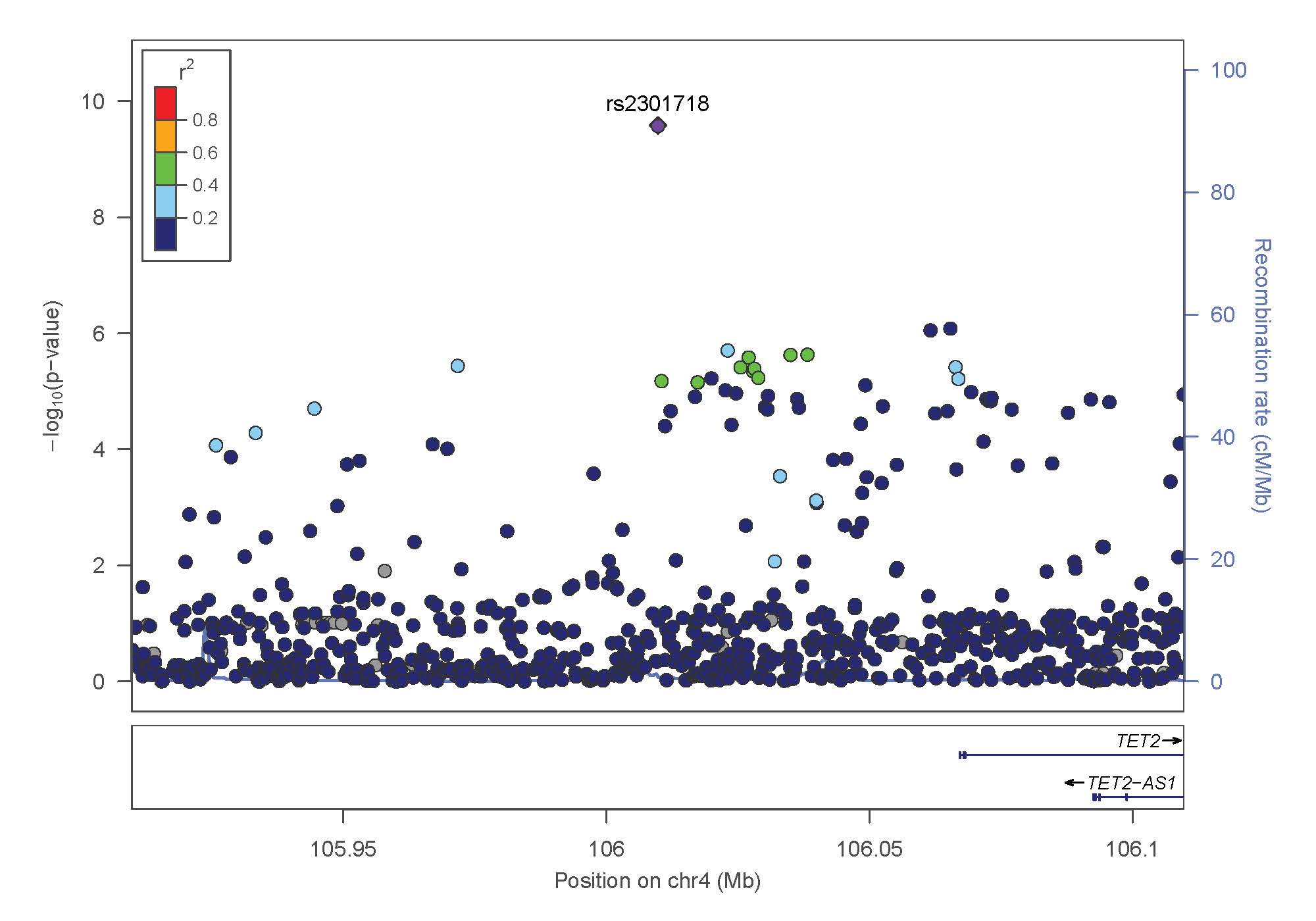

Supplement: Data S3. Regional plots of the identified genetic loci for human head size (±100 kb), related to Figure 1A and 1B [file mmc19.zip › Data S2/rs2301718.jpg]

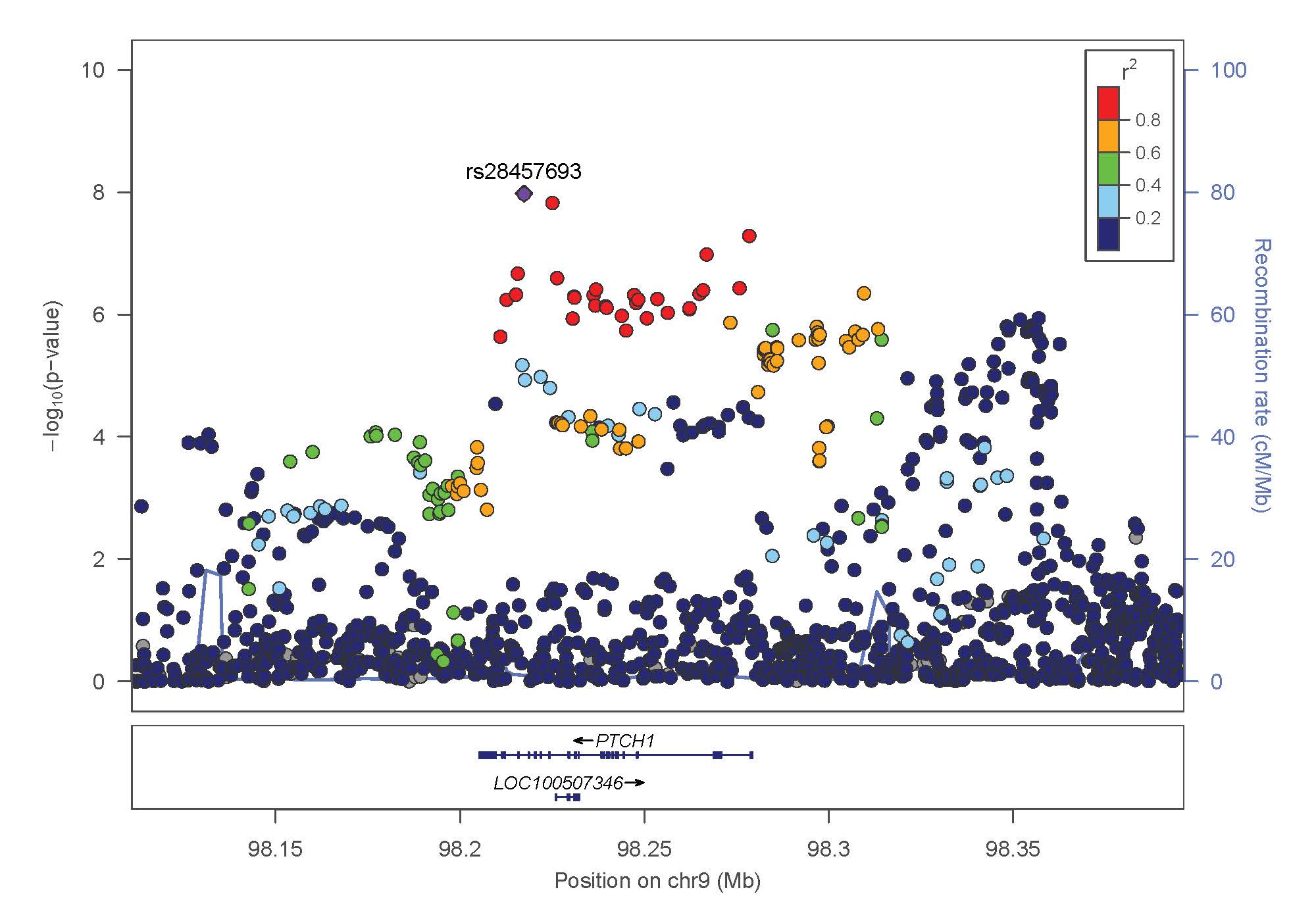

Supplement: Data S3. Regional plots of the identified genetic loci for human head size (±100 kb), related to Figure 1A and 1B [file mmc19.zip › Data S2/rs28457693.jpg]

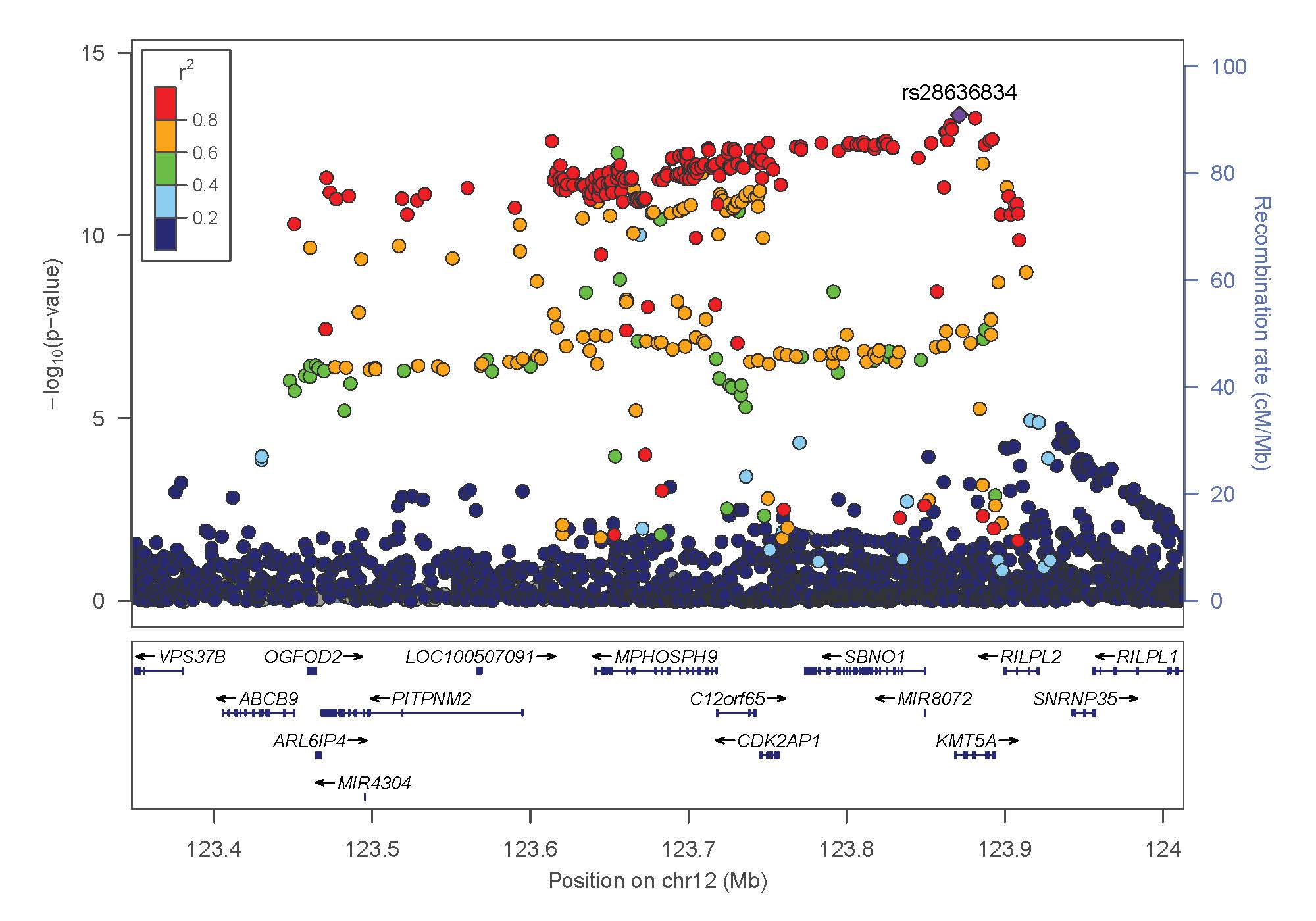

Supplement: Data S3. Regional plots of the identified genetic loci for human head size (±100 kb), related to Figure 1A and 1B [file mmc19.zip › Data S2/rs28636834.jpg]

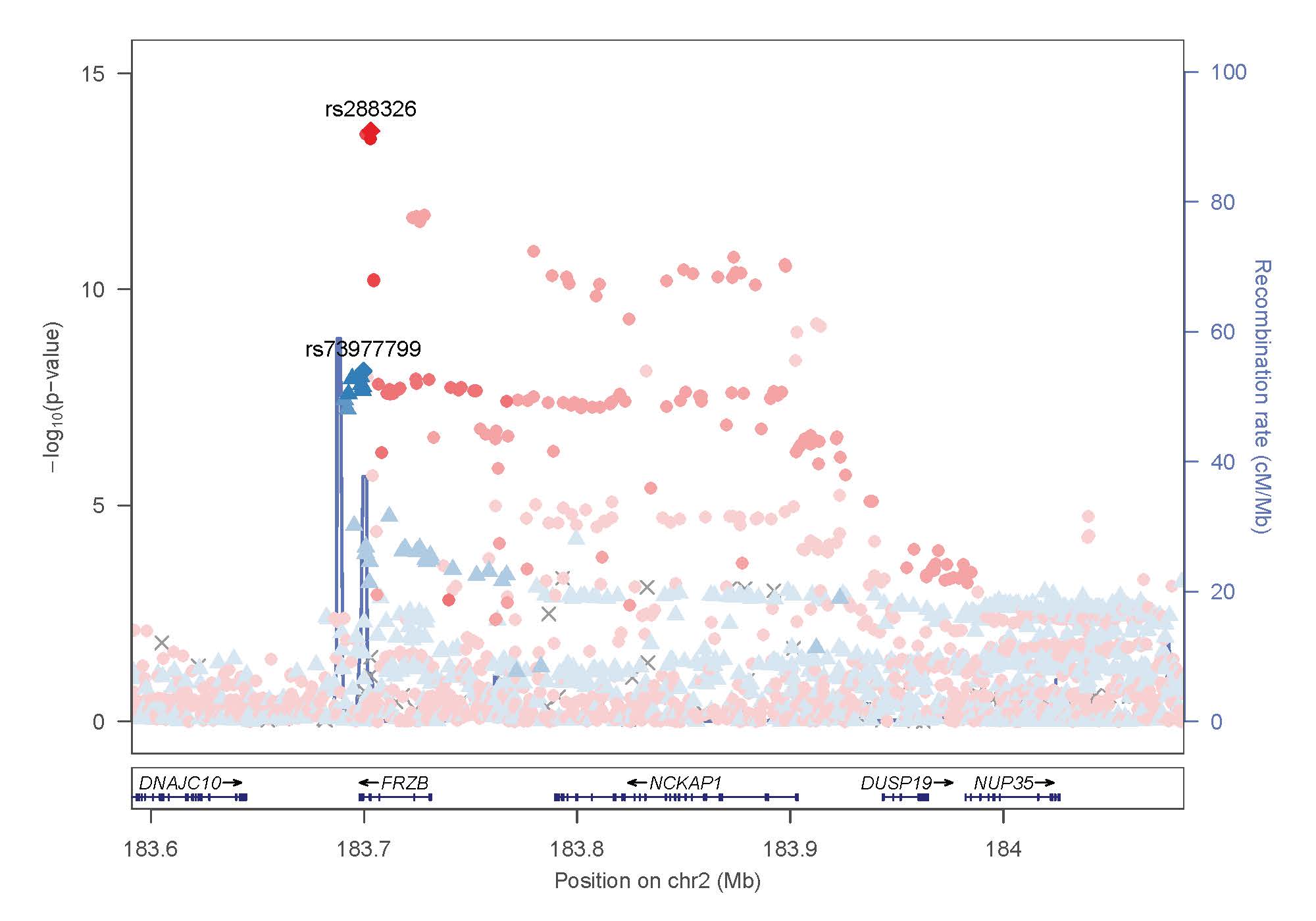

Supplement: Data S3. Regional plots of the identified genetic loci for human head size (±100 kb), related to Figure 1A and 1B [file mmc19.zip › Data S2/rs288326.jpg]

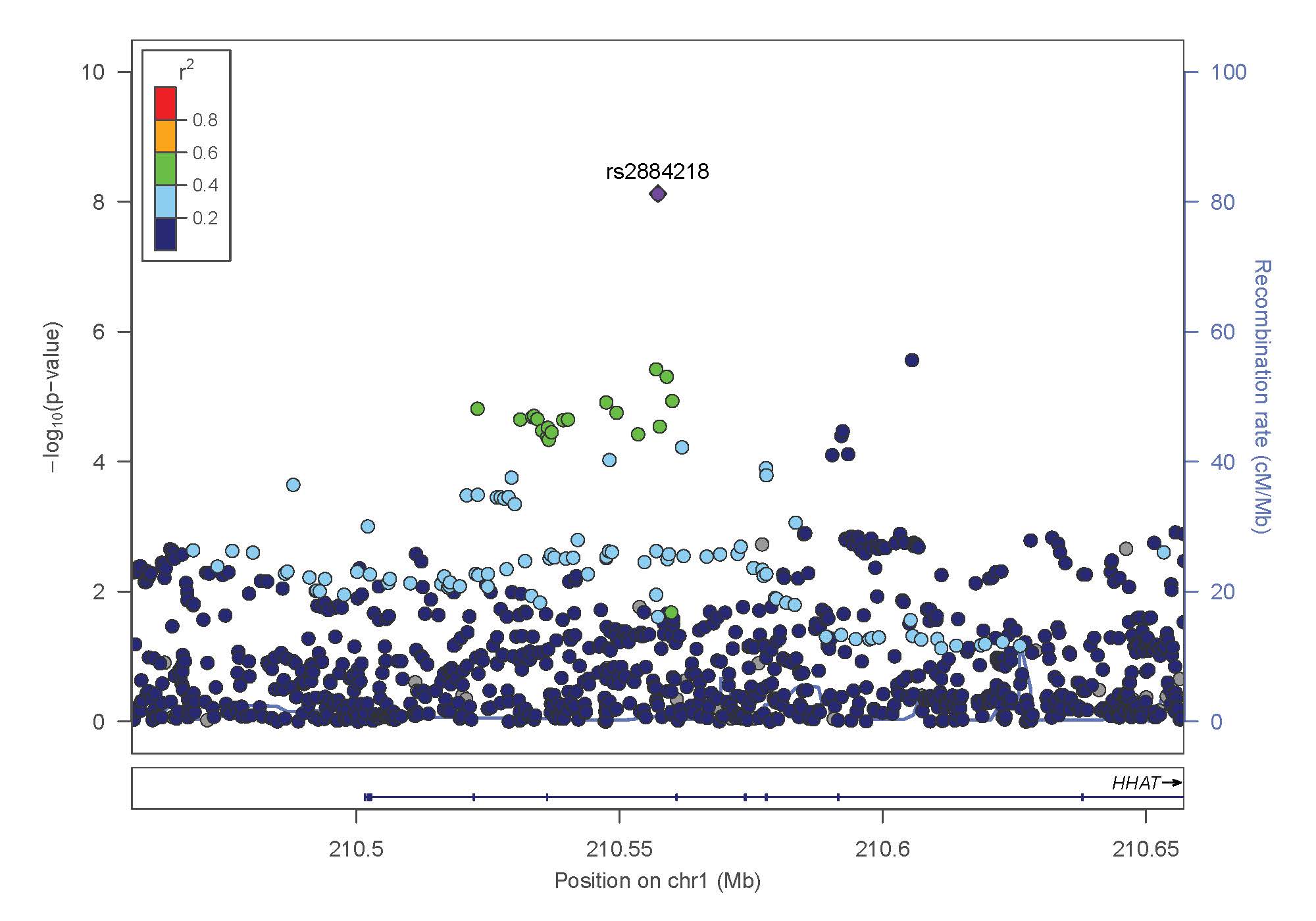

Supplement: Data S3. Regional plots of the identified genetic loci for human head size (±100 kb), related to Figure 1A and 1B [file mmc19.zip › Data S2/rs2884218.jpg]

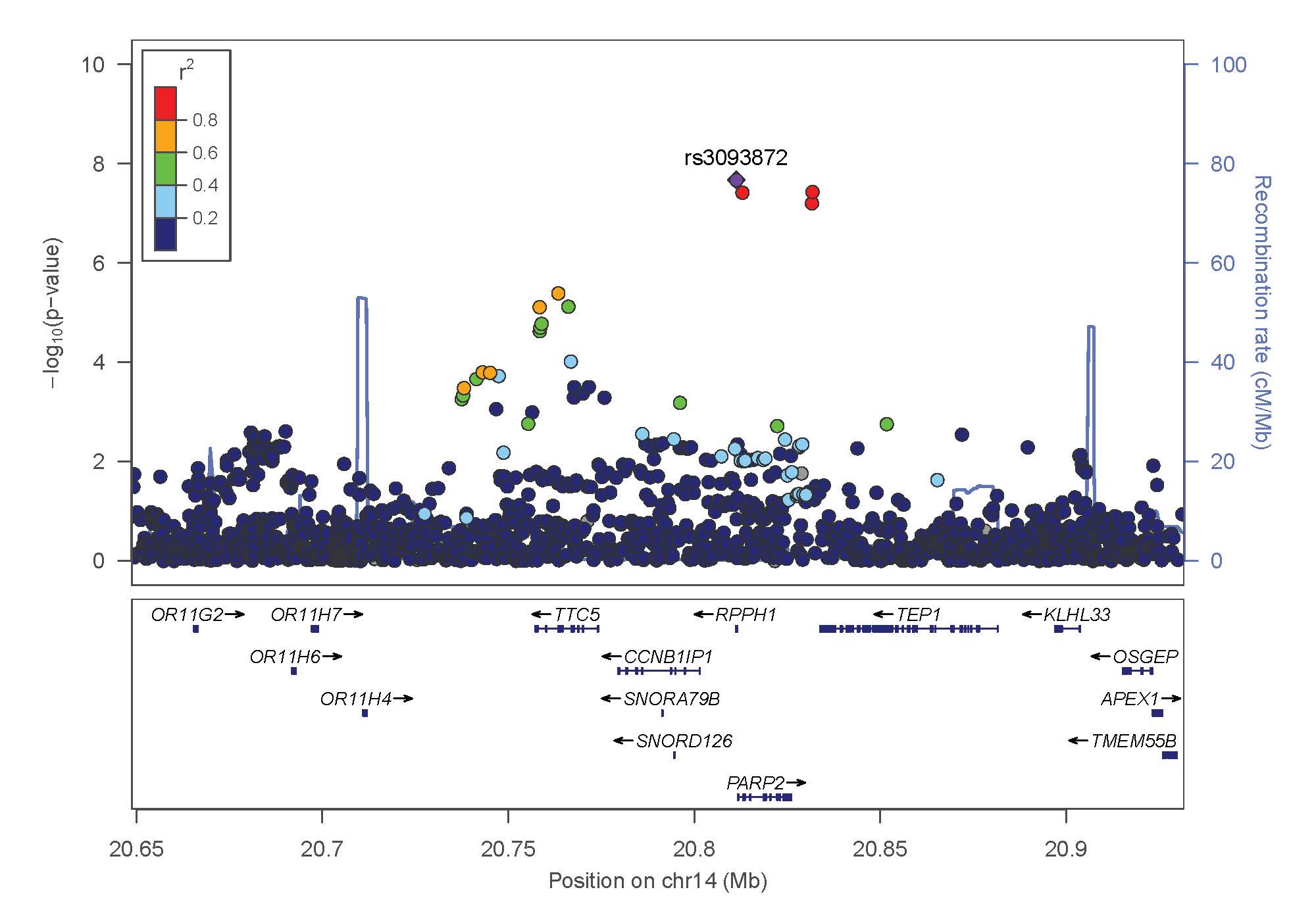

Supplement: Data S3. Regional plots of the identified genetic loci for human head size (±100 kb), related to Figure 1A and 1B [file mmc19.zip › Data S2/rs3093872.jpg]

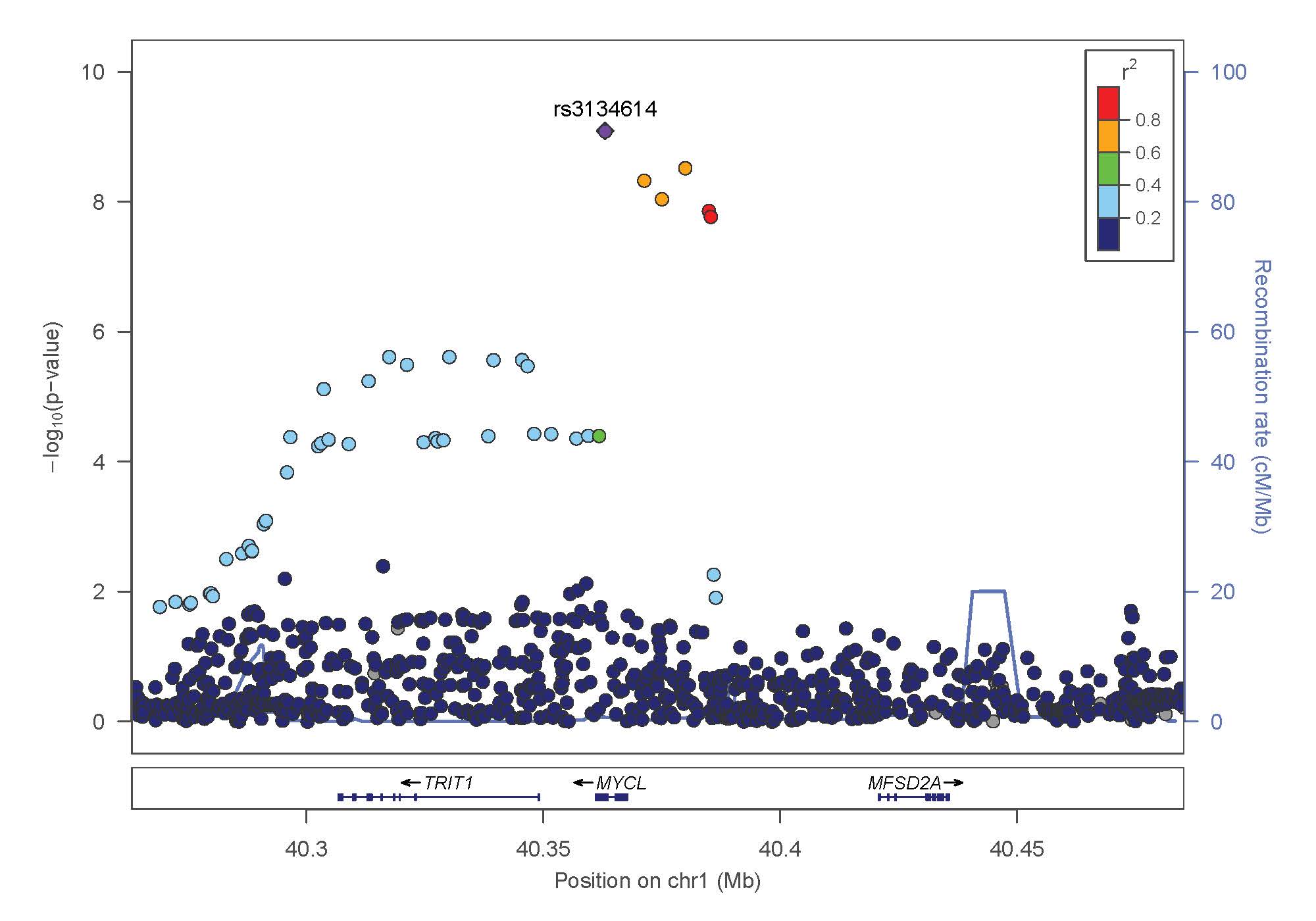

Supplement: Data S3. Regional plots of the identified genetic loci for human head size (±100 kb), related to Figure 1A and 1B [file mmc19.zip › Data S2/rs3134614.jpg]

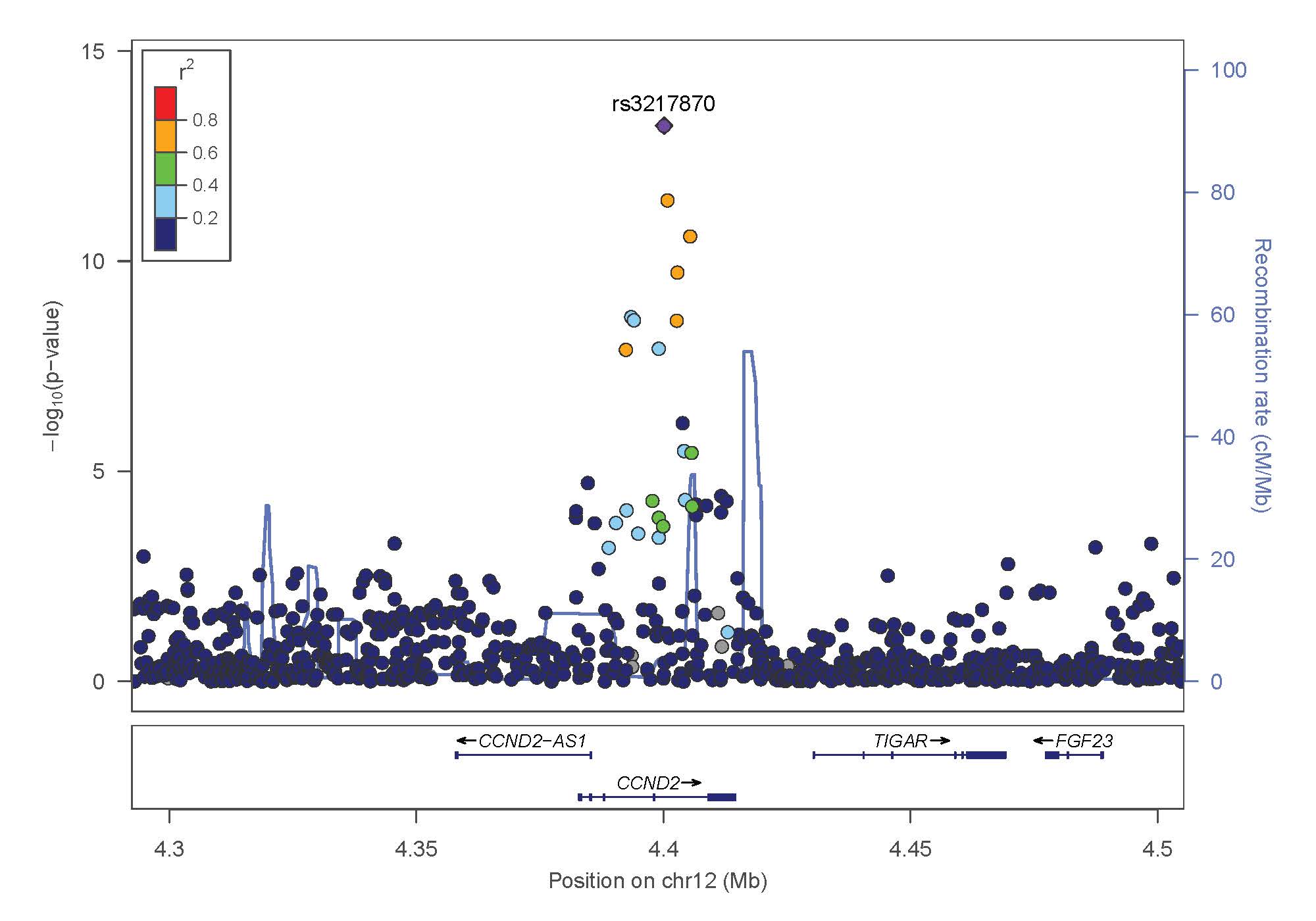

Supplement: Data S3. Regional plots of the identified genetic loci for human head size (±100 kb), related to Figure 1A and 1B [file mmc19.zip › Data S2/rs3217870.jpg]

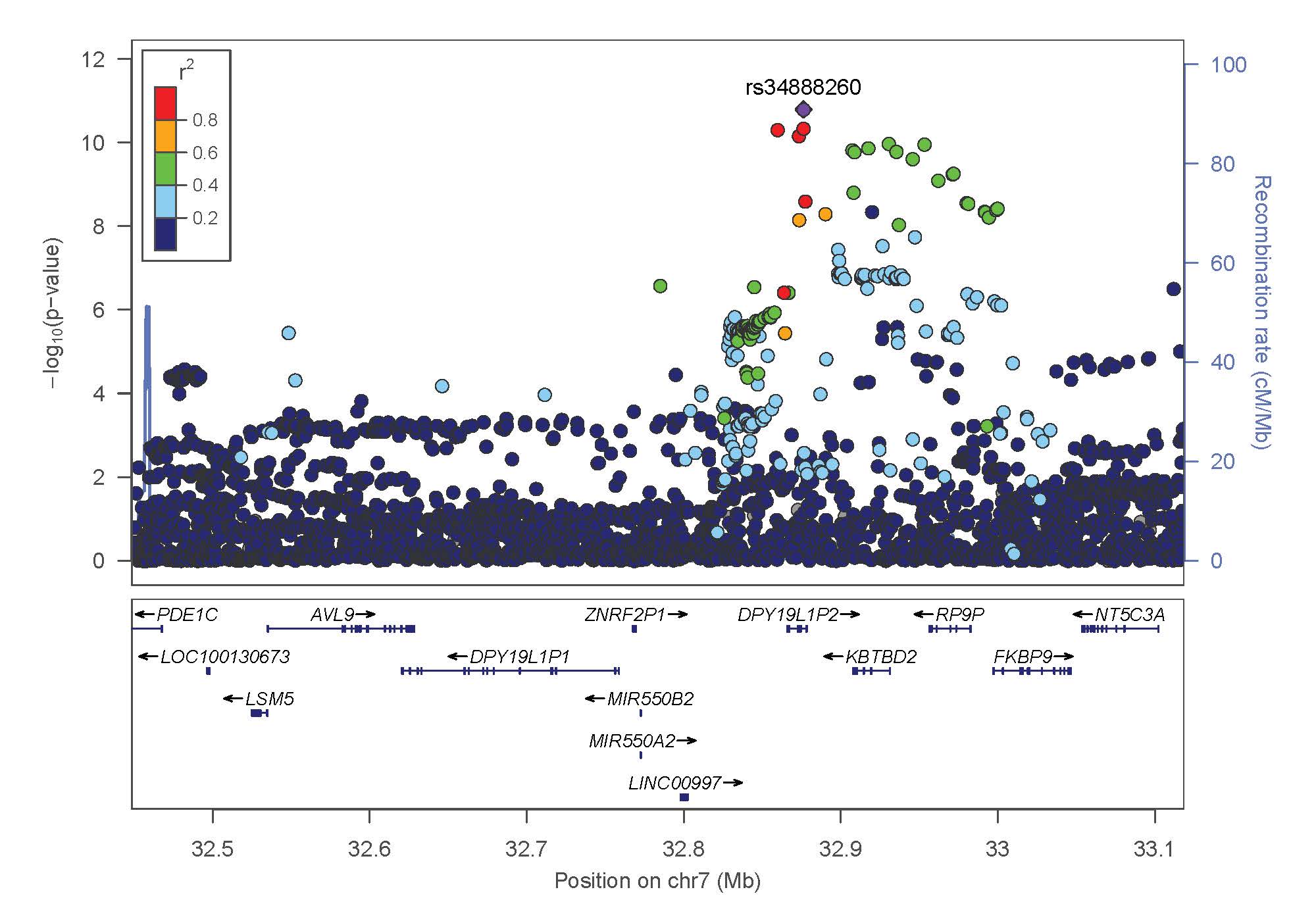

Supplement: Data S3. Regional plots of the identified genetic loci for human head size (±100 kb), related to Figure 1A and 1B [file mmc19.zip › Data S2/rs34888260.jpg]

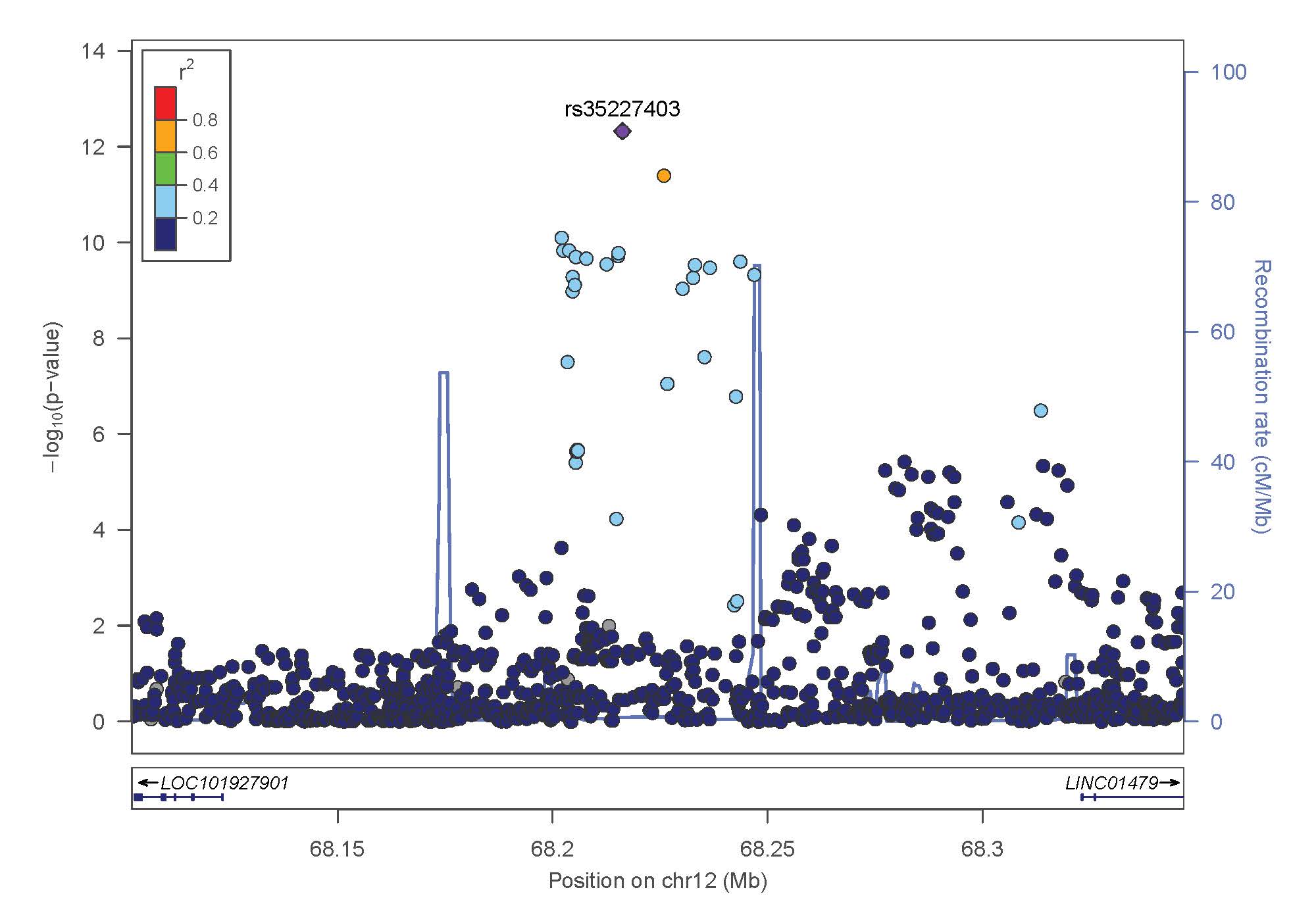

Supplement: Data S3. Regional plots of the identified genetic loci for human head size (±100 kb), related to Figure 1A and 1B [file mmc19.zip › Data S2/rs35227403.jpg]

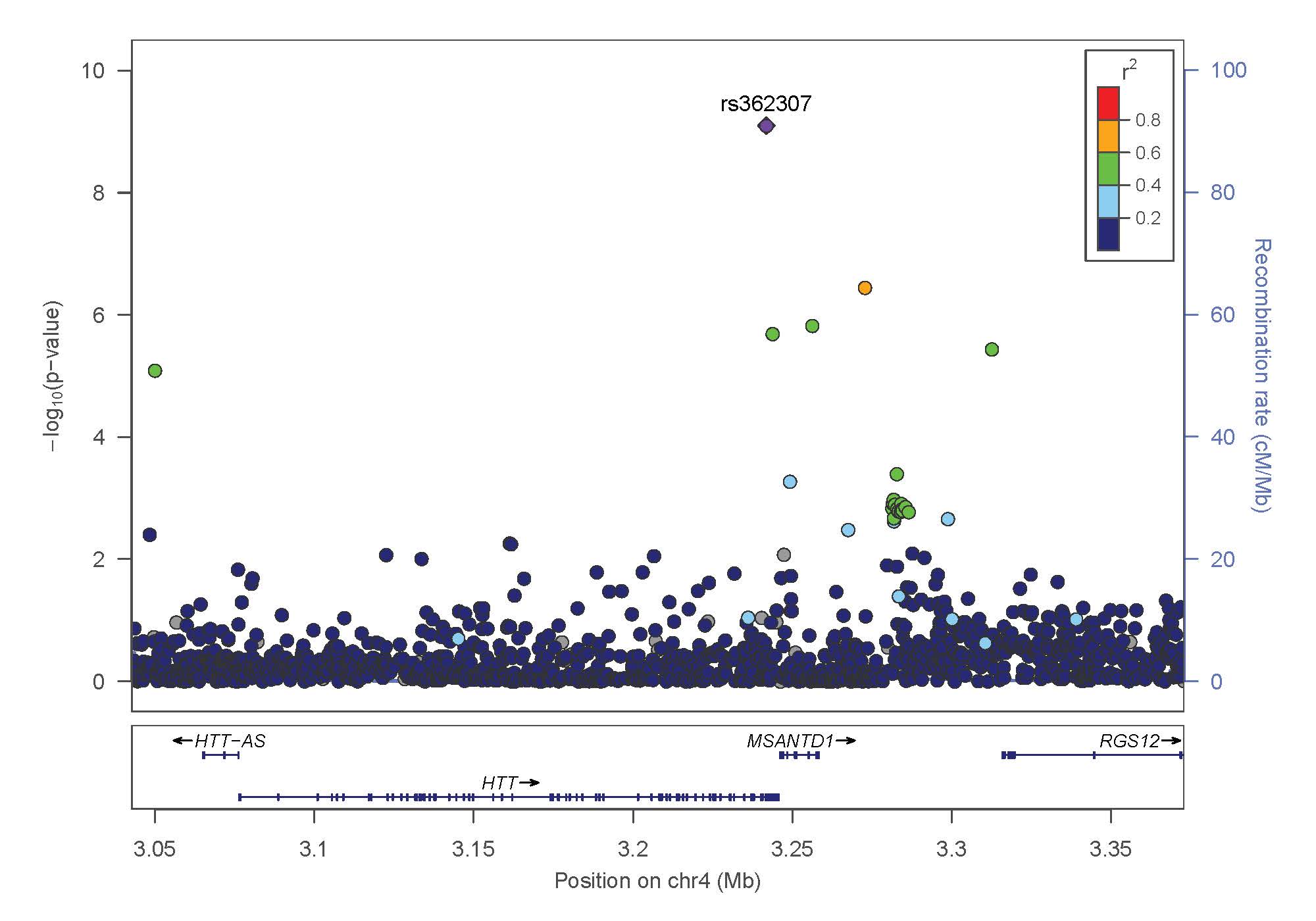

Supplement: Data S3. Regional plots of the identified genetic loci for human head size (±100 kb), related to Figure 1A and 1B [file mmc19.zip › Data S2/rs362307.jpg]

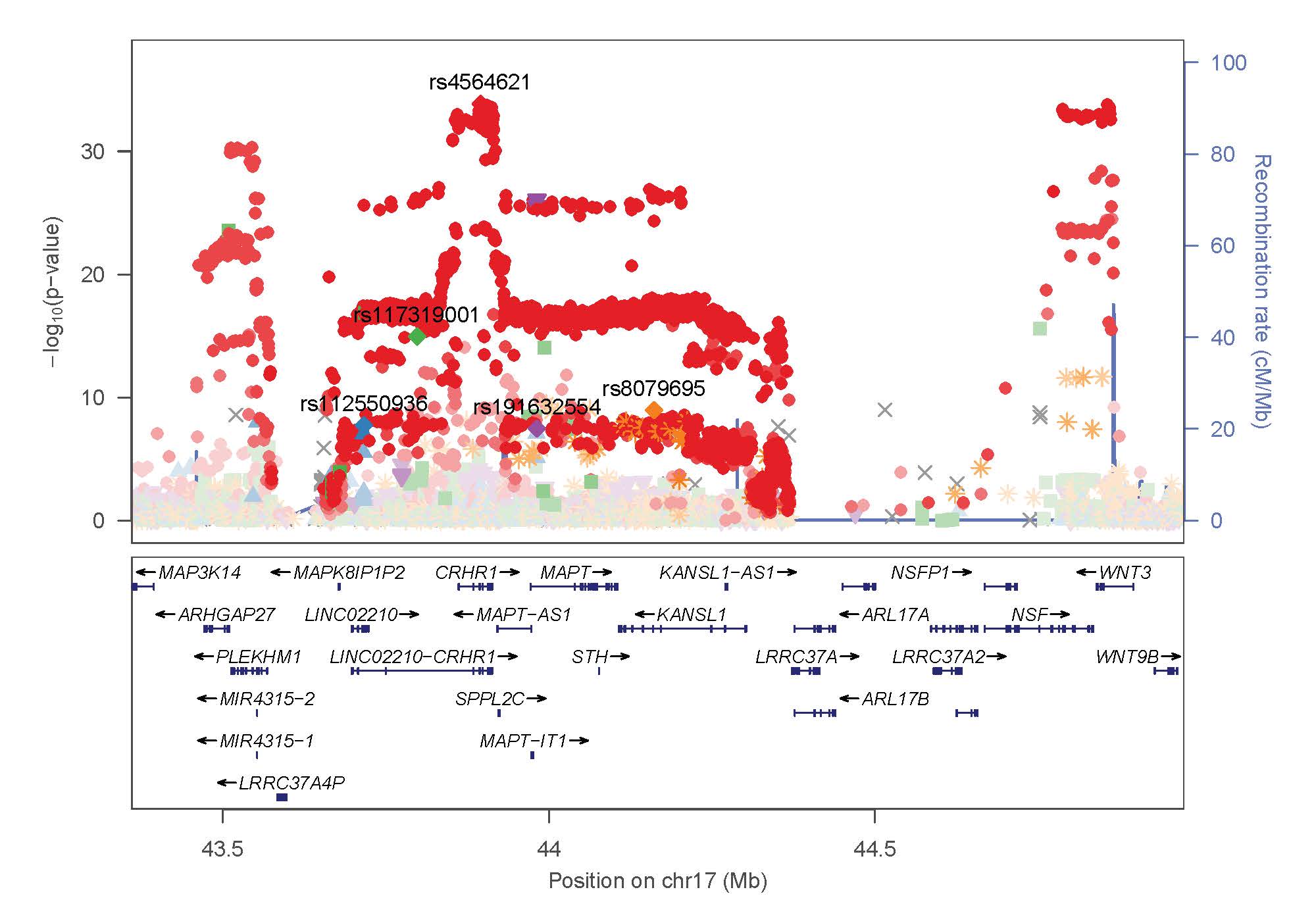

Supplement: Data S3. Regional plots of the identified genetic loci for human head size (±100 kb), related to Figure 1A and 1B [file mmc19.zip › Data S2/rs4564621.jpg]

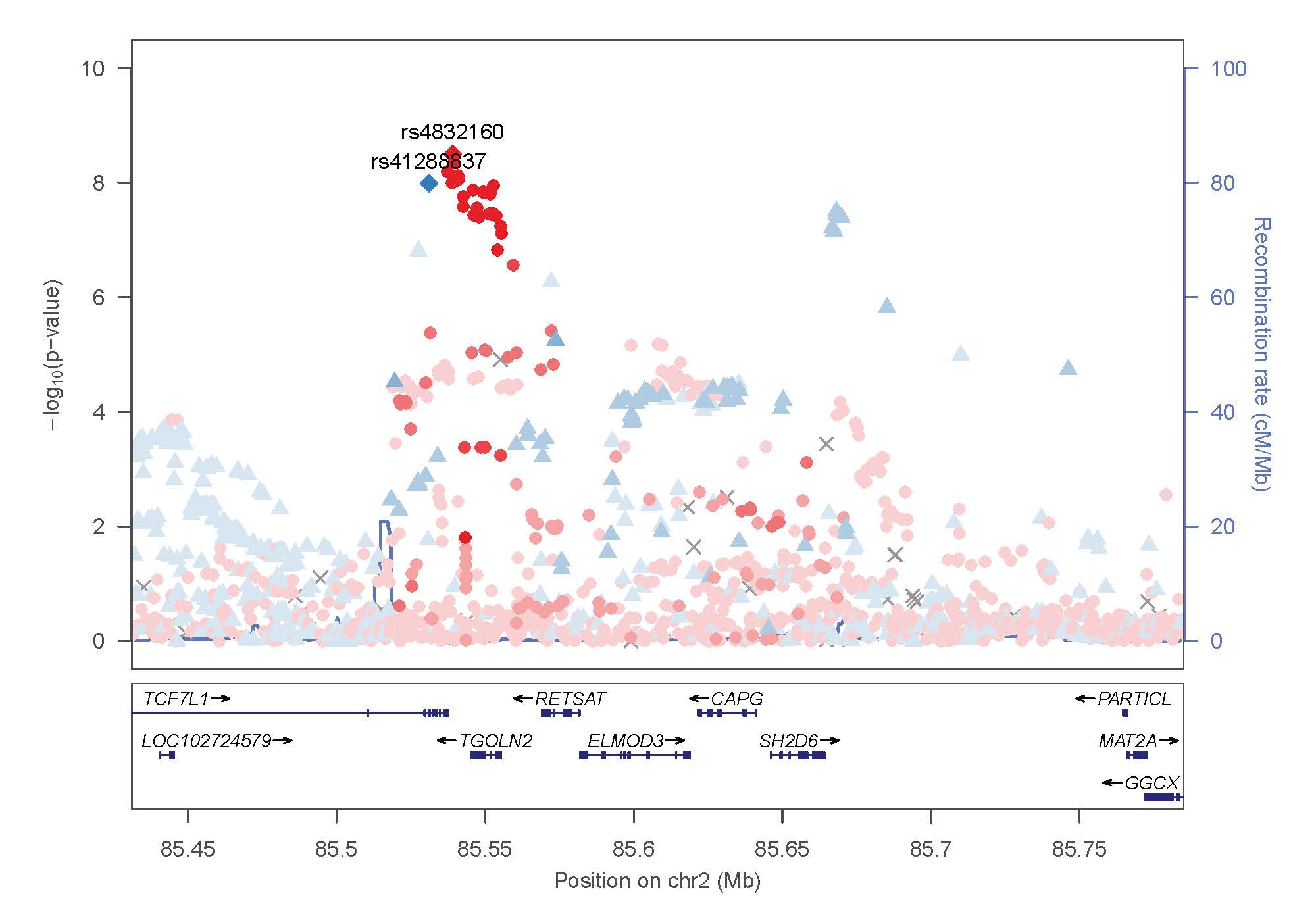

Supplement: Data S3. Regional plots of the identified genetic loci for human head size (±100 kb), related to Figure 1A and 1B [file mmc19.zip › Data S2/rs4832160.jpg]

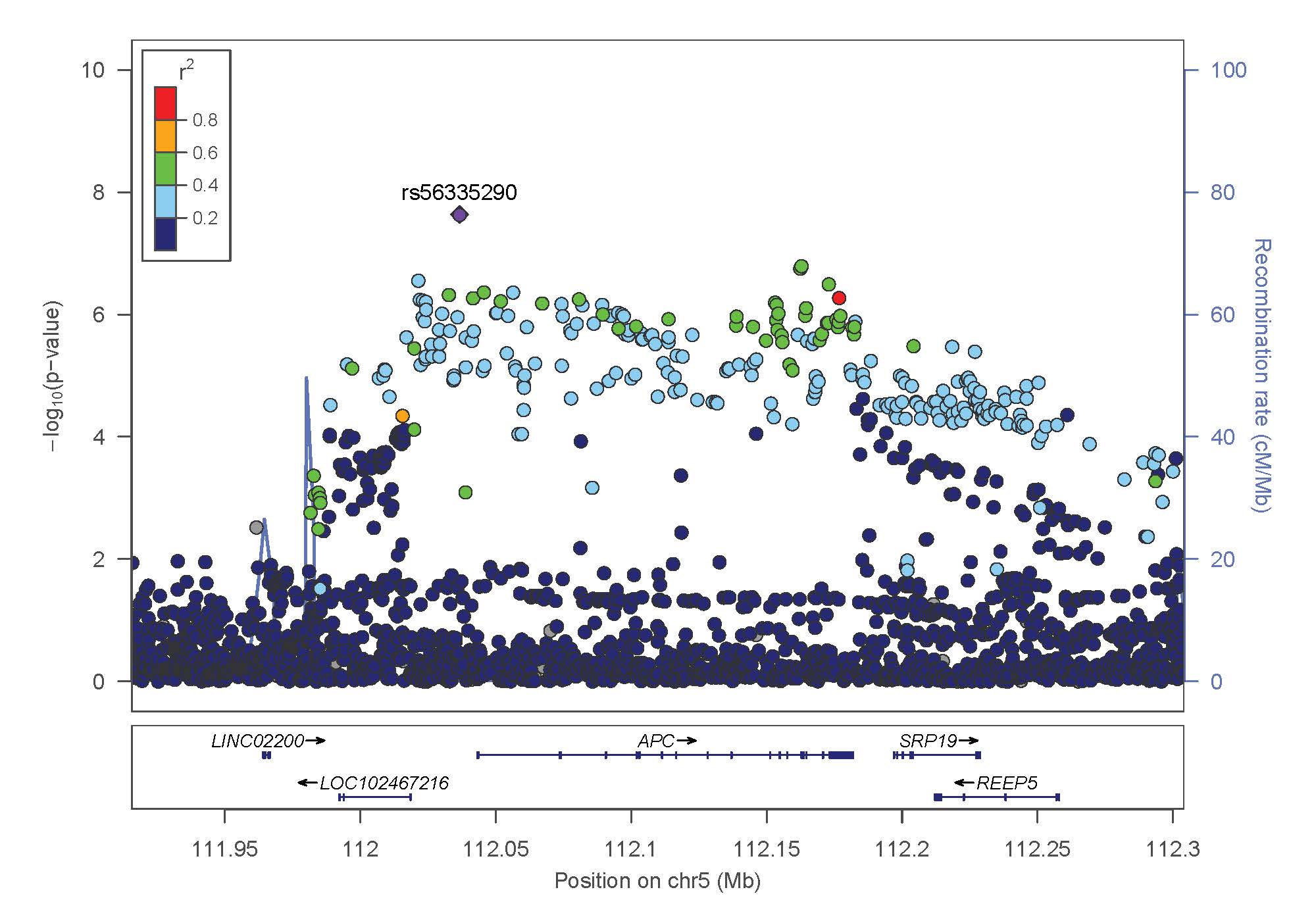

Supplement: Data S3. Regional plots of the identified genetic loci for human head size (±100 kb), related to Figure 1A and 1B [file mmc19.zip › Data S2/rs56335290.jpg]

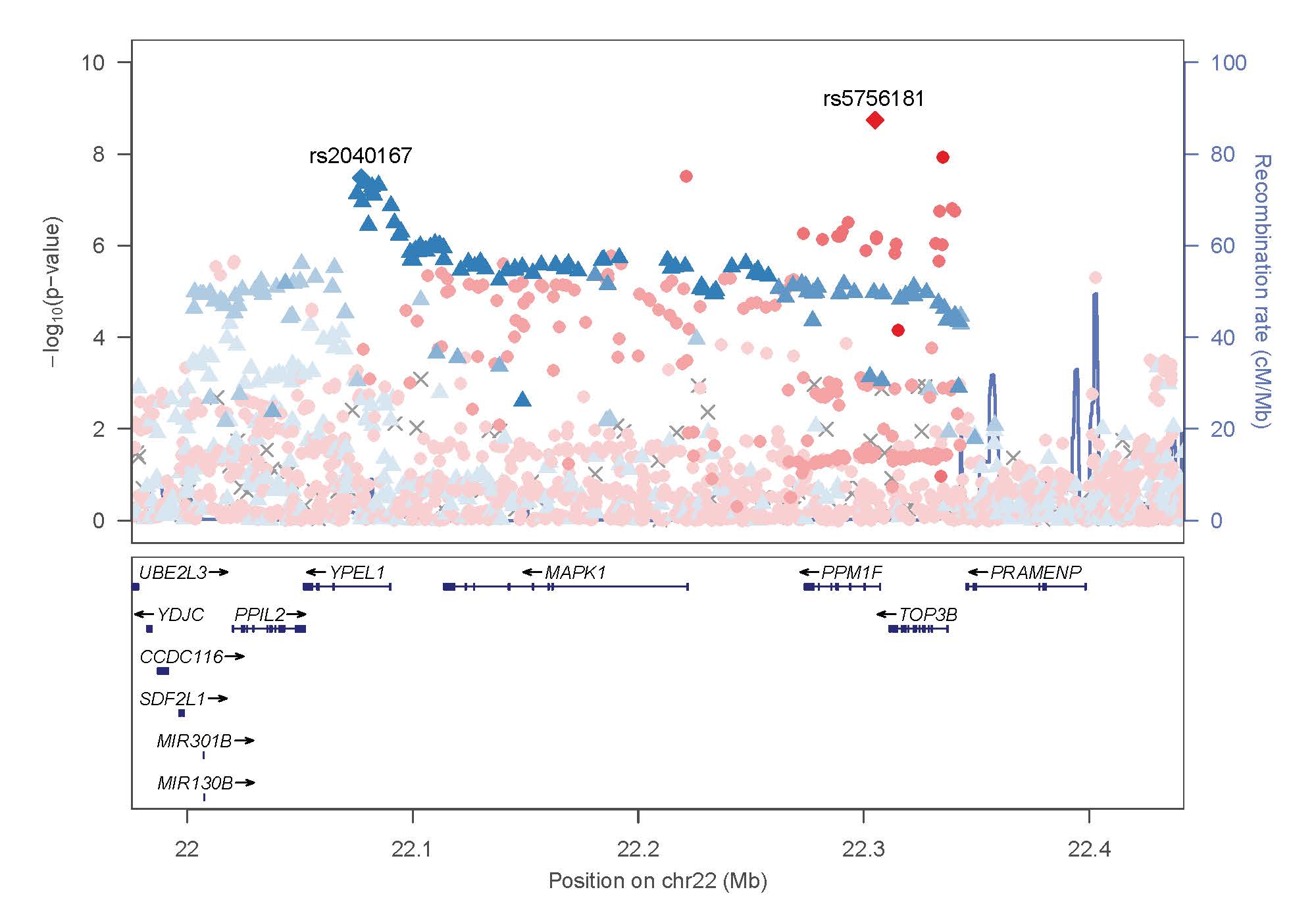

Supplement: Data S3. Regional plots of the identified genetic loci for human head size (±100 kb), related to Figure 1A and 1B [file mmc19.zip › Data S2/rs5756181.jpg]

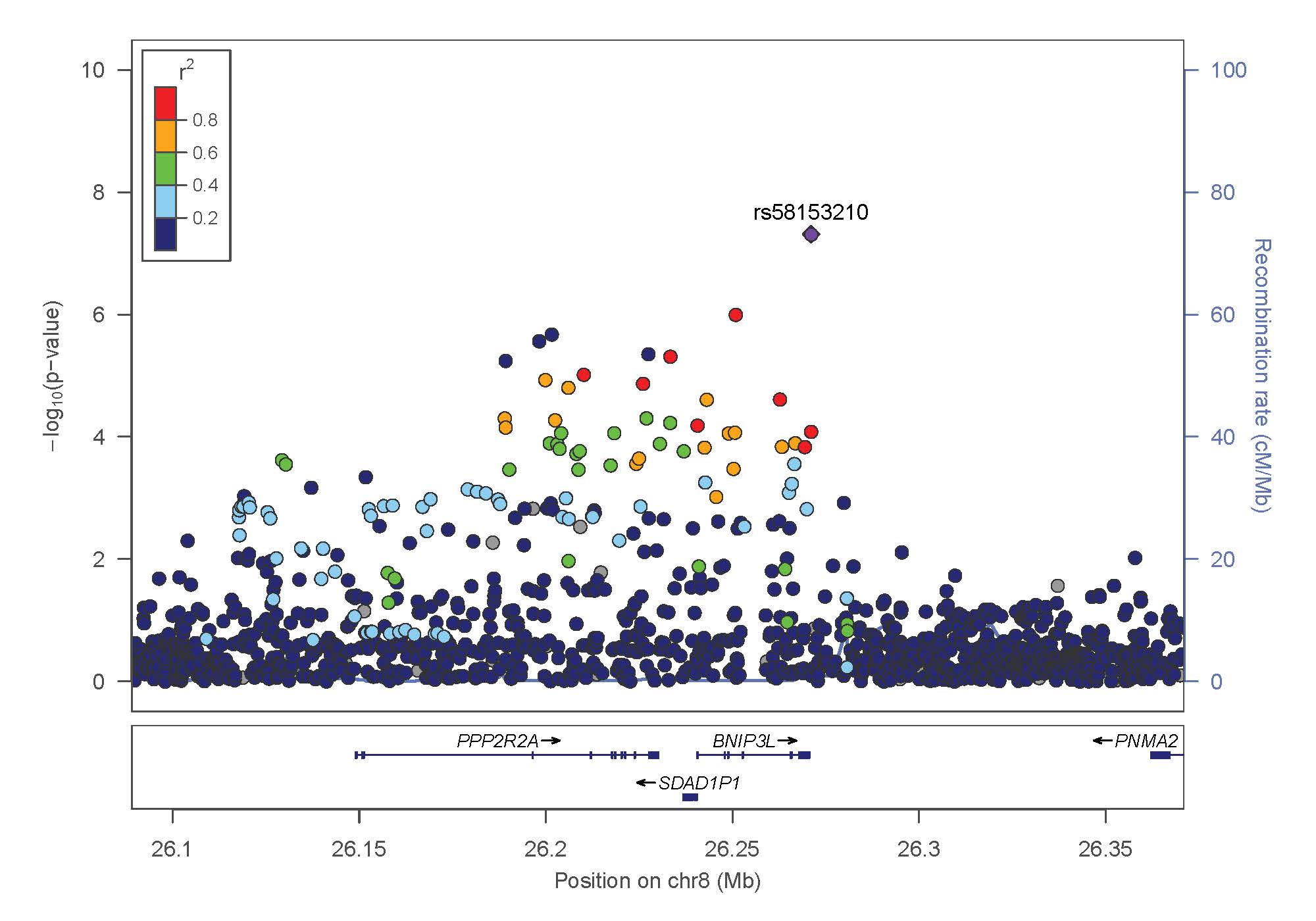

Supplement: Data S3. Regional plots of the identified genetic loci for human head size (±100 kb), related to Figure 1A and 1B [file mmc19.zip › Data S2/rs58153210.jpg]

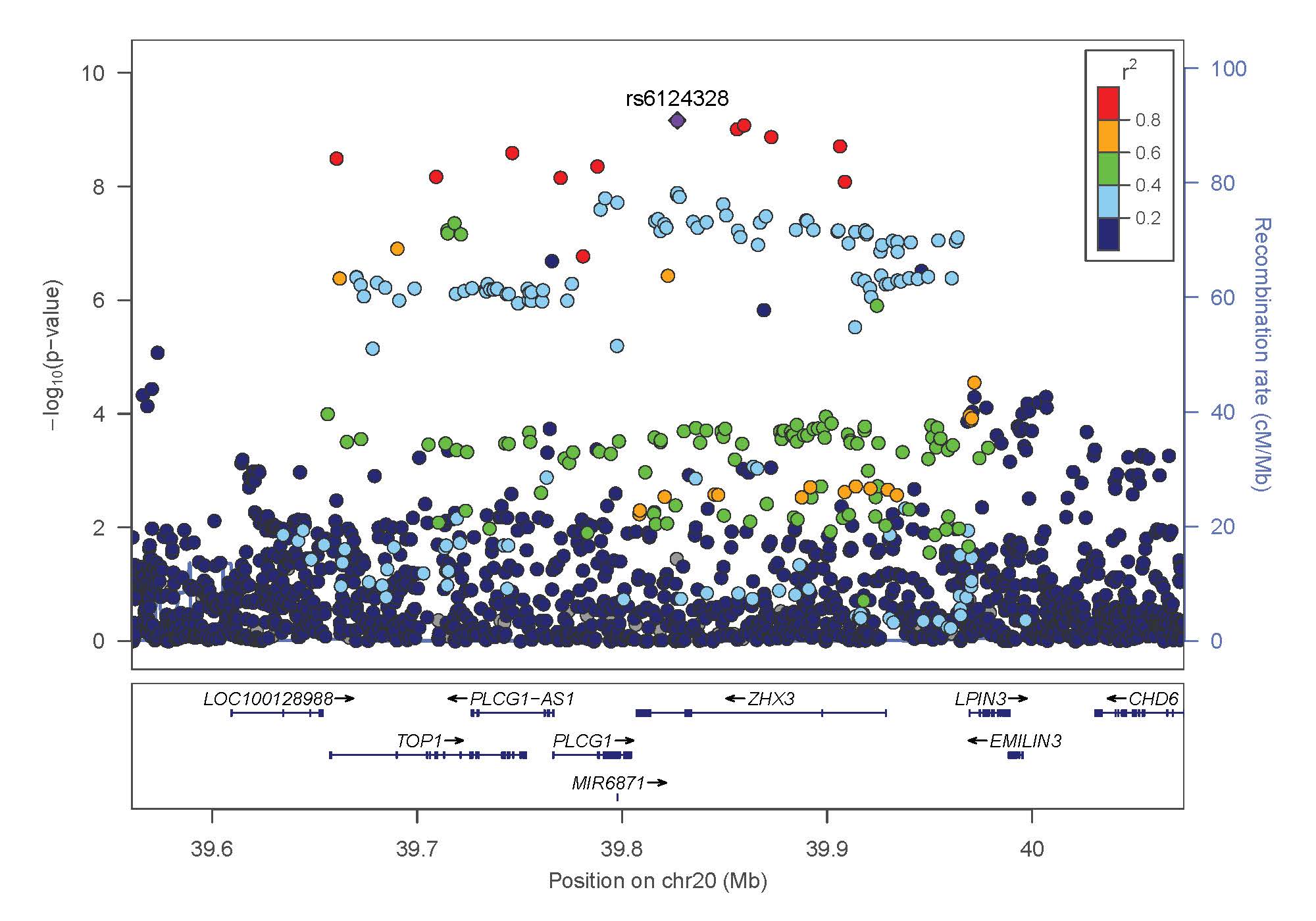

Supplement: Data S3. Regional plots of the identified genetic loci for human head size (±100 kb), related to Figure 1A and 1B [file mmc19.zip › Data S2/rs6124328.jpg]

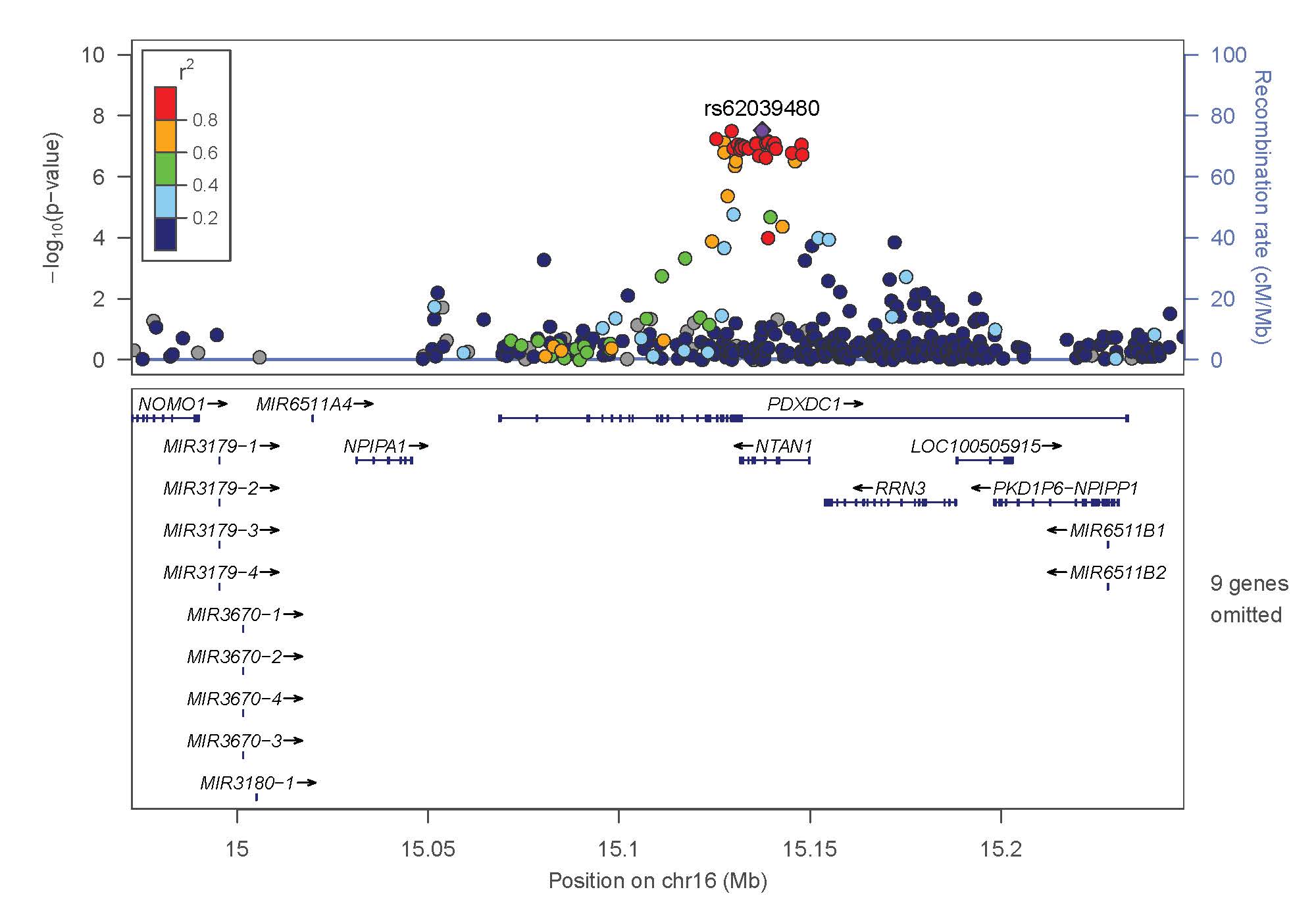

Supplement: Data S3. Regional plots of the identified genetic loci for human head size (±100 kb), related to Figure 1A and 1B [file mmc19.zip › Data S2/rs62039480.jpg]

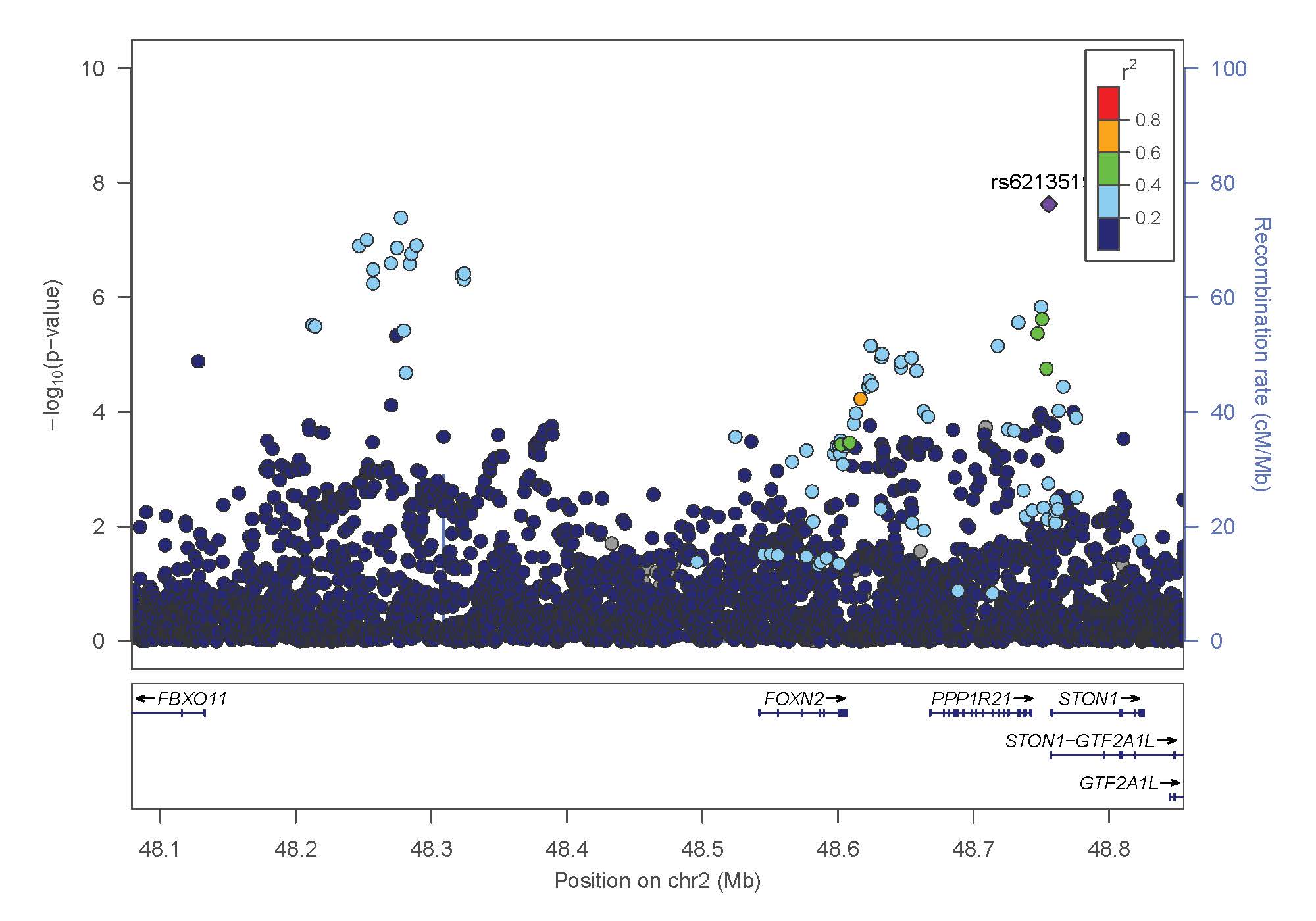

Supplement: Data S3. Regional plots of the identified genetic loci for human head size (±100 kb), related to Figure 1A and 1B [file mmc19.zip › Data S2/rs62135193.jpg]

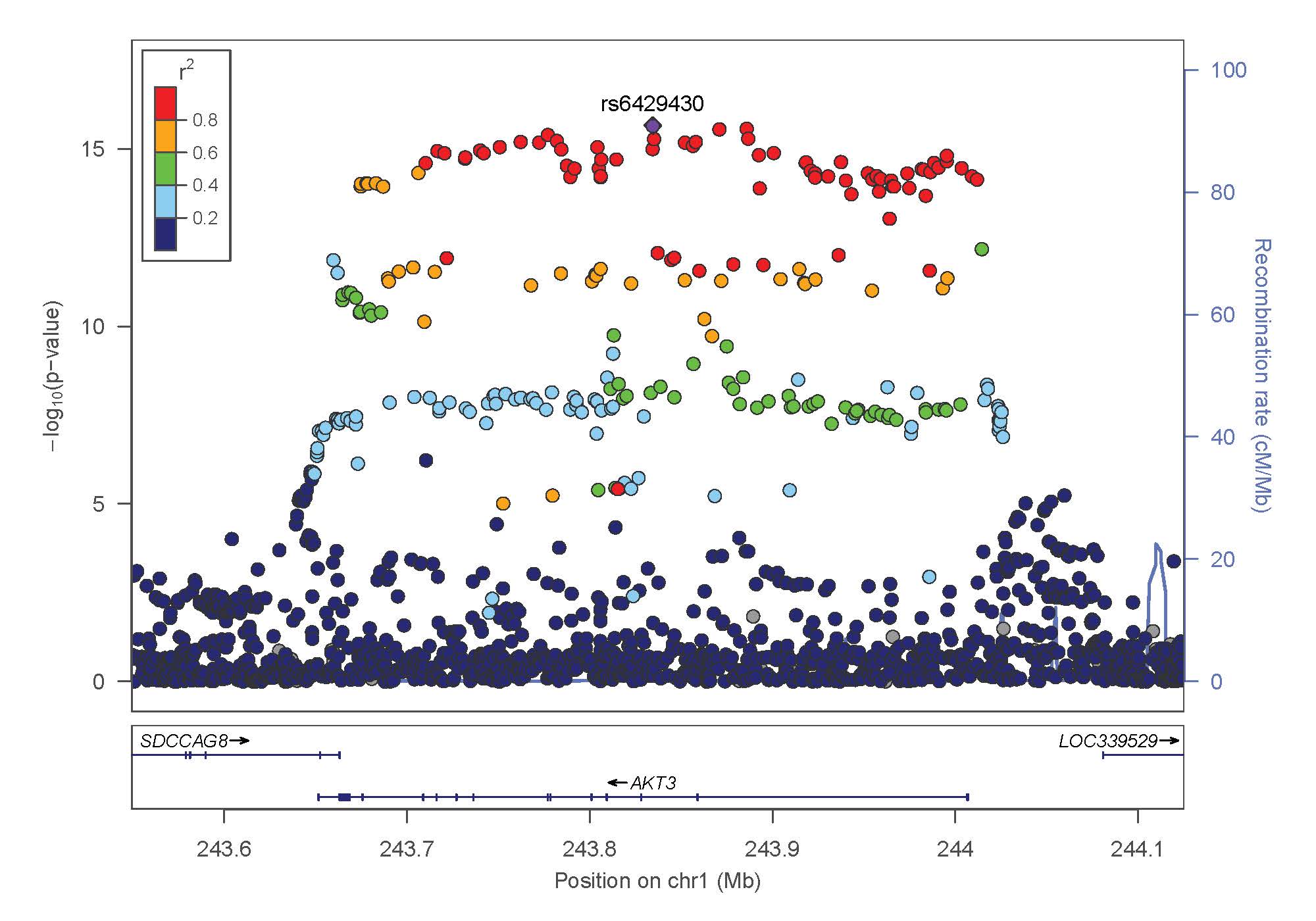

Supplement: Data S3. Regional plots of the identified genetic loci for human head size (±100 kb), related to Figure 1A and 1B [file mmc19.zip › Data S2/rs6429430.jpg]

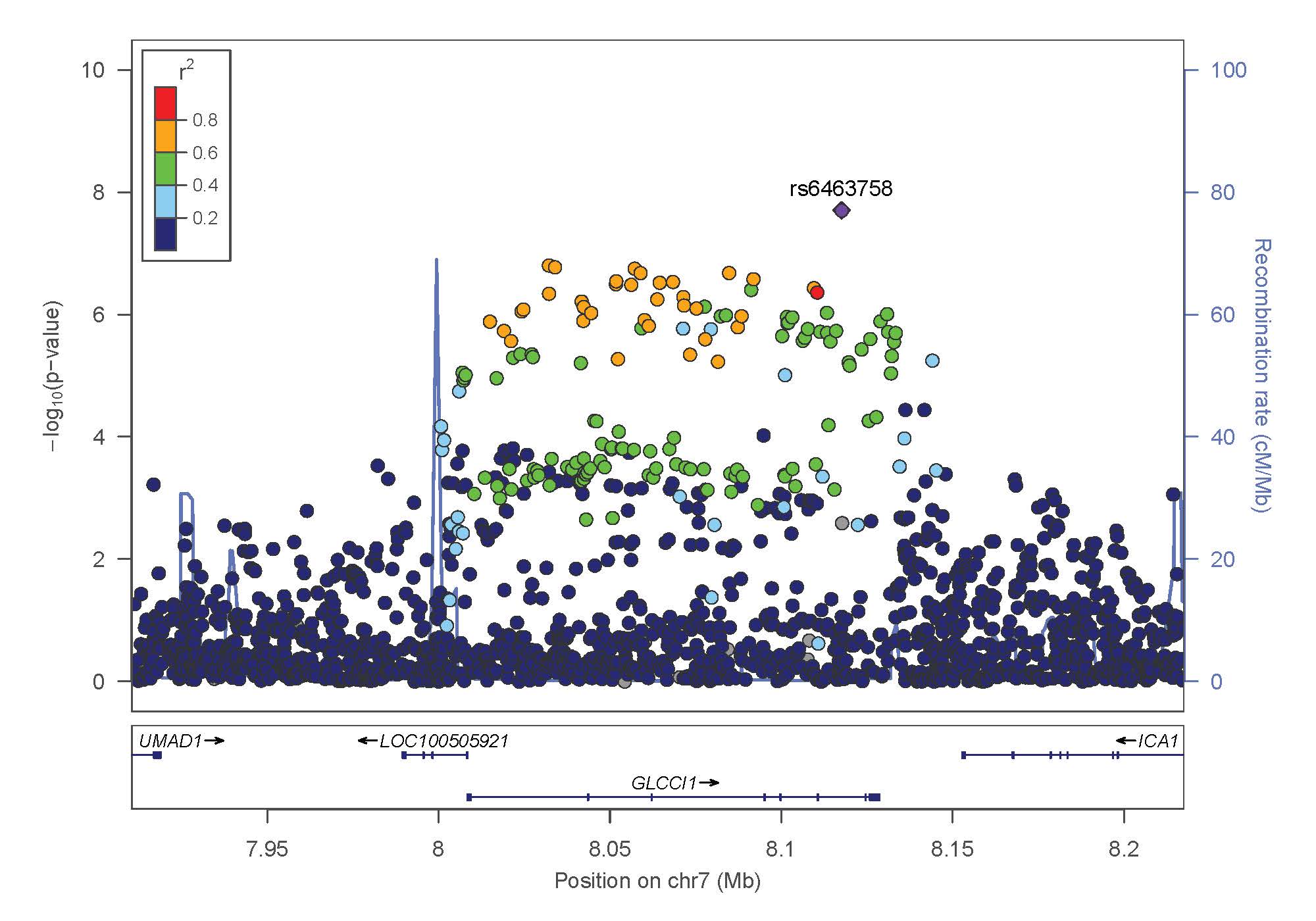

Supplement: Data S3. Regional plots of the identified genetic loci for human head size (±100 kb), related to Figure 1A and 1B [file mmc19.zip › Data S2/rs6463758.jpg]

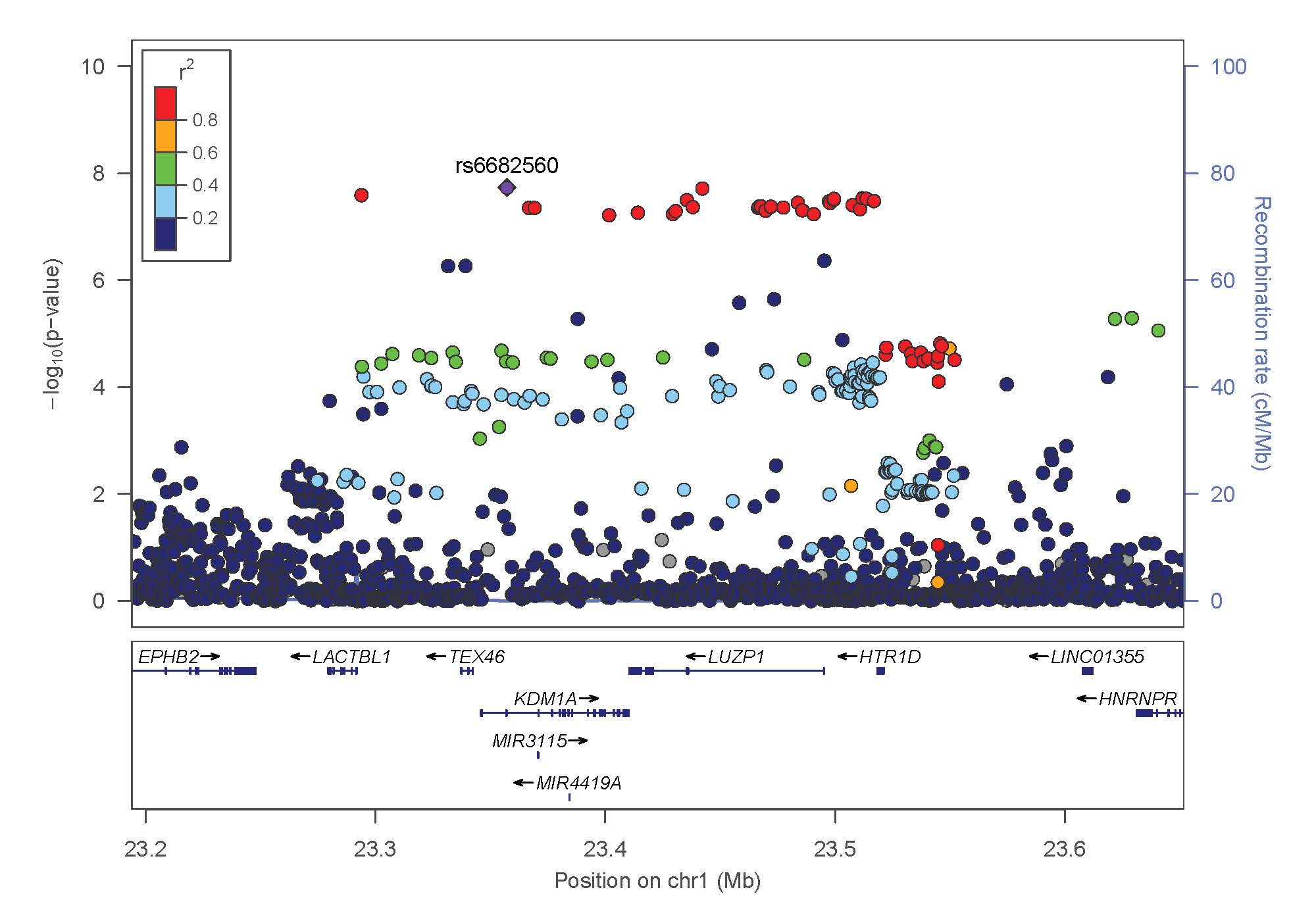

Supplement: Data S3. Regional plots of the identified genetic loci for human head size (±100 kb), related to Figure 1A and 1B [file mmc19.zip › Data S2/rs6682560.jpg]

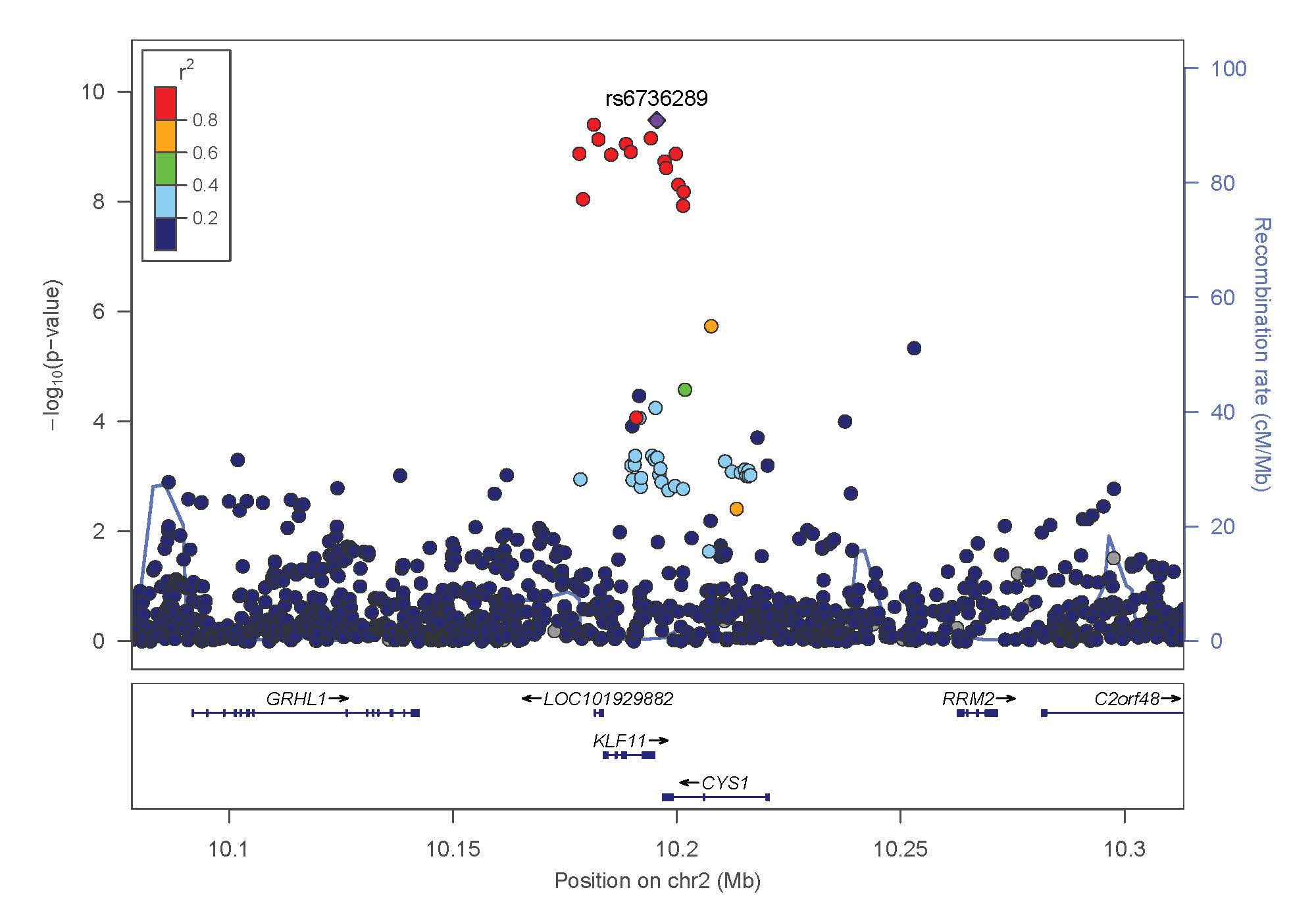

Supplement: Data S3. Regional plots of the identified genetic loci for human head size (±100 kb), related to Figure 1A and 1B [file mmc19.zip › Data S2/rs6736289.jpg]

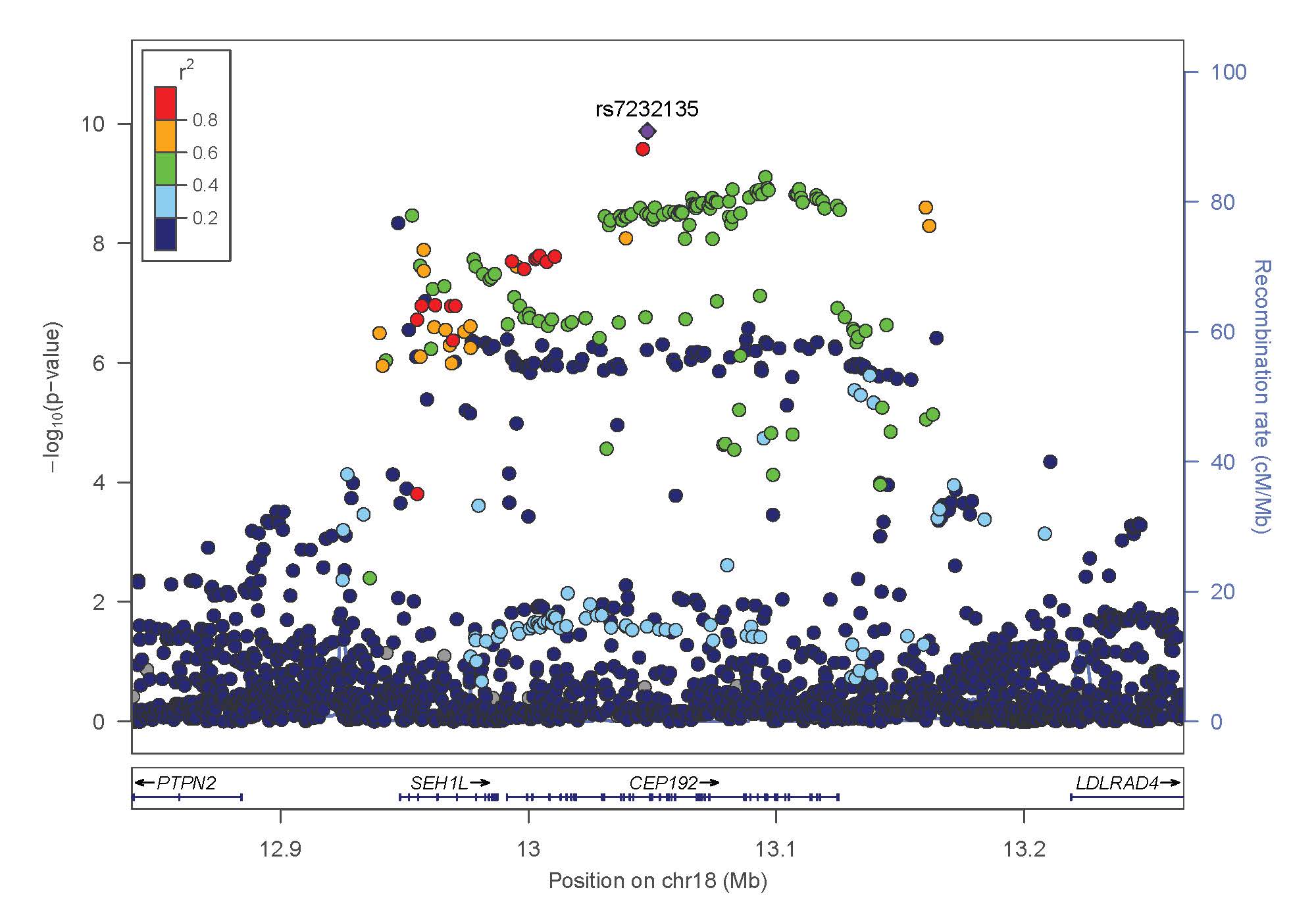

Supplement: Data S3. Regional plots of the identified genetic loci for human head size (±100 kb), related to Figure 1A and 1B [file mmc19.zip › Data S2/rs7232135.jpg]

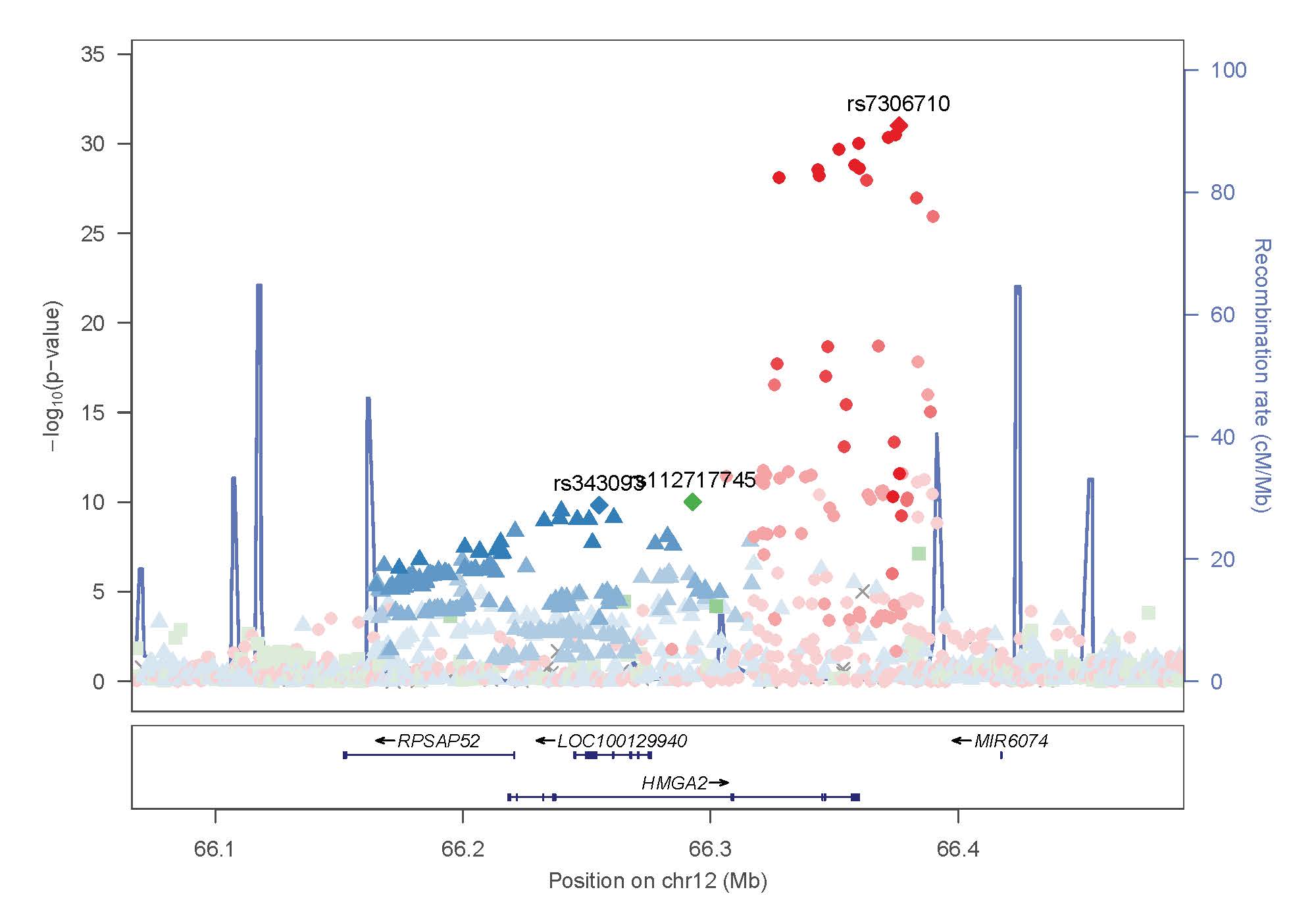

Supplement: Data S3. Regional plots of the identified genetic loci for human head size (±100 kb), related to Figure 1A and 1B [file mmc19.zip › Data S2/rs7306710.jpg]

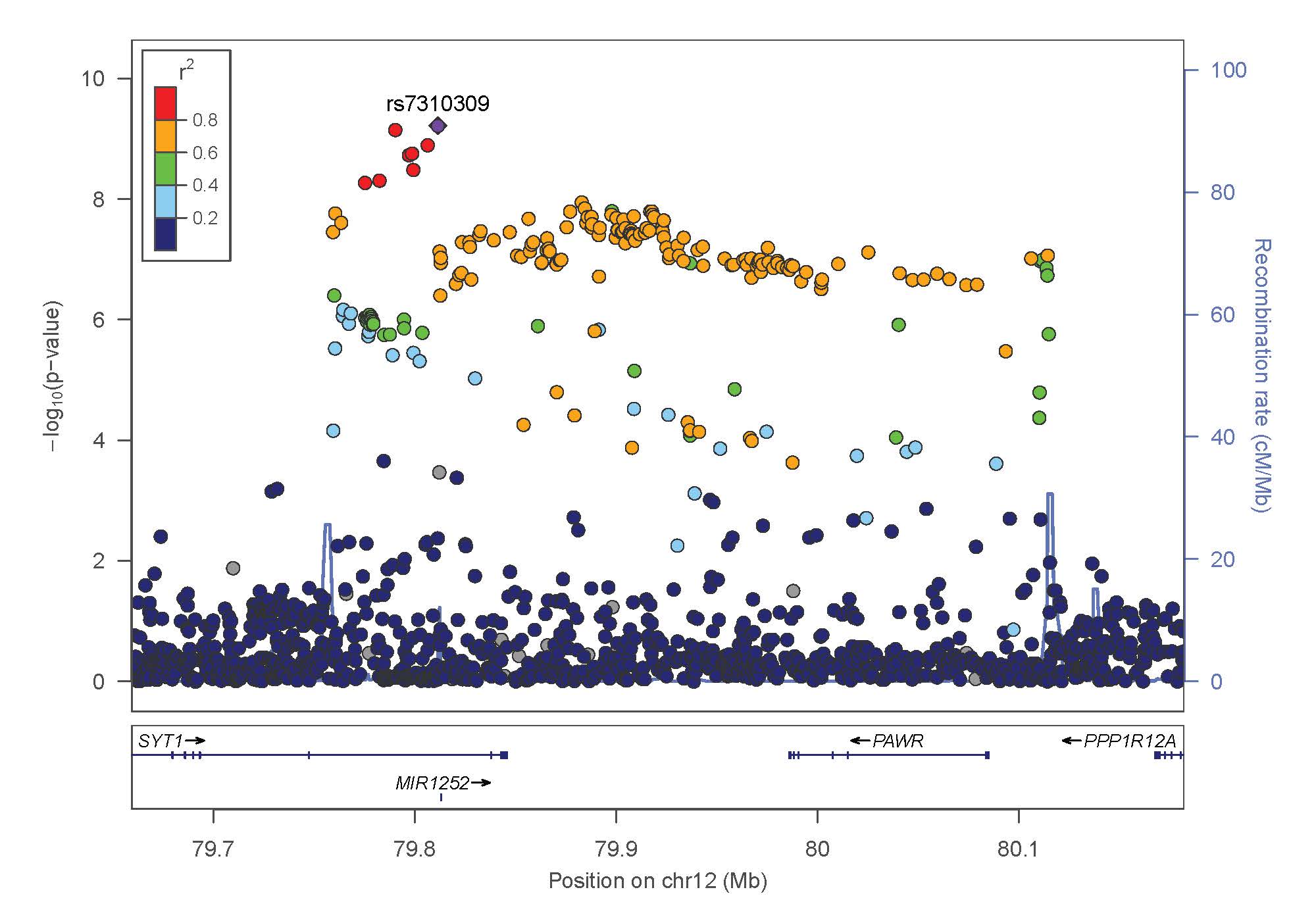

Supplement: Data S3. Regional plots of the identified genetic loci for human head size (±100 kb), related to Figure 1A and 1B [file mmc19.zip › Data S2/rs7310309.jpg]

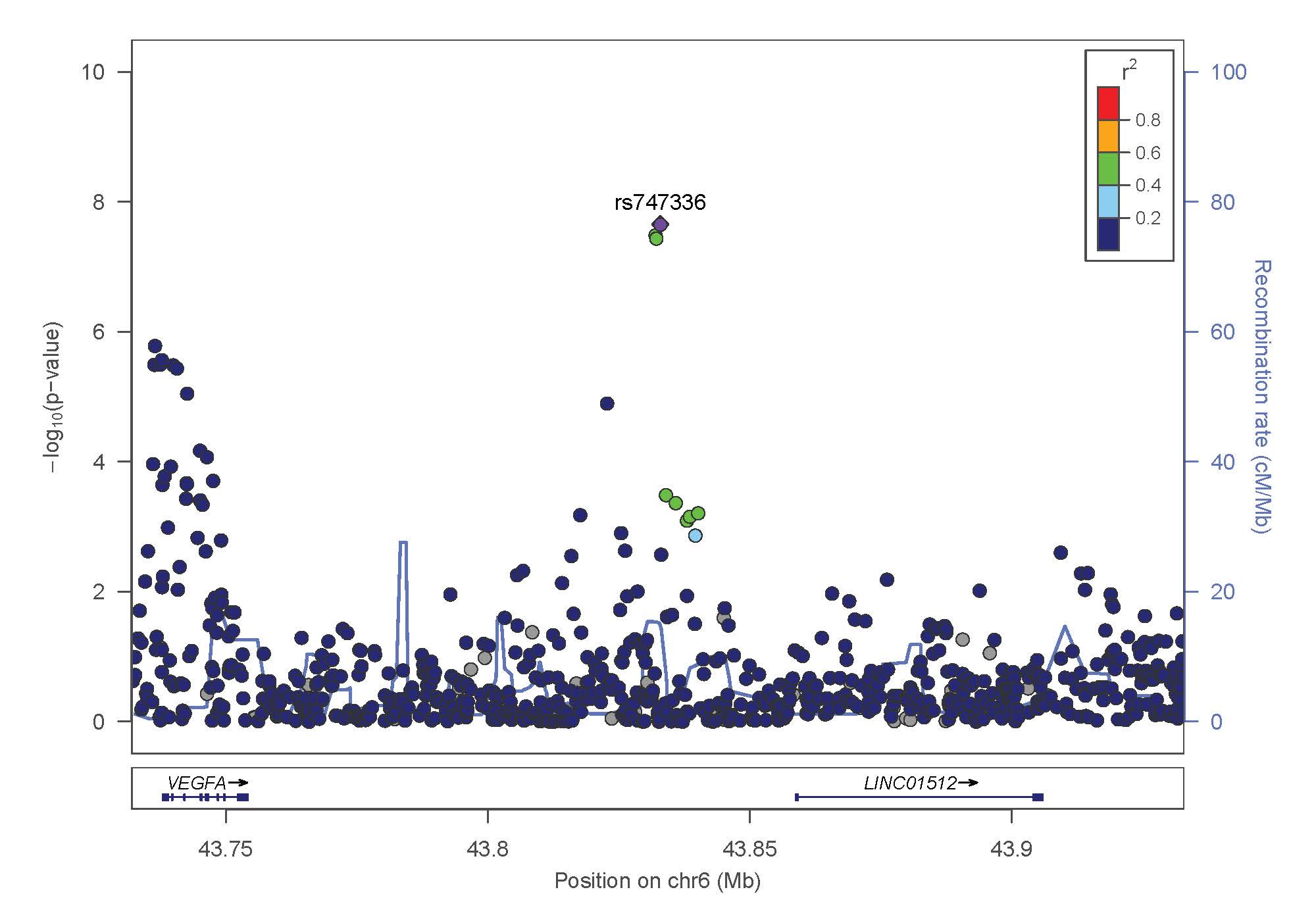

Supplement: Data S3. Regional plots of the identified genetic loci for human head size (±100 kb), related to Figure 1A and 1B [file mmc19.zip › Data S2/rs747336.jpg]

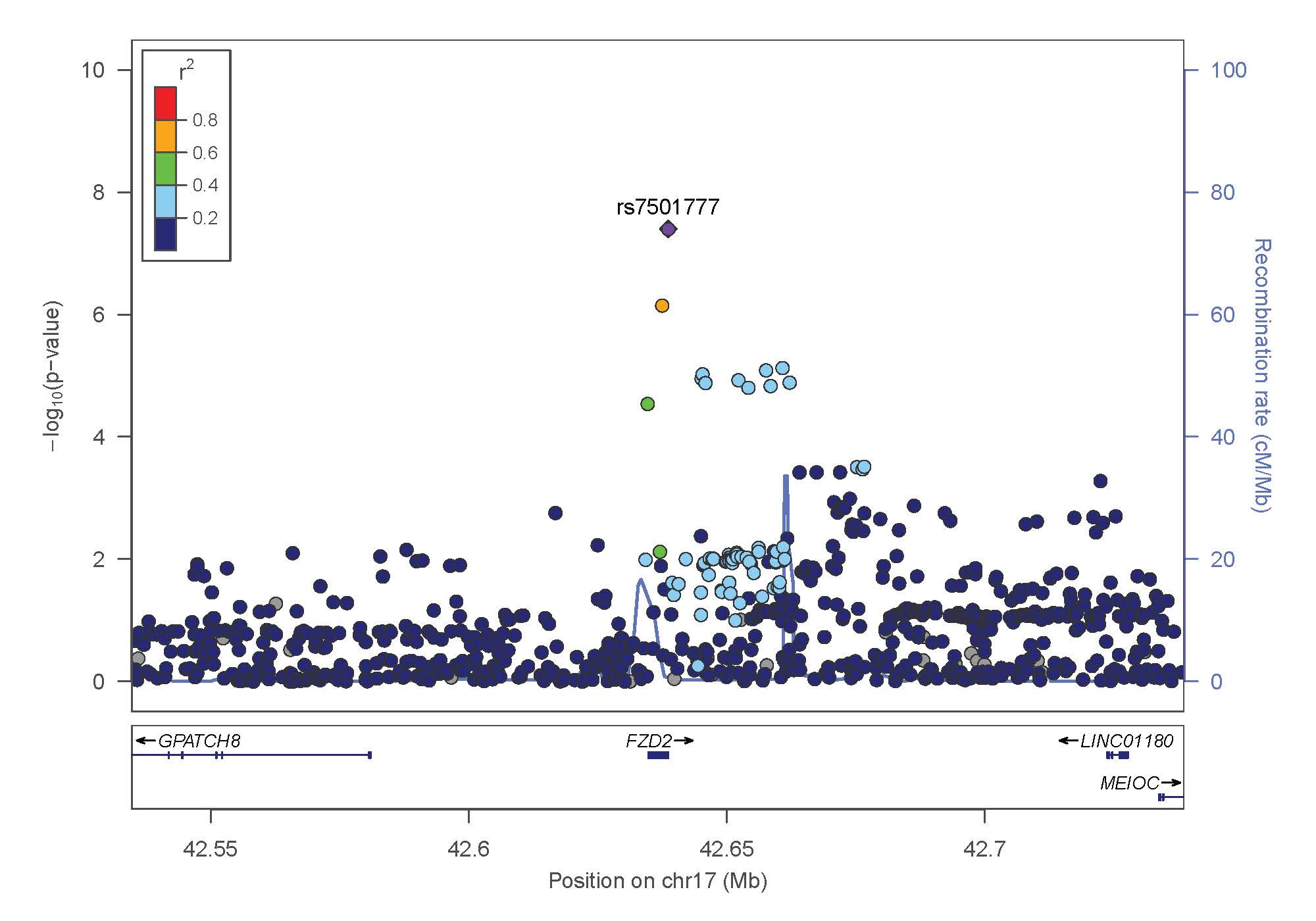

Supplement: Data S3. Regional plots of the identified genetic loci for human head size (±100 kb), related to Figure 1A and 1B [file mmc19.zip › Data S2/rs7501777.jpg]

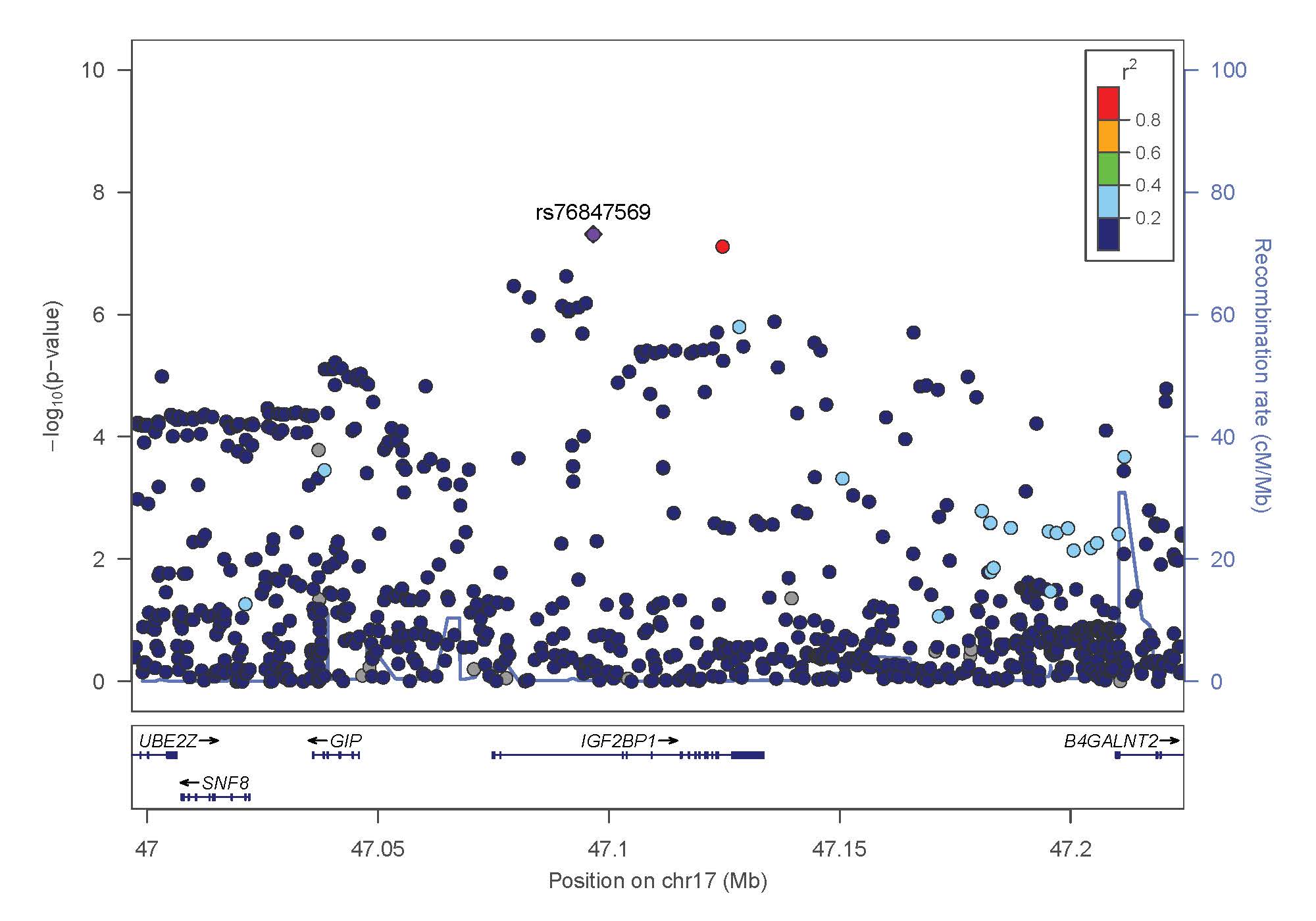

Supplement: Data S3. Regional plots of the identified genetic loci for human head size (±100 kb), related to Figure 1A and 1B [file mmc19.zip › Data S2/rs76847569.jpg]

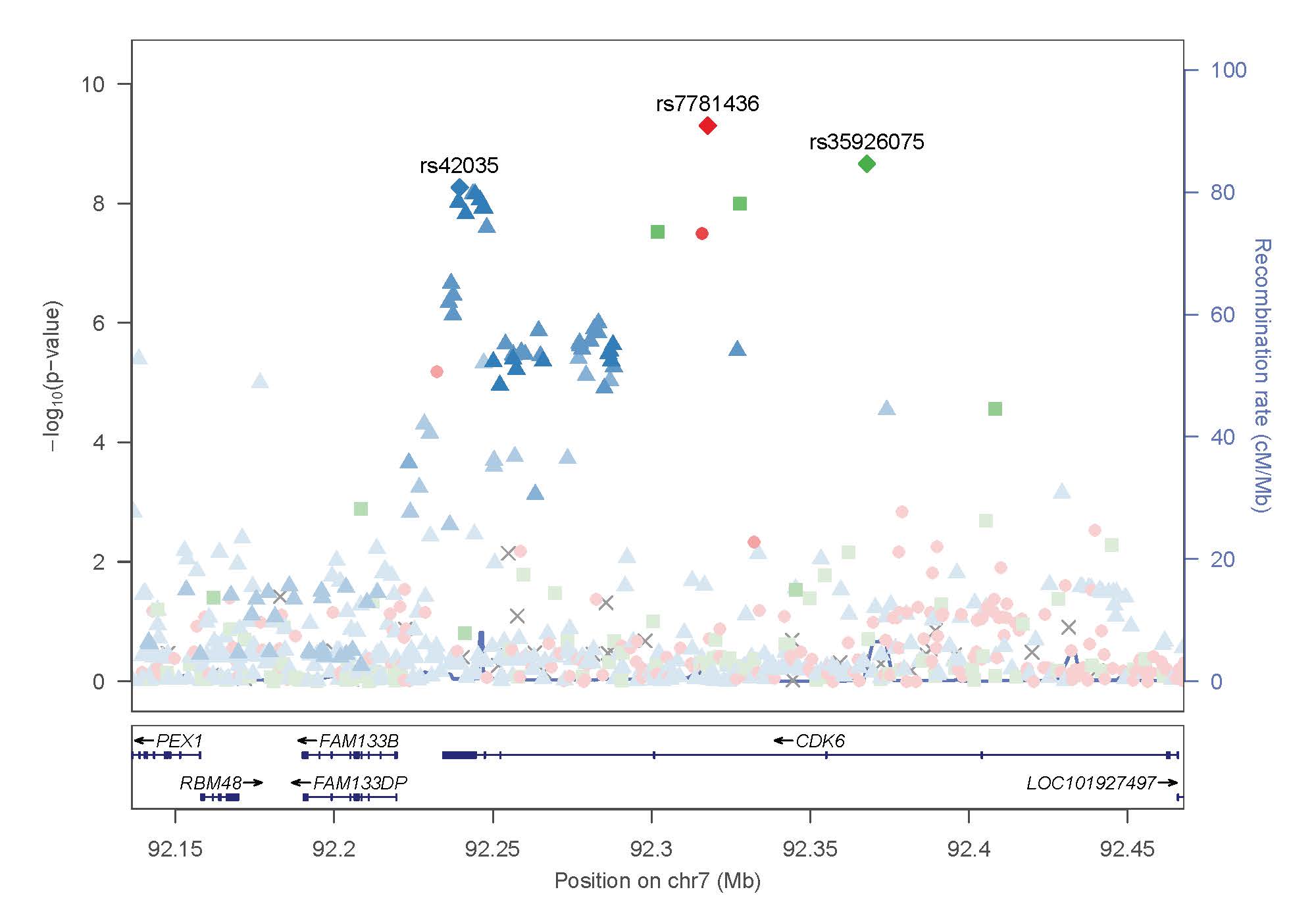

Supplement: Data S3. Regional plots of the identified genetic loci for human head size (±100 kb), related to Figure 1A and 1B [file mmc19.zip › Data S2/rs7781436.jpg]

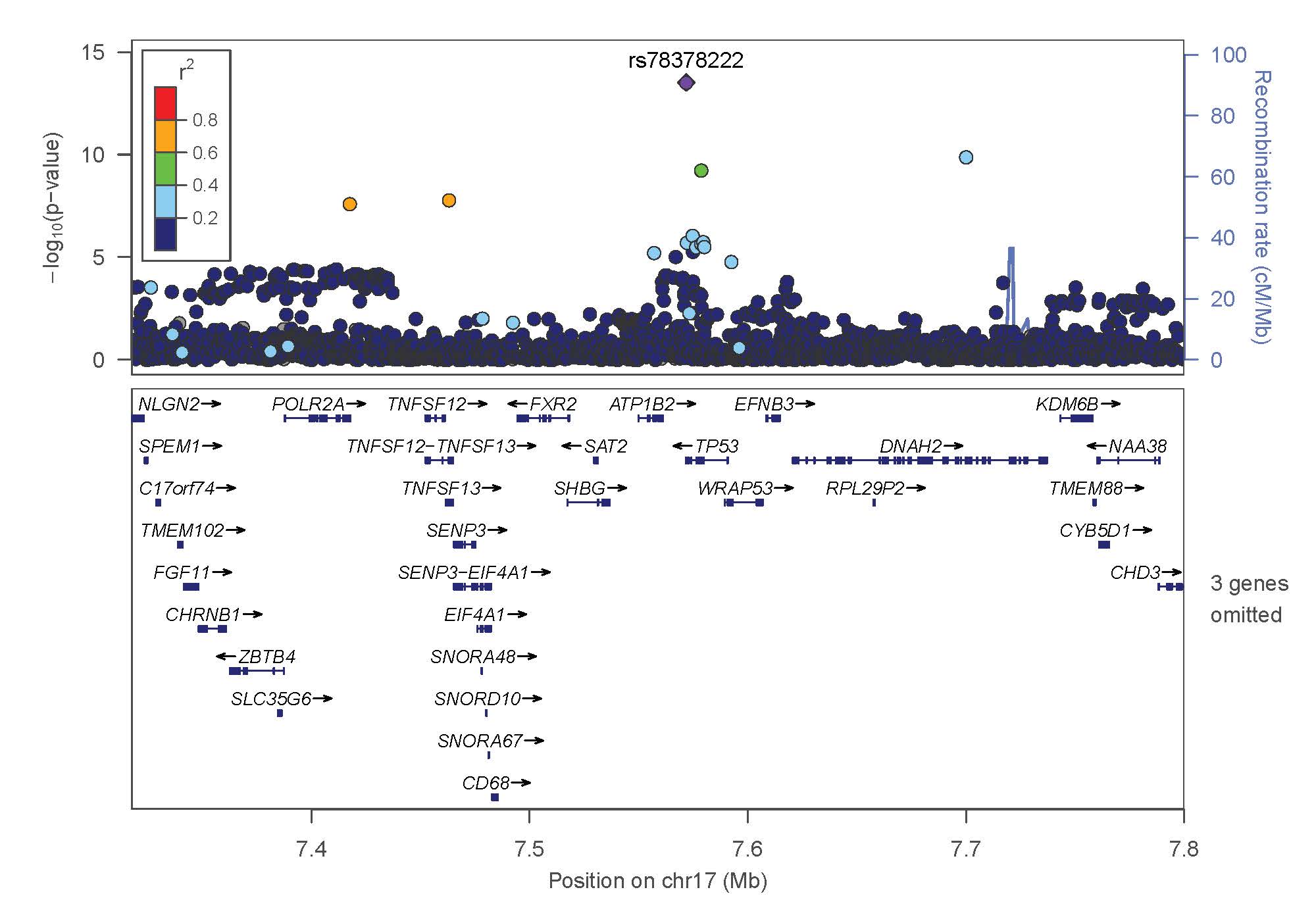

Supplement: Data S3. Regional plots of the identified genetic loci for human head size (±100 kb), related to Figure 1A and 1B [file mmc19.zip › Data S2/rs78378222.jpg]

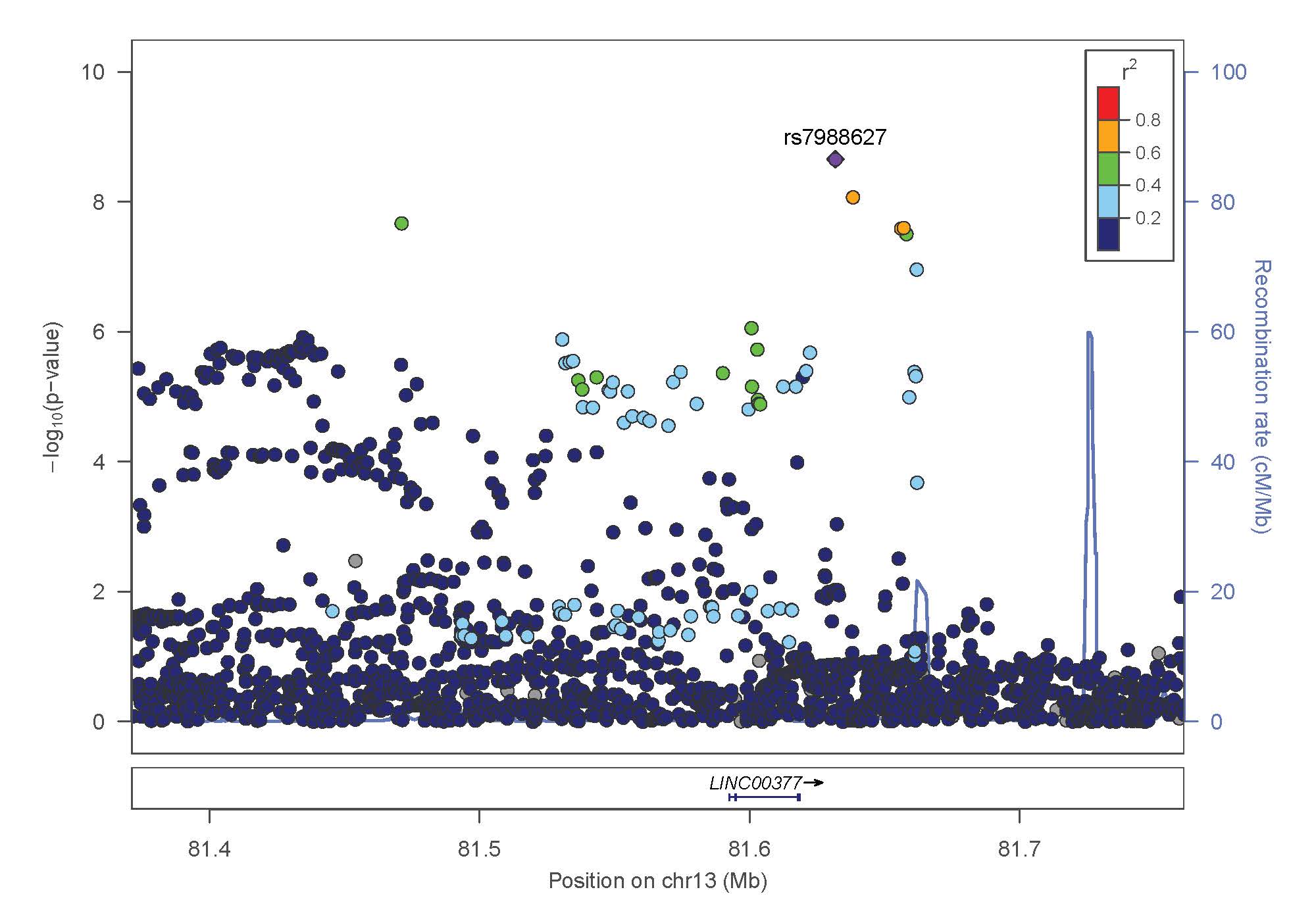

Supplement: Data S3. Regional plots of the identified genetic loci for human head size (±100 kb), related to Figure 1A and 1B [file mmc19.zip › Data S2/rs7988627.jpg]

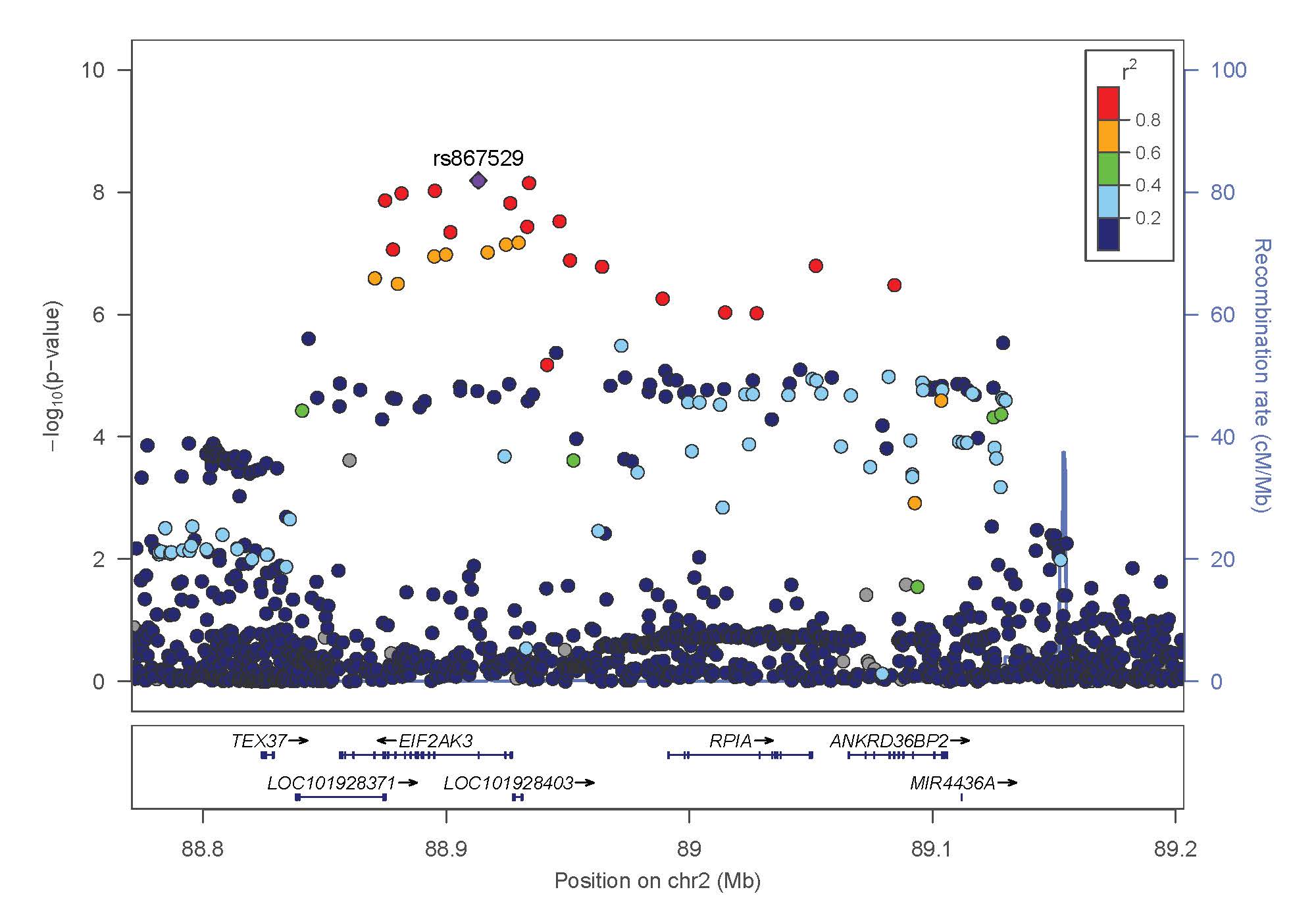

Supplement: Data S3. Regional plots of the identified genetic loci for human head size (±100 kb), related to Figure 1A and 1B [file mmc19.zip › Data S2/rs867529.jpg]

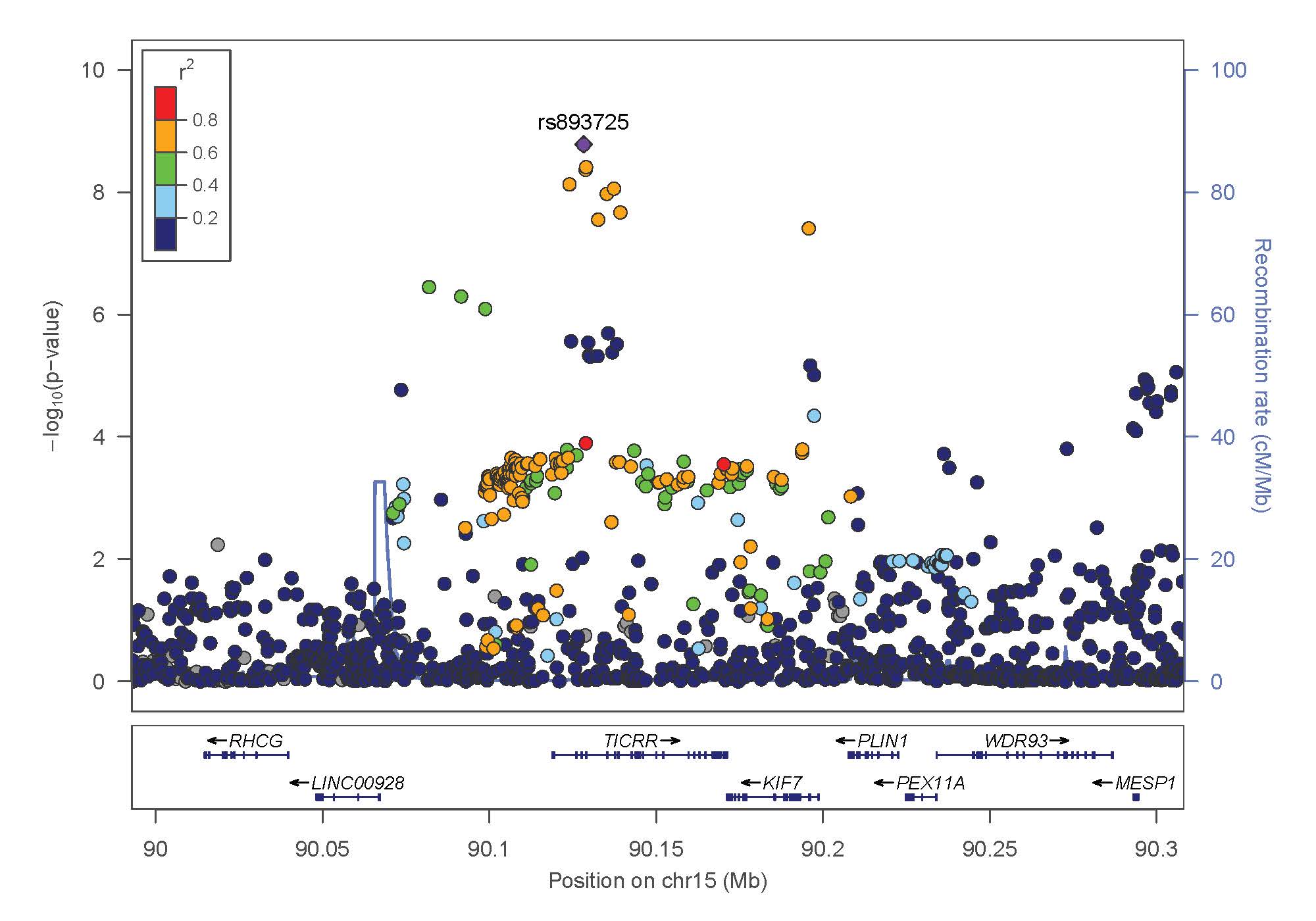

Supplement: Data S3. Regional plots of the identified genetic loci for human head size (±100 kb), related to Figure 1A and 1B [file mmc19.zip › Data S2/rs893725.jpg]

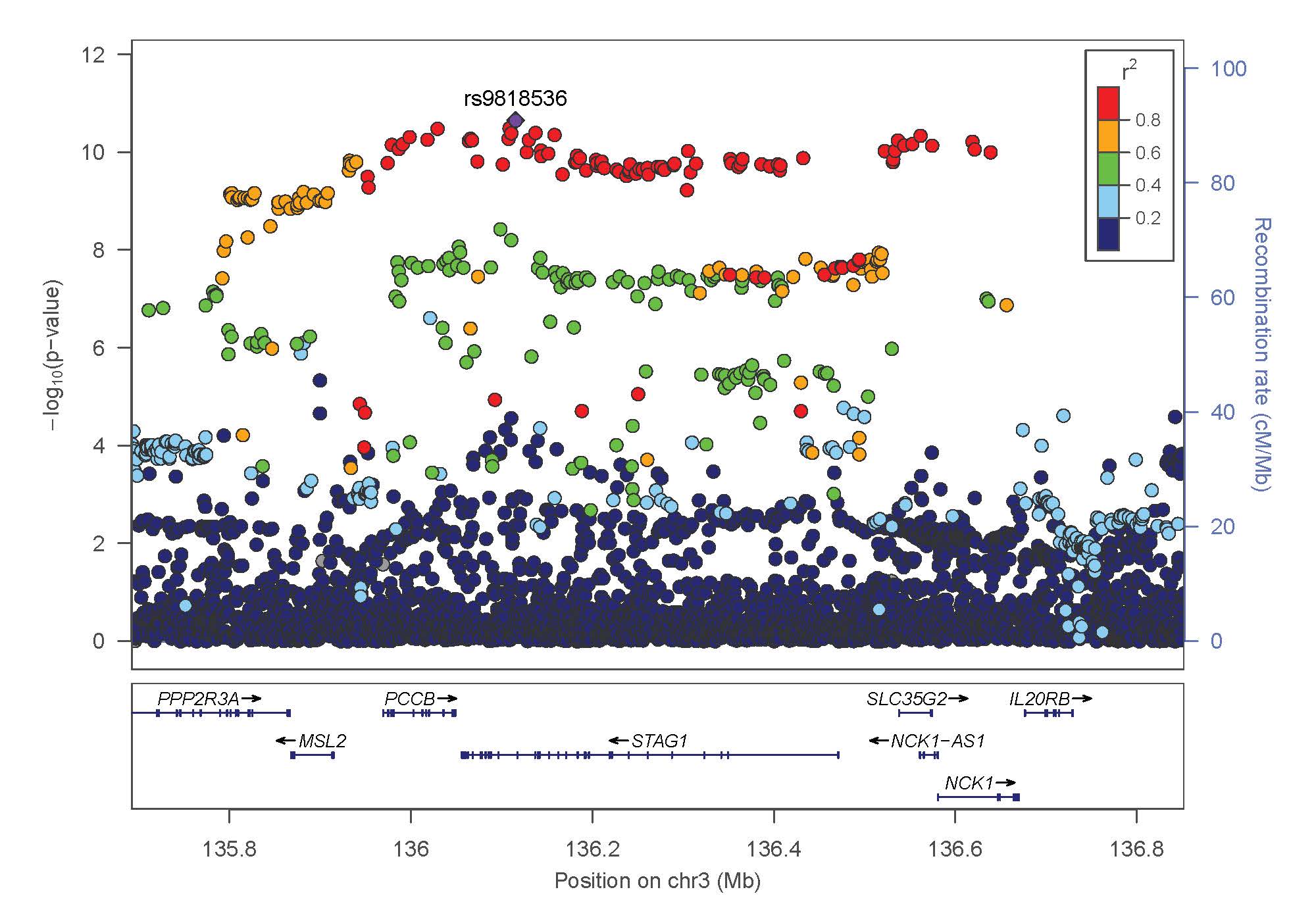

Supplement: Data S3. Regional plots of the identified genetic loci for human head size (±100 kb), related to Figure 1A and 1B [file mmc19.zip › Data S2/rs9818536.jpg]
