# Supplementary material for: Genetic variants for head size share genes and pathways with cancer
Source: Cell Rep Med. 2024 May 3;5(5):101529. doi: 10.1016/j.xcrm.2024.101529 (PMC11148644; doi:10.1016/j.xcrm.2024.101529)
Supplement: Document S2. Article plus supplemental information [file mmc20.pdf]

Cell Reports Medicine

Report

Genetic variants for head size share genes and pathways with cancer

Graphical abstract

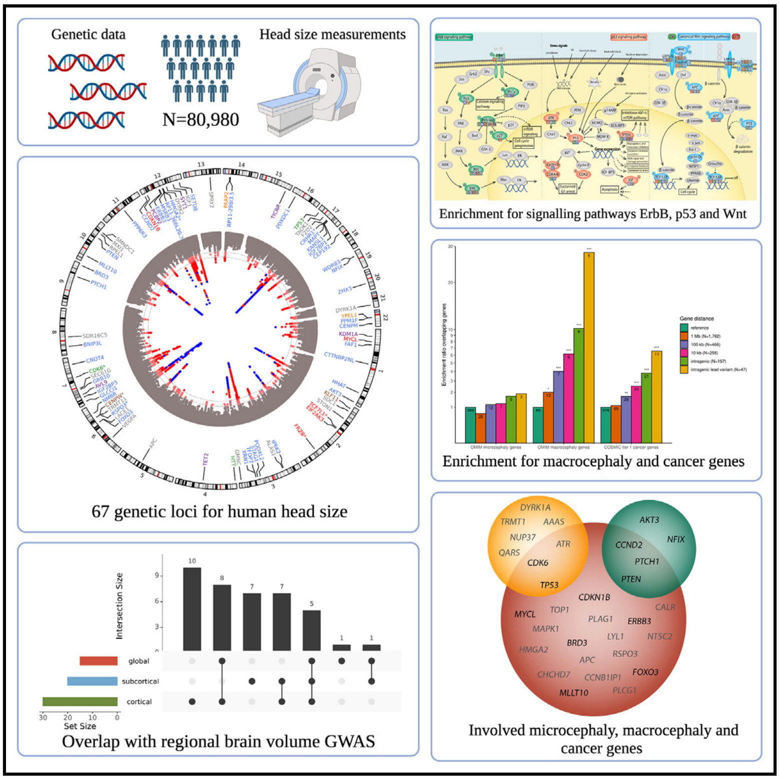

Authors

Maria J. Knol, Raymond A. Poot, Tavia E. Evans, ..., Paul M. Thompson, Sudha Seshadri, Hieab H.H. Adams

Correspondence

hieab.adams@radboudumc.nl

In brief

Knol, Poot, et al. identify 67 loci for human head size in a genome-wide association study. Genes harboring the lead variants enrich for cancer genes and pathways, which was not seen for height variants. These findings suggest a potential link between a larger head and a higher cancer risk.

Highlights

- Knol, Poot, et al. identify 67 genetic loci associated with human head size
- Genes harboring or near head size genetic variants enrich for macrocephaly genes
- Head size genetic variants preferentially locate to cancer genes and pathways
- Further research is needed on the potential link between head size and cancer risk

Knol et al., 2024, Cell Reports Medicine *5*, 101529
May 21, 2024 © 2024 The Author(s). Published by Elsevier Inc.
https://doi.org/10.1016/j.xcrm.2024.101529

CellPress

Cell Reports Medicine

CellPress
OPEN ACCESS

Report

Genetic variants for head size share genes and pathways with cancer

Maria J. Knol,[1,115] Raymond A. Poot,[2,115] Tavia E. Evans,[3,4] Claudia L. Satizabal,[5,6,7] Aniket Mishra,[8] Muralidharan Sargurupremraj,[5] Sandra van der Auwera,[9,10] Marie-Gabrielle Duperron,[8] Xueqiu Jian,[11] Isabel C. Hostettler,[12,13,14] Dianne H.K. van Dam-Nolen,[4] Sander Lamballais,[3] Mikolaj A. Pawlak,[15,16]

(Author list continued on next page)

[1]Department of Epidemiology, Erasmus MC University Medical Center, Rotterdam, the Netherlands
[2]Department of Cell Biology, Erasmus MC University Medical Center, Rotterdam, the Netherlands
[3]Department of Clinical Genetics, Erasmus MC University Medical Center, Rotterdam, the Netherlands
[4]Department of Radiology and Nuclear Medicine, Erasmus MC University Medical Center, Rotterdam, the Netherlands
[5]Glenn Biggs Institute for Alzheimer's & Neurodegenerative Diseases, UT Health San Antonio, San Antonio, TX, USA
[6]The Framingham Heart Study, Framingham, MA, USA
[7]Department of Neurology, Boston University School of Medicine, Boston, MA, USA
[8]University of Bordeaux, Inserm, Bordeaux Population Health Research Center, team VINTAGE, UMR 1219, Bordeaux, France
[9]Department of Psychiatry and Psychotherapy, University Medicine Greifswald, Greifswald, Germany
[10]German Centre of Neurodegenerative Diseases (DZNE), Site Rostock/Greifswald, Greifswald, Germany
[11]Brown Foundation Institute of Molecular Medicine, McGovern Medical School, University of Texas Health Science Center at Houston, Houston, TX, USA
[12]Stroke Research Centre, University College London, Institute of Neurology, London, UK
[13]Department of Neurosurgery, Klinikum rechts der Isar, University of Munich, Munich, Germany
[14]Neurosurgical Department, Cantonal Hospital St. Gallen, St. Gallen, Switzerland
[15]Department of Neurology, Poznań University of Medical Sciences, Poznań, Poland
[16]Department of Human Genetics, Radboud University Medical Center, Nijmegen, the Netherlands
[17]Department of Epidemiology, School of Public Health, University of Alabama at Birmingham School of Medicine, Birmingham, AL, USA
[18]Language and Genetics Department, Max Planck Institute for Psycholinguistics, Nijmegen, the Netherlands
[19]Clinical Translational Neuroscience Laboratory, Department of Psychiatry and Human Behavior, University of California, Irvine, Irvine, CA, USA
[20]Center for the Neurobiology of Learning and Memory, University of California, Irvine, Irvine, CA, USA
[21]Department of Biomedicine, University of Basel, Basel, Switzerland
[22]Institute of Medical Genetics and Pathology, University Hospital Basel, Basel, Switzerland
[23]Institute of Computational Life Sciences, Zurich University of Applied Sciences, Wädenswil, Switzerland

(Affiliations continued on next page)

SUMMARY

The size of the human head is highly heritable, but genetic drivers of its variation within the general population remain unmapped. We perform a genome-wide association study on head size (N = 80,890) and identify 67 genetic loci, of which 50 are novel. Neuroimaging studies show that 17 variants affect specific brain areas, but most have widespread effects. Gene set enrichment is observed for various cancers and the p53, Wnt, and ErbB signaling pathways. Genes harboring lead variants are enriched for macrocephaly syndrome genes (37-fold) and high-fidelity cancer genes (9-fold), which is not seen for human height variants. Head size variants are also near genes preferentially expressed in intermediate progenitor cells, neural cells linked to evolutionary brain expansion. Our results indicate that genes regulating early brain and cranial growth incline to neoplasia later in life, irrespective of height. This warrants investigation of clinical implications of the link between head size and cancer.

INTRODUCTION

The size of the human head, measured by head circumference or intracranial volume, correlates closely with brain size. Head size is determined by growth in the first years of life and is largely completed by 6 years of age, whereas the rest of the body typically grows until early adulthood.[1] Head size is highly genetically determined, ranging from near 90% during childhood to 75% during

© 2024 The Author(s). Published by Elsevier Inc.
This is an open access article under the CC BY license (http://creativecommons.org/licenses/by/4.0/).

Cora E. Lewis,[17] Amaia Carrion-Castillo,[18] Theo G.M. van Erp,[19,20] Céline S. Reinbold,[21,22,23] Jean Shin,[24,25] Markus Scholz,[26,27] Asta K. Håberg,[28,29] Anders Kämpe,[30,31] Gloria H.Y. Li,[32] Reut Avinun,[33] Joshua R. Atkins,[34,35] Fang-Chi Hsu,[36] Alyssa R. Amod,[37] Max Lam,[38,39] Ami Tsuchida,[8,40] Mariël W.A. Teunissen,[16,41] Nil Aygün,[42] Yash Patel,[43] Dan Liang,[42] Alexa S. Beiser,[6,7,44] Frauke Beyer,[45,46,47] Joshua C. Bis,[48] Daniel Bos,[1,4] R. Nick Bryan,[49] Robin Bülow,[50] Svenja Caspers,[51,52] Gwenaëlle Catheline,[53,54] Charlotte A.M. Cecil,[1,55] Shareefa Dalvie,[37] Jean-François Dartigues,[56] Charles DeCarli,[57] Maria Enlund-Cerullo,[58,59] Judith M. Ford,[60,61] Barbara Franke,[16,62,63] Barry I. Freedman,[64] Nele Friedrich,[65] Melissa J. Green,[66,67] Simon Haworth,[68] Catherine Helmer,[69] Per Hoffmann,[21,22,70] Georg Homuth,[71] M. Kamran Ikram,[1,72] Clifford R. Jack, Jr.,[73] Neda Jahanshad,[74] Christiane Jockwitz,[51,75] Yoichiro Kamatani,[76] Annchen R. Knodt,[33] Shuo Li,[44] Keane Lim,[77] W.T. Longstreth,[78,79] Fabio Macciardi,[80] The Cohorts for Heart and Aging

(Author list continued on next page)

[24]The Hospital for Sick Children, University of Toronto, Toronto, Canada
[25]Departments of Physiology and Nutritional Sciences, University of Toronto, Toronto, Canada
[26]Institute for Medical Informatics, Statistics and Epidemiology, University of Leipzig, Leipzig, Germany
[27]LIFE Research Center for Civilization Disease, Leipzig, Germany
[28]Department of Neuromedicine and Movement Science, Norwegian University of Science and Technology (NTNU), Trondheim, Norway
[29]Department of Radiology and Nuclear Medicine, St. Olavs University Hospital, Trondheim, Norway
[30]Department of Molecular Medicine and Surgery, Karolinska Institutet, Stockholm, Sweden
[31]Department of Clinical Genetics, Karolinska University Hospital, Stockholm, Sweden
[32]Department of Pharmacology and Pharmacy, Li Ka Shing Faculty of Medicine, The University of Hong Kong, Hong Kong, China
[33]Laboratory of NeuroGenetics, Department of Psychology & Neuroscience, Duke University, Durham, NC, USA
[34]School of Biomedical Sciences and Pharmacy, The University of Newcastle, Callaghan, NSW, Australia
[35]Centre for Brain and Mental Health Research, Hunter Medical Research Institute, Newcastle, NSW, Australia
[36]Department of Biostatistics and Data Science, Wake Forest University School of Medicine, Winston-Salem, NC, USA
[37]Department of Child and Adolescent Psychiatry, TU Dresden, Dresden, Germany
[38]North Region, Institute of Mental Health, Singapore, Singapore
[39]Population and Global Health, LKC Medicine, Nanyang Technological University, Singapore, Singapore
[40]Groupe d'imagerie neurofonctionnelle, Institut des Maladies Neurodégénératives, UMR 5293, CNRS, CEA, Université de Bordeaux, Bordeaux, France
[41]Department of Neurology, Maastricht University Medical Center+, Maastricht, the Netherlands
[42]Department of Genetics UNC Neuroscience Center, University of North Carolina at Chapel Hill, Chapel Hill, NC, USA
[43]Institute of Medical Sciences, University of Toronto, Toronto, ON, Canada
[44]Department of Biostatistics, Boston University School of Public Health, Boston, MA, USA
[45]Department of Neurology, Max Planck Institute for Cognitive and Brain Sciences, Leipzig, Germany
[46]Collaborative Research Center 1052 Obesity Mechanisms, Faculty of Medicine, University of Leipzig, Leipzig, Germany
[47]Day Clinic for Cognitive Neurology, University Hospital Leipzig, Leipzig, Germany
[48]Cardiovascular Health Research Unit, Department of Medicine, University of Washington, Seattle, WA, USA
[49]Department of Radiology, University of Pennsylvania, Philadelphia, PA, USA
[50]Institute of Diagnostic Radiology and Neuroradiology, University Medicine Greifswald, Greifswald, Germany
[51]Institute of Neuroscience and Medicine (INM-1), Research Centre Jülich, Jülich, Germany
[52]Institute for Anatomy I, Medical Faculty & University Hospital Düsseldorf, Heinrich Heine University Düsseldorf, Düsseldorf, Germany
[53]University of Bordeaux, CNRS, INCIA, UMR 5287, team NeuroImagerie et Cognition Humaine, Bordeaux, France
[54]EPHE-PSL University, Bordeaux, France
[55]Department of Child and Adolescent Psychiatry, Erasmus MC University Medical Center, Rotterdam, the Netherlands
[56]University of Bordeaux, Inserm, Bordeaux Population Health Research Center, team SEPIA, UMR 1219, Bordeaux, France
[57]Department of Neurology and Center for Neuroscience, University of California at Davis, Sacramento, CA, USA
[58]Children's Hospital, University of Helsinki and Helsinki University Hospital, Helsinki, Finland
[59]Folkhälsan Research Center, Helsinki, Finland
[60]San Francisco Veterans Administration Medical Center, San Francisco, CA, USA
[61]University of California, San Francisco, San Francisco, CA, USA
[62]Department of Psychiatry, Radboud University Medical Center, Nijmegen, the Netherlands
[63]Donders Institute for Brain, Cognition, and Behaviour, Radboud University, Nijmegen, the Netherlands
[64]Department of Internal Medicine, Section on Nephrology, Wake Forest School of Medicine, Winston-Salem, NC, USA
[65]Institute of Clinical Chemistry and Laboratory Medicine, University Medicine Greifswald, Greifswald, Germany
[66]School of Clinical Medicine, University of New South Wales, Sydney, NSW, Australia
[67]Neuroscience Research Australia, Sydney, NSW, Australia
[68]Bristol Dental School, University of Bristol, Bristol, UK
[69]University of Bordeaux, Inserm, Bordeaux Population Health Research Center, team LEHA, UMR 1219, Bordeaux, France
[70]Institute of Human Genetics, University of Bonn Medical School, Bonn, Germany
[71]Interfaculty Institute for Genetics and Functional Genomics, University Medicine Greifswald, Greifswald, Germany

(Affiliations continued on next page)

CellPress
OPEN ACCESS

Research in Genomic Epidemiology (CHARGE) Consortium, The Enhancing NeuroImaging Genetics through Meta-Analysis (ENIGMA) Consortium, Outi Mäkitie,[30,31,58,59] Bernard Mazoyer,[40,81] Sarah E. Medland,[82,83,84] Susumu Miyamoto,[85] Susanne Moebus,[86] Thomas H. Mosley,[87,88] Ryan Muetzel,[1,55] Thomas W. Mühleisen,[21,51,89] Manabu Nagata,[85] Soichiro Nakahara,[19,90] Nicholette D. Palmer,[91] Zdenka Pausova,[24,25] Adrian Preda,[92] Yann Quidé,[66,67] William R. Reay,[34,35] Gennady V. Roshchupkin,[1,4] Reinhold Schmidt,[93] Pamela J. Schreiner,[94] Kazuya Setoh,[76] Chin Yang Shapland,[18,95,96] Stephen Sidney,[97] Beate St Pourcain,[18,63,95] Jason L. Stein,[42] Yasuharu Tabara,[76] Alexander Teumer,[9,98] Anne Uhlmann,[37] Aad van der Lugt,[4] Meike W. Vernooij,[1,4] David J. Werring,[12]

(Author list continued on next page)

[72]Department of Neurology, Erasmus MC University Medical Center, Rotterdam, the Netherlands
[73]Department of Radiology, Mayo Clinic, Rochester, MN, USA
[74]Imaging Genetics Center, Mark & Mary Stevens Neuroimaging & Informatics Institute, Keck USC School of Medicine, Los Angeles, CA, USA
[75]Department of Psychiatry, Psychotherapy and Psychosomatics, RWTH Aachen University, Medical Faculty, Aachen, Germany
[76]Center for Genomic Medicine, Kyoto University Graduate School of Medicine, Kyoto, Japan
[77]Research Division, Institute of Mental Health, Singapore, Singapore
[78]Department of Neurology, University of Washington, Seattle, WA, USA
[79]Department of Epidemiology, University of Washington, Seattle, WA, USA
[80]Laboratory of Molecular Psychiatry, Department of Psychiatry and Human Behavior, School of Medicine, University of California, Irvine, Irvine, CA, USA
[81]Centre Hospitalo-Universitaire de Bordeaux, Bordeaux, France
[82]Psychiatric Genetics, QIMR Berghofer Medical Research Institute, Brisbane, QLD, Australia
[83]School of Psychology, University of Queensland, Brisbane, QLD, Australia
[84]Faculty of Medicine, University of Queensland, Brisbane, QLD, Australia
[85]Department of Neurosurgery, Kyoto University Graduate School of Medicine, Kyoto, Japan
[86]Institute for Urban Public Health, University of Duisburg-Essen, Essen, Germany
[87]Department of Medicine, Division of Geriatrics, University of Mississippi Medical Center, Jackson, MS, USA
[88]Memory Impairment and Neurodegenerative Dementia (MIND) Center, Jackson, MS, USA
[89]C. and O. Vogt Institute for Brain Research, Medical Faculty, Heinrich Heine University Düsseldorf, Düsseldorf, Germany
[90]Unit 2, Candidate Discovery Science Labs, Drug Discovery Research, Astellas Pharma Inc, 21 Miyukigaoka, Tsukuba, Ibaraki 305-8585, Japan
[91]Department of Biochemistry, Wake Forest School of Medicine, Winston-Salem, NC, USA
[92]Department of Psychiatry, University of California, Irvine, Irvine, CA, USA
[93]Clinical Division of Neurogeriatrics, Department of Neurology, Medical University of Graz, Graz, Austria
[94]University of Minnesota School of Public Health, Minneapolis, MN, USA
[95]MRC Integrative Epidemiology Unit, University of Bristol, Bristol, UK
[96]Population Health Sciences, University of Bristol, Bristol, UK
[97]Kaiser Permanente Division of Research, Oakland, CA, USA
[98]Institute for Community Medicine, University Medicine Greifswald, Greifswald, Germany
[99]Department of Human Genetics, Donders Institute for Brain, Cognition, and Behaviour, Radboud University Medical Center, Nijmegen, the Netherlands
[100]Department of Clinical Genetics MUMC+, GROW School of Oncology and Developmental Biology, and MHeNs School of Mental Health and Neuroscience, Maastricht University, Maastricht, the Netherlands
[101]Bordeaux Population Health, University of Bordeaux, INSERM U1219, Bordeaux, France
[102]West Region, Institute of Mental Health, Singapore, Singapore
[103]Yong Loo Lin School of Medicine, National University of Singapore, Singapore, Singapore
[104]Lee Kong Chian School of Medicine, Nanyang Technological University, Singapore, Singapore
[105]SAMRC Unit on Risk and Resilience, University of Cape Town, Cape Town, South Africa
[106]Centre for Genomic Sciences, Li Ka Shing Faculty of Medicine, The University of Hong Kong, Hong Kong, China
[107]Department of Medicine, Li Ka Shing Faculty of Medicine, The University of Hong Kong, Hong Kong, China
[108]Departments of Psychiatry and Neuroscience, Faculty of Medicine and Centre Hospitalier Universitaire Sainte-Justine, University of Montreal, Montreal, QC, Canada
[109]Department of Psychiatry, Faculty of Medicine, McGill University, Montreal, QC, Canada
[110]Tri-institutional Center for Translational Research in Neuroimaging and Data Science (TReNDS) {Georgia State, Georgia Tech, Emory}, Atlanta, GA, USA
[111]Laboratory of Epidemiology, Demography, and Biometry, Intramural Research Program, National Institute of Aging, The National Institutes of Health, Bethesda, MD, USA
[112]Human Genetics Center, School of Public Health, University of Texas Health Science Center at Houston, Houston, TX, USA
[113]Department of Neurology, Bordeaux University Hospital, Bordeaux, France
[114]Latin American Brain Health (BrainLat), Universidad Adolfo Ibáñez, Santiago, Chile
[115]These authors contributed equally

(Affiliations continued on next page)

B. Gwen Windham,[87,88] A. Veronica Witte,[45,46,47] Katharina Wittfeld,[9,10] Qiong Yang,[44] Kazumichi Yoshida,[85] Han G. Brunner,[99,100] Quentin Le Grand,[101] Kang Sim,[102,103,104] Dan J. Stein,[37,105] Donald W. Bowden,[91] Murray J. Cairns,[34,35] Ahmad R. Hariri,[33] Ching-Lung Cheung,[32,106,107] Sture Andersson,[58] Arno Villringer,[45,47] Tomas Paus,[108,109] Sven Cichon,[21,22,51] Vince D. Calhoun,[110] Fabrice Crivello,[40] Lenore J. Launer,[111] Tonya White,[4,55] Peter J. Koudstaal,[72] Henry Houlden,[12] Myriam Fornage,[11,112] Fumihiko Matsuda,[76] Hans J. Grabe,[9] M. Arfan Ikram,[1] Stéphanie Debette,[101,113] Paul M. Thompson,[74,116] Sudha Seshadri,[5,6,7,116] and Hieab H.H. Adams[16,114,116,117,*]

[116]These authors contributed equally
[117]Lead contact
*Correspondence: hieab.adams@radboudumc.nl
https://doi.org/10.1016/j.xcrm.2024.101529

adulthood.[2] Rare genetic syndromes have revealed individual genes strongly affecting head size.[3] Nevertheless, genetic determinants of its variation within the general population are still poorly characterized, with no coherent and well-supported picture of associated biological pathways.

A previous genome-wide association study (GWAS) on 47,000 individuals identified 18 genetic loci for intracranial volume,[4] while another GWAS on head size in 46,000 children and adults identified 17 loci for head size including low-frequency variants in *TP53*.[5] Here, we increased the sample size to a total GWAS discovery sample size of 80,890 individuals, and validated the results in an independent sample of 25,088 individuals. Our GWAS analyses show strong enrichment for genes and multiple pathways involved in cancer, macrocephaly genes, and show preferential expression of genes near variants in intermediate progenitor cells.

RESULTS

We performed a meta-analysis of GWASs for head size, as proxied by intracranial volume from brain imaging, or head circumference (Tables S1–S3 and S4; STAR Methods). Compared with previous efforts,[5,6] we nearly doubled the sample size (N = 80,890), in majority from European ancestry (N = 75,309). We identified 90 independent genetic variants in 67 loci associated with human head size in the European sample (Figure 1A; Tables S6–S8; Data S1, S2, and S3), of which 50 loci were novel. Although the results showed some bias (linkage disequilibrium [LD] score regression intercept 1.056; Table S5), the identified variants remained genome-wide significant after correction for this amount of bias. Most variants (N = 48) showed consistent directions of association among the European, African (N = 1,356), and Asian (N = 4,225) ancestry samples (Figure 1B; Table S6), suggesting population-specific genetic effects on head size in these loci. Since we had limited non-European samples, we also tested the combined effect of the lead variants, which showed positive associations in African and East Asian ancestry samples ($\beta_{African}$ = 0.34, confidence interval [CI] 0.08–0.60; $\beta_{East\ Asian}$ = 0.40, CI 0.24–0.57). In the European validation sample (N = 25,088), 20 of the 89 lead variants were associated with head size at a Bonferroni significance level ($p < 5.6 \times 10^{-4}$) and 54 at a nominal significance level, while all lead variants showed the same direction of effect. In the UK Biobank validation sample (N = 23,046), the 89 available lead variants together explained 2.3% of the phenotypic head size variance. A meta-analysis combining the European discovery and validation sample (N = 101,241) identified 102 genomic loci with 126 lead variants (Table S8), of which 60 loci overlapped with the 67 genomic loci identified by the discovery meta-analysis.

Head-specific growth vs. general growth

We investigated whether variants affecting head size are specific for growth of the human head or are driven, at least in part, by an effect on human body height. Accordingly, we performed a height-adjusted head size GWAS (N = 50,424). The genetic correlation between head size and height ($\rho_{genetic}$ = 0.26, $p = 2.1 \times 10^{-30}$) disappeared in this model ($\rho_{genetic} = -0.02$, p = 0.58) (Figure 1C), confirming the removal of height-associated effects. Importantly, there was no significant reduction for any of the lead variants' effect sizes with head size (Table S6). We further explored the effect of these variants on the size of other body parts using area measures obtained from bone density scans (N = 3,313). As expected, a polygenic score of the lead variants was associated with the skull area, even after adjusting for height ($p = 2.1 \times 10^{-12}$). One lead genetic variant (rs12277225) was significantly associated with the L1-L4 spine area ($p = 1.3 \times 10^{-5}$), but the other lead variants did not affect bone area measures of arm, leg, and spine (Table S9). Altogether, this indicates that the effect of the identified variants on head size is predominantly head-specific.

Regional brain volumetric effects

Head size may reflect growth of specific brain regions. Indeed, 15 lead genetic variants or variants in LD ($r^2 > 0.6$) from 12 genetic loci were previously reported to affect volumes of subregions of the brain (Figure 2A; Table S10). We screened all loci previously associated with these regional brain volumes, and found 16 of those 132 loci significantly related with head size after multiple testing correction (Table S11). To determine if the current findings can be localized to specific brain regions, we investigated the 90 independent head size variants in relation to more fine-grained measures of brain morphometry—corrected for head size—in 22,145 individuals (Figure 2B; Table S12). Thirty-nine variants were associated with one or multiple cortical, subcortical, and global brain regions of which 17 variants were preferentially associated with one or two specific cortical or subcortical regions. For example, rs111939932, an intronic variant in *PCBP2*, is associated with nucleus accumbens volume and is an expression quantitative trait locus (eQTL) for several genes, including *ATP5G2* in the nucleus accumbens and basal ganglia. Further analysis revealed its localized effects on this structure's shape (Figure 2C; Table S13). In the largest GWAS on nucleus accumbens volume,[7] this variant was nominally significant (p = 0.02), showing the improved power of our

CellPress
OPEN ACCESS

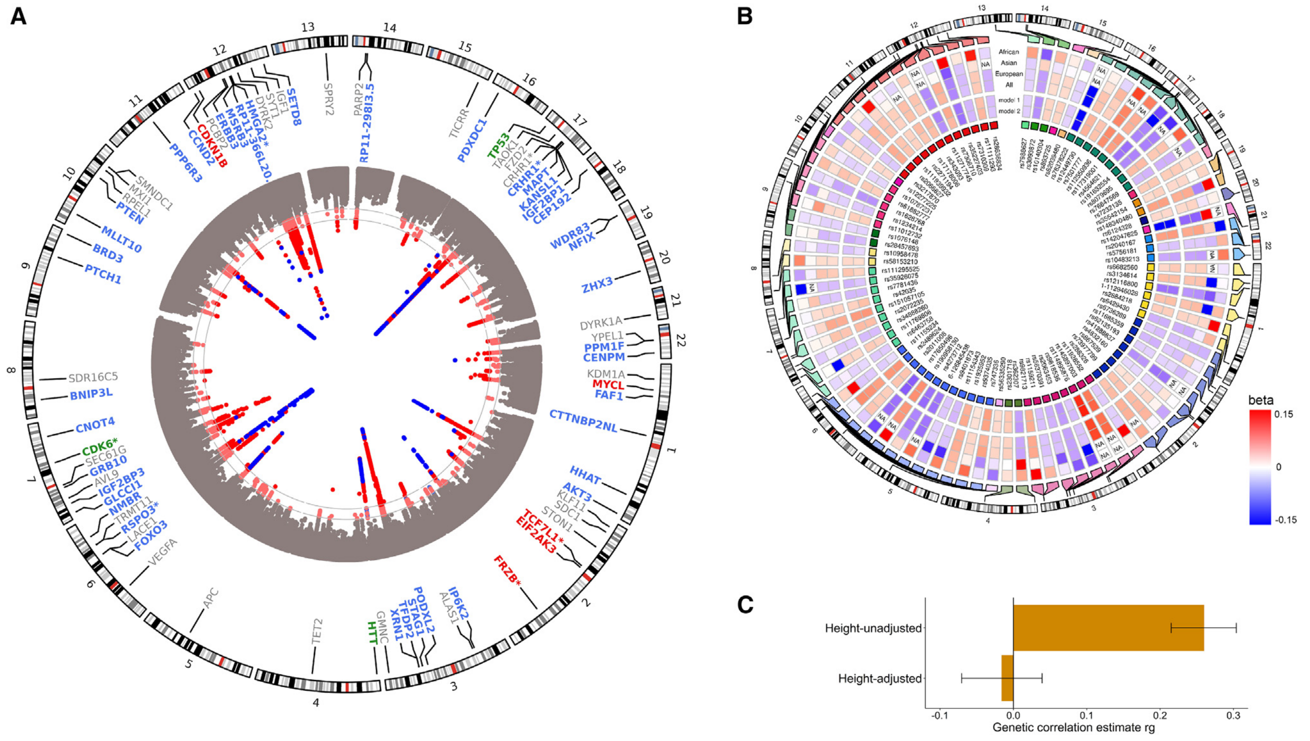

Figure 1. Genome-wide association studies on human head size

(A) Circos Manhattan plot of the European ancestry head size GWAS, with gray lines corresponding to genome-wide significant ($p < 5 \times 10^{-8}$) or sub-significant ($p < 1 \times 10^{-6}$) p value thresholds. Known variants are in blue, novel ones in red. For each lead variant, the nearest gene is presented, with the color corresponding to its position to the lead variant: exonic (red), 3′-UTR (green), intronic (blue), intergenic including up- and downstream, exonic and intronic non-coding RNA (gray). Nearest genes for more than one locus are denoted with an asterisk (*).

(B) Circos heatmap showing the betas of lead variants in African, Asian, and European ancestry meta-analyses, as well as the transancestral meta-analysis. Differences between the height-unadjusted (model 1) and -adjusted (model 2) meta-analysis are also shown.

(C) Bar plot of the genetic correlation coefficient ($\rho_{genetic}$) of the height-unadjusted and -adjusted head size GWAS with the height GWAS, with their accompanying 95% confidence intervals.

current study to identify novel brain morphometry loci. For the other 51 variants there was no apparent association with particular brain regions. Overall, these results suggest that most head size variants affect generalized brain or cranial growth, while a minority influence regional brain growth.

Genetic correlation with neuropsychiatric traits

Genetic correlation analyses with neuropsychiatric traits have been conducted previously.[5,6] We replicated positive genetic correlations with cognitive functioning and Parkinson's disease, also when only including new samples (Figure S1; Table S14). The replicated correlation with Parkinson's disease provides independent evidence for the proposed brain overgrowth hypothesis in this disorder.[8] Novel genetic correlations were found with multiple psychiatric traits; negative correlations with attention-deficit hyperactivity disorder ($\rho_{genetic} = -0.18$, $p = 4.5 \times 10^{-7}$), insomnia ($\rho_{genetic} = -0.19$, $p = 1.8 \times 10^{-5}$), major depressive disorder ($\rho_{genetic} = -0.11$, $p = 2.6 \times 10^{-4}$), and neuroticism ($\rho_{genetic} = -0.11$, $p = 5.4 \times 10^{-4}$) (Figure S1; Table S14). Since psychiatric disorders themselves are genetically correlated, incorporating head size and other brain anatomy traits could aid in disentangling underlying genetic factors.

Pathway analysis

To obtain novel insights into the biological mechanisms underlying head size variation, we performed a gene set enrichment analysis of Kyoto Encyclopedia of Genes and Genomes (KEGG)[9] gene sets and found 14 to be significantly enriched (Figure 3A; Table S15). Nine of those gene sets represent different cancer types that substantially overlap between each other and share underlying biological pathways (Figure 3B). The remaining gene sets represent the p53, Wnt, and ErbB signaling pathways, all involved in tumorigenesis including in the abovementioned cancer types.[10] Remarkably, lead variants in our GWAS were predominantly intragenic for the seven genes in the p53 pathway, eight genes in the Wnt pathway, and six genes in the ErbB-EGFR pathway (Figure 3C), suggesting that modulation of these pathways plays an important role in head size variation.

The p53 signaling pathway showed the strongest enrichment ($p_{adjusted} = 7.6 \times 10^{-4}$) (Figure 3A; Table S15). Tumor suppressor protein p53, encoded by *TP53*, is activated by different stress signals to regulate the cell cycle and apoptosis. Our lead signal in this locus was *TP53* 3′-UTR variant rs78378222 with predicted deleterious effects (CADD = 15.93), which was identified previously.[5] Three other genes in this pathway (*ATR*, *CDK6*, and

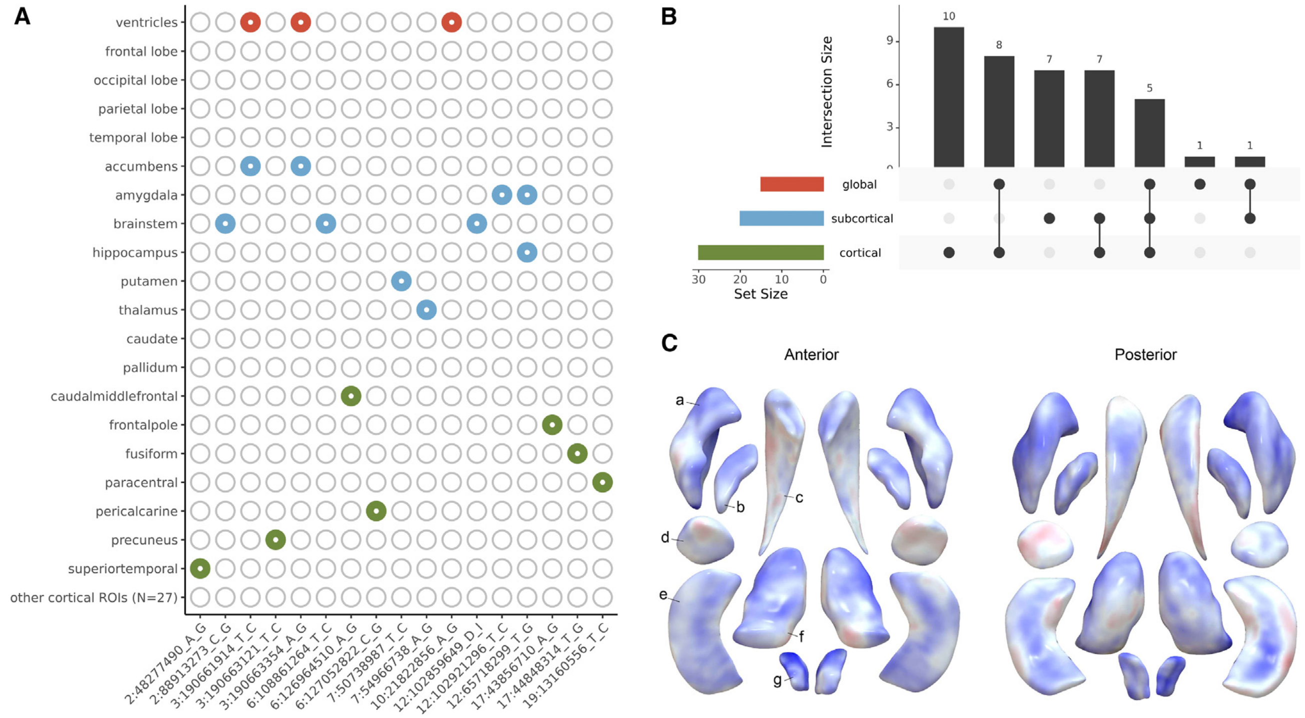

Figure 2. Genetic loci for head size and effects on regional brain volumes

(A) Heatmap showing head size loci that overlap with previously identified loci for global brain volumes (red), subcortical volumes (blue), and cortical region of interest volumes (green).

(B) UpSet plot of associations between head size lead variants and brain volumes. Intersection size corresponds to the frequency of the combination depicted below the bar. Set size corresponds to the frequency of associations with one of the brain volume categories (i.e., global, subcortical, or cortical).

(C) Plot showing the subcortical shape analysis of rs111939932 using log Jacobian determinants. Colors correspond to t values, with positive associations depicted in blue, and negative ones in red. Letters point to different subcortical structures: a, putamen; b, pallidum; c, caudate; d, amygdala; e, hippocampus; f, thalamus; g, accumbens.

PTEN) also contained 3′-UTR or exonic variants in LD ($r^2 > 0.6$) with lead variants. Identified genes act in cell-cycle arrest and cellular senescence (*CDK6, CDK2*, and *CCND2*), apoptosis (*IGF1*), or inhibition of the insulin growth factor (IGF)-1/mammalian target of rapamycin (mTOR) pathway (*PTEN*), suggesting comprehensive involvement of the p53 signaling pathway in head growth. This finding is in line with evidence that p53 signaling regulates both normal and malignant neural stem cell populations.[11–13]

The Wnt signaling pathway has links to carcinogenesis and the developing and adult central nervous system,[14,15] as well as to bone development including cranial growth.[16] Of the eight overlapping genes, three contained exonic or 3′-UTR variants in LD ($r^2 > 0.6$) with identified lead variants (*APC*, *TP53*, and *TCF7L1*). Wnt signaling pathway gene *FRZB*, not annotated in KEGG, also contained exonic and 3′-UTR variants. In total, 1,948 genetic variants in LD with the identified lead variants ($r^2 > 0.6$), including 35 exonic variants, are eQTLs for *WNT3* in 27 different tissues including the cerebellar hemispheres. In addition, various exonic, 3′-UTR and 5′-UTR variants in LD with the lead variants are eQTLs for *TCF7L1* in brain tissues. These observations suggest that variants in this pathway affect brain and cranial growth in the human population.

The ErbB pathway ($p_{adjusted} = 0.014$, Figure 3A), also known as the EGFR signaling pathway, has six overlapping genes near head size variants, which are involved in calcium signaling (*PLCG1*), MAPK signaling (*NCK1* and *MAPK1*), and PI3K-AKT signaling (*ERBB3*, *AKT3*, and *CDKN1B*). In addition, five genetic variants are eQTLs for *EGFR* in the cerebellum. Interestingly, both *AKT3* and *CDKN1B* are linked to clinical head size syndromes and cancer risk[17–20] and contain, respectively, 3′-UTR variants and an exonic variant that reach genome-wide significance. ErbB signaling is involved in neurodevelopment,[21–23] making it a plausible pathway involved in head size variation.

Since the above signaling pathways also have universal roles in cell growth, we determined their enrichment in the height GWAS. We found that only the Wnt signaling pathway was significantly enriched in the height GWAS ($p_{adjusted} = 0.038$), suggesting that the p53 and ErbB signaling pathways are more specifically involved in head growth rather than generalized body growth.

Functional prioritization using gene expression

Using a transcriptome-wide association study (TWAS), we identified 156 head size-associated variants functioning as eQTLs, regulating the expression of 112 genes (eGenes) in relevant tissue types (Table S16). Genomic overlap with additional gene-regulatory and epigenetic features provides evidence for 67 eQTLs regulating the expression of 58 eGenes (RegulomeDB probability score >0.5), including *AKT3* in brain tissue and

CellPress
OPEN ACCESS

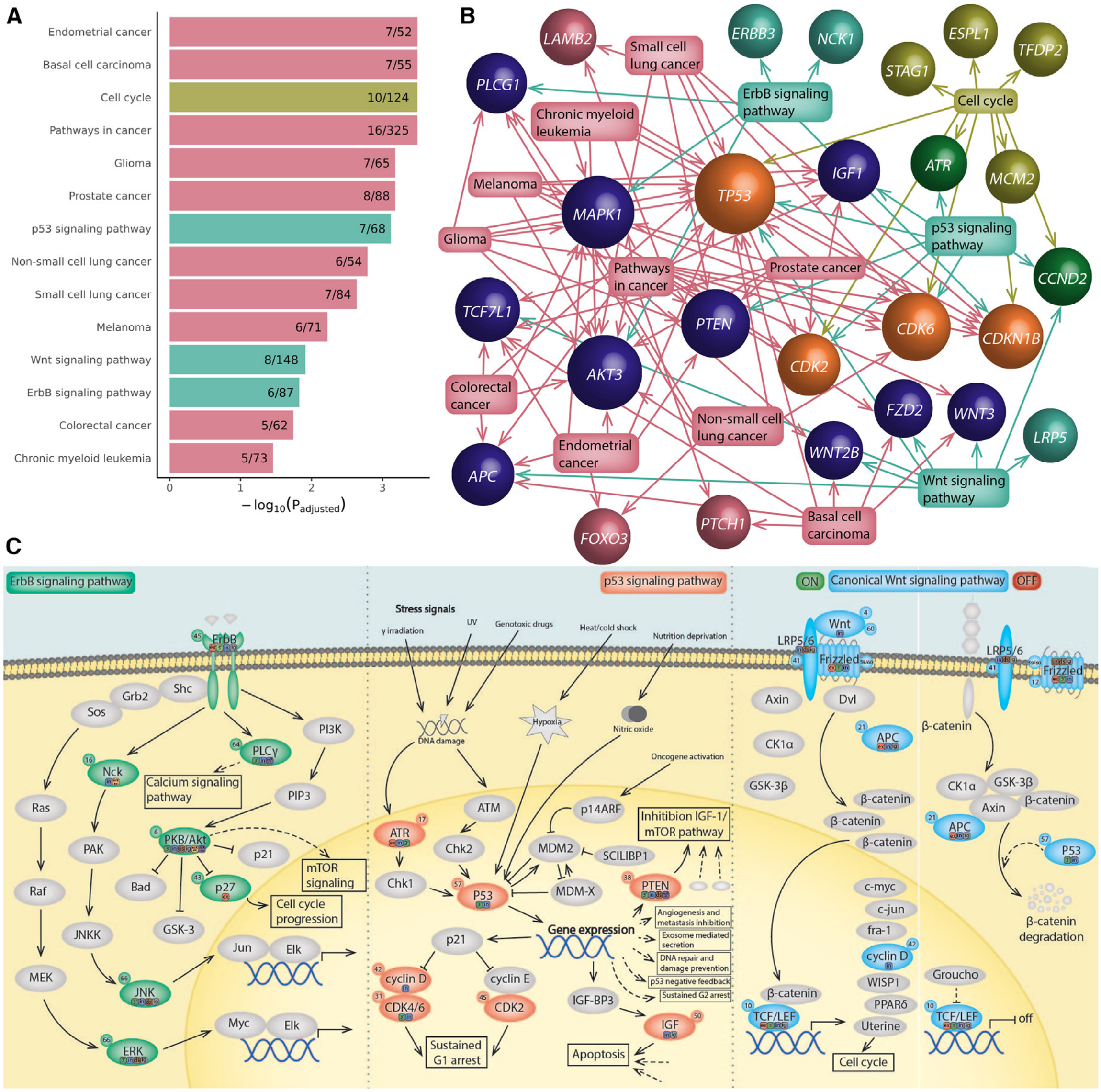

Figure 3. Gene sets enriched in human head size loci

(A) Bar plots presenting enriched KEGG gene sets. $-\log_{10}$ of adjusted *p* value and proportion of nearby genes overlapping with the gene set are presented. Cancer gene sets are depicted in pink, cell growth and death gene sets in yellow-green, and signal transduction gene sets in turquoise.

(B) Network graph showing enriched KEGG gene sets and their included genes near genetic lead variants. Gene sets are shown in squares with arrows to overlapping genes. Colors correspond to gene set categories: only cancer gene sets (pink), only cell growth and death gene sets (yellow-green), only signal transduction gene sets (turquoise), cancer gene sets and cell growth and death gene sets (dark blue), cell growth and death and signal transduction gene sets (green), or all three gene set categories (orange). Sphere size corresponds to the number of gene sets linked to that gene.

(C) Schematic overview of enriched signaling pathways with proteins encoded by genes near (<10 kb) identified genetic loci. Proteins encoded by these genes are colored (green, ErbB pathway; red, p53 pathway; blue, Wnt pathway), other proteins are depicted in gray. Circles next to protein names provide the locus number of the encoding gene. Locations of lead variants and variants in LD ($r^2 > 0.6$) are shown in squares next to the proteins: exonic (e; red), 3′-UTR (3′; green), 5′-UTR (5; light green), intronic (i; blue), intergenic including up- and downstream, exonic and intronic non-coding RNA (g; gray). For Frizzled, not only FZD2 but also FRZB is taken into consideration.

TCF7L1 in the cerebellum—part of the ErbB and Wnt pathway, respectively. In addition, 22 eGenes were suggested to be regulated by 22 splicing QTLs (sQTLs), including *AKT3*. The omnibus test revealed a shared effect for 80 eGenes across the tested gene expression panels (Table S17), including *WNT3*, *AKT3*, and *EGFR*.

Enrichment of Mendelian head size genes and cancer genes

Target genes of GWAS variants are often close to the lead variant.[24] Accordingly, we determined the enrichment of different categories of genes located nearby head size variants, stratified by their distance (Table S18).

First, we investigated genes mutated in OMIM syndromes associated with abnormal head size, i.e., macrocephaly or microcephaly (Tables S19 and S20). We found increasing enrichment for macrocephaly genes with decreasing distance to the lead variants, culminating in a 37-fold enrichment of macrocephaly genes in genes containing an intragenic lead variant (Figure 4A). In contrast, microcephaly genes were not enriched with shorter distance from lead variants. The striking enrichment of macrocephaly genes did not change in the height-adjusted head size GWAS (Table S21). Furthermore, there was only a modest enrichment for macrocephaly genes in the height GWAS, even for the top 67 loci (i.e., the same number of loci as our GWAS; Table S21). Macrocephaly syndrome genes with intragenic lead variants include *AKT3*, *PTCH1*, *PTEN*, *CCND2*, and *NFIX* (Table S19). We conclude that common genetic variants near genes associated with macrocephaly syndromes, but not microcephaly syndromes, contribute to variation in head size in the general population. Our GWAS of head size may therefore identify novel macrocephaly genes. Accordingly, a patient with intellectual disability[25] presented with macrocephaly and a mutation in *TICRR*, a gene for which a lead variant and variants in LD were eQTLs in 12 different tissues. *TICRR* acts in initiation of DNA replication and interacts with *CDK2*,[26] a gene nearby another lead variant. *TICRR* is therefore an interesting candidate macrocephaly syndrome gene.

We determined whether cancer genes are enriched close to lead variants (Figure 4A). Indeed, there was a 9-fold enrichment for high-fidelity cancer genes (first-tier COSMIC[27]) among genes with an intragenic lead variant, which persisted after height adjustment (Table S21). There was only a modest enrichment of cancer genes close to height GWAS variants, providing additional evidence that cancer-related genes are specifically relevant for head size variation.

At a variant-level, no genetic correlation was found with GWAS meta-analyses of various cancer types[28–31] (Table S22).

Autosomal dominance score

We did not observe a significant enrichment for microcephaly genes (Figure 4A). This may be due to differences between the micro- and macrocephaly gene sets. Macrocephaly typically results from mutations with an autosomal dominant inheritance pattern (64.6%, Table S19), whereas microcephaly predominantly involves mutations with an autosomal recessive inheritance pattern (72.3%, Table S20). We observed a profound increase for genes with a predicted dominant inheritance pattern closer to our lead variants (Figure 4B). However, neither dominant nor recessive microcephaly genes were enriched (Table S21) and the predominant recessive inheritance patterns of microcephaly genes could not explain their lack of enrichment. An alternative explanation is that microcephaly syndromes are more clinically heterogeneous and the underlying mechanisms are less specific to brain and cranial growth.

Gain of function and loss of function

The overlap among macrocephaly genes, microcephaly genes, and cancer genes is shown in Figure 4C. Macrocephaly-associated genes were more enriched for high-fidelity cancer genes than microcephaly-associated genes (enrichment ratio 12.9 vs. 3.2, Table S21). We therefore investigated whether the same mutation type, i.e., gain of function or loss of function, causes both macrocephaly syndromes as a germline mutation but also associate with cancer as somatic mutations. We found that this was the case for the vast majority of macrocephaly-associated genes with a defined role in cancer (37 of 41 genes, Table S19), i.e., the same type of mutation associated with both macrocephaly and cancer. Moreover, germline mutations in 14 of these 37 genes, including our GWAS genes *PTEN*, *PTCH1*, and *SUFU*, are associated with a syndrome or condition with a suggested cancer predisposition (Table S19). Our GWAS data and these observations may therefore suggest that subtle up-regulation of oncogenes and oncogenic pathways or down-regulation of tumor suppressor genes and pathways increases head size in the general population.

Brain cell expression

As neural progenitors are the actively dividing cells in the developing brain, their expressed genes may explain the observed genetic variants for head size.[32] Indeed, genes at or near the head size loci were enriched in differentially expressed neural progenitor cell genes (Figure 4D; Table S23). Subsequently, we looked at a single-cell RNA-sequencing (scRNA-seq) dataset from cell types in the human cortex.[33,34] Intriguingly, we find that genes close to head size variants are strongly enriched for genes preferentially expressed in intermediate progenitor cells (IPCs) (Figure 4E; Tables S24 and S25; Figure S2). Increased proliferation of IPCs in a primate-specific area of the brain, the outer region subventricular zone, is believed to be responsible for the evolutionary expansion of the human brain.[35,36] This suggests that genetic variation regulating the proliferation or neuronal differentiation of IPCs plays an important role in determining human head size. Indeed, Wnt pathway genes, p53 pathway genes, and *PTCH1*, *SUFU*, and *NFIX*, which we find near genetic variants determining head size, are examples of regulators of IPCs.[37–43] To understand which type of variants influence head size, we performed a partitioned heritability analysis that classifies variants into categories based on functional elements. We found an enrichment for variants in the regulatory elements of both neural progenitors and their neuronal progenies (enrichment$_{progenitors}$ = 12.7, p = 8.3 × 10^{-4}; enrichment$_{neurons}$ = 16.1, p = 3.7 × 10^{-4}).

Finally, we assessed whether a similar pattern was seen for the Catalog of Somatic Mutations in Cancer (COSMIC) first-tier cancer genes. Indeed, our differential gene expression analysis dataset indeed showed an enrichment of cancer genes in the genes specific for neural progenitors (enrichment = 2.9, p = 1.7 × 10^{-6}, Table S23). However, no significant enrichment was found for IPCs using the scRNA-seq data.

DISCUSSION

Here we performed the largest head size GWAS to date and found that associated genetic variants significantly locate to cancer genes and cancer-associated pathways. Genes near head size variants were enriched for high-fidelity cancer genes even after adjustment for height, suggesting a specific association of head growth with cancer, rather than general growth. Germline mutations in multiple macrocephaly syndrome genes

CellPress
OPEN ACCESS

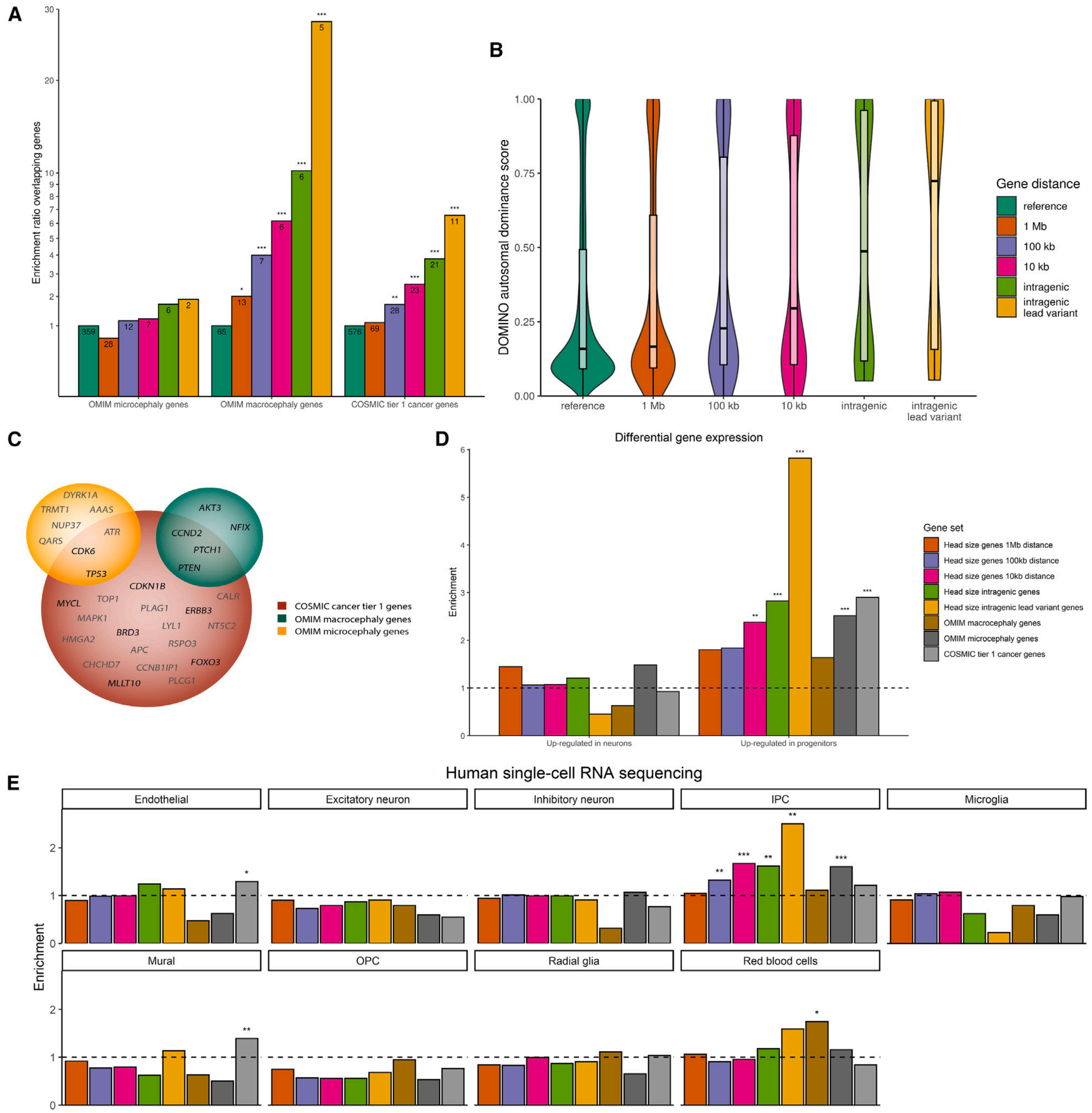

Figure 4. Gene enrichments stratified by distance from head size lead variants

(A) Enrichment of OMIM macro- and microcephaly genes and COSMIC tier 1 genes near identified genetic loci. Depicted are enrichments of genes within 1 Mb (orange), 100 kb (purple), or 10 kb (pink) of identified genetic loci, genes with intragenic genetic variants (light green) and genes with intragenic genetic lead variants (yellow) in comparison with genes in the reference genome (dark green). $^*p < 0.05$; $^{**}p < 0.0125$ (0.05/4); $^{***}p < 0.0025$ (0.05/4/5).

(B) Violin plots showing DOMINO autosomal dominance scores of different gene sets. $^*p < 0.05$; $^{**}p < 0.01$; $^{***}p < 0.001$.

(C) Venn diagram showing genes within 10 kb of genetic loci that overlap with OMIM microcephaly genes (yellow) or macrocephaly genes (green) or COSMIC cancer tier 1 genes (red). Genes with intragenic lead variants are depicted in black, others in gray.

(D) Bar plot showing enrichments of gene sets for genes differentially expressed in neurons and progenitors. $^*p < 0.05$; $^{**}p < 0.025$ (0.05/2); $^{***}p < 0.003$ (0.05/2/8).

(E) Bar plots showing enrichments of gene sets for the various cell types in the human cortical brain using single-cell RNA-sequencing data. $^*p < 0.05$; **FDR < 0.05; $^{***}p < 0.0007$ (0.05/9/8).

are known to be an increased cancer risk, including *PTEN* (Cowden syndrome) and *PTCH1* (Gorlin syndrome) (Table S19). Our GWAS was performed in the general population, which prompts the question of whether the link between head size and cancer extends beyond rare genetic syndromes.

Previous meta-analyses of prospective observational studies found associations between adult height and increased risk for various forms of cancer.[44] Similarly, head circumference at birth has previously been positively associated with brain cancer during childhood,[45] and with different types of cancer later in life including stomach cancer and breast cancer,[46] with stronger associations than for respectively birth weight or birth length. The correlation between head size at birth and breast cancer later in life was further supported by a pooled analysis of 32 studies,[47] but not by another prospective cohort study.[48] Our study provides further evidence for this link between head size and cancer.

The abovementioned observational studies together with our genetic results suggest that early growth rather than later adolescent growth may be associated with neoplasia, since cranial growth is completed around the sixth year of age, whereas height is primarily determined by peri-pubertal growth. Head size at birth and its growth during early infancy in relation to cancer risk therefore deserves further studies to identify potential underlying pathophysiological mechanisms and its potential clinical implications.[45,49,50]

Limitations of the study

Although this study suggests an association between head growth and cancer, further studies are needed to investigate whether head size is causally related to cancer development. In our study, we were not able to account for environmental factors such as socio-economic status and diet, especially during childhood, which would be important to adjust for in future studies. In addition, the clinical implications of the findings of our study need to be investigated, for example if patients with clinical macrocephaly syndromes need to be screened for cancer more extensively.

CONSORTIA

The members of the Cohorts for Heart and Aging Research in Genomic Epidemiology (CHARGE) Consortium are Philippe Amouyel, Konstantinos Arfanakis, Benjamin S. Aribisala, Mark E. Bastin, Ganesh Chauhan, Christopher Chen, Ching-Yu Cheng, Philip L. de Jager, Ian J. Deary, Debra A. Fleischman, Rebecca F. Gottesman, Vilmundur Gudnason, Saima Hilal, Edith Hofer, Deborah Janowitz, J. Wouter Jukema, David C.M. Liewald, Lorna M. Lopez, Oscar Lopez, Michelle Luciano, Oliver Martinez, Wiro J. Niessen, Paul Nyquist, Jerome I. Rotter, Tatjana Rundek, Ralph L. Sacco, Helena Schmidt, Henning Tiemeier, Stella Trompet, Jeroen van der Grond, Henry Völzke, Joanna M. Wardlaw, Lisa Yanek, and Jingyun Yang.

The members of the Enhancing NeuroImaging Genetics through Meta-Analysis (ENIGMA) Consortium are Ingrid Agartz, Saud Alhusaini, Laura Almasy, David Ames, Katrin Amunts, Ole A. Andreassen, Nicola Armstrong, Manon Bernard, John Blangero, Laura M.E. Blanken, Marco P. Boks, Dorret I. Boomsma, Adam M. Brickman, Henry Brodaty, Randy L. Buckner, Jan K. Buitelaar, Dara M. Cannon, Vaughan J. Carr, Stanley V. Catts, M. Mallar Chakravarty, Qiang Chen, Christopher R.K. Ching, Aiden Corvin, Benedicto Crespo-Facorro, Joanne E. Curran, Gareth E. Davies, Eco J.C. de Geus, Greig I. de Zubicaray, Anouk den Braber, Sylvane Desrivières, Allissa Dillman, Srdjan Djurovic, Wayne C. Drevets, Ravi Duggirala, Stefan Ehrlich, Susanne Erk, Thomas Espeseth, Iryna O. Fedko, Guillén Fernández, Simon E. Fisher, Tatiana M. Foroud, Tian Ge, Sudheer Giddaluru, David C. Glahn, Aaron L. Goldman, Robert C. Green, Corina U. Greven, Oliver Grimm, Narelle K. Hansell, Catharina A. Hartman, Ryota Hashimoto, Andreas Heinz, Frans Henskens, Derrek P. Hibar, Beng-Choon Ho, Pieter J. Hoekstra, Avram J. Holmes, Martine Hoogman, Jouke-Jan Hottenga, Hilleke E. Hulshoff Pol, Assen Jablensky, Mark Jenkinson, Tianye Jia, Karl-Heinz Jöckel, Erik G. Jönsson, Sungeun Kim, Marieke Klein, Peter Kochunov, John B. Kwok, Stephen M. Lawrie, Stephanie Le Hellard, Hervé Lemaître, Carmel Loughland, Andre F. Marquand, Nicholas G. Martin, Jean-Luc Martinot, Mar Matarin, Daniel H. Mathalon, Karen A. Mather, Venkata S. Mattay, Colm McDonald, Francis J. McMahon, Katie L. McMahon, Rebekah E. McWhirter, Patrizia Mecocci, Ingrid Melle, Andreas Meyer-Lindenberg, Patricia T. Michie, Yuri Milaneschi, Derek W. Morris, Bryan Mowry, Kwangsik Nho, Thomas E. Nichols, Markus N. Nöthen, Rene L. Olvera, Jaap Oosterlaan, Roel A. Ophoff, Massimo Pandolfo, Christos Pantelis, Irene Pappa, Brenda Penninx, G. Bruce Pike, Paul E. Rasser, Miguel E. Rentería, Simone Reppermund, Marcella Rietschel, Shannon L. Risacher, Nina Romanczuk-Seiferth, Emma Jane Rose, Perminder S. Sachdev, Philipp G. Sämann, Andrew J. Saykin, Ulrich Schall, Peter R. Schofield, Sara Schramm, Gunter Schumann, Rodney Scott, Li Shen, Sanjay M. Sisodiya, Hilkka Soininen, Emma Sprooten, Velandai Srikanth, Vidar M. Steen, Lachlan T. Strike, Anbupalam Thalamuthu, Arthur W. Toga, Paul Tooney, Diana Tordesillas-Gutiérrez, Jessica A. Turner, Maria del C. Valdés Hernández, Dennis van der Meer, Nic J.A. Van der Wee, Neeltje E.M. Van Haren, Dennis van 't Ent, Dick J. Veltman, Henrik Walter, Daniel R. Weinberger, Michael W. Weiner, Wei Wen, Lars T. Westlye, Eric Westman, Anderson M. Winkler, Girma Woldehawariat, Margaret J. Wright, and Jingqin Wu.

STAR★METHODS

Detailed methods are provided in the online version of this paper and include the following:

- KEY RESOURCES TABLE
- RESOURCE AVAILABILITY
  - Lead contact
  - Materials availability
  - Data and code availability
- EXPERIMENTAL MODEL AND SUBJECT DETAILS
  - Study population
  - Genotyping
  - Phenotyping
- METHOD DETAILS
  - Genome-wide association studies
  - Functional annotations
  - Effects on anthropomorphic measures and regional brain volumes
  - Genetic correlations
  - Enrichment analyses
  - Experimental datasets of brain cells

- QUANTIFICATION AND STATISTICAL ANALYSIS

SUPPLEMENTAL INFORMATION

Supplemental information can be found online at https://doi.org/10.1016/j.xcrm.2024.101529.

ACKNOWLEDGMENTS

Acknowledgments are provided for the studies that contributed new samples in addition to samples in previous efforts.[5,6]

The TWAS analysis in this study is supported by the following funding resource: P30AG066546 (South Texas Alzheimer's Disease Research Center).

The 1000BRAINS study was funded by the Institute of Neuroscience and Medicine, Research Center Juelich, Germany. We thank the Heinz Nixdorf Foundation (Germany) for the generous support of the Heinz Nixdorf Recall Study on which 1000BRAINS is based. We also thank the scientists and the study staff of the Heinz Nixdorf Recall Study and 1000BRAINS. Funding was also granted by the Initiative and Networking Fund of the Helmholtz Association (S. Caspers) and the European Union's Horizon 2020 Research and Innovation Programme under grant agreements 785907 (Human Brain Project SGA2; K. Amunts, S. Caspers, and S. Cichon) and 945539 (Human Brain Project SGA3; K. Amunts, S. Caspers, and S. Cichon).

The Three-City (3C) Study (Bordeaux and Dijon) is conducted under a partnership agreement between the Institut National de la Santé et de la Recherche Médicale (INSERM), the Institut de Santé Publique et Développement of the Victor Segalen Bordeaux 2 University, and Sanofi-Aventis. The Fondation pour la Recherche Médicale funded the preparation and initiation of the study. The 3C Study is also supported by the Caisse Nationale Maladie des Travailleurs Salariés, Direction Générale de la Santé, Mutuelle Générale de l'Education Nationale, Institut de la Longévité, Regional Governments of Aquitaine and Bourgogne, Fondation de France, Ministry of Research-INSERM Program "Cohortes et collections de données biologiques," French National Research Agency COGINUT (ANR-06-PNRA-005), the Fondation Plan Alzheimer (FCS 2009–2012), and the Caisse Nationale pour la Solidarité et l'Autonomie (CNSA). This project has received funding from the European Union's Horizon 2020 Research and Innovation Programme under grant agreement nos. 643417 and 640643, the French National Research Agency (ANR) - France 2030: ANR-18-RHUS-0002 and ANR-23-IAHU-0001, and the University of Bordeaux Initiative of Excellence (IdEX). Part of the computations were performed at the Bordeaux Bioinformatics Center (CBiB), the University of Bordeaux, and the CREDIM (Centre de Ressource et Développement en Informatique Médicale) at University of Bordeaux, on a server infrastructure supported by the Fondation Claude Pompidou. The project is supported through the following funding organizations under the aegis of JPND (www.jpnd.eu; BRIDGET project): Australia, National Health and Medical Research Council; Austria, Federal Ministry of Science, Research and Economy; Canada, Canadian Institutes of Health Research; France, French National Research Agency; Germany, Federal Ministry of Education and Research; Netherlands, The Netherlands Organisation for Health Research and Development; and United Kingdom, Medical Research Council.

The Atherosclerosis Risk in Communities (ARIC) study has been funded in whole or in part with federal funds from the National Heart, Lung, and Blood Institute, National Institutes of Health, Department of Health and Human Services (contract numbers HHSN268201700001I, HHSN268201700002I, HHSN268201700003I, HHSN268201700004I, and HHSN268201700005I), R01HL087641 and R01HL086694; National Human Genome Research Institute contract U01HG004402; and National Institutes of Health contract HHSN268200625226C. The authors thank the staff and participants of the ARIC study for their important contributions. Infrastructure was partly supported by grant number UL1RR025005, a component of the National Institutes of Health and NIH Roadmap for Medical Research. This project was supported in part by National Institute of Neurological Disorders and Stroke grant NS087541.

Data and samples were collected by the Australian Schizophrenia Research Bank (ASRB), supported by the Australian NHMRC, the Pratt Foundation, Ramsay Health Care, and the Viertel Charitable Foundation. The ASRB was also supported by the Schizophrenia Research Institute (Australia), utilizing infrastructure funding from NSW Health and the Macquarie Group Foundation. DNA analysis was supported by the Neurobehavioral Genetics Unit, using funding from NSW Health. M.J.C. was supported by an NHMRC Senior Research Fellowship (1121474), and M.J.C. and M.J.G. were supported by NHMRC project grants 1147644 and 1051672.

The authors are deeply indebted to Gael Jobard, Marc Joliot, Emmanuel Mellet, Laurent Petit, and Laure Zago for their contribution to the design, acquisition, and analyses of the BIL&GIN. A.C.-C. was funded by a grant to C.F. from the Netherlands Organization for Scientific Research (NWO) (054-15-101) and B.M. and F.C. by a grant from the French National Research Agency (ANR) (grant no. 15-HBPR-0001-03) as part of the FLAG-ERA consortium project "MULTI-LATERAL," a partner project to the European Union's Flagship Human Brain Project.

The Coronary Artery Risk Development in Young Adults Study (CARDIA) is conducted and supported by the National Heart, Lung, and Blood Institute (NHLBI) in collaboration with the University of Alabama at Birmingham (75N92023D00002 and 75N92023D00005), Northwestern University (75N92023D00004), University of Minnesota (75N92023D00006), and Kaiser Foundation Research Institute (75N92023D00003). CARDIA was also partially supported by the Intramural Research Program of the National Institute on Aging (NIA) and an intra-agency agreement between NIA and NHLBI (AG0005). This manuscript has been reviewed by CARDIA for scientific content.

The CROMIS-2 ICH study is funded by the Stroke Association and British Heart Foundation. Funding for genotyping was provided by the UCLH/UCL National Institute for Health Research (NIHR) Biomedical Research Centre.

The Diabetes Heart Study (DHS) was supported in part by the National Institutes of Health through R01 HL67348, R01 HL092301, R01 NS058700, R01 NS075107, and R01 AG058921 and the General Clinical Research Center at Wake Forest School of Medicine (M01 RR07122 and F32 HL085989). The authors thank the investigators, staff, and participants of the DHS for their valuable contributions.

The Duke Neurogenetics Study (DNS) was supported by Duke University as well as National Institutes of Health grants R01DA033369 and R01DA031579. R.A., A.R.K., and A.R.H. received further support from National Institutes of Health grant R01AG049789.

We are grateful to all the participants of the Epidemiological Prevention Study Zoetermeer (EPOZ). We would like to thank Dr. Ir. Natalie Terzikhan for imputing the genetic data.

The Erasmus Stroke Study (ESS) was supported by Stroke Research Foundation and Erasmus MC MRACE grants.

This work was supported by the National Center for Research Resources at the National Institutes of Health (grant numbers NIH 1 U24 RR021992 [Function Biomedical Informatics Research Network] and NIH 1 U24 RR025736-01 [Biomedical Informatics Research Network Coordinating Center]), the National Center for Research Resources and the National Center for Advancing Translational Sciences, National Institutes of Health through grant UL1 TR000153, and the National Institutes of Health through 5R01MH094524 and P20GM103472. J.M.F. was funded by the Veterans Administration (1IK6CX002519).

This work was supported by the NHLBI's Framingham Heart Study (contracts N01-HC-25195, HHSN268201500001I, and 75N92019D00031) and its contract with Affymetrix, Inc., for genotyping services (contract no. N02-HL-6-4278). A portion of this research utilized the Linux Cluster for Genetic Analysis (LinGA-II) funded by the Robert Dawson Evans Endowment of the Department of Medicine at Boston University School of Medicine and Boston Medical Center. This study was also supported by grants from the National Institute of Aging (R01s AG033040, AG033193, AG054076, AG049607, AG008122, AG016495, U01-AG049505, AG052409, AG058589, and RF1AG059421) and the National Institute of Neurological Disorders and Stroke (R01-NS017950). We would like to thank the dedication of the Framingham Study participants as well as the Framingham Study team, especially investigators and staff from the Neurology group, for their contributions to data collection. C.D. is supported by the Alzheimer's Disease Center (P30 AG 010129) and by National Institute on Aging grants R01AG054076 and P30 AG072972. The views expressed in this manuscript are those of the authors and do not necessarily

represent the views of the NHLBI, the National Institutes of Health, or the US Department of Health and Human Services.

The Generation R Study was supported by ZonMw (TOP project 91211021 [T.W.]), the Sophia Foundation (grant S18-20 [R.L.M.]), and the European Union's Horizon 2020 Research and Innovation Programme (no. 733206 LifeCycle Project [S. Lamballais], no. 848158 EarlyCause Project [C.A.M.C.], and Marie Skłodowska-Curie grant agreement no. 707404 [C.A.M.C.]). The Generation R Study is conducted by the Erasmus Medical Center in close collaboration with the School of Law and Faculty of Social Sciences of the Erasmus University Rotterdam; the Municipal Health Service Rotterdam Area, Rotterdam; the Rotterdam Homecare Foundation, Rotterdam; and the Stichting Trombosedienst & Artsenlaboratorium Rijnmond (STAR-MDC), Rotterdam. We gratefully acknowledge the contribution of children and parents, general practitioners, hospitals, midwives, and pharmacies in Rotterdam. The general design of the Generation R Study is made possible by financial support from the Erasmus Medical Center, Rotterdam; the Erasmus University Rotterdam; the Netherlands Organization for Health Research and Development (ZonMw); the Netherlands Organisation for Scientific Research (NWO); the Ministry of Health, Welfare and Sport; and the Ministry of Youth and Families.

The Trøndelag Health Study (HUNT) is a collaboration between HUNT Research Center (Faculty of Medicine and Health Sciences, NTNU – Norwegian University of Science and Technology), Nord-Trøndelag County Council, Central Norway Health Authority, and the Norwegian Institute of Public Health. HUNT MRI was funded by the liaison committee between the Central Norway Regional Health Authority and the Norwegian University of Science and Technology as well as the Norwegian National Advisory Unit for functional magnetic resonance imaging (MRI).

The Institute of Mental Health (IMH) study was supported by research grants from the National Healthcare Group, Singapore (SIG/05004 and SIG/05028), and the Singapore Bioimaging Consortium (RP C-009/2006) research grants awarded to K. Sim.

The Internet-based Students HeAlth Research Enterprise (i-Share) study is conducted by the Universities of Bordeaux and Versailles Saint-Quentin-en-Yvelines (France). The i-Share study has received funding from the French National Agency (Agence Nationale de la Recherche [ANR]) via the "Investissements d'Avenir" program (grant number ANR-10-COHO-05) and from the University of Bordeaux Initiative of Exellence (IdEX). This project has also received funding from the European Research Council (ERC) under the European Union's Horizon 2020 Research and Innovation Programme under grant agreement no. 640643. The 3C Study is conducted under a partnership agreement among the Institut National de la Santé et de la Recherche Médicale (INSERM), the University of Bordeaux, and Sanofi-Aventis. The Fondation pour la Recherche Médicale funded the preparation and initiation of the study. S. Debette has received investigator-initiated research funding from the French National Research Agency (ANR) and from the Fondation Leducq. This work was supported by the National Foundation for Alzheimer's Disease and Related Disorders, the Institut Pasteur de Lille, the LabEx DISTALZ, and the Centre National de Génotypage. This project is an EU Joint Program - Neurodegenerative Disease Research (JPND) project. The project is supported through the following funding organizations under the aegis of JPND (www.jpnd.eu): Australia, National Health and Medical Research Council; Austria, Federal Ministry of Science, Research and Economy; Canada, Canadian Institutes of Health Research; France, French National Research Agency; Germany, Federal Ministry of Education and Research; Netherlands, The Netherlands Organisation for Health Research and Development; and United Kingdom, Medical Research Council. In addition, S. Debette is supported by a grant overseen by the French National Research Agency (ANR) as part of the Investment for the Future Program ANR-18-RHUS-0002. Part of the computations were performed at the Bordeaux Bioinformatics Center (CBiB), University of Bordeaux, and at the CREDIM at University of Bordeaux, on a server infrastructure supported by the Fondation Claude Pompidou.

LIFE-Adult is supported by LIFE – Leipzig Research Center for Civilization Diseases, an organizational unit affiliated to the Medical Faculty of the University of Leipzig. LIFE is funded by means of the European Union, European Regional Development Fund (ERDF) and by funds of the Free State of Saxony within the framework of the excellence initiative (project numbers 713–241202, 713–241202, 14505/2470, and 14575/2470). We thank all participants and Kerstin Wirkner, Ulrike Scharrer, Katrin Arelin, and everyone involved in MRI data acquisition and analysis.

The Nagahama Prospective Genome Cohort for Comprehensive Human Bioscience is grateful to the Nagahama City Office and the nonprofit organization Zeroji Club for their help in conducting the study. This project is supported by operational funds of Kyoto University and the Top Global University Project of the Ministry of Education, Culture, Sports, Science and Technology (MEXT) in Japan. We also receive Grant-in-Aid for Scientific Research from the Japan Society for the Promotion of Science and research grants from the Japan Agency for Medical Research and Development for the Practical Research Project for Rare/Intractable Diseases and the Comprehensive Research on Aging and Health Science for Dementia R&D. M.-G.D. received a grant from the Fondation Bettencourt Schueller.

As part of the Poznan MS study, M.A.P. reported receiving grants from the Polish National Science Centre: 2011/01/D/NZ4/05801.

The Rotterdam Study (RS) is funded by Erasmus Medical Center and Erasmus University, Rotterdam; the Netherlands Organization for Health Research and Development (ZonMw); the Research Institute for Diseases in the Elderly (RIDE); the Ministry of Education, Culture and Science; the Ministry for Health, Welfare and Sports; the European Commission (DG XII); and the Municipality of Rotterdam. The authors are grateful to the study participants, the staff from the Rotterdam Study, and the participating general practitioners and pharmacists. The generation and management of GWAS genotype data for the Rotterdam Study (RS I, RS II, and RS III) were executed by the Human Genotyping Facility of the Genetic Laboratory of the Department of Internal Medicine, Erasmus MC, Rotterdam, the Netherlands. The GWAS datasets are supported by the Netherlands Organisation of Scientific Research NWO Investments (no. 175.010.2005.011, 911-03-012); the Genetic Laboratory of the Department of Internal Medicine, Erasmus MC; the Research Institute for Diseases in the Elderly (014-93-015; RIDE2); the Netherlands Genomics Initiative (NGI)/ Netherlands Organisation for Scientific Research (NWO); and Netherlands Consortium for Healthy Aging (NCHA) project no. 050-060-810. We thank Pascal Arp, Mila Jhamai, Marijn Verkerk, Lizbeth Herrera, Marjolein Peters, and Carolina Medina-Gomez for their help in creating the GWAS database and Karol Estrada, Yurii Aulchenko, and Carolina Medina-Gomez for the creation and analysis of imputed data. H.H.H.A. is supported by ZonMw grant number 916.19.151.

Study of Health in Pomerania - TREND (SHIP-TREND) is part of the Community Medicine Research net of the University of Greifswald, Germany, which is funded by the Federal Ministry of Education and Research (grant nos. 01ZZ9603, 01ZZ0103, and 01ZZ0403), the Ministry of Cultural Affairs, and the Social Ministry of the Federal State of Mecklenburg-West Pomerania. MRI scans and genome-wide SNP typing in SHIP-TREND have been supported by a joint grant from Siemens Healthineers, Erlangen, Germany and the Federal State of Mecklenburg-West Pomerania. The University of Greifswald is a member of the Caché Campus program of the InterSystems GmbH.

The Canadian Institutes of Health Research and the Heart and Stroke Foundation of Canada fund the Saguenay Youth Study (SYS). Computations were performed on the GPC supercomputer at the SciNet HPC Consortium. SciNet is funded by the Canada Foundation for Innovation under the auspices of Compute Canada, the Government of Ontario, Ontario Research Fund - Research Excellence, and the University of Toronto.

This research has been conducted using the UK Biobank Resource under application number 23509.

Vitamin D Intervention in Infants (VIDI) is supported by the Finnish Medical Foundation, the Academy of Finland, the Sigrid Jusélius Foundation, the Swedish Research Council, the Novo Nordisk Foundation, Finska Läkaresällskapet, and the Folkhälsan Research Foundation. We want to thank Dr. Helena Hauta-alus, Dr. Elisa Holmlund-Suila, Dr. Saara Valkama, and Dr. Jenni Rosendahl for their contribution in acquiring the data.

AUTHOR CONTRIBUTIONS

Performed statistical analysis: M.J.K., R.A.P., T.E.E., C.L.S., A.M., S.v.d.A., M.-G.D., X.J., I.C.H., S. Lamballais, M.A.P., C.E.L., A.C.-C., T.G.M.v.E., C.S.R., J.S., M. Scholz, A.K., G.H.Y.L., R.A., J.R.A., F.-C.H., A.R.A., M.L., A.

Tsuchida, M.W.A.T., N.A., Y.P., D.L., M. Sargurupremraj, F.B., R.N.B., S. Dalvie, M.J.G., S.H., N.J., Y.K., A.R.K., S. Li, K.L., S.E.M., S.N., N.D.P., Y.Q., W.R.R., G.V.R., S.Y.S., W.V.W., K.W., M.J.C., C.-L.C., and H.H.H.A. Acquired data: T.E.E., I.C.H., D.H.K.v.D.-N., M.A.P., T.G.M.v.E., A.K.H., A.S.B., F.B., J.C.B., D.B., R.N.B., R.B., S. Caspers, G.C., C.A.M.C., J.-F.D., C.D., M.E.-C., J.M.F., B.F., B.I.F., N.F., C.H., P.H., G.H., M.K.I., C.R.J., C.J., Y.K., A.R.K., W.T.L., F. Macciardi, O.M., B.M., S.E.M., S. Miyamoto, S. Moebus, T.H.M., R.M., T.W.M., M.N., Z.P., A.P., R.S., P.J.S., K. Setoh, S. Sidney, B.S.P., J.L.S., Y.T., A. Teumer, A.U., A.v.d.L., M.W.V., D.J.W., B.G.W., A.V.W., Q.Y., K.Y., H.G.B., Q.L.G., K. Sim, D.J.S., D.W.B., M.J.C., A.R.H., S.A., A.V., T.P., S. Cichon, V.D.C., F.C., L.J.L., T.W., P.J.K., H.H., M.F., F. Matsuda, H.J.G., M.A.I., S. Debette, P.M.T., S. Seshadri, and H.H.H.A. Drafted the manuscript: M.J.K., R.A.P., and H.H.H.A. Revised the manuscript for important intellectual content: M.J.K., R.A.P., T.E.E., C.L.S., A.M., S.v.d.A., M.-G.D., X.J., I.C.H., D.H.K.v.D.-N., S. Lamballais, M.A.P., C.E.L., A.C.-C., T.G.M.v.E., C.S.R., J.S., M. Scholz, A.K.H., A.K., G.H.Y.L., R.A., J.R.A., F.-C.H., A.R.A., M.L., A. Tsuchida, M.W.A.T., N.A., Y.P., D.L., M. Sargurupremraj, A.S.B., F.B., J.C.B., D.B., R.N.B., R.B., S. Caspers, G.C., C.A.M.C., S. Dalvie, J.-F.D., C.D., M.E.-C., J.M.F., B.F., B.I.F., N.F., M.J.G., S.H., C.H., P.H., G.H., M.K.I., C.R.J., N.J., C.J., Y.K., A.R.K., S. Li, K.L., W.T.L., F. Macciardi, O.M., B.M., S.E.M., S. Miyamoto, S. Moebus, T.H.M., R.M., T.W.M., M.N., S.N., N.D.P., Z.P., A.P., Y.Q., W.R.R., G.V.R., R.S., P.J.S., K. Setoh, C.Y.S., S. Seshadri, B.S.P., J.L.S., Y.T., A. Teumer, A.U., A.v.d.L., M.W.V., D.J.W., B.G.W., A.V.W., K.W., Q.Y., K.Y., H.G.B., Q.L.G., K. Sim, D.J.S., D.W.B., M.J.C., A.R.H., C.-L.C., S.A., A.V., T.P., S. Cichon, V.D.C., F.C., L.J.L., T.W., P.J.K., H.H., M.F., F. Matsuda, H.J.G., M.A.I., S. Debette, P.M.T., S. Seshadri, and H.H.H.A.

DECLARATION OF INTERESTS

H.H. and I.C.H. received funding from Alzheimer's Research UK and the Dunhill Medical Trust Foundation. M.A.P. reported receiving grants and personal and travel fees from Roche, Novartis, Merck, and Biogen outside the submitted work. M. Scholz receives funding from Pfizer Inc. for a project not related to this research. C.D. serves as a consultant of Novartis Pharmaceuticals. B.F. has received educational speaking fees from Medice. N.J. and P.M.T. are MPIs of a research grant from Biogen Inc. for work unrelated to the contents of this manuscript. D.J.W. received funding from the Stroke Foundation/British Heart Foundation. D.J.S. has received consultancy honoraria from Discovery Vitality, Johnson & Johnson, Kanna, L'Oreal, Lundbeck, Orion, Sanofi, Servier, Takeda, and Vistagen. H.H. received funding from MRC, Wellcome Trust, and NIHR UCLH BRC. H.J.G. has received travel grants and speaker's honoraria from Fresenius Medical Care, Neuraxpharm, and Janssen Cilag as well as research funding from Fresenius Medical Care.

Received: November 30, 2021
Revised: September 18, 2023
Accepted: April 4, 2024
Published: May 3, 2024

REFERENCES

1. Dekaban, A.S. (1978). Changes in brain weights during the span of human life: relation of brain weights to body heights and body weights. Ann. Neurol. *4*, 345–356.
2. Smit, D.J.A., Luciano, M., Bartels, M., van Beijsterveldt, C.E.M., Wright, M.J., Hansell, N.K., Brunner, H.G., Estourgie-van Burk, G.F., de Geus, E.J.C., Martin, N.G., and Boomsma, D.I. (2010). Heritability of head size in Dutch and Australian twin families at ages 0-50 years. Twin Res. Hum. Genet. *13*, 370–380.
3. Pirozzi, F., Nelson, B., and Mirzaa, G. (2018). From microcephaly to megalencephaly: determinants of brain size. Dialogues Clin. Neurosci. *20*, 267–282.
4. Jansen, P.R., Nagel, M., Watanabe, K., Wei, Y., Savage, J.E., de Leeuw, C.A., van den Heuvel, M.P., van der Sluis, S., and Posthuma, D. (2020). Genome-wide meta-analysis of brain volume identifies genomic loci and genes shared with intelligence. Nat. Commun. *11*, 5606.
5. Haworth, S., Shapland, C.Y., Hayward, C., Prins, B.P., Felix, J.F., Medina-Gomez, C., Rivadeneira, F., Wang, C., Ahluwalia, T.S., Vrijheid, M., et al. (2019). Low-frequency variation in TP53 has large effects on head circumference and intracranial volume. Nat. Commun. *10*, 357.
6. Adams, H.H.H., Hibar, D.P., Chouraki, V., Stein, J.L., Nyquist, P.A., Rentería, M.E., Trompet, S., Arias-Vasquez, A., Seshadri, S., Desrivières, S., et al. (2016). Novel genetic loci underlying human intracranial volume identified through genome-wide association. Nat. Neurosci. *19*, 1569–1582.
7. Satizabal, C.L., Adams, H.H.H., Hibar, D.P., White, C.C., Knol, M.J., Stein, J.L., Scholz, M., Sargurupremraj, M., Jahanshad, N., Roshchupkin, G.V., et al. (2019). Genetic architecture of subcortical brain structures in 38,851 individuals. Nat. Genet. *51*, 1624–1636.
8. Krabbe, K., Karlsborg, M., Hansen, A., Werdelin, L., Mehlsen, J., Larsson, H.B.W., and Paulson, O.B. (2005). Increased intracranial volume in Parkinson's disease. J. Neurol. Sci. *239*, 45–52.
9. Kanehisa, M., Furumichi, M., Tanabe, M., Sato, Y., and Morishima, K. (2017). KEGG: new perspectives on genomes, pathways, diseases and drugs. Nucleic Acids Res. *45*, D353–D361.
10. Sanchez-Vega, F., Mina, M., Armenia, J., Chatila, W.K., Luna, A., La, K.C., Dimitriadoy, S., Liu, D.L., Kantheti, H.S., Saghafinia, S., et al. (2018). Oncogenic Signaling Pathways in The Cancer Genome Atlas. Cell *173*, 321–337.e10.
11. Zheng, H., Ying, H., Yan, H., Kimmelman, A.C., Hiller, D.J., Chen, A.-J., Perry, S.R., Tonon, G., Chu, G.C., Ding, Z., et al. (2008). p53 and Pten control neural and glioma stem/progenitor cell renewal and differentiation. Nature *455*, 1129–1133. https://doi.org/10.1038/nature07443.
12. Meletis, K., Wirta, V., Hede, S.-M., Nistér, M., Lundeberg, J., and Frisén, J. (2006). p53 suppresses the self-renewal of adult neural stem cells. Development *133*, 363–369. https://doi.org/10.1242/dev.02208.
13. Stecca, B., and Ruiz i Altaba, A. (2009). A GLI1-p53 inhibitory loop controls neural stem cell and tumour cell numbers. EMBO J. *28*, 663–676. https://doi.org/10.1038/emboj.2009.16.
14. Inestrosa, N.C., and Varela-Nallar, L. (2015). Wnt signalling in neuronal differentiation and development. Cell Tissue Res. *359*, 215–223. https://doi.org/10.1007/s00441-014-1996-4.
15. Chenn, A., and Walsh, C.A. (2002). Regulation of cerebral cortical size by control of cell cycle exit in neural precursors. Science *297*, 365–369. https://doi.org/10.1126/science.1074192.
16. Clevers, H. (2006). Wnt/beta-catenin signaling in development and disease. Cell *127*, 469–480. https://doi.org/10.1016/j.cell.2006.10.018.
17. Grey, W., Izatt, L., Sahraoui, W., Ng, Y.M., Ogilvie, C., Hulse, A., Tse, E., Holic, R., and Yu, V. (2013). Deficiency of the cyclin-dependent kinase inhibitor, CDKN1B, results in overgrowth and neurodevelopmental delay. Hum. Mutat. *34*, 864–868.
18. Wasserman, J.D., Tomlinson, G.E., Druker, H., Kamihara, J., Kohlmann, W.K., Kratz, C.P., Nathanson, K.L., Pajtler, K.W., Parareda, A., Rednam, S.P., et al. (2017). Multiple Endocrine Neoplasia and Hyperparathyroid-Jaw Tumor Syndromes: Clinical Features, Genetics, and Surveillance Recommendations in Childhood. Clin. Cancer Res. *23*, e123–e132.
19. Alcantara, D., Timms, A.E., Gripp, K., Baker, L., Park, K., Collins, S., Cheng, C., Stewart, F., Mehta, S.G., Saggar, A., et al. (2017). Mutations of AKT3 are associated with a wide spectrum of developmental disorders including extreme megalencephaly. Brain *140*, 2610–2622.
20. Davies, M.A., Stemke-Hale, K., Tellez, C., Calderone, T.L., Deng, W., Prieto, V.G., Lazar, A.J.F., Gershenwald, J.E., and Mills, G.B. (2008). A novel AKT3 mutation in melanoma tumours and cell lines. Br. J. Cancer *99*, 1265–1268.
21. Mei, L., and Nave, K.A. (2014). Neuregulin-ERBB signaling in the nervous system and neuropsychiatric diseases. Neuron *83*, 27–49.

22. Aguirre, A., Dupree, J.L., Mangin, J.M., and Gallo, V. (2007). A functional role for EGFR signaling in myelination and remyelination. Nat. Neurosci. *10*, 990–1002.

23. Kataria, H., Alizadeh, A., and Karimi-Abdolrezaee, S. (2019). Neuregulin-1/ErbB network: An emerging modulator of nervous system injury and repair. Prog. Neurobiol. *180*, 101643. https://doi.org/10.1016/j.pneurobio.2019.101643.

24. Brodie, A., Azaria, J.R., and Ofran, Y. (2016). How far from the SNP may the causative genes be? Nucleic Acids Res. *44*, 6046–6054.

25. Lelieveld, S.H., Reijnders, M.R.F., Pfundt, R., Yntema, H.G., Kamsteeg, E.J., de Vries, P., de Vries, B.B.A., Willemsen, M.H., Kleefstra, T., Löhner, K., et al. (2016). Meta-analysis of 2,104 trios provides support for 10 new genes for intellectual disability. Nat. Neurosci. *19*, 1194–1196.

26. Kumagai, A., Shevchenko, A., Shevchenko, A., and Dunphy, W.G. (2010). Treslin collaborates with TopBP1 in triggering the initiation of DNA replication. Cell *140*, 349–359.

27. Sondka, Z., Bamford, S., Cole, C.G., Ward, S.A., Dunham, I., and Forbes, S.A. (2018). The COSMIC Cancer Gene Census: describing genetic dysfunction across all human cancers. Nat. Rev. Cancer *18*, 696–705.

28. Zhang, H., Ahearn, T.U., Lecarpentier, J., Barnes, D., Beesley, J., Qi, G., Jiang, X., O'Mara, T.A., Zhao, N., Bolla, M.K., et al. (2020). Genome-wide association study identifies 32 novel breast cancer susceptibility loci from overall and subtype-specific analyses. Nat. Genet. *52*, 572–581.

29. Phelan, C.M., Kuchenbaecker, K.B., Tyrer, J.P., Kar, S.P., Lawrenson, K., Winham, S.J., Dennis, J., Pirie, A., Riggan, M.J., Chornokur, G., et al. (2017). Identification of 12 new susceptibility loci for different histotypes of epithelial ovarian cancer. Nat. Genet. *49*, 680–691.

30. Schumacher, F.R., Al Olama, A.A., Berndt, S.I., Benlloch, S., Ahmed, M., Saunders, E.J., Dadaev, T., Leongamornlert, D., Anokian, E., Cieza-Borrella, C., et al. (2018). Association analyses of more than 140,000 men identify 63 new prostate cancer susceptibility loci. Nat. Genet. *50*, 928–936.

31. Rashkin, S.R., Graff, R.E., Kachuri, L., Thai, K.K., Alexeeff, S.E., Blatchins, M.A., Cavazos, T.B., Corley, D.A., Emami, N.C., Hoffman, J.D., et al. (2020). Pan-cancer study detects genetic risk variants and shared genetic basis in two large cohorts. Nat. Commun. *11*, 4423.

32. Aygün, N., Elwell, A.L., Liang, D., Lafferty, M.J., Cheek, K.E., Courtney, K.P., Mory, J., Hadden-Ford, E., Krupa, O., de la Torre-Ubieta, L., et al. (2021). Genetic effects on brain traits impact cell-type specific gene regulation during neurogenesis. Preprint at bioRxiv. https://doi.org/10.1101/2020.10.21.349019.

33. Skene, N.G., Bryois, J., Bakken, T.E., Breen, G., Crowley, J.J., Gaspar, H.A., Giusti-Rodriguez, P., Hodge, R.D., Miller, J.A., Muñoz-Manchado, A.B., et al. (2018). Genetic identification of brain cell types underlying schizophrenia. Nat. Genet. *50*, 825–833.

34. Bhaduri, A., Andrews, M.G., Mancia Leon, W., Jung, D., Shin, D., Allen, D., Jung, D., Schmunk, G., Haeussler, M., Salma, J., et al. (2020). Cell stress in cortical organoids impairs molecular subtype specification. Nature *578*, 142–148.

35. Hansen, D.V., Lui, J.H., Parker, P.R.L., and Kriegstein, A.R. (2010). Neurogenic radial glia in the outer subventricular zone of human neocortex. Nature *464*, 554–561.

36. Cárdenas, A., Villalba, A., de Juan Romero, C., Picó, E., Kyrousi, C., Tzika, A.C., Tessier-Lavigne, M., Ma, L., Drukker, M., Cappello, S., and Borrell, V. (2018). Evolution of Cortical Neurogenesis in Amniotes Controlled by Robo Signaling Levels. Cell *174*, 590–606.e21.

37. Munji, R.N., Choe, Y., Li, G., Siegenthaler, J.A., and Pleasure, S.J. (2011). Wnt signaling regulates neuronal differentiation of cortical intermediate progenitors. J. Neurosci. *31*, 1676–1687.

38. Chodelkova, O., Masek, J., Korinek, V., Kozmik, Z., and Machon, O. (2018). Tcf7L2 is essential for neurogenesis in the developing mouse neocortex. Neural Dev. *13*, 8.

39. Pollock, A., Bian, S., Zhang, C., Chen, Z., and Sun, T. (2014). Growth of the developing cerebral cortex is controlled by microRNA-7 through the p53 pathway. Cell Rep. *7*, 1184–1196.

40. Glickstein, S.B., Monaghan, J.A., Koeller, H.B., Jones, T.K., and Ross, M.E. (2009). Cyclin D2 is critical for intermediate progenitor cell proliferation in the embryonic cortex. J. Neurosci. *29*, 9614–9624.

41. Antonelli, F., Casciati, A., Tanori, M., Tanno, B., Linares-Vidal, M.V., Serra, N., Bellés, M., Pannicelli, A., Saran, A., and Pazzaglia, S. (2018). Alterations in Morphology and Adult Neurogenesis in the Dentate Gyrus of Patched1 Heterozygous Mice. Front. Mol. Neurosci. *11*, 168.

42. Yabut, O.R., Ng, H.X., Fernandez, G., Yoon, K., Kuhn, J., and Pleasure, S.J. (2016). Loss of Suppressor of Fused in Mid-Corticogenesis Leads to the Expansion of Intermediate Progenitors. J. Dev. Biol. *4*, 29.

43. Harris, L., Zalucki, O., Gobius, I., McDonald, H., Osinki, J., Harvey, T.J., Essebier, A., Vidovic, D., Gladwyn-Ng, I., Burne, T.H., et al. (2016). Transcriptional regulation of intermediate progenitor cell generation during hippocampal development. Development *143*, 4620–4630.

44. Green, J., Cairns, B.J., Casabonne, D., Wright, F.L., Reeves, G., and Beral, V.; Million Women Study collaborators (2011). Height and cancer incidence in the Million Women Study: prospective cohort, and meta-analysis of prospective studies of height and total cancer risk. Lancet Oncol. *12*, 785–794.

45. Samuelsen, S.O., Bakketeig, L.S., Tretli, S., Johannesen, T.B., and Magnus, P. (2006). Head circumference at birth and risk of brain cancer in childhood: a population-based study. Lancet Oncol. *7*, 39–42.

46. McCormack, V.A., dos Santos Silva, I., Koupil, I., Leon, D.A., and Lithell, H.O. (2005). Birth characteristics and adult cancer incidence: Swedish cohort of over 11,000 men and women. Int. J. Cancer *115*, 611–617.

47. dos Santos Silva, I., De Stavola, B., and McCormack, V.; Collaborative Group on Pre-Natal Risk Factors and Subsequent Risk of Breast Cancer (2008). Birth size and breast cancer risk: re-analysis of individual participant data from 32 studies. PLoS Med. *5*, e193.

48. Sandvei, M.S., Lagiou, P., Romundstad, P.R., Trichopoulos, D., and Vatten, L.J. (2015). Size at birth and risk of breast cancer: update from a prospective population-based study. Eur. J. Epidemiol. *30*, 485–492.

49. McCormack, V.A., dos Santos Silva, I., De Stavola, B.L., Mohsen, R., Leon, D.A., and Lithell, H.O. (2003). Fetal growth and subsequent risk of breast cancer: results from long term follow up of Swedish cohort. BMJ *326*, 248. https://doi.org/10.1136/bmj.326.7383.248.

50. Vatten, L.J., Nilsen, T.I.L., Tretli, S., Trichopoulos, D., and Romundstad, P.R. (2005). Size at birth and risk of breast cancer: prospective population-based study. Int. J. Cancer *114*, 461–464. https://doi.org/10.1002/ijc.20726.

51. Winkler, T.W., Day, F.R., Croteau-Chonka, D.C., Wood, A.R., Locke, A.E., Mägi, R., Ferreira, T., Fall, T., Graff, M., Justice, A.E., et al. (2014). Quality control and conduct of genome-wide association meta-analyses. Nat. Protoc. *9*, 1192–1212.

52. Willer, C.J., Li, Y., and Abecasis, G.R. (2010). METAL: fast and efficient meta-analysis of genomewide association scans. Bioinformatics *26*, 2190–2191.

53. Bulik-Sullivan, B.K., Loh, P.R., Finucane, H.K., Ripke, S., Yang, J., Schizophrenia Working Group of the Psychiatric Genomics Consortium; Patterson, N., Daly, M.J., Price, A.L., and Neale, B.M. (2015). LD Score regression distinguishes confounding from polygenicity in genome-wide association studies. Nat. Genet. *47*, 291–295.

54. Pruim, R.J., Welch, R.P., Sanna, S., Teslovich, T.M., Chines, P.S., Gliedt, T.P., Boehnke, M., Abecasis, G.R., and Willer, C.J. (2010). LocusZoom: regional visualization of genome-wide association scan results. Bioinformatics *26*, 2336–2337.

55. Watanabe, K., Taskesen, E., van Bochoven, A., and Posthuma, D. (2017). Functional mapping and annotation of genetic associations with FUMA. Nat. Commun. *8*, 1826.

56. Gusev, A., Ko, A., Shi, H., Bhatia, G., Chung, W., Penninx, B.W.J.H., Jansen, R., de Geus, E.J.C., Boomsma, D.I., Wright, F.A., et al. (2016). Integrative approaches for large-scale transcriptome-wide association studies. Nat. Genet. *48*, 245–252.

57. Quinodoz, M., Royer-Bertrand, B., Cisarova, K., Di Gioia, S.A., Superti-Furga, A., and Rivolta, C. (2017). DOMINO: Using Machine Learning to

Predict Genes Associated with Dominant Disorders. Am. J. Hum. Genet. *101*, 623–629.

58. Amberger, J.S., Bocchini, C.A., Schiettecatte, F., Scott, A.F., and Hamosh, A. (2015). OMIM.org: Online Mendelian Inheritance in Man (OMIM(R)), an online catalog of human genes and genetic disorders. Nucleic Acids Res. *43*, D789–D798.

59. Psaty, B.M., O'Donnell, C.J., Gudnason, V., Lunetta, K.L., Folsom, A.R., Rotter, J.I., Uitterlinden, A.G., Harris, T.B., Witteman, J.C.M., and Boerwinkle, E.; CHARGE Consortium (2009). Cohorts for Heart and Aging Research in Genomic Epidemiology (CHARGE) Consortium: Design of prospective meta-analyses of genome-wide association studies from 5 cohorts. Circ. Cardiovasc. Genet. *2*, 73–80.

60. Thompson, P.M., Stein, J.L., Medland, S.E., Hibar, D.P., Vasquez, A.A., Renteria, M.E., Toro, R., Jahanshad, N., Schumann, G., Franke, B., et al. (2014). The ENIGMA Consortium: large-scale collaborative analyses of neuroimaging and genetic data. Brain Imaging Behav. *8*, 153–182.

61. Jorgensen, J.B., Paridon, E., and Quaade, F. (1961). The correlation between external cranial volume and brain volume. Am. J. Phys. Anthropol. *19*, 317–320. https://doi.org/10.1002/ajpa.1330190402.

62. Chauhan, G., Adams, H.H.H., Bis, J.C., Weinstein, G., Yu, L., Töglhofer, A.M., Smith, A.V., van der Lee, S.J., Gottesman, R.F., Thomson, R., et al. (2015). Association of Alzheimer's disease GWAS loci with MRI markers of brain aging. Neurobiol. Aging *36*, 1765.e7–1765.e16.

63. Giambartolomei, C., Vukcevic, D., Schadt, E.E., Franke, L., Hingorani, A.D., Wallace, C., and Plagnol, V. (2014). Bayesian test for colocalisation between pairs of genetic association studies using summary statistics. PLoS Genet. *10*, e1004383. https://doi.org/10.1371/journal.pgen.1004383.

64. Dong, S., and Boyle, A.P. (2019). Predicting functional variants in enhancer and promoter elements using RegulomeDB. Hum. Mutat. *40*, 1292–1298. https://doi.org/10.1002/humu.23791.

65. Bulik-Sullivan, B., Finucane, H.K., Anttila, V., Gusev, A., Day, F.R., Loh, P.R., ReproGen Consortium; Psychiatric Genomics Consortium; Genetic Consortium for Anorexia Nervosa of the Wellcome Trust Case Control Consortium 3; and Duncan, L., et al. (2015). An atlas of genetic correlations across human diseases and traits. Nat. Genet. *47*, 1236–1241.

66. Yengo, L., Sidorenko, J., Kemper, K.E., Zheng, Z., Wood, A.R., Weedon, M.N., Frayling, T.M., Hirschhorn, J., Yang, J., and Visscher, P.M.; GIANT Consortium (2018). Meta-analysis of genome-wide association studies for height and body mass index in approximately 700000 individuals of European ancestry. Hum. Mol. Genet. *27*, 3641–3649.

67. van der Lee, S.J., Knol, M.J., Chauhan, G., Satizabal, C.L., Smith, A.V., Hofer, E., Bis, J.C., Hibar, D.P., Hilal, S., van den Akker, E.B., et al. (2019). A genome-wide association study identifies genetic loci associated with specific lobar brain volumes. Commun. Biol. *2*, 285.

68. Vojinovic, D., Adams, H.H., Jian, X., Yang, Q., Smith, A.V., Bis, J.C., Teumer, A., Scholz, M., Armstrong, N.J., Hofer, E., et al. (2018). Genome-wide association study of 23,500 individuals identifies 7 loci associated with brain ventricular volume. Nat. Commun. *9*, 3945.

69. Hofer, E., Roshchupkin, G.V., Adams, H.H.H., Knol, M.J., Lin, H., Li, S., Zare, H., Ahmad, S., Armstrong, N.J., Satizabal, C.L., et al. (2019). Genetic Determinants of Cortical Structure (Thickness, Surface Area and Volumes) among Disease Free Adults in the CHARGE Consortium. Preprint at bioRxiv. https://doi.org/10.1101/409649.

70. Hibar, D.P., Adams, H.H.H., Jahanshad, N., Chauhan, G., Stein, J.L., Hofer, E., Renteria, M.E., Bis, J.C., Arias-Vasquez, A., Ikram, M.K., et al. (2017). Novel genetic loci associated with hippocampal volume. Nat. Commun. *8*, 13624.

71. Lee, J.J., Wedow, R., Okbay, A., Kong, E., Maghzian, O., Zacher, M., Nguyen-Viet, T.A., Bowers, P., Sidorenko, J., Karlsson Linnér, R., et al. (2018). Gene discovery and polygenic prediction from a genome-wide association study of educational attainment in 1.1 million individuals. Nat. Genet. *50*, 1112–1121.

72. Davies, G., Lam, M., Harris, S.E., Trampush, J.W., Luciano, M., Hill, W.D., Hagenaars, S.P., Ritchie, S.J., Marioni, R.E., Fawns-Ritchie, C., et al. (2018). Study of 300,486 individuals identifies 148 independent genetic loci influencing general cognitive function. Nat. Commun. *9*, 2098.

73. Malik, R., Chauhan, G., Traylor, M., Sargurupremraj, M., Okada, Y., Mishra, A., Rutten-Jacobs, L., Giese, A.K., van der Laan, S.W., Gretarsdottir, S., et al. (2018). Multiancestry genome-wide association study of 520,000 subjects identifies 32 loci associated with stroke and stroke subtypes. Nat. Genet. *50*, 524–537.

74. Lambert, J.C., Ibrahim-Verbaas, C.A., Harold, D., Naj, A.C., Sims, R., Bellenguez, C., DeStafano, A.L., Bis, J.C., Beecham, G.W., Grenier-Boley, B., et al. (2013). Meta-analysis of 74,046 individuals identifies 11 new susceptibility loci for Alzheimer's disease. Nat. Genet. *45*, 1452–1458.

75. Ferrari, R., Hernandez, D.G., Nalls, M.A., Rohrer, J.D., Ramasamy, A., Kwok, J.B.J., Dobson-Stone, C., Brooks, W.S., Schofield, P.R., Halliday, G.M., et al. (2014). Frontotemporal dementia and its subtypes: a genome-wide association study. Lancet Neurol. *13*, 686–699.

76. Nalls, M.A., Blauwendraat, C., Vallerga, C.L., Heilbron, K., Bandres-Ciga, S., Chang, D., Tan, M., Kia, D.A., Noyce, A.J., Xue, A., et al. (2019). Expanding Parkinson's disease genetics: novel risk loci, genomic context, causal insights and heritable risk. Preprint at bioRxiv. https://doi.org/10.1101/388165.

77. Duncan, L., Yilmaz, Z., Gaspar, H., Walters, R., Goldstein, J., Anttila, V., Bulik-Sullivan, B., Ripke, S., Eating Disorders Working Group of the Psychiatric Genomics Consortium; and Thornton, L., et al. (2017). Significant Locus and Metabolic Genetic Correlations Revealed in Genome-Wide Association Study of Anorexia Nervosa. Am. J. Psychiatr. *174*, 850–858.

78. Demontis, D., Walters, R.K., Martin, J., Mattheisen, M., Als, T.D., Agerbo, E., Baldursson, G., Belliveau, R., Bybjerg-Grauholm, J., Bækvad-Hansen, M., et al. (2019). Discovery of the first genome-wide significant risk loci for attention deficit/hyperactivity disorder. Nat. Genet. *51*, 63–75.

79. Grove, J., Ripke, S., Als, T.D., Mattheisen, M., Walters, R.K., Won, H., Pallesen, J., Agerbo, E., Andreassen, O.A., Anney, R., et al. (2019). Identification of common genetic risk variants for autism spectrum disorder. Nat. Genet. *51*, 431–444.

80. Bipolar Disorder and Schizophrenia Working Group of the Psychiatric Genomics Consortium (2018). Genomic Dissection of Bipolar Disorder and Schizophrenia, Including 28 Subphenotypes. Cell *173*, 1705–1715.e16.

81. van den Berg, S.M., de Moor, M.H.M., Verweij, K.J.H., Krueger, R.F., Luciano, M., Arias Vasquez, A., Matteson, L.K., Derringer, J., Esko, T., Amin, N., et al. (2016). Meta-analysis of Genome-Wide Association Studies for Extraversion: Findings from the Genetics of Personality Consortium. Behav. Genet. *46*, 170–182.

82. Hammerschlag, A.R., Stringer, S., de Leeuw, C.A., Sniekers, S., Taskesen, E., Watanabe, K., Blanken, T.F., Dekker, K., Te Lindert, B.H.W., Wassing, R., et al. (2017). Genome-wide association analysis of insomnia complaints identifies risk genes and genetic overlap with psychiatric and metabolic traits. Nat. Genet. *49*, 1584–1592.

83. Wray, N.R., Ripke, S., Mattheisen, M., Trzaskowski, M., Byrne, E.M., Abdellaoui, A., Adams, M.J., Agerbo, E., Air, T.M., Andlauer, T.M.F., et al. (2018). Genome-wide association analyses identify 44 risk variants and refine the genetic architecture of major depression. Nat. Genet. *50*, 668–681.

84. Turley, P., Walters, R.K., Maghzian, O., Okbay, A., Lee, J.J., Fontana, M.A., Nguyen-Viet, T.A., Wedow, R., Zacher, M., Furlotte, N.A., et al. (2018). Multi-trait analysis of genome-wide association summary statistics using MTAG. Nat. Genet. *50*, 229–237.

85. International Obsessive Compulsive Disorder Foundation Genetics Collaborative (IOCDF-GC) and OCD Collaborative Genetics Association Studies (OCGAS) (2018). Revealing the complex genetic architecture of obsessive-compulsive disorder using meta-analysis. Mol. Psychiatr. *23*, 1181–1188. https://doi.org/10.1038/mp.2017.154.

86. Ritchie, M.E., Phipson, B., Wu, D., Hu, Y., Law, C.W., Shi, W., and Smyth, G.K. (2015). limma powers differential expression analyses for RNA-sequencing and microarray studies. Nucleic Acids Res. *43*, e47.

87. Bryois, J., Skene, N.G., Hansen, T.F., Kogelman, L.J.A., Watson, H.J., Liu, Z., Eating Disorders Working Group of the Psychiatric Genomics Consortium; International Headache Genetics Consortium; 23andMe Research Team; and Brueggeman, L., et al. (2020). Genetic identification of cell types underlying brain complex traits yields insights into the etiology of Parkinson's disease. Nat. Genet. *52*, 482–493.

88. Finucane, H.K., Bulik-Sullivan, B., Gusev, A., Trynka, G., Reshef, Y., Loh, P.R., Anttila, V., Xu, H., Zang, C., Farh, K., et al. (2015). Partitioning heritability by functional annotation using genome-wide association summary statistics. Nat. Genet. *47*, 1228–1235.

89. Liang, D., Elwell, A.L., Aygün, N., Lafferty, M.J., Krupa, O., Cheek, K.E., Courtney, K.P., Yusupova, M., Garrett, M.E., Ashley-Koch, A., et al. (2020). Cell-type specific effects of genetic variation on chromatin accessibility during human neuronal differentiation. Preprint at bioRxiv. https://doi.org/10.1101/2020.01.13.904862.

STAR★METHODS

KEY RESOURCES TABLE

REAGENT or RESOURCE	SOURCE	IDENTIFIER
Deposited data		
Genome-wide association study summary statistics	CHARGE dbGaP and http://enigma.ini.usc.edu/research/download-enigma-gwas-results	phs000930 (dbGaP accession number)
Software and algorithms		
EasyQC	Winkler et al.[51]	Software - Universität Regensburg (uni-regensburg.de)
METAL	Willer et al.[52]	METAL Documentation - Genome Analysis Wiki (umich.edu)
LD score regression	Bulik-Sullivan et al.[53]	GitHub - bulik/ldsc: LD Score Regression (LDSC)
LocusZoom	Pruim et al.[54]	LocusZoom - Create Plots of Genetic Data
FUMA GWAS	Watanabe et al.[55]	Functional Mapping and Annotation of Genome-wide association studies (ctglab.nl)
TWAS-Fusion	Gusev et al.[56]	TWAS/FUSION (gusevlab.org)
DOMINO	Quinodoz et al.[57]	Domino (iob.ch)
Other		
OMIM database	Amberger et al.[58]	Home - OMIM
Cortical organoids' scRNA-seq data	Bhaduri et al.[34]	https://organoidreportcard.cells.ucsc.edu
e/sQTLs, and allele-specific expression in cultured primary human neural progenitors and their sorted neuronal progeny	Aygün et al.[32]	https://bitbucket.org/steinlabunc/expression_splicing_qtls_public/src/master/

RESOURCE AVAILABILITY

Lead contact

Further information and requests for resources and reagents should be directed to and will be fulfilled by the lead contact, Hieab H.H. Adams (Hieab.Adams@radboudumc.nl).

Materials availability

This study did not generate new unique reagents.

Data and code availability

The genome-wide summary statistics that support the findings of this study will be made available through the CHARGE dbGaP (accession number phs000930) and ENIGMA (http://enigma.ini.usc.edu/research/download-enigma-gwas-results) websites.

No previously unreported custom computer code or mathematical algorithm was used to generate results central to the conclusions.

Any additional information required to re-analyse the data reported in this work paper is available from the lead contact upon request.

EXPERIMENTAL MODEL AND SUBJECT DETAILS

Study population

Most studies participate in the Cohorts for Heart and Aging Research in Genomic Epidemiology (CHARGE)[59] or the Enhancing NeuroImaging Genetics through Meta-Analysis (ENIGMA)[60] consortium. We also included the results of the most recent head

circumference GWAS.[5] A complete overview of the included studies is shown in Table S1 and their population characteristics are presented in Table S2. Each contributing study was approved by their institutional review boards or local ethical committees. Written informed consent was obtained from all study participants.

Genotyping

Genotyping of individuals was performed on commercially available arrays, and imputed to 1000 Genomes (1KG) or Haplotype Reference Consortium (HRC) imputation panels (Table S3). Quality control was performed using the EasyQC software.[51] In each study, genetic variants with an imputation quality r^2 below 0.3 and a minor allele frequency (MAF) below 0.001 were excluded. Additionally, variants were filtered on study level requiring $(r^2\ x\ MAF\ x\ N) > 5$.

Phenotyping

Different methods were used to measure human head size across studies. Briefly, either head circumference was measured, or intracranial volume was measured on computed tomography (CT) or magnetic resonance imaging (MRI) scans. In total, human head size was measured using intracranial volume measured on CT or MRI scans in respectively 1,283 and 84,171 individuals, and using head circumference in 20,524 individuals (Table S4). These measures have previously shown to be phenotypically and genetically correlated,.[5,6,61] Genetic correlations between our MRI scans and head circumference measurements was 0.75. Together, this allowed us to perform a combined meta-analyse of different measures of head size.

METHOD DETAILS

Genome-wide association studies

GWAS were performed for each study adjusted for age, age^2 (if significant), gender, eigenstrat PC1-4 (if significant), study-specific adjustments and case-control status (if applicable). In a second model, additional adjustment for height were made. The METAL software[52] was used to perform a sample size weighted *Z* score meta-analysis. After meta-analysis, genetic variants available in less than 5,000 individuals were excluded. Comparable betas were derived using the formula $Zscore\ x\ \sqrt{\frac{1}{N\ x\ 2\ x\ MAF}}$ as was done previously.[62] Genomic inflation and polygenic heterogeneity were assessed using the LD score regression software[53] by comparing the genomic control inflation factor and the LD score regression intercept (Table S5).

GWAS meta-analyses were performed separately for African, Asian and European samples. We also performed a transancestral meta-analysis. Since the analyses in non-European samples were underpowered, we additionally used an inverse-variance weighted method to test the combined effects of the lead variants in the non-European samples. This analysis was performed using the gtx package as implemented in R.

Functional annotations

Regional association plots were made with the LocusZoom software.[54] The Functional Mapping and Annotation of Genome-Wide Association Studies (FUMA GWAS) platform[55] was used to derive the independent genomic loci and genetic lead variants, and to functionally annotate the identified genetic variants. Additionally, enrichment for KEGG[9] biological pathways was assessed for genes located nearby the identified genetic loci using the default options in FUMA, using hypergeometric tests. Genotype-Tissue Expression (GTEx) v7 was used to identify expression quantitative trait loci (eQTL) for the lead genetic variants and variants in LD ($r^2 > 0.6$).

We performed a transcriptome-wide association study (TWAS) using the association statistics from the European-only head size GWAS summary statistics and weights from 21 publicly available gene expression reference panels. We focused on the gene expression weights from blood (Young Finns Study, YFS), arterial (GTEx), brain (GTEx, CommonMind Consortium (CMC)) and peripheral nerve tissues (GTEx). Precomputed SNP-expression weights in the 1-Mb window were obtained for each gene in the reference panel, including the highly-tissue specific splicing QTL (sQTL) information on gene isoforms in the dorsolateral prefrontal cortex (DLPFC, CMC). Using the SNP-expression weights, SNP-trait effect estimates and the SNP correlation matrix, we used the TWAS-Fusion[56] to estimate the association statistic between the predicted expression and head size (TWAS *Z* score). Transcriptome-wide significant genes (eGenes) and the corresponding QTLs (eQTLs) were determined using Bonferroni correction in each reference panel, based on the average number of features (4,320 genes) tested across all the reference panels.[56] Finally, using a prior association probability of 1.1×10^{-5} and colocalization analysis (COLOC)[63] for each locus we estimated the posterior probability of a shared causal variant (PP4>0.75) between the gene expression and trait association. eGene regions with eQTLs not reaching genome-wide significance in the head size GWAS were considered putatively novel TWAS signals. Furthermore, functional validation of the eGenes was performed by integrating eQTL with the functional genomics feature from the RegulomeDB.[64] A RegulomeDB probability score greater than 0.5 and closer to 1 indicates the likelihood of the eQTL having a gene-regulatory role. Finally, accounting for pairwise correlation between the gene expression features we conducted the multiple degree of freedom omnibus analysis, to test for the shared effect of eGenes across the different gene reference panels. A significance threshold of $p < 3.48 \times 10^{-6}$ accounting for the number of genes (N = 14,385) tested was used to identify significant eGenes in the omnibus test.

Effects on anthropomorphic measures and regional brain volumes

The LD score regression software[53,65] was used to assess genetic correlations with adult height,[66] for both the height-unadjusted and height-adjusted model.

Dual-energy X-ray absorptiometry (DXA) measurements of the UK Biobank imaging subsample (*N* = 3,313) were used to examine the effect of the identified lead variants on anthropometric measures across the body, i.e., bone area of the arms, legs, pelvis, ribs, spine, trunk and vertebrae L1-L4. In these analyses values more than three standard deviations from the mean were considered outliers and removed from the analyses. We adjusted for age, age,^{2} gender and principal components (model 1), and additionally for height (model 2) to correct for an overall growth effect.

To investigate the effects of the identified variants for head size on growth in specific brain regions, we investigated the overlap between the identified loci for head size and previous genome-wide association studies (GWASs) on brain volumes.[7,67–70] We also analyzed the associations between the identified lead genetic variants and global volumes (i.e., four brain lobes and lateral ventricle volumes), subcortical volumes (i.e., volumes of eight subcortical structures) and cortical volumes (i.e., volumes of 34 cortical regions of interest) in the UK Biobank (*N* = 22,145). Volumes were derived using the FreeSurfer 6.0 software. Values more than 3.5 standard deviations away from the mean were considered outliers and removed from the analysis. In the first model, we adjusted for age, age,^{2} gender and principal components, and in the second model additionally for intracranial volume.

Additionally, we took the lead variants specifically associated with one or two subcortical volumes, and investigated their effects on the shape of seven subcortical structures, i.e., amygdala, caudate nucleus, hippocampus, nucleus accumbens, pallidum, putamen and thalamus. The radial distances and log Jacobian determinants were derived using the ENIGMA-Shape package (http://enigma.usc.edu/ongoing/enigma-shape-analysis/). Volumetric outliers more than 3.5 standard deviations from the mean were removed from the analysis.

We performed 10,000 permutations to define the number of independent DXA, brain volumetric and subcortical shape outcomes. We used this number to define our multiple testing adjusted *p* value thresholds for significance, i.e., 0.05/(number of independent outcomes x number of lead genetic variants).

Genetic correlations

We investigated the genetic correlations with neuropsychiatric traits using the LD score regression software.[53,65] Genetic correlation analyses were performed for educational attainment,[71] general cognitive function,[72] all stroke,[73] Alzheimer's disease,[74] frontotemporal dementia,[75] Parkinson's disease,[76] anorexia nervosa,[77] attention-deficit hyperactivity disorder,[78] autism spectrum disorder,[79] bipolar disorder,[80] extraversion,[81] insomnia,[82] major depressive disorder,[83] neuroticism,[84] obsessive compulsive disorder[85] and schizophrenia.[80] Analyses were performed in the entire GWAS dataset as well as in the GWAS set with newly included studies in comparison to the intracranial volume GWAS performed by Adams et al.[6]

We also performed genetic correlation analyses for publicly available cancer GWAS, namely for breast cancer,[28] ovarian cancer[29] and prostate cancer.[30] To obtain information on more cancer types, we additionally included GWAS of cancer registries from the UK Biobank and Kaiser Permanente Genetic Epidemiology Research on Adult Health and Aging (GERA).[31] Of those, we excluded cancer types with less than 1,000 cases, which left the following cancer types to be analyzed: bladder cancer (N_{cases} = 2,242), breast cancer (N_{cases} = 17,881), cervical cancer (N_{cases} = 6,563), colon cancer (N_{cases} = 3,793), endometrial cancer (N_{cases} = 2,037), esophageal/gastric cancer (N_{cases} = 1,091), kidney cancer (N_{cases} = 1,338), lung cancer (N_{cases} = 2,485), malignant melanoma (N_{cases} = 6,777), non-Hodgkin's lymphoma (N_{cases} = 2,400), prostate cancer (N_{cases} = 10,792) and rectal cancer (N_{cases} = 2,091). Genetic correlations with oral cavity/pharyngeal cancer (N_{cases} = 1,223) and ovarian cancer (N_{cases} = 1,259) could not be calculated due to low heritability estimates.

Enrichment analyses

We performed enrichment analyses of different gene sets: genes within 1 Mb, 100 kb or 10 kb of the identified genetic loci, genes within 10 kb of the identified genetic loci with intragenic genetic variants, and genes within 10 kb of the identified genetic loci with intragenic genetic lead variants. As a reference, we used the rest of the protein-coding genome.

First, the Online Mendelian Inheritance in Man (OMIM) database[58] was used to retrieve information on genes related to heritable phenotypes affecting head size (Tables S19 and S20). Second, the COSMIC database[27] was used to extract Tier 1 cancer genes. Taking the rest of the genome as our reference gene set, we calculated the enrichment of these macrocephaly, microcephaly and cancer genes in the abovementioned gene sets.

Lastly, DOMINO,[57] a previously developed machine learning tool, was used to assess if the genes in the different gene sets were more often predicted to harbor dominant changes in comparison with genes in the rest of the genome.

Mean autosomal dominance scores were compared with the reference genome using a Mann-Whitney test. Differences in the proportions for the OMIM macro- and microcephaly genes, intellectual disability genes and COSMIC genes were calculated using a Pearson's χ^2 test.

We performed these analyses for the head size height-unadjusted GWAS results, but also the GWAS in the subset of studies for which height was available, the height-adjusted GWAS and the height GWAS.[66] For comparison, we also selected the top 67 loci for the height GWAS, so the results were not driven by a difference in the number of associated loci.

Experimental datasets of brain cells

To assess whether the identified genes in the current study are enriched for genes differentially expressed in human progenitors versus neurons, we utilized differential gene expression data of those cell lines, derived from a previously published sample population ($N_{donor} = 85$ in progenitors and $N_{donor} = 74$ in neurons).[32] Using genes with at least 10 counts in more than 5% of the cell-type specific donors in either cell-type (resulting in 16,172 protein-coding genes out of 28,785 genes in total), we performed a paired differential gene expression analysis with design matrix: model.matrix(~ CellType + as.factor(DonorID) + RIN, data) as described previously,[32] using the limma R package.[86] We detected 1,095/1,420 protein genes upregulated in progenitors/neurons, respectively, for abs(logFC) > 1.5 and adjusted p value < 0.05. Performing a hypergeometric test, we evaluated if multiple protein-coding gene sets: head size gene sets with different distances from the lead variants, OMIM macrocephaly and microcephaly genes, and COSMIC tier 1 cancer genes are enriched among the protein-coding genes upregulated in progenitors or neurons.

Using a different approach, scRNA-seq data were used to investigate whether our genes of interest were enriched for genes specific for certain cortical brain cell types. Specifically, scRNA-seq data from the developing human cortex (gestational week 6–22, more than 189,000 cells) were used to identify the top 10% of genes specific for a certain cell type.[34] Using this data, we first performed LD score regression[53] based enrichment analyses of the head size GWAS summary statistics, as previously described.[33,87] Gene specificity was defined as the ratio of expression of a gene in a cell type by the total expression of that gene in all cell types. In parallel, we again tested the enrichment of various gene sets: head size gene sets with different distances from the lead variants, OMIM macrocephaly and microcephaly genes, and COSMIC tier 1 cancer genes, with the top 10% of cell specific genes for each cell type using hypergeometric tests. FDR correction was used to correct for the multiple gene sets tested for enrichment in each cell type.

To determine if regulatory elements of neural progenitors are enriched for the heritability of head size, we performed partitioned heritability analyses[53,88] using chromatin accessibility profiles from a population of 76 primary human neural progenitor cells and 61 of their differentiated neuronal progenies, as was done previously.[89]

QUANTIFICATION AND STATISTICAL ANALYSIS

Please see the statistical analyses and software in method details.

Cell Reports Medicine, Volume *5*

Supplemental information

Genetic variants for head size share

genes and pathways with cancer

Maria J. Knol, Raymond A. Poot, Tavia E. Evans, Claudia L. Satizabal, Aniket Mishra, Muralidharan Sargurupremraj, Sandra van der Auwera, Marie-Gabrielle Duperron, Xueqiu Jian, Isabel C. Hostettler, Dianne H.K. van Dam-Nolen, Sander Lamballais, Mikolaj A. Pawlak, Cora E. Lewis, Amaia Carrion-Castillo, Theo G.M. van Erp, Céline S. Reinbold, Jean Shin, Markus Scholz, Asta K. Håberg, Anders Kämpe, Gloria H.Y. Li, Reut Avinun, Joshua R. Atkins, Fang-Chi Hsu, Alyssa R. Amod, Max Lam, Ami Tsuchida, Mariël W.A. Teunissen, Nil Aygün, Yash Patel, Dan Liang, Alexa S. Beiser, Frauke Beyer, Joshua C. Bis, Daniel Bos, R. Nick Bryan, Robin Bülow, Svenja Caspers, Gwenaëlle Catheline, Charlotte A.M. Cecil, Shareefa Dalvie, Jean-François Dartigues, Charles DeCarli, Maria Enlund-Cerullo, Judith M. Ford, Barbara Franke, Barry I. Freedman, Nele Friedrich, Melissa J. Green, Simon Haworth, Catherine Helmer, Per Hoffmann, Georg Homuth, M. Kamran Ikram, Clifford R. JackJr., Neda Jahanshad, Christiane Jockwitz, Yoichiro Kamatani, Annchen R. Knodt, Shuo Li, Keane Lim, W.T. Longstreth, Fabio Macciardi, The Cohorts for Heart and Aging Research in Genomic Epidemiology (CHARGE) Consortium, The Enhancing NeuroImaging Genetics through Meta-Analysis (ENIGMA) Consortium, Outi Mäkitie, Bernard Mazoyer, Sarah E. Medland, Susumu Miyamoto, Susanne Moebus, Thomas H. Mosley, Ryan Muetzel, Thomas W. Mühleisen, Manabu Nagata, Soichiro Nakahara, Nicholette D. Palmer, Zdenka Pausova, Adrian Preda, Yann Quidé, William R. Reay, Gennady V. Roshchupkin, Reinhold Schmidt, Pamela J. Schreiner, Kazuya Setoh, Chin Yang Shapland, Stephen Sidney, Beate St Pourcain, Jason L. Stein, Yasuharu Tabara, Alexander Teumer, Anne Uhlmann, Aad van der Lugt, Meike W. Vernooij, David J. Werring, B. Gwen Windham, A. Veronica Witte, Katharina Wittfeld, Qiong Yang, Kazumichi Yoshida, Han G. Brunner, Quentin Le Grand, Kang Sim, Dan J. Stein, Donald W. Bowden, Murray J. Cairns, Ahmad R. Hariri, Ching-Lung Cheung, Sture Andersson, Arno Villringer, Tomas Paus, Sven Cichon, Vince D. Calhoun, Fabrice Crivello, Lenore J. Launer, Tonya White, Peter J. Koudstaal, Henry Houlden, Myriam Fornage, Fumihiko Matsuda, Hans J. Grabe, M. Arfan Ikram, Stéphanie Debette, Paul M. Thompson, Sudha Seshadri, and Hieab H.H. Adams

Table of Contents

Supplementary Tables 2

Table S1. List of all contributing studies. *Related to STAR Methods' section "Study Population".* ... 2

Table S2. Population characteristics of new or updated contributing studies. *Related to STAR Methods' section "Study Population".* 5

Table S3. Information on genotyping and quality control. *Related to STAR Methods' section "Genotyping".* 7

Table S5. Lambda genomic control, LD score regression intercept and ratio, and SNP-based heritability for different models. *Related to STAR Methods' section "Genome-wide association studies".* 10

Table S10. Overlap between identified loci and previously identified loci in genome-wide association studies of brain volumes. *Related to Figure 2A.* 11

Table S14. Genetic correlation between the human head size and neuropsychiatric traits. *Related to Figure S3, Figure 1C and STAR Methods' section "Genetic correlations".* 14

Table S15. Kyoto Encyclopaedia of Genes and Genomes (KEGG) pathway analysis. *Related to Figure 3A.* 15

Table S22. Genetic correlation between the human head size and different cancer types. *Related to STAR Methods' section "Genetic correlations".* 18

Table S24. Enrichment of head size GWAS for brain cell types based on human scRNA-seq data. *Related to Figure 4E.* 19

Table S25. Enrichment of gene sets for brain cell types based on human scRNA-seq data. *Related to Figure 4E.* 21

Supplementary Figures 24

Figure S1. Genetic correlations with neuropsychiatric traits. *Related to Figure 1C.* 24

Figure S2. Enrichment of expression of human head size GWAS genes in cell subtypes in the human cortical brain, derived from single-cell RNA sequencing data. *Related to Figure 4.* 25

Supplementary Tables

Table S1. List of all contributing studies. *Related to STAR Methods' section "Study Population".*

**Study**
1000Brains
3C-Bordeaux
3C-Dijon
AddNeuroMed
ADNI
ADNI2GO
AGES
ALSPAC
ARIC-Black
ARIC-White
ASPS
ASPSFam
ASRB
Betula
BFS
BIG
BIL&GIN
BrainSCALE
BRCDECC
CARDIA-Black
CARDIA-White
CHAP
CHAP-Black
CHS
COPSAC2000
COPSAC2010
CROATIA-KORCULA
CROMIS-2 ICH
DHS
DNS
EDIS-SCES
EDIS-SiMES
EPIGEN
EPOZ
ERF
ESS
FBIRN
FHS
GeneSTAR

GenR
GIG
GSP
HKOS
HUBIN
HUNT
IMAGEN
IMH
INMA
i-Share
LBC1936
LIFE-Adult
LLS
MCIC
Meth-CT
MooDS
MPIP
Nagahama
NCNG
NESDA
neuroIMAGE
NOMAS
NOMAS-Black
NOMAS-Hispanic
NTR - Adults
OATS
ORCADES
PAFIP
Poznan MS
PROSPER
QTIM
RAINE
ROSMAP1
ROSMAP2
RSI
RSII
RSIII
SHIP
SHIP-TREND
Sydney MAS
SYS adolescents
SYS adults
TOP
UK Biobank
UMCU

VIDI
VIKING
WHICAP
WHICAP-Black

Table S2. Population characteristics of new or updated contributing studies. *Related to STAR Methods' section "Study Population".*

Cohort	Study Design	Ancestry	Total N	N Females	Mean Age (SD)	Age Range
**European samples**						
**1000 Brains**	Population-based	European	751	343	66.8 (6.8)	53 - 85
**3C-Bordeaux**	Population-based	European	564	323	72.7 (4.0)	65 - 82
**ARIC whites**	Population-based	European	1390	812	76.3 (5.3)	65 - 90
**ASRB**	Case-control (SCZ and healthy controls)	European	233	95	38.5 (11.3)	19 - 64
**CARDIA whites**	Population-based	European	349	185	51.0 (3.2)	42 - 56
**CROMIS-2 ICH**	Population-based	European	642	270	75.7 (10.8)	41-100
**DHS**	Family-based study	European	460	258	60.6 (9.0)	33 - 82
**DNS**	Population-based	European	516	275	19.8 (1.24)	18 - 22
**EPOZ**	Population-based	European	251	144	69.4 (6.0)	60 - 86
**ESS**	Case-only (TIA and stroke cases)	European	641	293	62.6 (13.8)	17 - 93
**FHS (update - previous + additional samples)**	Population-based	European	4710	2519	56.9 (13.0)	26 - 97
**GenR (additional samples)**	Population-based	European	265	139	10.3 (0.7)	9 - 12
**HUNT**	Population-based	European	903	478	58.9 (4.2)	50 - 66
**LIFE-Adult**	Population-based	European	1825	890	63.2 (11.3)	20 - 82
**Poznan MS**	Case-only (Multiple sclerosis)	European	204	142	34.0 (7.9)	18 - 54
**RSI (update - previous + additional samples)**	Population-based	European	1103	635	79.1 (6.0)	70 - 100
**RSII (update - previous + additional samples)**	Population-based	European	1159	603	69.6 (7.1)	60 - 100
**RSIII (update - previous + additional samples)**	Population-based	European	2582	1427	57.3 (6.9)	50 - 90
**SHIP-TREND (additional samples)**	Population-based	European	1084	530	51.7 (14.2)	21-82

**SYS adolescents**	Family-based study	European	980	506	15.0 (1.8)	12 - 19
**SYS adults**	Family-based study	European	594	320	49.3 (5.0)	36 - 63
**UK Biobank**	Population-based	European	14571	7592	55.1 (7.4)	40 - 70
**VIDI**	RCT/Population-based	European	755	374	1.99 (0.03)	1 - 2
**Non-European/mixed ancestry samples**						
**ARIC-Black**	Population-based	African-American	675	434	74.3 (5.1)	61 - 89
**CARDIA-Black**	Population-based	African-American	132	75	49.0 (3.6)	43 - 56
**FBIRN**	Case-control (SCZ and healthy controls)	Mixed	275	65	38.9 (11.3)	18 - 62
**HKOS**	Population-based	Southern Chinese	888	709	58.5 (11.5)	32 - 96
**IMH**	Case-control (SCZ and healthy controls)	Singaporean	37	22	28.7 (6.5)	19 - 45
**Meth-CT**	Case-control (addiction, SIPD, healthy controls)	South African	114	25	26.6 (6.4)	18 - 53
**Nagahama**	Population-based	Japanese	2889	1861	68.3 (5.3)	59 - 81
**Validation samples**						
**BIL&GIN**	Population-based	European	265	136	25.5 (6.1)	18 - 53
**i-Share**	Population-based	European	1777	1278	22.1 (2.3)	18 - 35
**UK Biobank (independent sample)**	Population-based	European	23890	12600	65.1 (7.4)	47 - 81

Table S3. Information on genotyping and quality control. *Related to STAR Methods' section "Genotyping".*

Cohort	HWE	MAF	Call Rate	Association software	Imputation	Reference	Genotype Platform
**European samples**							
**1000 Brains**	1x10-6	0.01	0.95	plink 1.9	minimac (release 2013-07-17)	EUR 1000 Genomes (phase 1 version 3; Nov 2010)	Illumina Human OmniExpress12, Human CoreExome12, HumanOmni1-Quad, Infinium OmniExpressExome-8
**3C-Bordeaux**	1x10-6	0.01	0.95	plink 1.9	minimac3	Haplotype Reference Consortium 1.1	Illumina Human 610-Quad BeadChip
**ARIC whites**	1x10-6	0.01	0.95	probABEL	minimac3	Haplotype Reference Consortium 1.1	Affymetrix 6.0
**ASRB**	1x10-6	0.01	0.95	mach2qtl	minimac4	EUR 1000 Genomes (phase 1 version 5; February 18 2015)	Illumina Human610-Quad BeadChip
**CARDIA whites**	1x10-4	0.03	0.95	probABEL	BEAGLE	ALL 1000 Genomes (phase 1 version 3; Nov 2010)	Affymetrix 6.0
**CROMIS-2 ICH**	1x10-8	0.01	0.95	SNPTEST	Michigang Imputation Server	Haplotype Reference Consortium 1.1	Illumina Infinium® GlobalScreeningArray-24v1.0
**DHS**	1x10-6	0.05	0.95	GWAF	IMPUTE2	ALL 1000 Genomes (phase 1 version 3; Nov 2010)	Affymetrix 5.0
**DNS**	1x10-6	0.01	0.9	plink 1.9	IMPUTE2	full 1000 Genomes Project Phase 3 (May 2013, >70 million variants, release "v5a")	Illumina HumanOmniExpress/Illumina HumanOmniExpress-24
**EPOZ**	1x10-4	-	0.975	rvtest	minimac3	Haplotype Reference Consortium 1.1	Infinium Global Screening Array
**ESS**	1x10-7	-	0.975	rvtest	minimac3	Haplotype Reference Consortium 1.1	Metabochip

**FHS**	1x10-6	0.01	0.97	Perl and R	MACH/minimac	EUR 1000 Genomes (phase 1 version 3; March 2012)	Affymetrix 500K (250K Nsp & 250K Sty), MIPS 50K
**GenR**	1x10-6	0.01	0.98	rvtest	minimac3	Haplotype Reference Consortium 1.1	Illumina 610 Quad array
**HUNT**	1x10-6	0.01	0.95	mach2qtl (1.1.2)	minimac (release 2013-07-17)	EUR 1000 Genomes (phase 1 version 3; Nov 2010)	Illumina Omni 2.5M BeadChip array
**LIFE-Adult**	1x10-6	-	0.97	plink 1.9	IMPUTE v2.3.2	1000 Genomes (phase 1 version 3; March 2012)	Affymetrix Axiom-CEU
**Poznan MS**	NA	NA	NA	rvtest	minimac3	Haplotype Reference Consortium 1.1	Illumina HumanOmniExpress/Illumina HumanOmniExpress-24
**RSI**	1x10-6	0.01	0.98	rvtest	minimac3	Haplotype Reference Consortium 1.1	Illumina Human 550 (+duo) and 610-Quad BeadChip
**RSII**	1x10-6	0.01	0.98	rvtest	minimac3	Haplotype Reference Consortium 1.1	Illumina Human 550 duo
**RSIII**	1x10-6	0.01	0.98	rvtest	minimac3	Haplotype Reference Consortium 1.1	Illumina Human610-Quad BeadChip
**SHIP-TREND**	1x10-4	-	0.94	EPACTS	minimac3	Haplotype Reference Consortium 1.1	Illumina GSA-24
**SYS adolescents**	1x10-6	0.01	0.95	probABEL	IMPUTE v2.2.2	EUR 1000 Genomes (phase 1 version 3; March 2012)	Illumina Human610-Quad BeadChip (610K SNPs) & Illumina HumanOmniExpress BeadChip
**SYS adults**	1x10-6	0.01	0.95	probABEL	IMPUTE v2.2.2	EUR 1000 Genomes (phase 1 version 3; March 2012)	Illumina Human610-Quad BeadChip (610K SNPs) & Illumina HumanOmniExpress BeadChip
**UK Biobank**	1x10-6	0.01	0.9	HASE	IMPUTE4	Haplotype Reference Consortium 1.1	Applied Biosystems™ UK BiLEVE Axiom™ Array by Affymetrix and Applied Biosystems™ UK Biobank Axiom™ Array
**VIDI**	-	-	0.95	plink 1.9	IMPUTE2	1000 Genomes (phase 3 version 5)	Illumina Infinium Global Screening Array

**Non-European/mixed ancestry samples**							
**ARIC-Black**	1x10-6	0.01	0.95	probABEL	IMPUTE2 2.2.2	ALL 1000 Genomes (phase 1 version 3; Nov 2010)	Affymetrix 6.0
**CARDIA-Black**	1x10-4	0.03	0.95	probABEL	BEAGLE	ALL 1000 Genomes (phase 1 version 3; Nov 2010)	Affymetrix 6.0
**FBIRN**	1x10-6	0.01	0.95	raremetal/4.14.1	Michigan Imputation	ALL 1000 Genomes (phase 1 version 3; Nov 2010)	Illumina MEGA+Psych chip
**HKOS**	1x10-6	0.01	0.95	GCTA MLMA	Michigan Imputation Server	Haplotype Reference Consortium 1.1	Illumina Human 610-Quad BeadChip & Illumina Global Screening Array
**IMH**	1x10-6	0.01	0.95	rvtest	-	-	Illumina 1M duo3 array
**Meth-CT**	1x10-6	0.01	0.95	mach2qtl (1.1.2)	minimac (release 2013-07-17)	ALL 1000 Genomes (phase 1 version 3; Nov 2010)	Infinium PsychArray-24 v1.1 BeadChip
**Nagahama**	1x10-7	0.01	0.99	rvtest	Michigan Imputation Server	Japanese 3,000 reference panel (AGP)	Human 610K Quad, HumanOmni2.5-4, HumanOmni2.5-8, HumanOmni2.5s, HumanExome and Human core exome, 5.0M and ASA (Illumina, San Diego, CA, USA)
**Validation samples**							
**BIL&GIN**	1x10-6	0.01	0.95	plink 1.9	minimac	Haplotype Reference Consortium 1.1	Illumina InfiniumOmniExpressExome-8v1-4
**i-Share**	1x10-3	0.01	0.98	GCTA MLMA	minimac4	Haplotype Reference Consortium 1.1	Affymetrix Precision Medicine Axiom Array
**UK Biobank (independent sample)**	1x10-6	0.01	0.9	BOLT-LMM	IMPUTE4	Haplotype Reference Consortium version 1.1, UK10K and 1000Genomes	Applied Biosystems™ UK BiLEVE Axiom™ Array by Affymetrix and Applied Biosystems™ UK Biobank Axiom™ Array

Table S5. Lambda genomic control, LD score regression intercept and ratio, and SNP-based heritability for different models. *Related to STAR Methods' section "Genome-wide association studies".*

Model	Lambda.GC	Intercept	SE intercept	Ratio	SE ratio	Heritability	SE heritability
Model 1	1.2865	1.0562	0.0106	0.1315	0.0249	0.2487	0.0164
Model 1 height subset	1.2664	1.0488	0.0093	0.1320	0.0252	0.2935	0.0210
Model 1 new cohorts	1.1715	1.0247	0.0087	0.1031	0.0363	0.2448	0.0198
Model 2	1.2498	1.0461	0.0086	0.1351	0.0252	0.2970	0.0205

Table S10. Overlap between identified loci and previously identified loci in genome-wide association studies of brain volumes.
Related to Figure 2A.
Highest linkage disequilibrium (r2) value with a genome-wide significant genetic variant is presented in brackets.
Abbreviations: HC - head circumference, ICV - intracranial volume.

Genomic Locus	rsID	trait1	trait2	trait3	trait4
6	rs6429430	HC + ICV, Haworth et al. 2019 (r2=1)			
7	rs6736289	HC + ICV, Haworth et al. 2019 (r2=1)			
9	rs62135193	Superiortemporal, Grasby et al. 2020 (r2=1)			
10	rs41288837	HC + ICV, Haworth et al. 2019 (r2=1)			
11	rs867529	Brainstem, Satizabal et al. 2019 (r2=1)			
12	rs288326	HC + ICV, Haworth et al. 2019 (r2=1)	HC, Haworth et al. 2019 (r2=1)		
17	rs2063453	HC + ICV, Haworth et al. 2019 (r2=1)			
18	rs1159211	Accumbens, Satizabal et al. 2019 (r2=1)	Precuneus, Grasby et al. 2020 (r2=1)	Lateral ventricles, Vojinovic et al. 2018 (r2=1)	HC + ICV, Haworth et al. 2019 (r2=0.798)
18	rs9821713	HC + ICV, Haworth et al. 2019 (r2=1)	Accumbens, Satizabal et al. 2019 (r2=1)	Lateral ventricles, Vojinovic et al. 2018 (r2=1)	
23	rs1935952	HC + ICV, Haworth et al. 2019 (r2=1)	Brainstem, Satizabal et al. 2019 (r2=1)		
24	6-126845438	HC + ICV, Haworth et al. 2019 (r2=1)	ICV, Adams et al. 2016 (r2=1)		

24	rs11154343	HC + ICV, Haworth et al. 2019 (r2=1)			
24	rs17650496	HC + ICV, Haworth et al. 2019 (r2=1)	ICV, Adams et al. 2016 (r2=1)		
24	rs190958130	Pericalcarine, Grasby et al. 2020 (r2=1)			
24	rs2011008	HC + ICV, Haworth et al. 2019 (r2=1)	ICV, Adams et al. 2016 (r2=1)		
24	rs4273712	HC + ICV, Haworth et al. 2019 (r2=1)	ICV, Adams et al. 2016 (r2=1)	Caudalmiddlefrontal, Grasby et al. 2020 (r2=1)	
24	rs9401873	ICV, Adams et al. 2016 (r2=1)	HC + ICV, Haworth et al. 2019 (r2=1)		
28	rs34888260	HC + ICV, Haworth et al. 2019 (r2=0.992)			
29	rs2072235	Putamen, Satizabal et al. 2019 (r2=0.654)			
30	rs151057105	Thalamus, Satizabal et al. 2019 (r2=0.991)			
37	rs11012732	Lateral ventricles, Vojinovic et al. 2018 (r2=0.897)			
39	rs1628768	HC + ICV, Haworth et al. 2019 (r2=1)	ICV, Adams et al. 2016 (r2=1)		
42	rs3217870	HC + ICV, Haworth et al. 2019 (r2=1)			
43	rs2066827	HC + ICV, Haworth et al. 2019 (r2=1)			
46	rs17178006	Hippocampus, Hibar et al. 2017 (r2=1)	Amygdala, Satizabal et al. 2019 (r2=1)		
47	rs7306710	HC + ICV, Haworth et al. 2019 (r2=1)			
50	rs11111293	Amygdala, Satizabal et al. 2019 (r2=1)	Brainstem, Satizabal et al. 2019 (r2=0.753)		

51	rs28636834	HC + ICV, Haworth et al. 2019 (r2=1)	HC, Haworth et al. 2019 (r2=1)		
57	rs78378222	HC + ICV, Haworth et al. 2019 (r2=1)	HC, Haworth et al. 2019 (r2=1)		
60	rs4564621	ICV, Adams et al. 2016 (r2=1)	HC + ICV, Haworth et al. 2019 (r2=1)	Fusiform, Grasby et al. 2020 (r2=1)	Frontalpole, Grasby et al. 2020 (r2=1)
60	rs8079695	ICV, Adams et al. 2016 (r2=1)	Fusiform, Grasby et al. 2020 (r2=1)		
63	rs148340480	Paracentral, Grasby et al. 2020 (r2=0.805)			
67	rs10483213	HC + ICV, Haworth et al. 2019 (r2=1)			

Table S14. Genetic correlation between the human head size and neuropsychiatric traits. *Related to Figure S3, Figure 1C and STAR Methods' section "Genetic correlations".*
Abbreviations: P - P-value; Rg - genetic correlation estimate; SE - standard error.
Bold p-values represent Bonferroni significant correlations (P < (0.05/16))

		**Model 1 - full set**			**Model 1 - new set**		
**Trait**	**Category**	**Rg**	**SE**	**P**	**Rg**	**SE**	**P**
Educational attainment	Cognitive functioning	0.2599	0.0210	**3.98E-35**	0.2356	0.0248	**1.90E-21**
General cognitive function	Cognitive functioning	0.2340	0.0240	**2.06E-22**	0.2251	0.0272	**1.14E-16**
All stroke	Neurological disorders	-0.0295	0.0438	5.00E-01	0.0003	0.0517	9.95E-01
Alzheimer's disease	Neurological disorders	-0.0950	0.0743	2.01E-01	-0.1506	0.0875	8.54E-02
Frontotemporal dementia	Neurological disorders	0.1335	0.1569	3.95E-01	0.2343	0.1886	2.14E-01
Parkinson's disease	Neurological disorders	0.2035	0.0446	**5.07E-06**	0.2049	0.0487	**2.54E-05**
Anorexia nervosa	Psychiatric traits	0.0864	0.0515	9.35E-02	0.0187	0.0656	7.76E-01
Attention-deficit hyperactivity disorder	Psychiatric traits	-0.1804	0.0357	**4.46E-07**	-0.1421	0.0410	**5.37E-04**
Autism spectrum disorder	Psychiatric traits	0.0746	0.0505	1.40E-01	0.1006	0.0671	1.34E-01
Bipolar disorder	Psychiatric traits	0.0464	0.0293	1.14E-01	0.0521	0.0368	1.57E-01
Extraversion	Psychiatric traits	-0.0557	0.0621	3.70E-01	-0.0664	0.0722	3.58E-01
Insomnia	Psychiatric traits	-0.1899	0.0443	**1.78E-05**	-0.1759	0.0509	**5.47E-04**
Major depressive disorder	Psychiatric traits	-0.1098	0.0300	**2.57E-04**	-0.0983	0.0358	5.98E-03
Neuroticism	Psychiatric traits	-0.1101	0.0319	**5.43E-04**	-0.0887	0.0355	1.23E-02
Obsessive compulsive disorder	Psychiatric traits	0.1298	0.0526	1.35E-02	0.1733	0.0634	6.26E-03
Schizophrenia	Psychiatric traits	-0.0171	0.0287	5.51E-01	-0.0363	0.0336	2.80E-01

Table S15. Kyoto Encyclopaedia of Genes and Genomes (KEGG) pathway analysis. *Related to Figure 3A.*

GeneSet	N_genes	N_overlap	p	adjP	genes	link
KEGG ENDOMETRIAL CANCER	52	7	4.71E-06	0.000321	AKT3, PTEN, TP53, TCF7L1, MAPK1, APC, FOXO3	http://www.broadinstitute.org/gsea/msigdb/cards/KEGG_ENDOMETRIAL_CANCER
KEGG CELL CYCLE	124	10	5.16E-06	0.000321	CCND2, CDKN1B, ESPL1, CDK2, TP53, MCM2, STAG1, TFDP2, ATR, CDK6	http://www.broadinstitute.org/gsea/msigdb/cards/KEGG_CELL_CYCLE
KEGG PATHWAYS IN CANCER	325	16	5.95E-06	0.000321	WNT2B, AKT3, PTEN, CDKN1B, CDK2, IGF1, TP53, FZD2, WNT3, TCF7L1, PLCG1, MAPK1, LAMB2, APC, CDK6, PTCH1	http://www.broadinstitute.org/gsea/msigdb/cards/KEGG_PATHWAYS_IN_CANCER
KEGG BASAL CELL CARCINOMA	55	7	6.90E-06	0.000321	WNT2B, TP53, FZD2, WNT3, TCF7L1, APC, PTCH1	http://www.broadinstitute.org/gsea/msigdb/cards/KEGG_BASAL_CELL_CARCINOMA
KEGG PROSTATE CANCER	88	8	1.92E-05	0.000658	AKT3, PTEN, CDKN1B, CDK2, IGF1,	http://www.broadinstitute.org/gsea/msigdb/cards/KEGG_PROSTATE_CANCER

					TP53, TCF7L1, MAPK1	
KEGG GLIOMA	65	7	2.12E-05	0.000658	AKT3, PTEN, IGF1, TP53, PLCG1, MAPK1, CDK6	http://www.broadinstitute.org/gsea/msigdb/cards/KEGG_GLIOMA
KEGG P53 SIGNALING PATHWAY	68	7	2.86E-05	0.000759	PTEN, CCND2, CDK2, IGF1, TP53, ATR, CDK6	http://www.broadinstitute.org/gsea/msigdb/cards/KEGG_P53_SIGNALING_PATHWAY
KEGG NON SMALL CELL LUNG CANCER	54	6	6.99E-05	0.001624	AKT3, TP53, PLCG1, MAPK1, FOXO3, CDK6	http://www.broadinstitute.org/gsea/msigdb/cards/KEGG_NON_SMALL_CELL_LUNG_CANCER
KEGG SMALL CELL LUNG CANCER	84	7	0.000112	0.002313	AKT3, PTEN, CDKN1B, CDK2, TP53, LAMB2, CDK6	http://www.broadinstitute.org/gsea/msigdb/cards/KEGG_SMALL_CELL_LUNG_CANCER
KEGG MELANOMA	71	6	0.000322	0.005995	AKT3, PTEN, IGF1, TP53, MAPK1, CDK6	http://www.broadinstitute.org/gsea/msigdb/cards/KEGG_MELANOMA
KEGG WNT SIGNALING PATHWAY	148	8	0.000722	0.012211	WNT2B, LRP5, CCND2, TP53, FZD2, WNT3, TCF7L1, APC	http://www.broadinstitute.org/gsea/msigdb/cards/KEGG_WNT_SIGNALING_PATHWAY
KEGG ERBB SIGNALING PATHWAY	87	6	0.000956	0.014823	AKT3, CDKN1B, ERBB3, PLCG1, MAPK1, NCK1	http://www.broadinstitute.org/gsea/msigdb/cards/KEGG_ERBB_SIGNALING_PATHWAY

KEGG COLORECTAL CANCER	62	5	0.001266	0.018109	AKT3, TP53, TCF7L1, MAPK1, APC	http://www.broadinstitute.org/gsea/msigdb/cards/KEGG_COLORECTAL_CANCER
KEGG CHRONIC MYELOID LEUKEMIA	73	5	0.002618	0.034778	AKT3, CDKN1B, TP53, MAPK1, CDK6	http://www.broadinstitute.org/gsea/msigdb/cards/KEGG_CHRONIC_MYELOID_LEUKEMIA

Table S22. Genetic correlation between the human head size and different cancer types. *Related to STAR Methods' section "Genetic correlations".*

Abbreviations: P - P-value; Rg - genetic correlation estimate; SE - standard error.

Bold p-values represent nominally significant correlations (P < 0.05). No correlations survived Bonferroni multiple testing correction (P < (0.05/15)).

		**Model 1 - full set**			**Model 1 - new set**		
**Trait**	**Category**	**Rg**	**SE**	**P**	**Rg**	**SE**	**P**
Breast cancer Zhang et al.	Cancer	-0.0117	0.0293	6.89E-01	-0.0173	0.0309	5.77E-01
Ovarian cancer Phelan et al.	Cancer	-0.1831	0.0646	**4.62E-03**	-0.1637	0.0682	**1.63E-02**
Prostate cancer Schumacher et al.	Cancer	-0.0033	0.0379	9.30E-01	-0.0520	0.0405	1.99E-01
Bladder cancer Rashkin et al.	Cancer	-0.0699	0.1101	5.26E-01	-0.1205	0.1258	3.38E-01
Breast cancer Rashkin et al.	Cancer	0.0199	0.0433	6.45E-01	0.0430	0.0451	3.41E-01
Cervical cancer Rashkin et al.	Cancer	-0.0962	0.0747	1.97E-01	-0.0929	0.0830	2.63E-01
Colon cancer Rashkin et al.	Cancer	-0.0023	0.0770	9.76E-01	0.0449	0.0789	5.69E-01
Endometrial cancer Rashkin et al.	Cancer	-0.0297	0.0795	7.09E-01	0.0518	0.0877	5.55E-01
Oesphageal and gastric cancer Rashkin et al.	Cancer	0.0800	0.1399	5.68E-01	-0.0038	0.1548	9.81E-01
Kidney cancer Rashkin et al.	Cancer	-0.0525	0.0850	5.37E-01	-0.1515	0.0907	9.51E-02
Lung cancer Rashkin et al.	Cancer	0.0952	0.0590	1.07E-01	0.0813	0.0720	2.59E-01
Malignant melanoma Rashkin et al.	Cancer	0.0519	0.0905	5.66E-01	0.0013	0.1044	9.90E-01
Non-Hodgkin lymphoma Rashkin et al.	Cancer	0.0774	0.2177	7.22E-01	0.1016	0.2201	6.45E-01
Prostate cancer Rahskin et al.	Cancer	0.0699	0.0417	9.40E-02	0.0564	0.0477	2.37E-01
Rectal cancer Rashkin et al.	Cancer	-0.0445	0.0859	6.04E-01	-0.0072	0.0973	9.41E-01

Table S24. Enrichment of head size GWAS for brain cell types based on human scRNA-seq data. *Related to Figure S2.*

Cell type or subtype	(Sub)type	Enrichment	Enrichment SE	Enrichment P	P	FDR
Cell type	IPC	2.023	0.1437	3.14E-11	0.0005	0.0050
Cell type	Endothelial	1.742	0.1226	8.30E-09	0.7043	0.7825
Cell type	Red blood cells	1.672	0.1191	6.85E-08	0.0226	0.1131
Cell type	Microglia	1.693	0.1359	9.06E-07	0.3655	0.6092
Cell type	Mural	1.611	0.1229	1.21E-06	0.9050	0.9050
Cell type	Radial glia	1.600	0.1216	2.51E-06	0.1545	0.3863
Cell type	Inhibitory neuron	1.406	0.1018	0.000112	0.2911	0.5822
Cell type	Excitatory neuron	1.291	0.0980	0.003729	0.1111	0.3703
Cell type	OPC	1.240	0.0934	0.010566	0.5065	0.6968
Cell subtype	vRG	2.149	0.1543	5.61E-12	0.0009	0.0090
Cell subtype	IPC.div1	2.243	0.1608	1.05E-11	0.0002	0.0064
Cell subtype	tRG	1.904	0.1338	3.51E-11	0.0423	0.1588
Cell subtype	IPC.new	2.053	0.1491	1.05E-10	0.0042	0.0313
Cell subtype	Newborn_Neuron	1.844	0.1236	2.26E-10	0.0006	0.0090
Cell subtype	Endothelial	1.807	0.1260	1.48E-09	0.4992	0.6511
Cell subtype	Layer_VI_Occipital	1.811	0.1338	1.99E-09	0.0869	0.2606
Cell subtype	early_RG	1.920	0.1490	3.11E-09	0.0831	0.2606
Cell subtype	IPC.div2	1.968	0.1533	3.46E-09	0.0271	0.1356
Cell subtype	PFC	1.779	0.1330	1.11E-08	0.1159	0.3074
Cell subtype	IPCnewborn	2.000	0.1638	1.12E-08	0.0103	0.0620
Cell subtype	oRG	1.711	0.1273	3.53E-08	0.1873	0.4015
Cell subtype	SST.MGE1	1.610	0.1131	2.26E-07	0.2219	0.4160
Cell subtype	oRGAstrocyte	1.641	0.1269	5.81E-07	0.2835	0.4476
Cell subtype	Microglia	1.738	0.1434	6.50E-07	0.8210	0.8493

Cell subtype	Mural	1.638	0.1285	1.60E-06	0.8765	0.8765
Cell subtype	late_RG	1.495	0.1042	4.33E-06	0.7382	0.7909
Cell subtype	Upper_Layer_Occipital	1.529	0.1152	2.02E-05	0.1383	0.3192
Cell subtype	Cajal_Retzius	1.347	0.1047	0.000944	0.4594	0.6265
Cell subtype	Parietal_and_Temporal	1.314	0.0957	0.001451	0.1229	0.3074
Cell subtype	Upper_Layer_PFC	1.356	0.1170	0.002683	0.6781	0.7557
Cell subtype	OPC	1.293	0.1004	0.003865	0.6801	0.7557
Cell subtype	Upper_Layer	1.305	0.1052	0.005159	0.2009	0.4019
Cell subtype	Deep_Layer	1.270	0.1072	0.012312	0.6409	0.7557
Cell subtype	MGE2	1.209	0.0848	0.016121	0.2697	0.4476
Cell subtype	Layer_IV	1.225	0.0951	0.020491	0.4418	0.6265
Cell subtype	Layer_VI_Pan.area	1.177	0.0884	0.045774	0.3694	0.5541

Table S25. Enrichment of gene sets for brain cell types based on human scRNA-seq data. *Related to Figure 4E.*

**Gene list**	**Cell type**	**Fold change**	**P**	**FDR**
Head size intragenic lead variant genes	Endothelial	1.1362	0.4530	0.8965
Head size intragenic lead variant genes	Excitatory neuron	0.9083	0.6545	0.9999
Head size intragenic lead variant genes	Inhibitory neuron	0.9090	0.6539	0.9896
Head size intragenic lead variant genes	IPC	2.4997	3.31E-03	0.0088
Head size intragenic lead variant genes	Microglia	0.2271	0.9904	0.9973
Head size intragenic lead variant genes	Mural	1.1354	0.4536	0.9997
Head size intragenic lead variant genes	OPC	0.6808	0.8308	0.9996
Head size intragenic lead variant genes	Radial glia	0.9090	0.6539	0.9908
Head size intragenic lead variant genes	Red blood cells	1.5907	0.1454	0.4105
Head size intragenic genes	Endothelial	1.2421	0.1827	0.7306
Head size intragenic genes	Excitatory neuron	0.8688	0.7491	0.9999
Head size intragenic genes	Inhibitory neuron	0.9937	0.5497	0.9896
Head size intragenic genes	IPC	1.6147	9.58E-03	0.0192
Head size intragenic genes	Microglia	0.6206	0.9669	0.9973
Head size intragenic genes	Mural	0.6206	0.9669	0.9997
Head size intragenic genes	OPC	0.5582	0.9843	0.9996
Head size intragenic genes	Radial glia	0.8694	0.7482	0.9908
Head size intragenic genes	Red blood cells	1.1800	0.2565	0.4105
Head size genes 10kb distance	Endothelial	0.9919	0.5486	0.8965
Head size genes 10kb distance	Excitatory neuron	0.7930	0.8901	0.9999
Head size genes 10kb distance	Inhibitory neuron	0.9919	0.5486	0.9896
Head size genes 10kb distance	IPC	1.6664	6.43E-04	0.0026
Head size genes 10kb distance	Microglia	1.0705	0.3836	0.9973
Head size genes 10kb distance	Mural	0.7930	0.8901	0.9997
Head size genes 10kb distance	OPC	0.5547	0.9962	0.9996
Head size genes 10kb distance	Radial glia	0.9919	0.5486	0.9908

Head size genes 10kb distance	Red blood cells	0.9522	0.6318	0.8424
Head size genes 100kb distance	Endothelial	0.9869	0.5603	0.8965
Head size genes 100kb distance	Excitatory neuron	0.7267	0.9753	0.9999
Head size genes 100kb distance	Inhibitory neuron	1.0128	0.4917	0.9896
Head size genes 100kb distance	IPC	1.3245	0.0226	0.0361
Head size genes 100kb distance	Microglia	1.0381	0.4255	0.9973
Head size genes 100kb distance	Mural	0.7786	0.9441	0.9997
Head size genes 100kb distance	OPC	0.5706	0.9992	0.9996
Head size genes 100kb distance	Radial glia	0.8311	0.8883	0.9908
Head size genes 100kb distance	Red blood cells	0.9090	0.7511	0.8584
Head size genes 1Mb distance	Endothelial	0.8962	0.9124	0.9949
Head size genes 1Mb distance	Excitatory neuron	0.9034	0.8969	0.9999
Head size genes 1Mb distance	Inhibitory neuron	0.9430	0.7756	0.9896
Head size genes 1Mb distance	IPC	1.0443	0.3040	0.3474
Head size genes 1Mb distance	Microglia	0.9112	0.8778	0.9973
Head size genes 1Mb distance	Mural	0.9190	0.8564	0.9997
Head size genes 1Mb distance	OPC	0.7471	0.9996	0.9996
Head size genes 1Mb distance	Radial glia	0.8417	0.9805	0.9908
Head size genes 1Mb distance	Red blood cells	1.0599	0.2403	0.4105
OMIM macrocephaly genes	Endothelial	0.4761	0.9583	0.9949
OMIM macrocephaly genes	Excitatory neuron	0.7930	0.7689	0.9999
OMIM macrocephaly genes	Inhibitory neuron	0.3174	0.9896	0.9896
OMIM macrocephaly genes	IPC	1.1110	0.4444	0.4444
OMIM macrocephaly genes	Microglia	0.7930	0.7689	0.9973
OMIM macrocephaly genes	Mural	0.6344	0.8875	0.9997
OMIM macrocephaly genes	OPC	0.9509	0.6136	0.9996
OMIM macrocephaly genes	Radial glia	1.1110	0.4444	0.9908
OMIM macrocephaly genes	Red blood cells	1.7458	0.0464	0.3715
OMIM microcephaly genes	Endothelial	0.6231	0.9949	0.9949

OMIM microcephaly genes	Excitatory neuron	0.5930	0.9973	0.9999
OMIM microcephaly genes	Inhibitory neuron	1.0681	0.3630	0.9896
OMIM microcephaly genes	IPC	1.6022	3.26E-04	0.0026
OMIM microcephaly genes	Microglia	0.5930	0.9973	0.9973
OMIM microcephaly genes	Mural	0.5040	0.9997	0.9997
OMIM microcephaly genes	OPC	0.5333	0.9994	0.9996
OMIM microcephaly genes	Radial glia	0.6527	0.9908	0.9908
OMIM microcephaly genes	Red blood cells	1.1571	0.1876	0.4105
COSMIC tier 1 cancer genes	Endothelial	1.2939	0.0172	0.1373
COSMIC tier 1 cancer genes	Excitatory neuron	0.5486	0.9999	0.9999
COSMIC tier 1 cancer genes	Inhibitory neuron	0.7646	0.9734	0.9896
COSMIC tier 1 cancer genes	IPC	1.2155	0.0603	0.0805
COSMIC tier 1 cancer genes	Microglia	0.9796	0.5847	0.9973
COSMIC tier 1 cancer genes	Mural	1.3910	0.0025	0.0203
COSMIC tier 1 cancer genes	OPC	0.7636	0.9740	0.9996
COSMIC tier 1 cancer genes	Radial glia	1.0391	0.4044	0.9908
COSMIC tier 1 cancer genes	Red blood cells	0.8430	0.9018	0.9018

Supplementary Figures

Figure S1. Genetic correlations with neuropsychiatric traits. *Related to Figure 1C.*

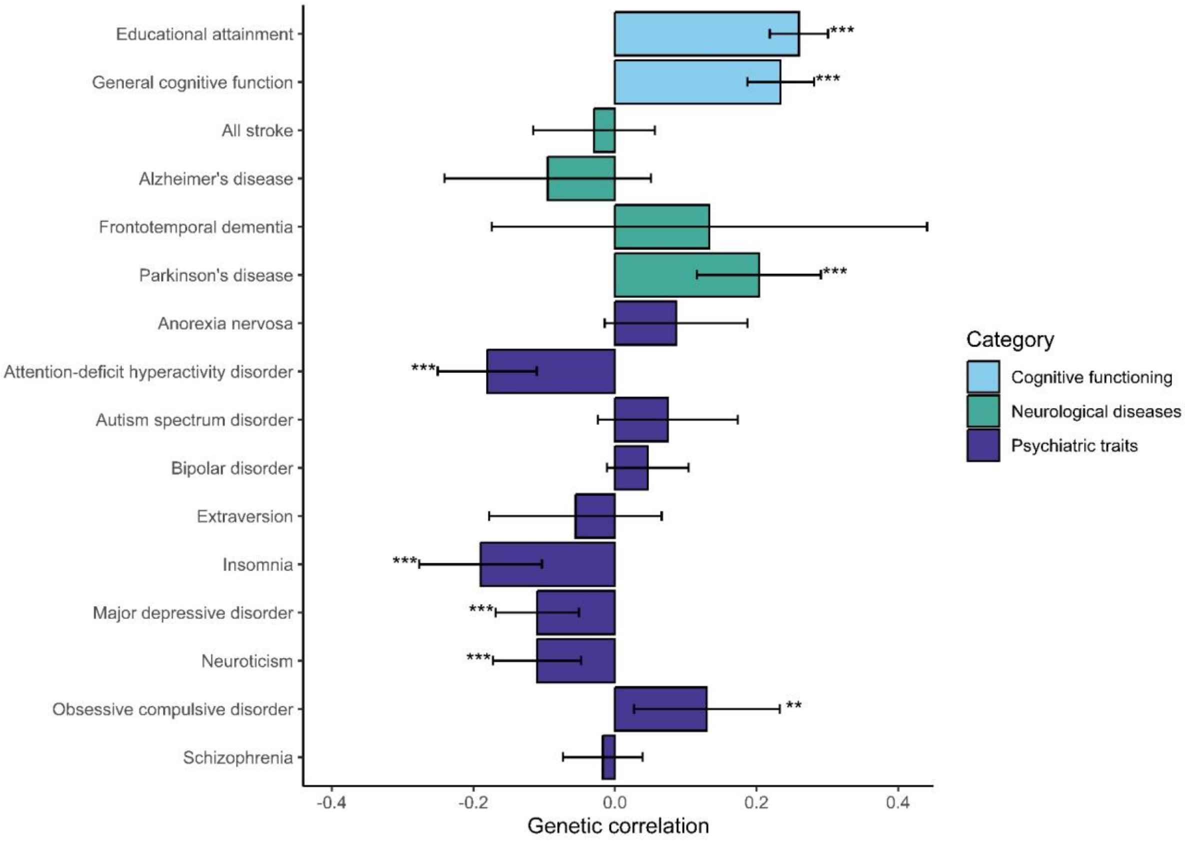

Genetic correlations of the human head size with cognitive functioning, neurological diseases and psychiatric traits. Analyses were performed in both the complete GWAS set, and in the GWAS set with only newly contributing studies. Significant results are denoted by asterisks: *P<0.05; **P<0.017 (0.05/3); ***P<0.003 (0.05/16).

Figure S2. Enrichment of expression of human head size GWAS genes in cell subtypes in the human cortical brain, derived from single-cell RNA sequencing data. *Related to Figure 4.*

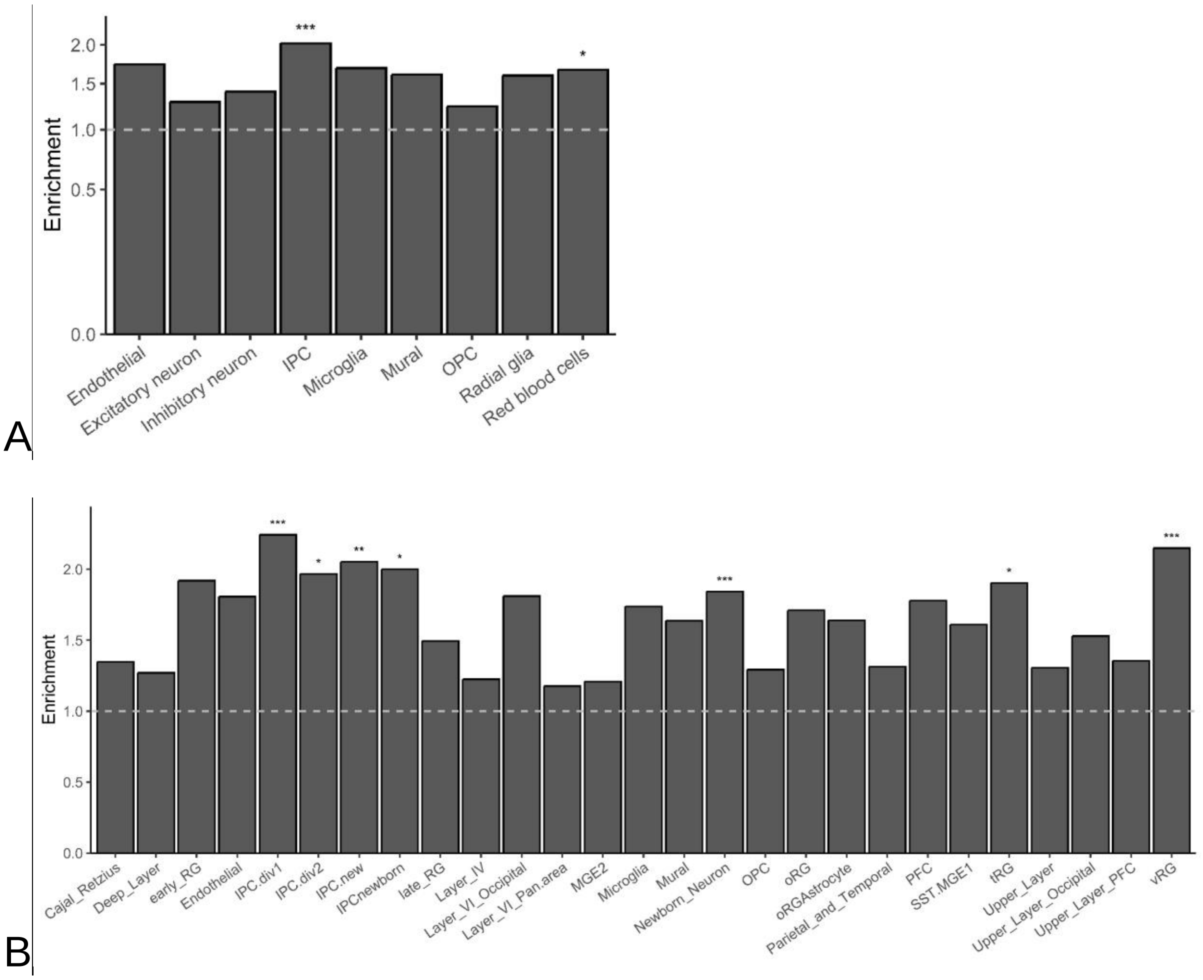

(A) Enrichment of expression of human head size GWAS genes in cell types of the human cortical brain, derived from single-cell RNA sequencing data. Significant results are denoted by asterisks: **P*<0.05; ***FDR*<0.05; ****P*<0.006 (0.05/9).

(B) Enrichment of expression of human head size GWAS genes in cell subtypes in the human cortical brain, derived from single-cell RNA sequencing data. Significant results are denoted by asterisks: **P*<0.05; ***FDR*<0.05; ****P*<0.002 (0.05/27).
